# Iconic but Invasive: The Public Perception of the Chinese Windmill Palm (*Trachycarpus fortunei*) in Switzerland

**DOI:** 10.1007/s00267-022-01646-3

**Published:** 2022-04-26

**Authors:** Matteo Tonellotto, Vincent Fehr, Marco Conedera, Marcel Hunziker, Gianni Boris Pezzatti

**Affiliations:** 1 Community Ecology Research Unit, Swiss Federal Institute for Forest, Snow and Landscape Research WSL, A Ramèl 18, 6593 Cadenazzo, Switzerland; 2 Economics and Social Sciences Research Unit, Swiss Federal Institute for Forest, Snow and Landscape Research WSL, Zürcherstrasse 111, 8903 Birmensdorf, Switzerland

**Keywords:** Invasive alien species, Ornamental plants, Charismatic invasive species, Public awareness, Invasive species management

## Abstract

Biological invasions strongly increased during the last centuries and are challenging environmental managers worldwide. In this context, public acceptance of management measures is a key factor determining the long-term success of the control of invasive species. However, in the case of charismatic and iconic invasive species, the public has often been unwilling to accept strict management measures. Here, we studied the public perception of the Chinese windmill palm (*Trachycarpus fortunei*) in Switzerland, which is declared as invasive in southern Switzerland but also recognized as iconic. We conducted a nation-wide online survey in the multilingual and multicultural context of Switzerland, investigating the influence of social and cultural factors on the knowledge of, the attitude toward, and the willingness to control the invasive *T. fortunei*. Results confirm that the knowledge and perception of invasive plants have a strong social and cultural component and may vary greatly as a function of the cultural background, education level, age, and other social characteristics. Furthermore, information on the invasiveness of the focal species provided during the survey significantly affected informants’ perceptions, which are closely related to the acceptance of possible management and control measures. This allows us to highlight the importance of a holistic approach that includes targeted public information when dealing with biological invasions, especially in the case of charismatic and iconic species. Based on the obtained results, we suggest avenues for refining management and control strategies of *T. fortunei* in Switzerland, many of which generally applicable to other cases of invasive species.

## Introduction

The rate of introductions of species to regions beyond their native range has strongly increased during the last centuries worldwide (Seebens et al. 2017). The pathways and causes of such translocations of species strongly depend on trends in human mobility and trade (e.g., Meyerson and Mooney 2007) and vary greatly over time and space, as well as among taxonomic groups (Fry 2013). A proportion of these introduced species manages to go through the discrete and successive phases of survival, establishment, and spontaneous dispersal, eventually becoming invasive and causing ecological and/or socio-economic damages (Alpert et al. 2000; Lonsdale 1999). To prevent further impacts a wide range of different management approaches has been developed to control invasive species (Ruiz and Carlton 2003).

However, the control of invasive species is not always straightforward as the support by the public may lack as it may be for instance the case for invasive species that are perceived as charismatic or iconic (Jarić et al. 2020). Charismatic species can be broadly defined as species that evoke responses in humans (usually positive) and affect people’s perceptions, attitudes, and behaviors toward them (Albert et al. 2018; Jarić et al. 2020). Iconic species share most characteristics with charismatic species but are also of cultural importance, venerated by the public and often represent specific geographical places (Albert et al. 2018, Horsley et al. 2020). In conservation science, the concept of charismatic species has a long tradition and represents a central trait for flagship species (Home et al. 2009). Including socio-ecological approaches to appropriately investigate the phenomenon of charismatic and iconic invasive species has however recently attracted attention also in invasion science (Jarić et al. 2020). In this respect, the general public’s knowledge, preferences, and attitudes toward invasive species may be highly heterogeneous and volatile over time depending on their education level, economic interests, environmental awareness, and cultural backgrounds (Adams et al. 2011; Hunziker et al. 2008; Sharp et al. 2011). Clarifying social aspects such as interests, values, perceptions, and possible conflicts associated with invasive alien species represents a fundamental step toward a broad public support for possible management and control measures (Junge et al. 2019; Kueffer and Kull 2017).

**Fig. 1:**
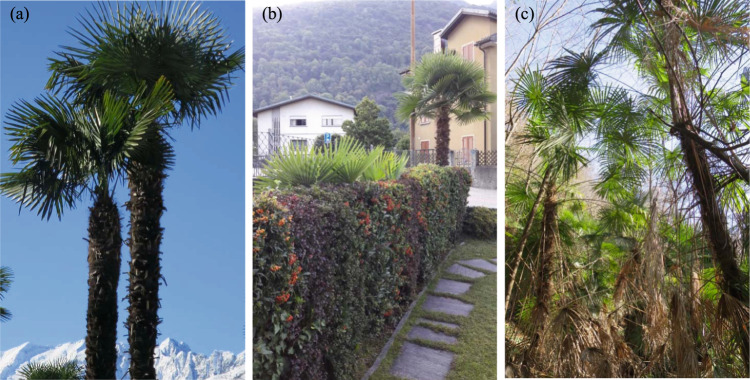
Individuals of *T. fortunei* in the Insubric region: **a** cultivated, **b** spreading in a hedge of a private garden, and **c** spreading inside a forest

In this study, we investigated the public perception of the Chinese windmill palm (*Trachycarpus fortunei* (Hook.) H. Wendl.) in Switzerland. *T. fortunei* can be considered a textbook example of an iconic but invasive alien plant species in southern Switzerland. Introduced in the early 18th century south of the Alps as a highly valued, exotic rarity (Walther 2002), it became one of the most popular ornamental plants in southern Switzerland. Its popularity is likely owed to its prominent, subtropical look within a landscape dominated by native deciduous trees (Fig. 1). *T. fortunei* has become widely used as a symbol for the region of southern Switzerland. This is well reflected by the frequent use of palms in marketing campaigns by the tourism industry to attract tourists (see Supplementary Materials S1). The strong association of *T. fortunei* with Ticino (the name of the canton of southern Switzerland) is further illustrated by the German common name “Tessinerpalme” (“palm from Ticino”) for *T. fortunei* (Info Flora 2021). Meanwhile, *T. fortunei* is considered part and parcel of the regional cultural heritage of southern Switzerland (Vogelaar and Hale 2013) and can be designated as an iconic species within this region.

However, during the second half of the twentieth century, *T. fortunei* was reported to naturalize south of the Alps (Walther 2002) following changes in climate (Walther et al. 2007), disturbance regimes (Grund et al. 2005), and land-use (Carraro et al. 1999; Conedera et al. 2018). Since the early 2000s, *T. fortunei* is building monodominant stands in certain areas in southern Switzerland where it is suspected to suppress the recruitment of native, woody species by competing for space and light (Info Flora 2021). Beside the ecological impacts, it is assumed to weaken the protection forests, resulting in an increase of gravitational hazards (Info Flora 2021, Pezzatti et al. 2021; Zanelli et al. 2006). Consequently, in 2014, it was officially classified as an invasive species in southern Switzerland (Info Flora 2014). In Switzerland north of the Alps, *T. fortunei* is still relatively rarely cultivated and confined to the mildest urban or near-lake sites where it sporadically escapes from cultivations but does not build self-sustaining populations yet (Info Flora 2021).

The differing extent of spread and concern about *T. fortunei* north and south of the Alps combined with the cultural-linguistic diversity of the country, make Switzerland an excellent model system to study the general public’s knowledge and perception (i.e., preferences and attitudes toward control measures) of an iconic and invasive plant as a function of their cultural background, which, in turn, is expected to affect the acceptance of control measures. To this purpose, we conducted a nation-wide online survey with the overall aim of assessing the public’s knowledge (i.e., information level in terms of the species and awareness of its invasion potential) and perception (i.e., general attitude toward the species, related word association) of *T. fortunei* and to capture the public’s acceptance for different measures to reduce *T. fortunei*’s spreading potential (e.g., removal of female inflorescences, substituting *T. fortunei* with non-invasive plant species of similar appearance). We then quantified if the detected differences can be explained by (i) linguistic (and related cultural) background (ii) type of residence (e.g., urban vs. rural or mountain areas), and (iii) the ownership of a garden suitable for palms. In a second step, we related the willingness to accept proposed control measures on *T. fortunei* as a function of the collected data in terms of information level, perception, and preference. Finally, we discuss the obtained results in the light of possible recommendations for refining control strategies of iconic invasive species in the frame of a comprehensive and integrative management.

## Material and Methods

### Study Area

**Fig. 2:**
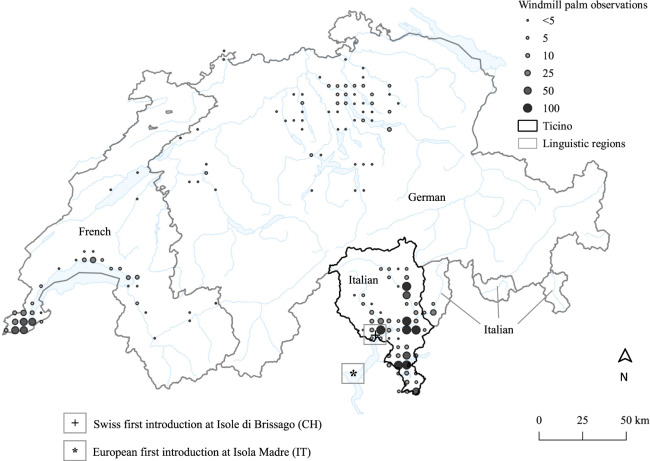
Current distribution of *T. fortunei* in Switzerland (Info Flora 2021). *T. fortunei* is the most widespread in the Canton of Ticino (Italian speaking). The sites of first introductions of *T. fortunei* are marked for Europe (*) and Switzerland (+). As German is the second language for most of the Romansch speaking inhabitants, this language region was merged with the German language region

Switzerland is a Central-European country covering an area of 41,285 km^2^ that is topographically divided by the European Alps into a northern (German- and French-speaking) and a southern (Italian-speaking) part (Fig. 2). The climatic and socio-cultural conditions are correspondingly highly heterogeneous. The lowlands north of the Alps have a temperate climate with cold winters (i.e., monthly average temperature of coldest month (January) = 0.3 °C, number of freezing days = 76, number of ice days = 23.7; climate normal of 1981–2010 at the MeteoSwiss station Zurich-Fluntern). Frost periods of several days frequently occur, where temperatures can drop below −12 °C, limiting the cultivation and the spread of *T. fortunei*. According to Larcher and Winter (1981), leaves of adult individuals of *T. fortunei* are killed at −14 °C whilst leaves of seedlings are killed at −12 °C. However, first leave damages usually occur at −11 and −9 °C for adults and seedlings, respectively. Thus, the cultivation of *T. fortunei* north of the Alps is restricted to the mildest parts close to lakes and urban areas. In contrast, the lowlands south of the Alps are characterized by a warm-temperate climate with mild winters (i.e., monthly average temperature of coldest month (January) = 3.2 °C, number of freezing says = 28.1, number of ice days = 0.7; climate normal of 1981–2010 at the Swiss Meteorological Station Lugano). Since the year 1960, frost periods lasting several days were nearly absent and temperatures never dropped below −9 °C. Thus, winter temperatures do not constitute a limiting factor for the cultivation and the spread of *T. fortunei* in the lowlands south of the Alps.

From the socio-cultural point of view, the 8.6 million permanent residents identify one of the three official languages as their first language, namely German (62%), French (23%) and Italian (8%, mostly located south of the Alps). German is the second language for most of the 0.5% of the population for whom the fourth national language (Romansch) is their first language. The remaining 6.5% comprise other languages, which are spoken by foreign residents (22.5%) a portion of which do not speak any of the official languages (FSO 2020).

### Sampling Design and Data Collection

We conducted a nationwide online survey with the professional support of the survey programming and hosting service group Bilendi (https://www.bilendi.co.uk). The target sample consisted of 2003 participants proportionally subdivided into gender and age classes according to data from the Swiss Federal Statistical Office (FSO 2020) (Table 1). An oversampling of Italian-speaking participants was forced (33% instead of 8%) to increase the likelihood of statistically assured results for the southern (Italian-speaking) part of the country, where *T. fortunei* is presently causing the greatest concerns calling for urgent control measures. The remaining participants were subdivided to match the true population distribution between native German (46%) and French-speaking (21%) individuals, respectively (Table 1). After a pre-test involving 20 selected participants, the online survey was launched on August 17, 2020, and closed one week later on August 24, 2020.

**Table 1:** Sampling structure of participants, categories, and their respective proportions

Characteristic of participants	Categories	Proportion (%)
Native language^a^	Italian	33.0
	French	21.0
	German	46.0
Age class^a^	18–29	17.0
	30–39	17.0
	40–49	17.0
	50–65	27.0
	>65	22.0
Gender^a^	Male	49.0
	Female	51.0
Zone of residence	Urban space: city center	22.0
	Urban space: suburbs	46.1
	Rural or mountain areas	31.9
Last qualification obtained	None	0.3
	Elementary or middle school	3.9
	Vocational education and training schools or specialized schools	35.4
	High school	13.0
	Professional education and training colleges	15.6
	Universities of applied sciences or teacher education	12.9
	Universities	18.9
Current occupation	Training	9.1
	Primary sector	11.8
	Secondary sector	13.5
	Tertiary sector	28.5
	Housewife/husband	6.7
	Retired	24.4
	Unemployed	6.0
Profession related to the environment	Yes	12.9
	No	87.1
Member of environmental association/organization	Yes	9.6
	No	90.4
*T. fortunei* in primary residences	Italian native speakers	23.1
	French native speakers	6.8
	German native speakers	6.4
*T. fortunei* in primary residences	In Ticino	27.5
	North of the Alps	6.1
*T. fortunei* in secondary residences	In Ticino	17.9
	North of the Alps	9.1

^a^Sample structure of these parameters has been defined by the study design

### Questionnaire

The questionnaire was conceived in Italian and translated into the two other target languages: French and German (see Supplementary Materials S2, which also includes an English version).

In total, the questionnaire consisted of 50 items, 24 of which asked for personal information to use as predictors (six at the beginning to match the participants’ quotas, twelve regarding the residences and gardens of participants, and six at the end retrieving standard personal data). Three questions asked whether and where the informant has seen *T. fortunei*. Regarding dependent variables, four questions focused on perceptions of *T. fortunei*, one on the level of knowledge of invasive alien plants, six on the level of knowledge of the species, six on the preferences of the participants, and five on management options of *T. fortunei*. The remaining question asked the participants to freely write a single word that they associate with the species. To assess how the information level of the participant on the invasion potential of *T. fortunei* affected the participant’s attitude about the species, selected preference questions were repeated after providing the participant with written information on *T. fortunei*’s Asiatic origin, invasive behavior, blacklist status (designation as an invasive species by an official authority), potential environmental impacts and possible control measures and on the federal government’s and canton’s costs for nature conservation and forest biodiversity.

Most of the thematic questions (19) consisted of single-choice queries, of which a small number required a five-step rating (six questions) or a Likert scale approach (six questions) (Moser and Kalton 1971). To help participants evaluating the species and the landscapes where it grows, two questions belonging to the preference category used pictures, the first representing three landscape pairs from which participants selected their preference. The second was an implicit-association test (IAT) asking the participants to indicate, within a time limit of eight seconds per image, whether they would accept the shown plant individual as an option to replace *T. fortunei*. The shown plant individuals belonged to seven species and four morphological groups (fan palms, feather palms, yucca-like plants and banana plants) and species/individuals of the first three groups showed a varying amount of dead leaves (a proxy for tidiness) (see Supplementary Materials S3).

To assess participants’ willingness to reduce the spread of *T. fortunei* in Swiss natural environments, participants were finally asked to evaluate six possible management approaches for *T. fortunei* along a stringency gradient going from light (monitoring and information campaigns) to medium (eradication from natural environments, prohibition in public places) to drastic (prohibition of any kind of trade and planting) options.

### Data Analysis

Free word associations in the three original languages were grouped into 46 broader subject categories, labeled with a representative English collective term (see Supplementary Materials S4 for details), and their frequency analyzed with respect to the linguistic groups.

Preliminary homogeneity tests revealed the lack of similarity of variances (*p* > 0.05) for most of the quasi-metric variables, preventing us from proceeding to a one-way ANOVA test. We thus analyzed the strength of associations among our variables using a non-parametric Kruskal–Wallis test (see Supplementary Materials S5).

To identify the relationship between the answers provided in terms of perception, preference, and knowledge, and the agreement the corresponding informant expresses with respect to the proposed management options, we used the answers to the related questions (i.e., Q27, Q31, Q32, Q33 for perception, Q11 for preference, Q7, Q24, Q25, Q29, Q33, Q35 for knowledge, see Supplementary Materials S2) as explanatory variables to analyze their effect on the acceptance of the proposed management options (i.e., from 1 = inappropriate to 5 = appropriate; Q44). We then expressed the extent of the influence of the selected questions as unit rates of change. The strengths of the obtained relationships were then analyzed by training linear regression models on each combination of question and management option, with the slope coefficients representing the rates of change, and their *p* values representing the significance measure. Statistical analyses were carried out using RStudio (version 1.3.1093, R Core Team 2021) and IBM SPSS Statistics software (version 26, IBM Corp 2021).

## Results

### Characteristics of the Sample

The 2003 Swiss residents participating to the survey met the planned distribution in terms of language, age class, and gender (Table 1). Just 43.5% of the public declared to have already seen *T. fortunei* in Switzerland. Residents of Ticino in southern Switzerland more often have *T. fortunei* in their gardens (27.5%) than residents of other cantons (6.1%). Among owners of *T. fortunei* in primary residences, 69.4% reported possessing between one and three individuals, whereas 24.4 and 6.3% possess four to ten or more than ten individuals, respectively. Of the 15.3% owning a secondary residence, 12.1% (*n* = 28) indicated to have at least one individual of *T. fortunei* in the garden of the secondary residence. Of the secondary residences located in Ticino, 17.9% have *T. fortunei* in the gardens, whilst 9.1% of the secondary residences north of the Alps have *T. fortunei* in cultivation.

### Information Level

A share of 39.0% of the public affirmed to know the meaning of the term “invasive alien species” in their respective languages (i.e., “neofita invasiva” in Italian, “invasiver Neophyt” in German, and “néophyte envahissante” in French). This share was significantly lower (7.6%) for those with elementary- and middle school-level education compared to those with a higher level of education (Kruskal–Wallis test, *p* < 0.05). Moreover, individuals with a tertiary-level (i.e., academic) degree knew the term significantly more compared to those with education levels up to the high school level (Kruskal–Wallis test, *p* < 0.05). The major sources of information were television (46.5%), newspapers (36.5%), and the internet (33.9%). On the other hand, 19.3% of the public did not know the meaning of the term, and 41.7% had never heard of it before. Concerning age, those in the over 65 age group showed a significantly greater knowledge of the term “invasive alien species” in their respective languages with respect to the youngest age group (i.e., 18–29 years old; Kruskal–Wallis test, *p* < 0.05). 82.8% of the public believed themselves to be insufficiently informed about the topic of invasive alien species in Switzerland.

In general, participants did not demonstrate deep knowledge of the focal species. Even though more than 90% knew that it is not native to Europe, only 34.4% were aware of its Asiatic origin. This percentage only increased to 37.2% among house owners (primary and secondary residences) that have *T. fortunei* in their garden. The Asiatic origin of *T. fortunei* was reported only by 30.7% of the under 49 age group and 38.2% of the 50–65 group, respectively.

The majority (52.0%) of Italian-speaking informants believed that the presence of the species in private and public places has an effect on the surrounding natural environment. This was the case only for 40.0% of French-speaking and 29.7% of the German-speaking informants. Those with low education levels (up to middle school certificate) rated the ecological impact of *T. fortunei* significantly more positively than those with higher levels of education (Kruskal–Wallis test, *p* < 0.05). Among owners of secondary residences, those possessing one or more individuals of *T. fortunei* (12.1%) had greater awareness of the consequences its presence in private and public gardens may have on the natural environment compared to owners of primary residences. However, when compared to those without any *T. fortunei* individuals, owners of primary residences with individuals were significantly more aware of the negative impact of *T. fortunei* on the surrounding natural environment (Kruskal–Wallis test, *p* < 0.05). Surprisingly, and in an apparent contradiction, owners of secondary residences with *T. fortunei* in their gardens assessed the impact of the species on the natural environments mainly as positive or very positive.

House owners who would like to have (but do not have) *T. fortunei* on their properties (29.6% and 24.6% for primary and secondary residences, respectively) displayed a less coherent knowledge of possible negative effects of cultivating *T. fortunei*. More than 40.0% of these informants had never heard of the term “invasive alien species” in their respective languages. Finally, almost half of the primary residence owners and more than half of the secondary residence owners who expressed the desire to have *T. fortunei* in their gardens, also rated the impact of the species on the surrounding natural environment as positive or very positive.

### Perception

**Fig. 3:**
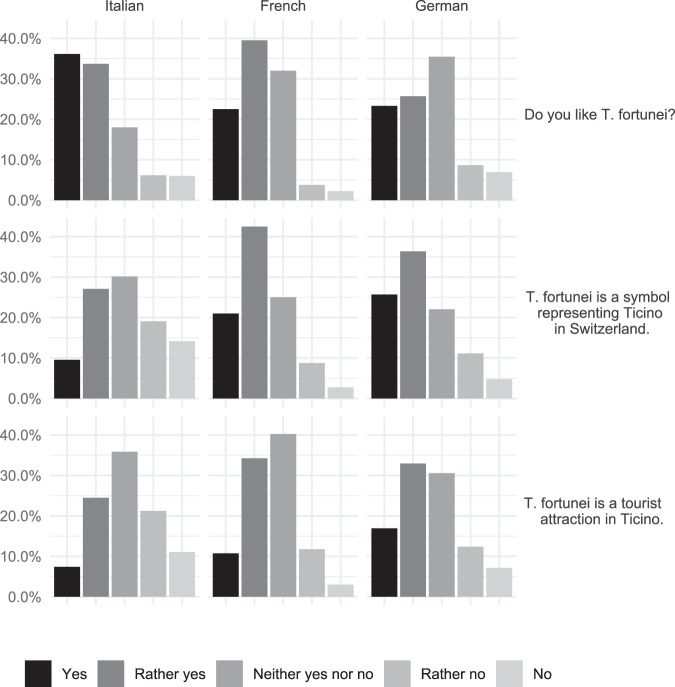
Perception of *T. fortunei* in the three main linguistic regions

Most participants (58.9%) appreciated *T. fortunei*, which is perceived as a symbol of the whole Insubric region (61.4%), and of the Canton of Ticino specifically (53.9%), where it was also considered a tourist attraction (42.9%). This perception varied significantly as a function of the native language, with the Italian and French speakers appreciating the species significantly more (69.9 and 62.0%, respectively) than German speakers (49.0%; Kruskal–Wallis test, *p* < 0.05, Fig. 3). In addition, most German and French native speakers perceived *T. fortunei* as a symbol (62.1 and 63.5%, respectively) and as a tourist attraction for Ticino (49.9 and 45.1%, respectively), whereas Italian native speakers were almost equally divided into three groups for both the role of the species as a symbol (yes: 36.6%, indifferent: 30.2%, no: 33.3%) and as a tourist attraction (yes: 31.9%, indifferent: 35.8%, no: 32.3%) for the Canton of Ticino.

The perception of *T. fortunei* was also significantly influenced by age. Under 50 years of age, participants expressed greater appreciation (67.5%) compared to over 50 (49.4%), with a further decrease in appreciation as the age class increases. In addition, older age classes conferred more importance to *T. fortunei* as a symbol of Ticino (under 64: 49.9%, over 65: 64.1%) and as a tourist attraction (under 64: 39.5%, over 65: 52.0%).

With respect to the place of residence, those living in urban areas liked *T. fortunei* significantly more (city centers: 63.5%, suburbs: 62.7%) than inhabitants of rural or mountain areas (50.1%; Kruskal–Wallis test, *p* < 0.05). Among owners of primary residences with one or more individuals of *T. fortunei*, there was a significantly higher proportion that viewed the species as a symbol of the Insubric lakes region (69.4%; Kruskal–Wallis test, *p* < 0.05). Other differences, albeit not statically significant, were found comparing palm owners and those who are not, with respect to the role of *T. fortunei* as a symbol of Ticino (59.4 and 47.8%, respectively) and as a tourist attraction (53.4 and 42.4%, respectively). In contrast, no related patterns were found in responses concerning the perception of *T. fortunei* based on education level.

Concerning the first word associated with *T. fortunei*, we have grouped the mentioned terms from the three national languages into 46 umbrella English terms for the purpose of this article. Table 2 summarizes the ten most frequent umbrella terms for each linguistic group. The most mentioned subject categories have been assigned to the terms “drug” (22.4%), “holidays” (9.8%), and “warm” (8.5%). The terms “holidays”, “warm”, “exotic”, “beautiful”, and “tropics” appeared in the top ten ranking regardless of the language. Subject categories belonging to “drug”, “south”, and “palm” were found to be very common only among the non-Italian speakers. With a share of 6.7% the umbrella term “invasive” was most cited among the Italian native speakers compared to less than 1.0% for French and German speakers.

**Table 2:** Ranks and frequencies of the often-recurring word categories based on linguistic regions

Category	Overall		Native language					
			Italian		French		German	
	Count	%	Rank	%	Rank	%	Rank	%
Drug	448	22.37			**2**	14.99	**1**	41.17
**Holidays**	196	9.79	**2**	10.45	**1**	16.71	**3**	6.28
**Warm**	170	8.49	**1**	13.13	**3**	12.53	6	3.36
**Exotic**	151	7.54	4	8.81	4	11.30	5	4.98
**Beautiful**	115	5.74	8	4.03	5	6.39	**2**	6.72
Sea	88	4.39	**3**	10.44	8	2.21		
**Tropics**	68	3.39	5	6.87	7	2.46	10	1.30
South	58	2.90			9	1.97	4	5.31
Invasive	53	2.65	6	6.72				
Palm	45	2.25			6	3.19	7	2.82
Luck	36	1.80	7	5.37				
Height	31	1.55	9	3.58				
Ticino	29	1.45					8	2.60
Fruit	28	1.40	10	2.54				
Unknown	25	1.25					9	1.84
Fiber	20	1.00			10	1.96		

The categories mentioned by all the three native languages and the first three categories mentioned by each language group are shown in bold

### Preference

**Fig. 4:**
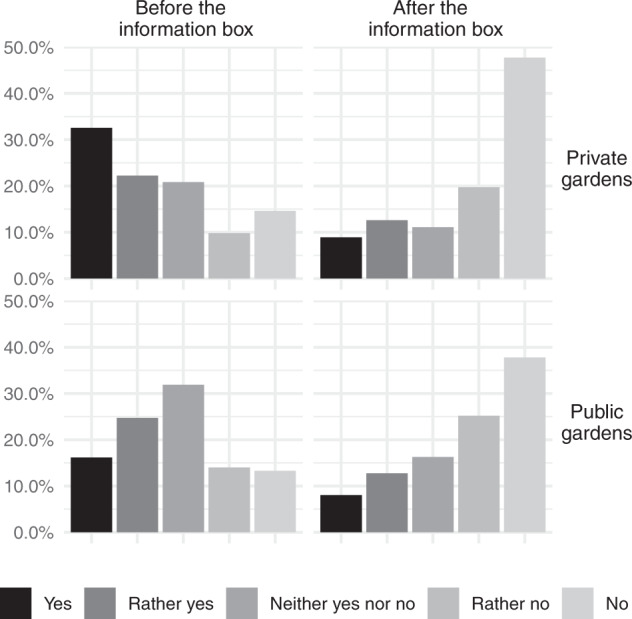
People’s preference regarding the presence of *T. fortunei* in private and public gardens before and after providing information on the invasive behavior of *T. fortunei* and the potential ecological and socio-economic consequences. Positive answers mean a positive preference concerning the presence of *T. fortunei* in the two places

Before receiving targeted information on the invasive potential of the species, participants generally looked favorably on the presence of *T. fortunei* in urban areas or other cultural settings, but not in natural environments. Indeed, the majority of the participants reported to appreciate the species in private (54.9%) and public gardens (66.2%) (Fig. 4), and at urbanized lakesides (60.2%), but not along roads (44.8%). On the other hand, *T. fortunei* was not appreciated along riversides (49.5%), at forest edges (63.2%), or in forests (67.4%). In addition, Latin-based language informants (i.e., Italian or French speakers) liked *T. fortunei* in courtyards significantly less than German speakers (Kruskal–Wallis test, *p* < 0.05). In a similar contrast, only 49.6% of owners of primary residences with *T. fortunei* in their gardens appreciated *T. fortunei* in courtyards, while 64.3% of owners with the species in secondary residences do. Finally, over 65 age classes were less likely to appreciate *T. fortunei* in private and public gardens (Kruskal–Wallis test, *p* < 0.05). No additional significant differences in the preferences of the species were revealed in our results.

The desire to see *T. fortunei* in private and public gardens markedly changed among participants after receiving information on the invasive character of the species (Fig. 4), regardless of group (i.e., based on native language, zone of residence, age class, education level; data not shown). Increases in the percentage of those that disliked seeing *T. fortunei* in both private and public gardens ranged from 25% to more than 60%. It is noteworthy that, slightly more owners of primary residences with one or more individual of *T. fortunei* liked to see more individuals of *T. fortunei* in private gardens even after having read the additional information on the invasive potential of *T. fortunei* (43.2 compared to 41.3%, with the remaining proportion being indifferent).

### Willingness to Reduce *T. fortunei*

It was generally agreed that federal and cantonal authorities should invest more in management strategies to control invasive alien species (68.7%) and prevent the spread of *T. fortunei* outside gardens and public parks (77.8%). Inhabitants of rural and mountain areas were significantly more ready to remove the inflorescences of female individuals of *T. fortunei* themselves or by hiring someone (78.0%) compared to those living in city centers (64.7%) and in suburbs (65.9%) (Kruskal–Wallis test, *p* < 0.05). Interestingly, 11.8% of primary residence owners with *T. fortunei* in their gardens would not be ready to remove female inflorescences even after having read the information box explaining the problems that the species may cause in natural environments. This was the case for 3.6% of secondary residence owners cultivating *T. fortunei*. No significant divergences from the whole occurred among age classes or education levels.

**Fig. 5:**
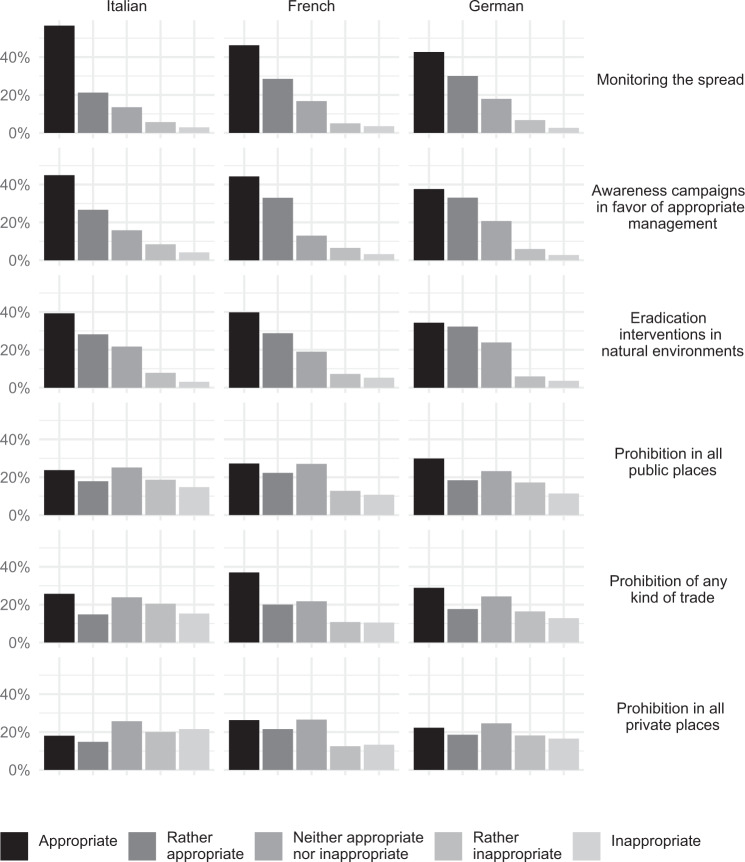
Acceptance of the six proposed management options based on the linguistic regions

Overall, the lightest management options (monitoring of spread, awareness campaigns, eradication interventions in natural environments) were assessed as appropriate by a clear majority of the public (Fig. 5). For the more stringent proposed management options, the consensus among informants decreased but remained relatively balanced and without strong opposition, whereas roughly one quarter was indifferent regarding the management suggestions. However, we uncovered evident inter-language divergences regarding the three strictest management options. Italian native speakers found the strictest measures significantly more inappropriate compared to the other linguistic groups (Kruskal–Wallis test, *p* < 0.05). Italian speakers were also the only group of informants that assessed the prohibition in private places as inappropriate (indifferent answers were ignored). Beside this exception, most participants of the three main linguistic groups accepted the strictest management options.

Older age groups tended to evaluate all management options, including a ban on *T. fortunei*, more positively than younger age groups. For instance, over 50 age groups viewed prohibition in all public places and of any kind of trade as an appropriate measure more than under 50 age groups (Kruskal–Wallis test, *p* < 0.05, with the exception in the latter case of the non-significant difference among the classes 40–49 and 50–65 years of age). Similarly, 52.8% of those 65 and up stood out significantly from other age classes in the support of prohibition of the species in all private places (Kruskal–Wallis test, *p* < 0.05). More than 50.0% of both primary and secondary residence owners with one or more individuals of *T. fortunei* tended to agree on the three less stringent management options. Fewer owners of both primary (36.9%) and secondary (42.9%) residences were in favor of prohibiting *T. fortunei* in all public places. Among owners with *T. fortunei* in their gardens, those of secondary residences displayed higher agreement with prohibition of any kind of trade of the species than those of primary residences (46.4 vs. 36.3% for agreement, 21.4 vs. 18.1% indifferent, 32.2 vs. 45.6% disagreement, respectively). The prohibition of *T. fortunei* in all private places was rejected by most owners of the focal species: indeed, only 30.7% of the primary residence owners and 35.7% of the secondary residence owners agreed with this option (in this latter case, compared to the slightly higher 35.8% who consider the option as inappropriate). Furthermore, the owners of secondary residences in Ticino found the prohibition of *T. fortunei* in all private places less appropriate compared to those with a secondary residence in other part of Switzerland (Kruskal–Wallis test, *p* < 0.05).

### Acceptance of Substitute Species

The IAT revealed that each morphological group (fan palms, feather palms, yucca-like plants, and banana plants) comprises at least one species that was accepted as an alternative ornamental species for *T. fortunei* by the majority of the participants (Supplementary Materials S3). Individuals without dead leaves in the picture were accepted the most (*Washingtonia robusta* [62.4%], *Cordyline australis* [58.4%], *Ensete ventricosum* [57.2%] and *Jubaea chilensis* [53.2%]), individuals with few dead leaves were accepted to a lesser extent (*Phoenix canariensis* [52.4%], *Yucca gloriosa* [46.5%] and *Brahea armata* [35.5%]) and the only individual with many dead leaves (*W. robusta*) was accepted by the fewest participants (18.8%). Overall, not the morphological group but the amount of dead leaves attached to the plant shown in the picture was the pivotal factor explaining the acceptance of a species as a substitute for *T. fortunei*. Linguistic groups were all in agreement and in line with the general results reported above. Compared to the rest of the informants, owners of *T. fortunei* (primary and secondary residences) expressed greater appreciation for every proposed species, except for some individuals with many dead leaves (*B. armata* [42.7%] and *W. robusta* [30.0%]).

### Factors Influencing the Acceptance of Management Options

**Fig. 6:**
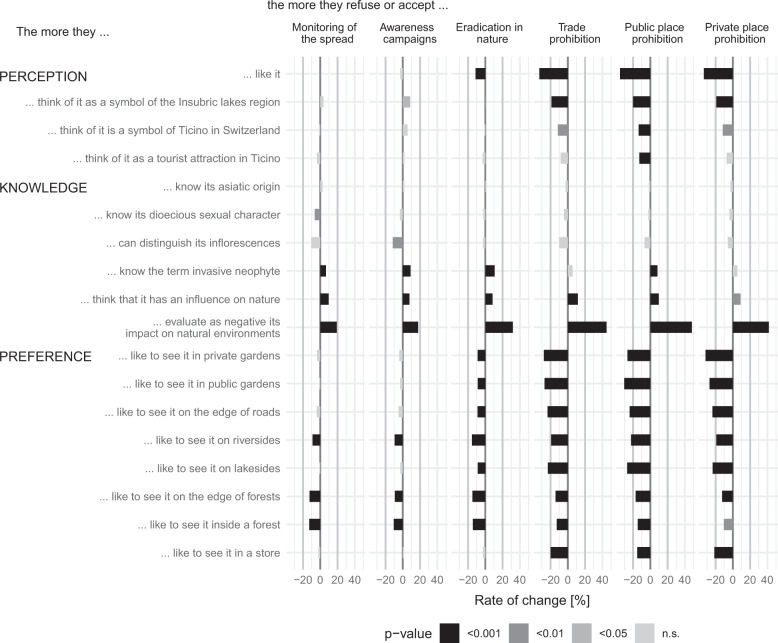
Relationships between perceptions, knowledge, and preferences (*y*-axis) and the acceptance of the proposed management options (*x*-axis). For every unit of change in perceptions, knowledge, and preferences, there is a specific rate of change (%) in the evaluation of the management option. For example, the more the people like the windmill palm (i.e., first line), the less they appreciate the strictest management options (i.e., prohibitions; *p* < 0.001). All *p* values are Bonferroni corrected

The level of appreciation of *T. fortunei* displayed a highly significant relationship with the willingness to accept the most stringent (i.e., prohibition of the species) management options (*p* < 0.001) (line 1 in Fig. 6). In this case, for every unit of change toward a greater appreciation of *T. fortunei*, the approval of the stricter management options decreased by more than 30.0%. In other words, the more the participants liked *T. fortunei*, the less they were willing to accept strict measures. In contrast, the degree of appreciation of *T. fortunei* did not significantly influence the approval of the lighter management alternatives (i.e., monitoring the spread, awareness campaigns, and eradication in natural environments, see lines 1–4 in Fig. 6).

Participants’ knowledge of the term “invasive alien species”, how the presence of *T. fortunei* in private and public gardens influenced surrounding natural environments, and especially the awareness of the negative impact of the species, were found to be determining factors in the approval of each management option (*p* < 0.001, see lines 8–10 in Fig. 6). In these latter cases, the greater the knowledge was, the higher was the approval rate of the measures. In contrast, knowledge of the origin of *T. fortunei*, of its dioecious sexual character (having female and male reproductive organs on separate individuals), and the ability to distinguish male and female inflorescences did not produce any clear change in the appreciation of the management alternatives (lines 5–7 in Fig. 6).

Highly significant appreciation and high rates of change in the approval of severe management options were found especially among those unwilling to see *T. fortunei* in private and public gardens (*p* < 0.001, see lines 11–12 in Fig. 6). In other words, the more the informants liked to see *T. fortunei* in private and public places, the less they were willing to accept the measures. Nevertheless, the results also showed that generally those preferring to see *T. fortunei* in every context proposed were less likely to accept the strictest management options or the eradication of the species in natural environments. This was also the case with the lower appreciation of lighter measures among those who liked to see *T. fortunei* on the edge and inside forests or along riversides (lines 14, 16, and 17 in Fig. 6).

Supplementary Materials S6 include additional plots that have not been shown here. In particular, the level of information, perceptions, the importance of the information box, preferences, and the management of *T. fortunei* based on social groups are presented.

## Discussion

Issues related to invasive alien species are not only concerned with environmental and ecological aspects, but also imply a significant social and cultural component (Kapitza et al. 2019). Public acceptance is not always guaranteed for invasive species management, especially not in the case of charismatic and iconic species, where the implementation of effective management measures can face low public acceptance (Jarić et al. 2020). In this paper, we conducted a nationwide online survey to investigate socio-cultural aspects and general public’s attitude toward possible control measures of the iconic Chinese windmill palm (*T. fortunei*) in Switzerland, which may serve as a textbook example to study social and cultural aspects of a charismatic and iconic invasive species.

### Information Level

This case study of *T. fortunei* in Switzerland confirms the difficulty for the general public to become sufficiently informed about the invasive alien species issue broadly, and about individual invasive species in particular (Shackleton and Shackleton 2016; Junge et al. 2019). Despite supporting a generic increase in public investment for the prevention and control of its spread, our results show that the public has a low level of knowledge concerning the specific case of *T. fortunei* and its invasive potential in particular. This is partially true also of southern Switzerland, where the invasive behavior of the species is already visible. The low state of knowledge about the potential negative effects might be caused by the iconic nature of the focal species, which distracts from and/or outweighs possible negative consequences. The fact that *T. fortunei* was officially declared invasive very recently in 2014 (Info Flora 2014) and the still pending scientific proof of negative ecological or socio-economic effects (Vogelaar and Hale 2013), might also explain the lack of awareness about undesirable effects.

The context dependence and the psychological, cognitive, and social dimensions of people’s judgment on non-native invasive species and their impact on local ecosystems (Kueffer and Kull 2017) are well reflected in our findings. Examples include the differences in knowledge about the potential threats of *T. fortunei* found at the language level (Italian speakers being more aware) and with respect to *T. fortunei* ownership patterns. Also, the quantity and quality of knowledge increases as a function of the level of education and age (Bowler and Donovan 1994; Potgieter et al. 2019). Owners of *T. fortunei* showed greater knowledge compared to non-owners and among these, owners of secondary residences were found to be the most informed. This may be the consequence of the greater attachment of seasonal residents to local environmental quality (Stedman 2006).

### Perceptions and Preferences

Our findings confirmed *T. fortunei* as a charismatic species. The majority of participants have already seen it in cultivation—a sign for its high recognition value (Jarić et al. 2020). Also, the publics’ perception was largely very positive: among the most mentioned expressions were terms such as “holidays”, “warm”, “exotic”, and “beautiful”. The iconic status of *T. fortunei* was further evidenced by the fact that the majority of the participants agreed on its symbolic character for the Insubric region and the Canton of Ticino. Constituting a symbolic character within a certain region is a key trait of a species designated as iconic (Albert et al. 2018). However, divergences among different groups in the perception of *T. fortunei* exist and indicate different degrees of exposure to the problem, possibly reflecting how differently *T. fortunei* impacts the landscape and how aesthetic values are related to the local inhabitant’s identity (Berenguer et al. 2006; Hunziker et al. 2008; Dickie et al. 2014). Whilst French and German speakers mainly indicated words suggesting positive feelings toward the species, Italian speakers repeatedly mentioned the term “invasive”, which indicates a negative association with the species. Unsurprisingly, French and German speakers also frequently mentioned drug-related words. This association can be explained by the common names of *T. fortunei* in their native languages, which are “palmier chanvre” and “Hanfpalme”, “chanvre” and “Hanf” corresponding to the French and German words for hemp, respectively. The Italian common name for *T. fortunei* (i.e., palma di Fortune), on the contrary, does not present this association (and has nothing to do with the Italian word “fortuna” = “luck/happiness” neither). In line with Dullinger et al. (2017), people living in urban spaces have been found to appreciate *T. fortunei* significantly more compared to inhabitants of rural or mountain areas. Existing discrepancies in the perception between urban and rural inhabitants may also relate to the context-dependent differences in the conflicts that the invading behavior of the species may cause (e.g., Novoa et al. 2017).

Our results also demonstrate the importance of detailed and targeted information for raising awareness and modulating the perceptions of potentially problematic phenomenon (Bowler and Donovan 1994; García-Llorente et al. 2008; Junge et al. 2019; Ryan 2012). Before information on potential negative effects of *T. fortunei* was provided, the public—with the exception of the over 50-year olds—expressed their appreciation for the presence of *T. fortunei* in urban and other cultural settings. After having received the information on the potential negative effects, the appreciation of the species decreased significantly across all groups of participants. However, a slight majority of owners of *T. fortunei* (primary residence) still liked seeing individuals of the species in private and public places even after having received the information on potential negative effects. As already reported by Lindemann-Matthies (2016), the appreciation of *T. fortunei* may overcome the awareness of the damage it could cause to the natural environment, what is typical for charismatic and iconic species (Jarić et al. 2020). The desirability of a species may thus highly influence the general public’s overall perception of and attitude toward the species (Cordeiro et al. 2020; Selge et al. 2011).

### Willingness to Reduce *T. fortunei*

As Lindemann-Matthies (2016) showed for ornamental invasive alien plants in Switzerland in general, people are only conditionally willing to reduce the propagule pressure of *T. fortunei*, and largely approve only light management options (i.e., monitoring the spread, awareness campaigns, and eradication in nature). In the present study, owners of individuals of *T. fortunei* only agreed with the light management options but have a stronger inclination to rate its prohibition in private places and, to a lesser extent, its ban in public places also, as inappropriate. These outcomes show that most *T. fortunei* owners appreciate the species and that they value its aesthetics greater than its potential negative effects on the natural environment. However, older age classes tended to agree with most of the proposed management options (i.e., light and strict), which is the result of their lower appreciation of the species despite their perception of it as a symbol and tourist attraction for southern Switzerland. This discrepancy between generations might be explained by the shifting baseline syndrome, which refers to a shift over time of how the state of a certain ecosystem is expected to be (Pauli 1995; Soga and Gaston 2018). Consequently, older age classes might have witnessed the transition from sites uninvaded by *T. fortunei* to *T. fortunei*-dominated sites, whereas for younger generations palm-infested sites are perceived as a natural state of the ecosystem. Finally, although the willingness to accept stringent management measures was found to be inversely related to the level of appreciation for *T. fortunei*, we have also demonstrated how awareness of potential negative ecological and socio-economic impacts of *T. fortunei* on natural environments resulted in increased acceptance of proposed management options. Our findings correspond with prior studies showing that control measures are generally supported by the public in case they are informed about potential ecological risks (Verbrugge et al. 2013). As public support can be seen as a prerequisite in case of invasive, charismatic species control (Höbart et al. 2020), our findings mark an important step forward in understanding the mechanisms related to willingness to take action regarding charismatic, invasive species.

### Acceptance of Substitute Species

A promising solution for increasing the public acceptance of measures to ban or to limit the spread of an iconic invasive species is the promotion of alternative non-invasive species of similar charisma and aesthetic value, which may be used to substitute those posing an invasion risk (Drew et al. 2010). As alternative species for *T. fortunei*, other climatically suited but non-invasive palm species (e.g., *Brahea armata*, *Jubaea chilensis*, *Phoenix canaeriensis*, *Washingtonia robusta*) and palmlike species from the Aspargaceae family (e.g., *Cordyline australis*) and banana plants (e.g., *Musa basjoo*) obtained the support of the majority of the participants and are thus worth considering as substitutes, provided that the dead leaves, which often persist on many palm species for several years (also on healthy individuals), will be removed to obtain a tidy look. Choosing species on which the dead leaves do fall away from themselves (e.g., *Jubaea chilensis*) should be preferably cultivated to save costs and efforts for pruning.

In addition, although most suggested alternative species for *T. fortunei* are currently not reported to naturalize in the Insubric region, *Phoenix canariensis* and *Washingtonia robusta* have been reported to frequently naturalize and showing an invasive behavior in the Mediterranean region (Fehr et al. 2020). The recommendation of alternative species should thus carefully consider also possible future climate conditions for the region of interest. On the other hand, the promotion of alternative and non-invasive ornamental species may also motivate nursery and landscape industries to remove invasive plant species from their stocks, and to participate in consumer education about non-invasive alternative species (Gagliardi and Brand 2007). Conversely, consumers aware of the invasion potential of a species are likely to demand substitute species, potentially increasing the sales of the horticultural industry which is tempted to extend the product range with new species and cultivars (Drew et al. 2010).

### Recommendations for Refining Management Strategies

Our findings reinforce the suitability of using a holistic approach in the management of invasive alien species, which put experts’ opinions and public awareness and agreement on the same level (Crowley et al. 2017; Luz 2000). In this context, extensive research is essential to consider different perceptions that may co-exist among local communities, stakeholders and cultural groups (Crowley et al. 2017, García-Llorente et al. 2008). Information and co-operation then become key factors toward shaping an effective and durable public acceptance of landscape conservation measures (Bremner and Park 2007; Cordeiro et al. 2020; Novoa et al. 2017; Ryan 2012; Schenk et al. 2007). Increasing the public awareness about invasive alien species could be achieved by involving the public in citizen science projects e.g., monitoring invasive alien species (Crall et al. 2012, Paganelli et al. 2021)

We showed that public awareness and perceptions of an iconic and invasive species can vary greatly according to the cultural background (language), education age, and ownership of the focal species. We also found that providing information to the public on the effects of invasive species can have a strong effect on their attitudes toward invasive species and consequently on their willingness to accept potential management actions. Targeted information programs that recognize shared values of stakeholders are the best prerequisite for establishing efficient and long-lasting dialogs (Dickie et al. 2014). When planning local or regional eradication or control actions, public information should adapt to the people concerned. Similarly, the information provided should differentiate between control measures for private and for public areas, because of the divergence in the attitudes of the population in this respect. Special attention should be paid to informing professional operators, such as gardeners and landscape architects. Proposed management options should be then calibrated according to the specific residents’ perceptions. For instance, where the high propagule pressure already caused the uncontrolled spread of *T. fortunei* in gardens, people may be willing to accept stricter management options and even the eradication of the species from their own gardens. Suggesting non-invasive substitute species might increase the public acceptance and the interest of the gardening and landscape industry in removing individuals of *T. fortunei*.

## Conclusion

The present survey on the case of the iconic but invasive Chinese windmill palm (*T. fortunei*) in Switzerland revealed significant differences in the knowledge and perceptions of the invasion potential of non-native species within the population that eventually lead to several levels of acceptance of possible management options. Accompanying any management approach for invasive non-native plants with a targeted information campaign thus represents a prerequisite for the success of the proposed measures, especially in the case of very strict management options such as the potential ban of an iconic species such as *T. fortunei* in southern Switzerland.

## Supplementary information

Supplementary Materials S1

Supplementary Materials S2

Supplementary Materials S3

Supplementary Materials S4

Supplementary Materials S5

Supplementary Materials S6
