# Supplementary material for: Iconic but Invasive: The Public Perception of the Chinese Windmill Palm (Trachycarpus fortunei) in Switzerland
Source: Environ Manage. 2022 Apr 26;70(4):618–32. doi: 10.1007/s00267-022-01646-3 (PMC9439986; doi:10.1007/s00267-022-01646-3)
Supplement: Supplementary file 1 — Supplementary Materials S1 [file 267_2022_1646_MOESM1_ESM.pdf]

Supplementary Materials S1

Palm trees as iconic elements of the study region

Examples of the use of palms as iconic regional element for touristic advertisement by the mean of poster illustrations. Each poster is labeled with the author and year of publication.

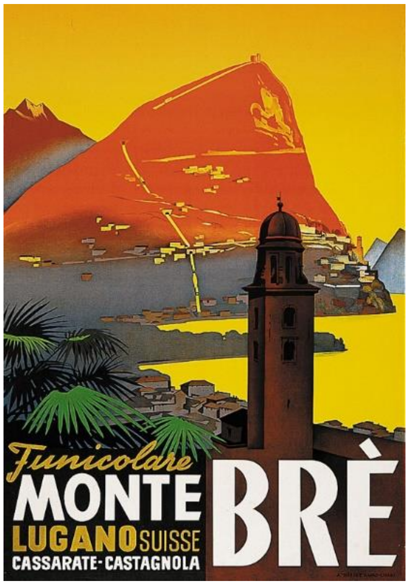

Ernst Otto, 1930
Trachycarpus fortunei

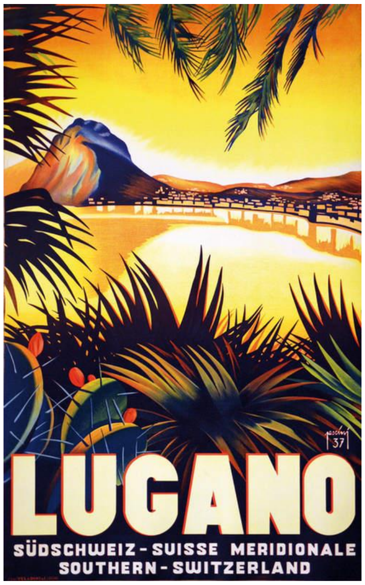

Pescini Mario, 1937
generic palms

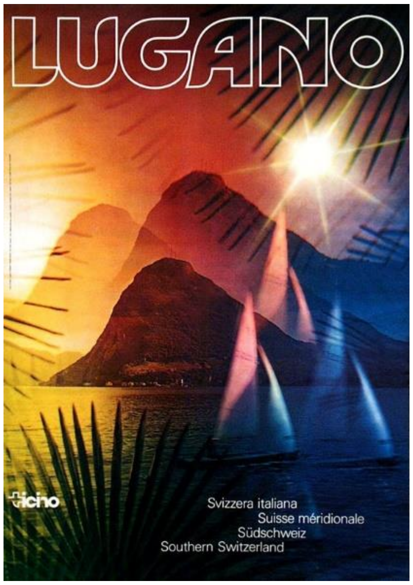

Galli Orio, 1978
Trachycarpus fortunei

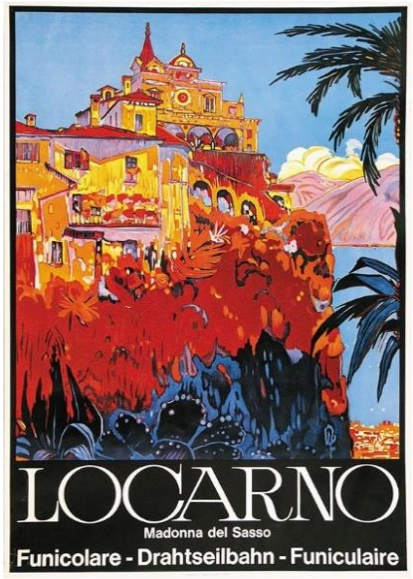

Buzzi Daniele, 1930
generic palms

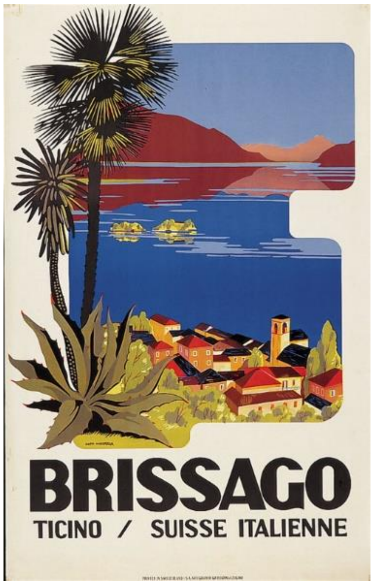

Anderegg Sepp, 1945
Trachycarpus fortunei

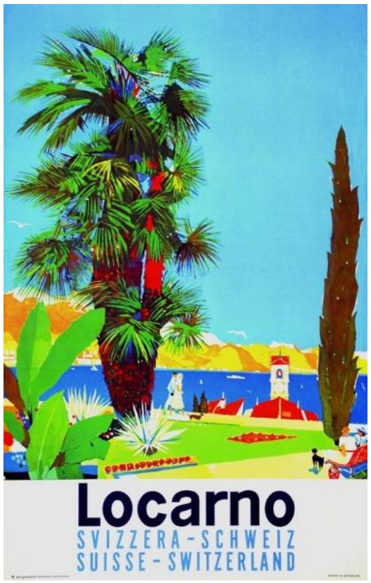

Buzzi Daniele, 1968
generic palms

Screenshot of the current official regional tourism webpage proposing winter walks in the study area in a palm landscape with a snowy background.

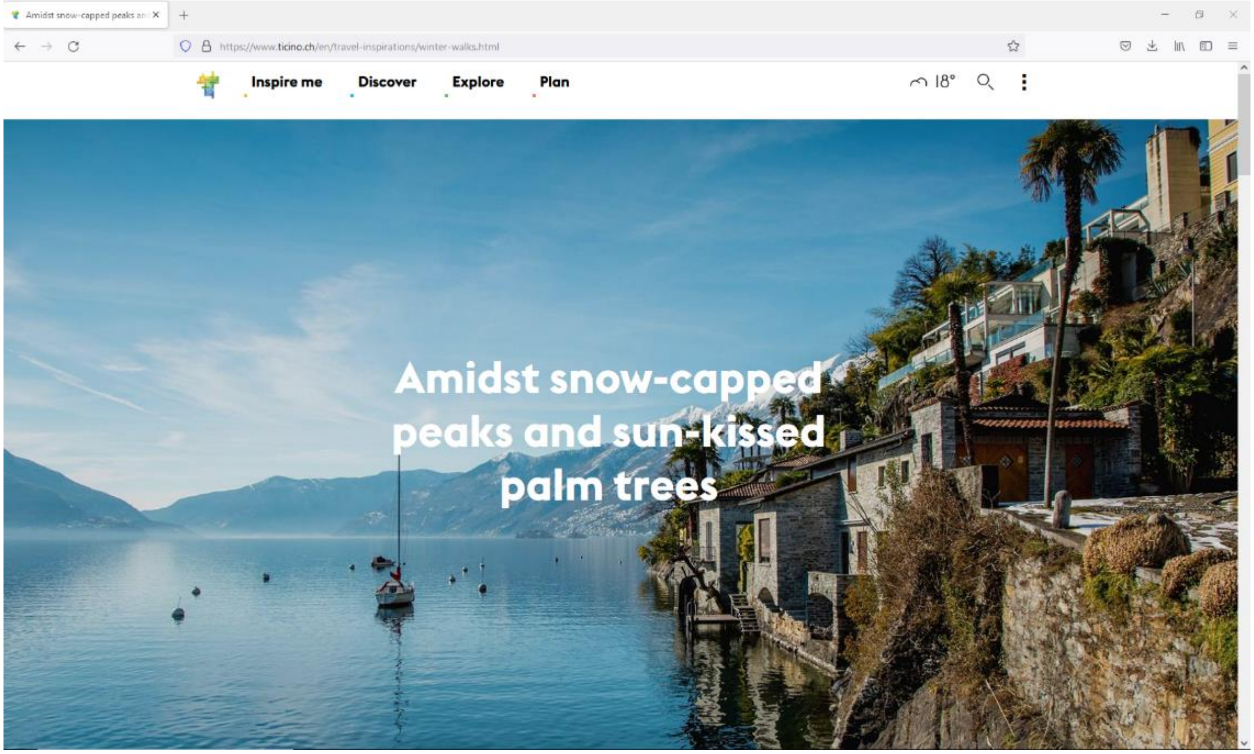

Sources (accessed 21 March 2022):

https://swisspostermuseum.com/sammlung/cat/Kanton-Tessin/1?show=50

https://swisspostermuseum.com/sammlung/cat/Kanton-Tessin/2/?show=50

https://www.ticino.ch/en/travel-inspirations/winter-walks.html
