# Supplementary material for: Iconic but Invasive: The Public Perception of the Chinese Windmill Palm (Trachycarpus fortunei) in Switzerland
Source: Environ Manage. 2022 Apr 26;70(4):618–32. doi: 10.1007/s00267-022-01646-3 (PMC9439986; doi:10.1007/s00267-022-01646-3)
Supplement: Supplementary file 2 — Supplementary Materials S2 [file 267_2022_1646_MOESM2_ESM.pdf]

Supplementary Materials S2

Survey - English translation and original questionnaires in the three Swiss national languages

Page 2	English version
Page 16	Italian version
Page 30	German version
Page 44	French version

English version

The purpose of this questionnaire is to investigate the perception and the knowledge of the windmill palm (*Trachycarpus fortunei*) among the Swiss population. The image here below shows some examples of the windmill palm.

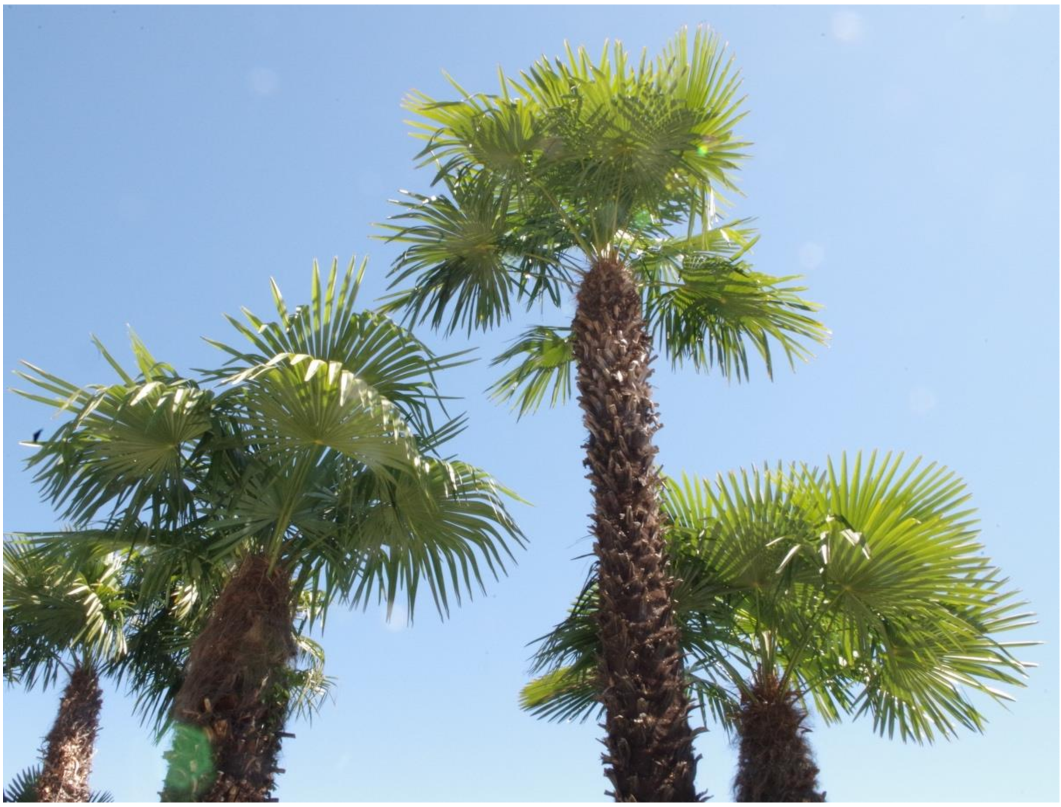

The completion of the questionnaire is voluntary and anonymous, personal information is only used for statistical purposes. It takes 15 minutes to complete.
The survey is part of a Master's thesis conducted at the Swiss Federal Institute of Technology Zurich (ETHZ) in collaboration with the Swiss Federal Institute for Forest, Snow and Landscape Research (WSL, Cadenazzo site).

1. Canton of origin
   Aargau (AG), Appenzell Innerrhoden (AI), Appenzell Ausserrhoden (AR), Bern (BE), Basel-Landschaft (BL), Basel-Stadt (BS), Fribourg (FR), Geneva (GE), Glarus (GL), Grisons (GR), Jura (JU), Lucerne (LU), Neuchâtel (NE), Nidwalden (NW), Obwalden (OW), St. Gallen (SG), Schaffhausen (SH), Solothurn (SO), Schwyz (SZ), Thurgau (TG), Ticino (TI), Uri (UR), Vaud (VD), Valais (VS), Zug (ZG), Zurich (ZH)

2. Canton of permanent residence
   Aargau (AG), Appenzell Innerrhoden (AI), Appenzell Ausserrhoden (AR), Bern (BE), Basel-Landschaft (BL), Basel-Stadt (BS), Fribourg (FR), Geneva (GE), Glarus (GL), Grisons (GR), Jura (JU), Lucerne (LU), Neuchâtel (NE), Nidwalden (NW), Obwalden (OW), St. Gallen (SG), Schaffhausen (SH), Solothurn (SO), Schwyz (SZ), Thurgau (TG), Ticino (TI), Uri (UR), Vaud (VD), Valais (VS), Zug (ZG), Zurich (ZH)

3. Where do you live?
   1/ Urban space: city center
   2/ Urban space: suburbs
   3/ Rural or mountain areas

4. Native language
   1/ Italian
   2/ French
   3/ German
   4/ Romansh
   5/ Other …………

5. Year of birth

2002	2001	2000	1999	1998	1997	1996	1995	1994	1993
1992	1991	1990	1989	1988	1987	1986	1985	1984	1983
1982	1981	1980	1979	1978	1977	1976	1975	1974	1973
1972	1971	1970	1969	1968	1967	1966	1965	1964	1963
1962	1961	1960	1959	1958	1957	1956	1955	1954	1953
1952	1951	1950	1949	1948	1947	1946	1945	1944	1943
1942	1941	1940	1939	1938	1937	1936	1935	1934	1933
1932	1931	1930	1929	1928	1927	1926	1925	1924	1923
1922	1921	1920							

6. Gender
   1/ Female
   2/ Male
   3/ Other …………

7. Where do you think the windmill palm comes from?
   1/ Africa
   2/ Asia
   3/ Europe

4/ North America
5/ Oceania
6/ South America

8. Have you already seen the windmill palm in Switzerland?
   1/ Yes
   2/ No **(→ to question 11)**

9. In which Swiss Cantons have you already seen the windmill palm?
   Aargau (AG), Appenzell Innerrhoden (AI), Appenzell Ausserrhoden (AR), Bern (BE), Basel-Landschaft (BL), Basel-Stadt (BS), Fribourg (FR), Geneva (GE), Glarus (GL), Grisons (GR), Jura (JU), Lucerne (LU), Neuchâtel (NE), Nidwalden (NW), Obwalden (OW), St. Gallen (SG), Schaffhausen (SH), Solothurn (SO), Schwyz (SZ), Thurgau (TG), Ticino (TI), Uri (UR), Vaud (VD), Valais (VS), Zug (ZG), Zurich (ZH)

10. Please indicate in which environment/place you have already seen the windmill palm.
   1/ Private garden
   2/ Public garden (parc)
   3/ Lakeside
   4/ Riverside
   5/ Edge of a forest
   6/ Inside a forest
   7/ Edge of a road
   8/ Store
   9/ Other

11. Would you like to see the windmill palm in the environments/places listed here below?

	Yes	Rather yes	Neither yes nor no	Rather no	No
	1	2	3	4	5
1/ Private garden					
2/ Public garden (parc)					
3/ Lakeside					
4/ Riverside					
5/ Edge of a forest					
6/ Inside a forest					
7/ Edge of a road					
8/ Store					

12. Are there one or more windmill palms on properties adjacent to your primary residence?
   1/ Yes
   2/ No **(→ to question 14)**
   3/ I do not know **(→ to question 14)**

13. How many windmill palms are there on the properties adjacent to your primary residence?
    1/ A few (1-3)
    2/ Some (4-10)
    3/ A lot (>10)

14. If you have a private garden, or if your building has one, who primarily maintains it?
    1/ I do not have a private garden / The building does not have a private garden **(→ to question 19)**
    2/ Me, personally
    3/ A member of my family
    4/ A non-professional person
    5/ A professional

15. Are there any windmill palms in your garden or in the garden of your building?
    1/ Yes
    2/ No, but I would like to have some **(→ to question 19)**
    3/ No, and I do not want any **(→ to question 19)**
    4/ I do not know **(→ to question 19)**

16. How many windmill palms are there in your garden or in the garden of your building?
    1/ A few (1-3)
    2/ Some (4-10)
    3/ A lot (>10)

17. Was the decision to plant these windmill palms yours or of a member of your family?
    1/ Yes
    2/ No **(→ to question 19)**

18. Where did you get them? …………

19. Do you or your family have a secondary residence in Switzerland?
    1/ Yes
    2/ No **(→ to question 24)**

20. In which Canton?
    Aargau (AG), Appenzell Innerrhoden (AI), Appenzell Ausserrhoden (AR), Bern (BE), Basel-Landschaft (BL), Basel-Stadt (BS), Fribourg (FR), Geneva (GE), Glarus (GL), Grisons (GR), Jura (JU), Lucerne (LU), Neuchâtel (NE), Nidwalden (NW), Obwalden (OW), St. Gallen (SG), Schaffhausen (SH), Solothurn (SO), Schwyz (SZ), Thurgau (TG), Ticino (TI), Uri (UR), Vaud (VD), Valais (VS), Zug (ZG), Zurich (ZH)

21. If the secondary residence has a garden, who primarily maintains it?
    1/ It does not have a garden **(→ to question 24)**
    2/ Me, personally
    3/ A member of my family
    4/ A non-professional person

5/ A professional

22. Does the garden in question have one or more windmill palms?
    1/ Yes
    2/ No, but I would like to have some **(→ to question 24)**
    3/ No, and I do not want any **(→ to question 24)**

23. How many windmill palms are there in the garden in question?
    1/ A few (1-3)
    2/ Some (4-10)
    3/ A lot (>10)

24. Are you aware that windmill's palm specimens produce only one type of flower: either male or female? Therefore, not all of them produce seeds.
    1/ Yes
    2/ No **(→ to question 26)**

25. Can you distinguish the inflorescences?
    1/ Yes
    2/ No

26. Take a moment to think about the windmill palm. What is the first word that spontaneously comes to your mind? …………

27. Do you like the windmill palm?
    1/ Yes
    2/ Rather yes
    3/ Neither yes nor no
    4/ Rather no
    5/ No

    Reason for the choice …………

28. Do you wish there were more windmill palms in public places?
    1/ Yes
    2/ Rather yes
    3/ Neither yes nor no
    4/ Rather no
    5/ No

29. In your opinion, does the presence of the windmill palm in public and private places affect the surrounding natural environments in any way?
    1/ Yes
    2/ No
    3/ I do not know

30. Overall, how do you rate the impact of the windmill palm on Swiss natural environments?

1/ Very positive
2/ Positive
3/ Neither positive nor negative
4/ Negative
5/ Very negative
6/ I do not have an opinion

Here below we present a series of statements regarding the windmill palm. Do you agree with these statements?

31. The windmill palm is a symbol representing the Insubric Lakes region, of which Lakes Maggiore (Locarno), Ceresio (Lugano) and Lario (Como) are part of.
    1/ Agree
    2/ Rather agree
    3/ Neither agree nor disagree
    4/ Rather disagree
    5/ Disagree

32. The windmill palm is a symbol representing Ticino in Switzerland.
    1/ Agree
    2/ Rather agree
    3/ Neither agree nor disagree
    4/ Rather disagree
    5/ Disagree

33. In Ticino, the windmill palm is a touristic attraction.
    1/ Agree
    2/ Rather agree
    3/ Neither agree nor disagree
    4/ Rather disagree
    5/ Disagree

34. For each of the following pairs of images, indicate which one you prefer.

Edge of a forest

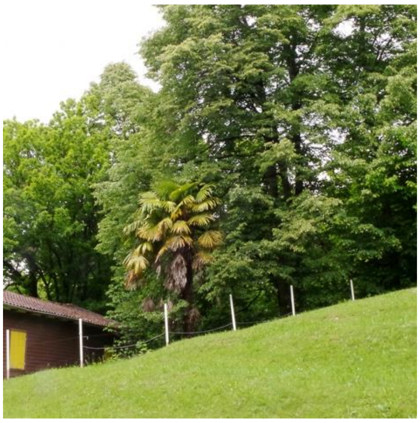
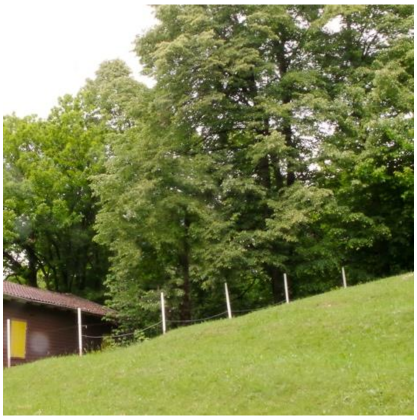

Lakeside

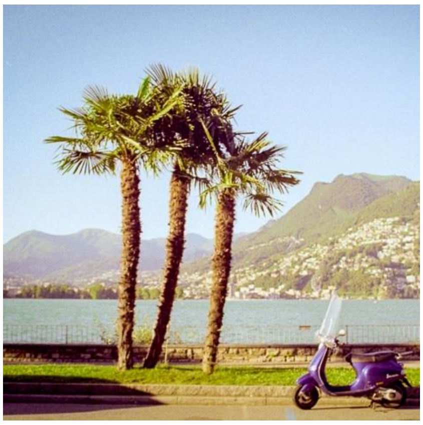
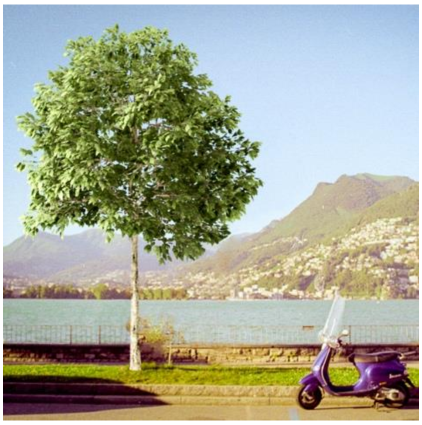

Courtyard

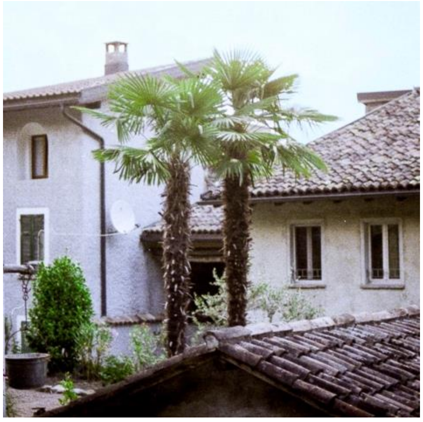
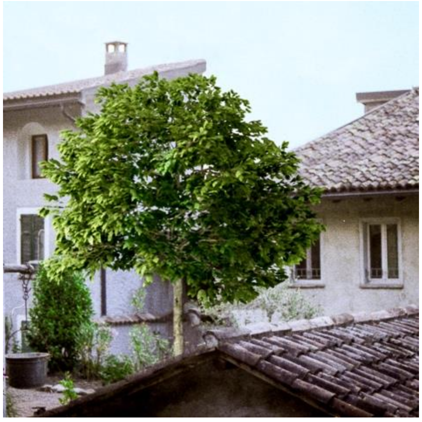

35. Have you heard the term "invasive alien species" before?
    1/ Yes, and I know its meaning
    2/ Yes, but I do not know its meaning
    3/ No [→ to info paragraph]

36. In which context have you heard the term "invasive alien species"?
    1/ On TV
    2/ On the radio
    3/ On a newspaper
    4/ On a magazine
    5/ On the internet
    6/ An acquaintance told me about it
    7/ In the work/training environment
    8/ Other …………

The windmill palm is a species native to East Asia and introduced in Europe after 1500 CE (hence, the term **alien species**). It produces many seeds which easily germinate and are spread in the environment by birds or by an inadequate management of vegetable waste. Because of this, the windmill palm has become **invasive**, diffusing through the woods from public and private gardens and adapting to several conditions. It is listed on Infoflora's **blacklist** of invasive neophytes.

Scientific research is currently ongoing to quantify the **effects** of the windmill palm in forests, where it competes with local vegetation. In fact, it could prevent regeneration and reduce important forest functions in the long term, such as protection from natural hazards, biodiversity, wood production and recreational function for the population.

Annual **removal** of the female inflorescences of the windmill palm would be an effective measure to prevent the spread of the species in natural environments.

The federal government spends several tens of millions of Swiss francs each year on nature conservation and forest biodiversity. The **budget** for this purpose has **doubled** in recent years to CHF 77 million in 2020. The Cantons contribute at least equally.

We are now re-presenting two topics asked previously.

37. Would you like to have one or more windmill palms in your garden or in the garden of your building?
    1/ Yes
    2/ Rather yes
    3/ Neither yes nor no
    4/ Rather no
    5/ No
    6/ I do not have a garden

38. Do you wish there were more windmill palms in public places?
    1/ Yes
    2/ Rather yes
    3/ Neither yes nor no
    4/ Rather no
    5/ No

39. Would you be willing to take action to remove the female inflorescences of the windmill palm on your property?
    1/ Yes, I would do it by myself
    2/ Yes, I would hire someone to do it
    3/ I would like to, but I do not have the possibility
    4/ No
    5/ I do not have windmill palms

40. Here again there is an image of the windmill palm

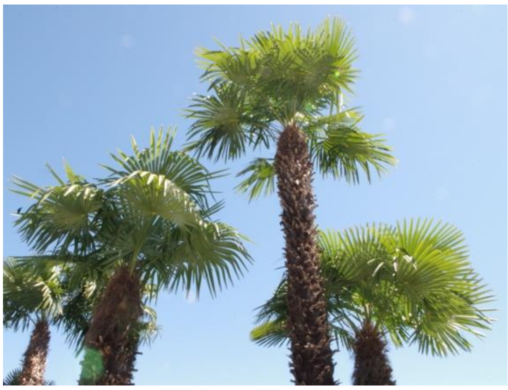

For each of the exotic species shown in the next photographs, please indicate whether you would like to see it in public and private places instead of the windmill palm. The proposed species are not considered invasive in Switzerland.

You must answer quickly. In fact, you only have 8 seconds for each picture. To answer YES you must press 1 (one) key on your keyboard. To answer NO, press 0 (zero).

To test your keyboard, press for YES
To test your keyboard, press for NO.

Please answer all questions before you change the page, otherwise your answers will not be recorded.

Would you like to see this species instead of the windmill palm?

Remember the choice: 1 = YES / 0 = NO

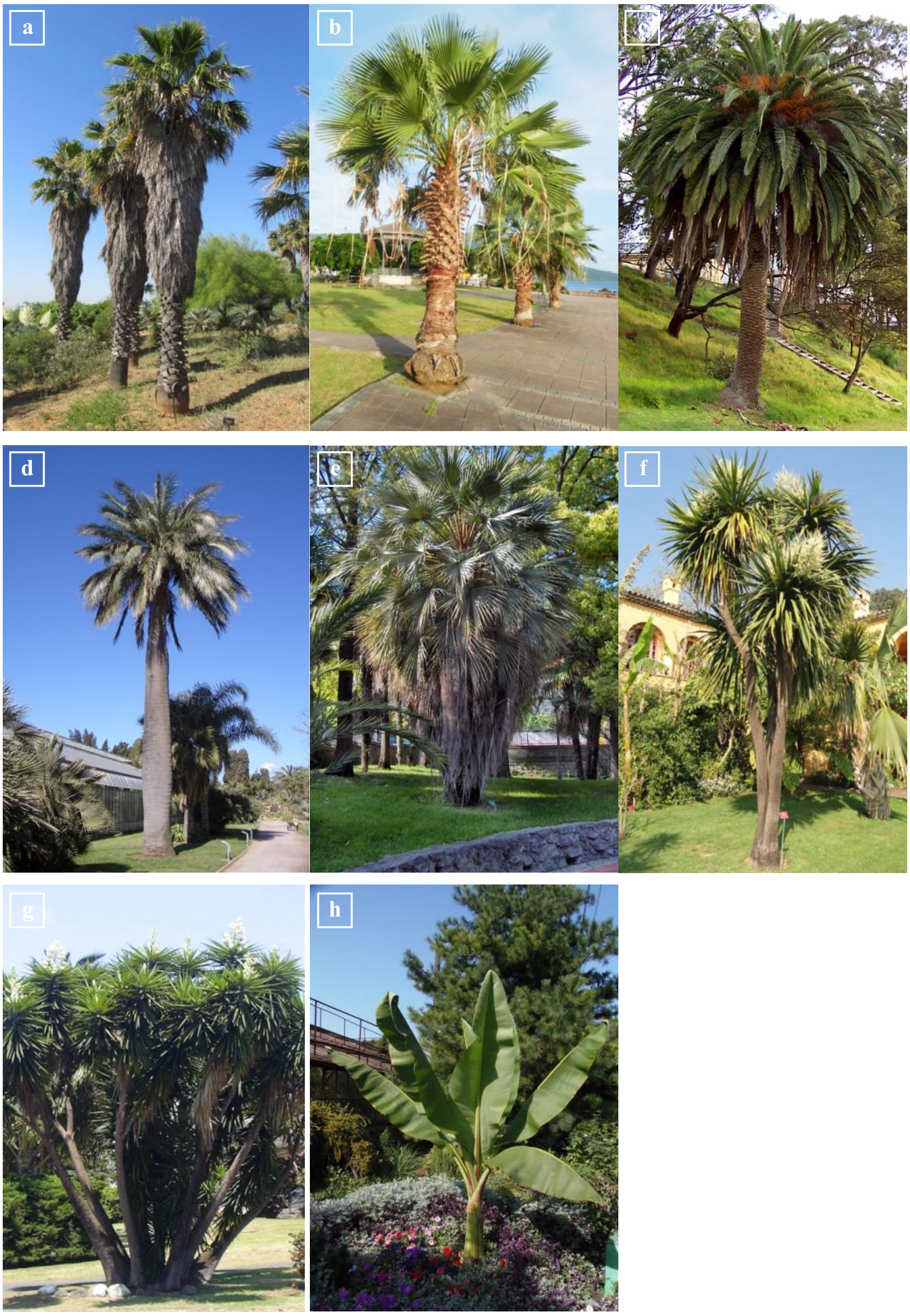
a
b
d
f
h

Here below we present a series of statements regarding the windmill palm. Do you agree with these statements?

41. The population is sufficiently informed concerning the topic of invasive species.
    1/ Agree
    2/ Rather agree
    3/ Neither agree nor disagree
    4/ Rather disagree
    5/ Disagree

42. Federal and cantonal authorities should invest more in management strategies for invasive neophytes, which the windmill palm is an example of.
    1/ Agree
    2/ Rather agree
    3/ Neither agree nor disagree
    4/ Rather disagree
    5/ Disagree

43. It is right to intervene to prevent the spread of the windmill palm outside private and public gardens.
    1/ Agree
    2/ Rather agree
    3/ Neither agree nor disagree
    4/ Rather disagree
    5/ Disagree

44. We now present a series of measures regarding the management of the windmill palm. Please indicate the extent to which you believe these options are appropriate.

	Inappropriate				Appropriate
	1	2	3	4	5
1/ Monitoring the spread					
2/ Awareness campaigns in favor of an appropriate management					
3/ Eradication in natural environments					
4/ Prohibition in all public places					
5/ Prohibition of any kind of trade					
6/ Prohibition in all private places					

To conclude the questionnaire, we ask you for some personal information.

45. Are you part of an association/organization involved in environmental protection?
    1/ Yes
    2/ No **(→ to question 47)**

46. What association/organization is it? …………

47. Last educational qualification obtained
    1/ None
    2/ Elementary school, middle school
    3/ Vocational education and training (VET), specialized school
    4/ High school
    5/ Professional education and training colleges (PET)
    6/ University of applied sciences (UAS), university of teacher education (UTE)
    7/ University, institute of technology

48. Current occupation
    1/ In training
    2/ Primary sector
    3/ Secondary sector
    4/ Tertiary sector
    5/ Housewife/husband
    6/ Retired
    7/ Unemployed

49. Is or was your profession related to the environment?
    1/ Yes
    2/ No **(→ to the ending text)**

50. What is the profession? …………

Thank you for taking the time to complete this questionnaire, your answers will be of great help to us. We remind you that your information will be treated confidentially. If you wish, do not hesitate to leave a comment here below.

Italian version

Il presente questionario ha lo scopo di investigare la percezione e le conoscenze che la popolazione svizzera ha riguardo alla palma di Fortune (*Trachycarpus fortunei*), visibile nell'immagine qui sotto.

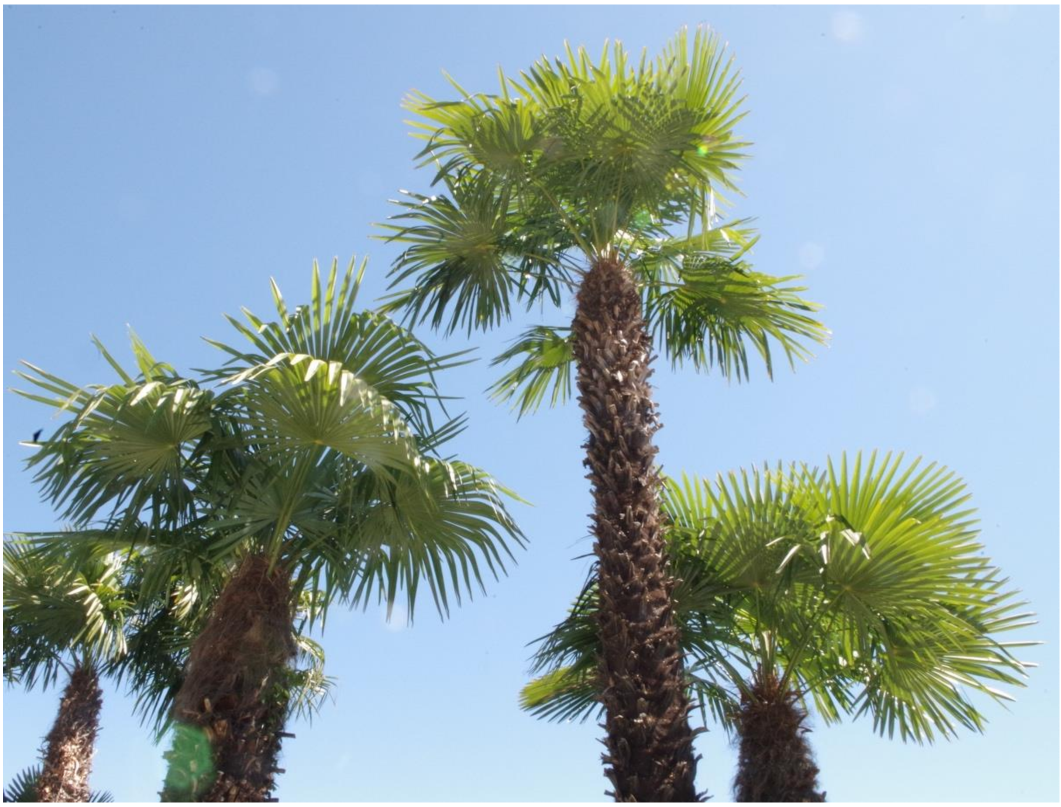

La compilazione del questionario è volontaria e in forma anonima, le informazioni personali in esso contenute servono unicamente a fini statistici. Per completarlo occorrono 15 minuti. Esso è parte di una tesi di Master condotta al Politecnico Federale di Zurigo (ETHZ) in collaborazione con l'Istituto federale di ricerca per la foresta, la neve e il paesaggio (WSL, sede di Cadenazzo).

WSL

ETH zürich

1. Cantone di origine
   Argovia (AG), Appenzello Interno (AI), Appenzello Esterno (AR), Berna (BE), Basilea Campagna (BL), Basilea Città (BS), Friborgo (FR), Ginevra (GE), Glarona (GL), Grigioni (GR), Giura (JU), Lucerna (LU), Neuchâtel (NE), Nidvaldo (NW), Obvaldo (OW), San Gallo (SG), Sciaffusa (SH), Soletta (SO), Svitto (SZ), Turgovia (TG), Ticino (TI), Uri (UR), Vaud (VD), Vallese (VS), Zugo (ZG), Zurigo (ZH)

2. Cantone di residenza permanente
   Argovia (AG), Appenzello Interno (AI), Appenzello Esterno (AR), Berna (BE), Basilea Campagna (BL), Basilea Città (BS), Friborgo (FR), Ginevra (GE), Glarona (GL), Grigioni (GR), Giura (JU), Lucerna (LU), Neuchâtel (NE), Nidvaldo (NW), Obvaldo (OW), San Gallo (SG), Sciaffusa (SH), Soletta (SO), Svitto (SZ), Turgovia (TG), Ticino (TI), Uri (UR), Vaud (VD), Vallese (VS), Zugo (ZG), Zurigo (ZH)

3. Dove vive?
   1/ Spazio urbano: centro
   2/ Spazio urbano: periferia
   3/ Spazio rurale o regione di montagna

4. Lingua madre
   1/ Italiano
   2/ Francese
   3/ Tedesco
   4/ Romancio
   5/ Altro …………

5. Anno di nascita …………

2002	2001	2000	1999	1998	1997	1996	1995	1994	1993
1992	1991	1990	1989	1988	1987	1986	1985	1984	1983
1982	1981	1980	1979	1978	1977	1976	1975	1974	1973
1972	1971	1970	1969	1968	1967	1966	1965	1964	1963
1962	1961	1960	1959	1958	1957	1956	1955	1954	1953
1952	1951	1950	1949	1948	1947	1946	1945	1944	1943
1942	1941	1940	1939	1938	1937	1936	1935	1934	1933
1932	1931	1930	1929	1928	1927	1926	1925	1924	1923
1922	1921	1920							

6. Genere
   1/ Donna
   2/ Uomo
   3/ Altro …………

7. Di dove pensa sia originaria la palma di Fortune?
   1/ Africa
   2/ Asia
   3/ Europa
   4/ Nord America
   5/ Oceania

6/ Sud America

8. In Svizzera, ha già potuto vedere questa palma?
   1/ Sì
   2/ No **(→ to question 11)**

9. In quali Cantoni svizzeri l'ha già vista?
   Argovia (AG), Appenzello Interno (AI), Appenzello Esterno (AR), Berna (BE), Basilea Campagna (BL), Basilea Città (BS), Friborgo (FR), Ginevra (GE), Glarona (GL), Grigioni (GR), Giura (JU), Lucerna (LU), Neuchâtel (NE), Nidvaldo (NW), Obvaldo (OW), San Gallo (SG), Sciaffusa (SH), Soletta (SO), Svitto (SZ), Turgovia (TG), Ticino (TI), Uri (UR), Vaud (VD), Vallese (VS), Zugo (ZG), Zurigo (ZH)

10. Indichi in quale ambiente/luogo ha già visto la palma di Fortune.
    1/ Giardino privato
    2/ Giardino pubblico (parco)
    3/ Bordo lago
    4/ Bordo fiume
    5/ Margine di un bosco
    6/ All'interno di un bosco
    7/ Bordo di una strada
    8/ Negozio
    9/ Altro

11. Le piacerebbe vedere la palma di Fortune negli ambienti/luoghi elencati?

	Sì	Piuttosto sì	Né sì né no	Piuttosto no	No
	1	2	3	4	5
1/ Giardino privato					
2/ Giardino pubblico (parco)					
3/ Bordo lago					
4/ Bordo fiume					
5/ Margine di un bosco					
6/ All'interno di un bosco					
7/ Bordo di una strada					
8/ Negozio					

12. Ci sono una o più palme di Fortune nelle proprietà adiacenti alla sua abitazione primaria?
    1/ Sì
    2/ No **(→ to question 14)**
    3/ Non lo so **(→ to question 14)**

13. Quante palme di Fortune ci sono nelle proprietà adiacenti alla sua abitazione primaria?
    1/ Poche (1-3)
    2/ Alcune (4-10)

3/ Molte (>10)

14. Se ha un giardino privato, o se il suo condominio ne ha uno, chi lo cura principalmente?
    1/ Non ho un giardino privato / Il condominio non ha un giardino privato **(→ to question 19)**
    2/ Io personalmente
    3/ Un membro della mia famiglia
    4/ Un incaricato non professionista
    5/ Un professionista

15. Ci sono palme di Fortune nel suo giardino o in quello del suo condominio?
    1/ Sì
    2/ No, ma vorrei averne **(→ to question 19)**
    3/ No, e non vorrei averne **(→ to question 19)**
    4/ Non lo so **(→ to question 19)**

16. Quante palme di Fortune ci sono nel suo giardino o in quello del suo condominio?
    1/ Poche (1-3)
    2/ Alcune (4-10)
    3/ Molte (>10)

17. La decisione di piantare queste palme di Fortune è stata sua o di un membro della sua famiglia?
    1/ Sì
    2/ No **(→ to question 19)**

18. Dove se le è procurate? …………

19. Lei o la sua famiglia possedete una residenza secondaria in Svizzera?
    1/ Sì
    2/ No **(→ to question 24)**

20. In quale Cantone?
    Argovia (AG), Appenzello Interno (AI), Appenzello Esterno (AR), Berna (BE), Basilea Campagna (BL), Basilea Città (BS), Friborgo (FR), Ginevra (GE), Glarona (GL), Grigioni (GR), Giura (JU), Lucerna (LU), Neuchâtel (NE), Nidvaldo (NW), Obvaldo (OW), San Gallo (SG), Sciaffusa (SH), Soletta (SO), Svitto (SZ), Turgovia (TG), Ticino (TI), Uri (UR), Vaud (VD), Vallese (VS), Zugo (ZG), Zurigo (ZH)

21. Se la residenza secondaria ha un giardino, chi lo cura principalmente?
    1/ Non ha un giardino **(→ to question 24)**
    2/ Io personalmente
    3/ Un membro della mia famiglia
    4/ Un incaricato non professionista
    5/ Un professionista

22. Il giardino in questione ha una o più palme di Fortune?

1/ Sì
2/ No, ma vorrei averne **(→ to question 24)**
3/ No, e non vorrei averne **(→ to question 24)**

23. Quante palme di Fortune ci sono nel giardino in questione?
1/ Poche (1-3)
2/ Alcune (4-10)
3/ Molte (>10)

24. È a conoscenza del fatto che gli esemplari di palma di Fortune producono un solo tipo di fiori: o maschili o femminili? Non tutti quindi producono dei semi.
1/ Sì
2/ No **(→ to question 26)**

25. È in grado di distinguere le infiorescenze?
1/ Sì
2/ No

26. Si prenda un momento per pensare alla palma di Fortune. Qual è la prima parola che spontaneamente le viene in mente?
…………

27. Le piace la palma di Fortune?
1/ Sì
2/ Piuttosto sì
3/ Né sì né no
4/ Piuttosto no
5/ No

Motivo della scelta …………

28. Vorrebbe ci fossero più palme di Fortune nei luoghi pubblici?
1/ Sì
2/ Piuttosto sì
3/ Né sì né no
4/ Piuttosto no
5/ No

29. Secondo lei, la presenza della palma di Fortune nei luoghi pubblici e privati influenza in qualche modo gli ambienti naturali circostanti?
1/ Sì
2/ No
3/ Non lo so

30. In generale, come valuta l'impatto della palma di Fortune sugli ambienti naturali svizzeri?
1/ Molto negativo
2/ Negativo
3/ Né negativo né positivo

4/ Positivo
5/ Molto positivo
6/ Non ho un'opinione al riguardo

Di seguito presentiamo una serie di affermazioni riguardanti la palma di Fortune. È d'accordo con queste affermazioni?

31. La palma di Fortune è un simbolo che rappresenta la regione dei laghi insubrici, di cui i laghi Maggiore (Locarno), Ceresio (Lugano) e Lario (Como) fanno parte.
    1/ Molto d'accordo
    2/ Piuttosto d'accordo
    3/ Né d'accordo né in disaccordo
    4/ Piuttosto in disaccordo
    5/ Molto in disaccordo

32. La palma di Fortune è un simbolo che rappresenta il Ticino in Svizzera.
    1/ Molto d'accordo
    2/ Piuttosto d'accordo
    3/ Né d'accordo né in disaccordo
    4/ Piuttosto in disaccordo
    5/ Molto in disaccordo

33. In Ticino, la palma di Fortune è un'attrazione per i turisti.
    1/ Molto d'accordo
    2/ Piuttosto d'accordo
    3/ Né d'accordo né in disaccordo
    4/ Piuttosto in disaccordo
    5/ Molto in disaccordo

34. Per ognuna delle seguenti coppie di fotografie, indichi quale preferisce.

Edge of a forest

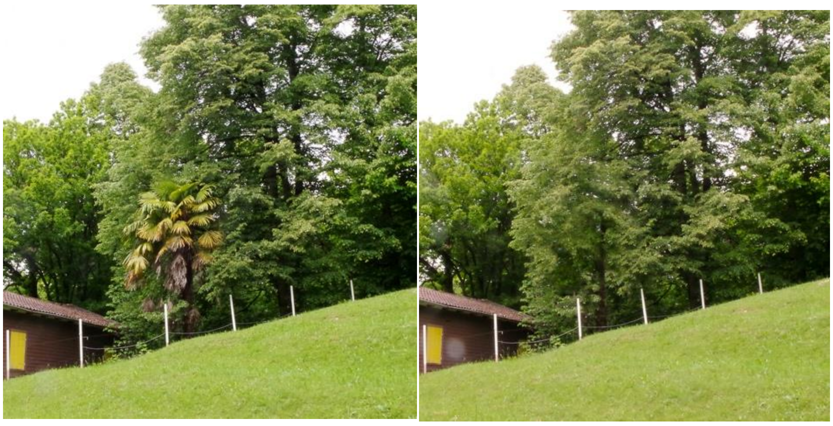

Longlake

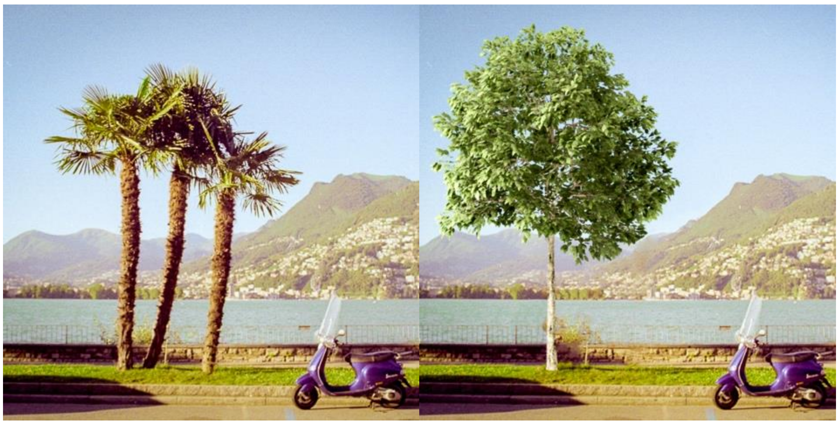

Courtyard

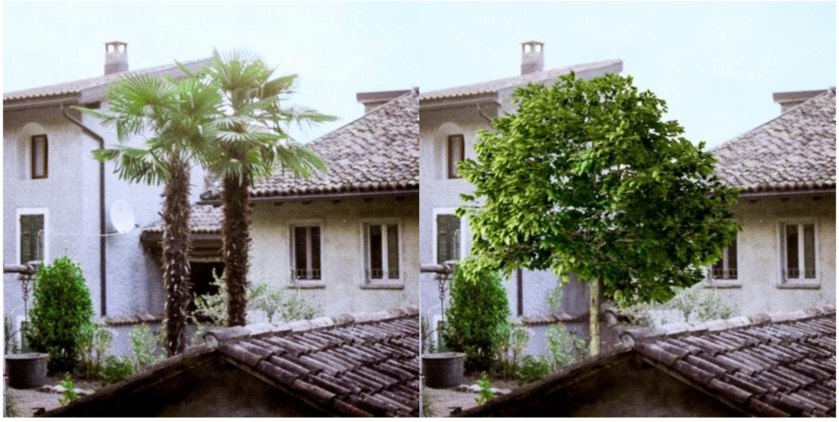

35. Ha già sentito il termine «neofita invasiva»?
    1/ Sì, e ne conosco il significato
    2/ Sì, ma non ne conosco il significato
    3/ No [→ to info paragraph]

36. In quale contesto ha sentito il termine «neofita invasiva»?
    1/ Alla televisione
    2/ Alla radio
    3/ Su un quotidiano
    4/ Su una rivista
    5/ Su internet
    6/ Un conoscente me ne ha parlato
    7/ In ambito lavorativo / di formazione
    8/ Altro …………

La palma di Fortune è una specie originaria dell'est asiatico introdotta in Europa dall'uomo dopo il 1500 d.C. (da qui il termine **neofita**). Produce molti semi che germinano facilmente e sono disseminati nell'ambiente da uccelli o da una gestione non adeguata degli scarti vegetali. Per questo motivo la palma di Fortune è diventata **invasiva**, diffondendosi nel bosco partendo da giardini pubblici e privati e adattandosi a diverse condizioni. È elencata nella **lista nera** di Infoflora delle neofite invasive.

Attualmente sono in corso ricerche scientifiche per quantificare gli **effetti** della palma di Fortune negli ambienti boschivi, dove entra in concorrenza con la vegetazione locale. Potrebbe infatti impedirne la rinnovazione e ridurre a lungo termine le importanti funzioni dei boschi, come la protezione dai pericoli naturali, la biodiversità, la produzione di legna e la funzione ricreativa per la popolazione.

La **rimozione** annuale delle infiorescenze femminili della palma di Fortune sarebbe una misura efficace per impedire la diffusione della specie negli ambienti naturali.

Per la protezione della natura e per la biodiversità forestale, la Confederazione spende ogni anno diverse decine di milioni di franchi. Il **budget** destinato a questo scopo è **raddoppiato** negli ultimi anni giungendo a 77 milioni di CHF nel 2020. I Cantoni contribuiscono almeno in egual misura.

Le ripresentiamo ora due domande poste in precedenza.

37. Le piacerebbe avere una o più palme di Fortune nel suo giardino o nel giardino del suo palazzo?
    1/ Sì
    2/ Piuttosto sì
    3/ Né sì né no
    4/ Piuttosto no
    5/ No
    6/ Non ho un giardino

38. Vorrebbe ci fossero più palme di Fortune nei luoghi pubblici?
    1/ Sì
    2/ Piuttosto sì
    3/ Né sì né no
    4/ Piuttosto no
    5/ No

39. Sarebbe disposto ad attivarsi per la rimozione delle infiorescenze femminili della palma di Fortune sulla sua proprietà?
    1/ Sì, lo farei io stesso
    2/ Sì, incaricherei qualcuno per farlo
    3/ Vorrei, ma non ne ho la possibilità
    4/ No
    5/ Non possiedo palme di Fortune

40. Ecco nuovamente un’immagine della palma di Fortune

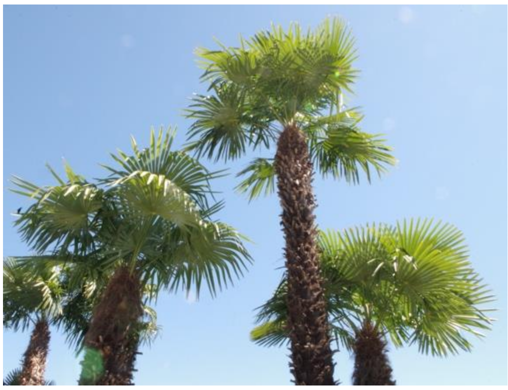

Per ognuna delle specie esotiche mostrate nelle prossime fotografie, indichi se le piacerebbe vederla nei luoghi pubblici e privati al posto della palma di Fortune. Le specie proposte non sono considerate invasive in Svizzera.

Deve rispondere velocemente. Infatti, ha solo 8 secondi di tempo per ogni immagine. Per rispondere SI deve premere il tasto 1 (uno) sulla tastiera. Per rispondere NO deve premere il tasto 0 (zero).

Per testare la sua tastiera prema adesso per il SI
Per testare la sua tastiera prema adesso per il NO

Per favore risponda a tutte le domande prima di cambiare pagina, altrimenti le sue risposte non saranno registrate.

Vorrebbe vedere questa specie al posto della palma di Fortune?

Si ricordi la scelta: 1 = SI / 0 = NO

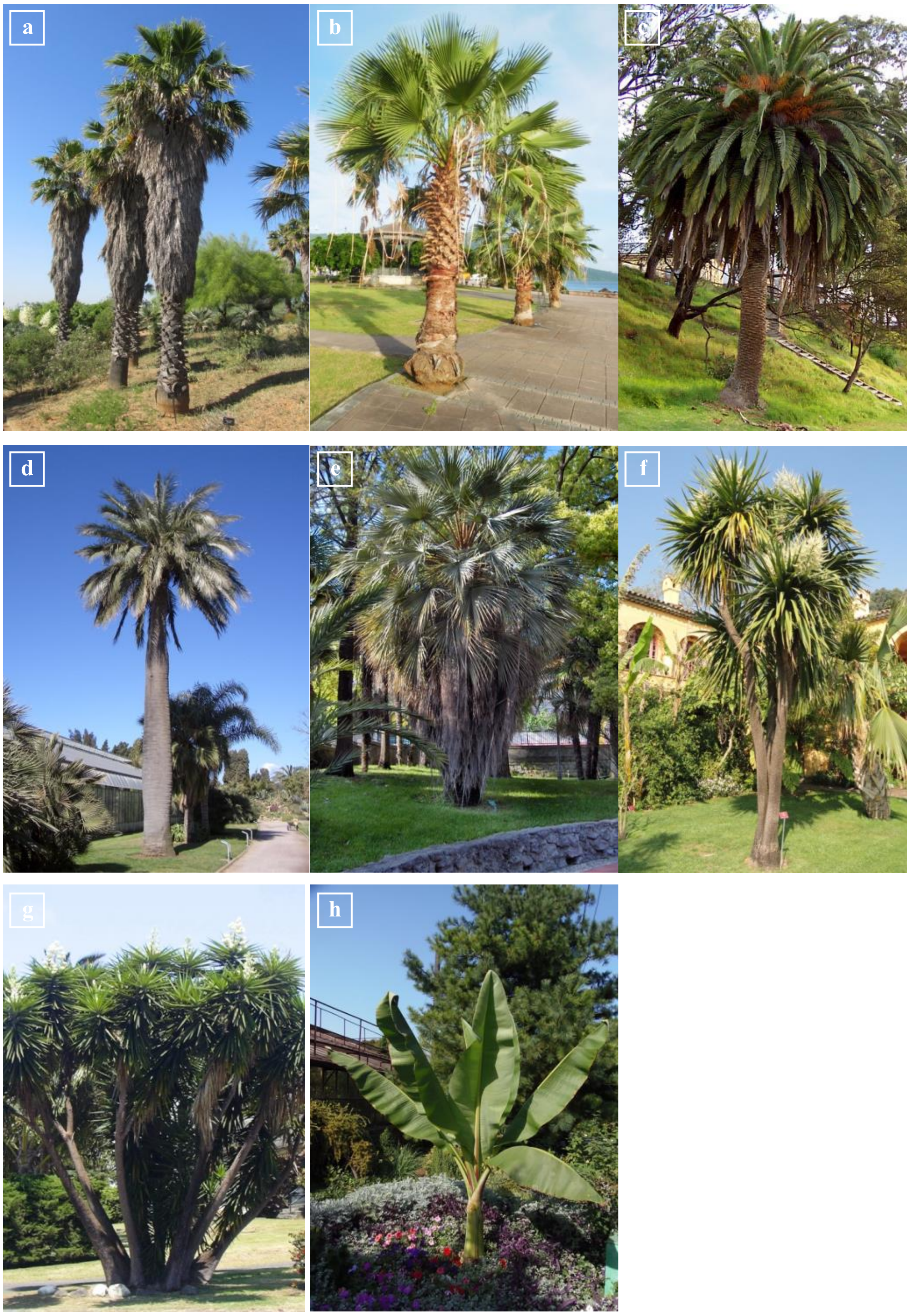
a
b
c
d
e
f
g
h

Di seguito presentiamo una serie di affermazioni riguardanti la palma di Fortune. È d'accordo con queste affermazioni?

41. La popolazione è sufficientemente informata riguardo al tema delle specie invasive.
    1/ Molto d'accordo
    2/ Piuttosto d'accordo
    3/ Né d'accordo né in disaccordo
    4/ Piuttosto in disaccordo
    5/ Molto in disaccordo

42. Le autorità federali e cantonali devono investire di più nelle strategie di gestione delle neofite invasive, di cui la palma di Fortune fa parte.
    1/ Molto d'accordo
    2/ Piuttosto d'accordo
    3/ Né d'accordo né in disaccordo
    4/ Piuttosto in disaccordo
    5/ Molto in disaccordo

43. È giusto intervenire per evitare la diffusione della palma di Fortune fuori dai giardini e dai parchi pubblici.
    1/ Molto d'accordo
    2/ Piuttosto d'accordo
    3/ Né d'accordo né in disaccordo
    4/ Piuttosto in disaccordo
    5/ Molto in disaccordo

44. Ora presentiamo una serie di misure riguardanti la gestione della palma di Fortune. Indichi in che misura ritiene queste opzioni appropriate.

	Non appropriata				Appropriata
	1	2	3	4	5
1/ Monitoraggio della diffusione					
2/ Campagne di sensibilizzazione a favore di una gestione adeguata					
3/ Interventi di eradicazione in ambienti naturali					
4/ Proibizione in tutti i luoghi pubblici					
5/ Proibizione di ogni tipo di commercio					
6/ Proibizione in tutti i luoghi privati					

Per concludere il questionario, le chiediamo qualche informazione personale.

45. Fa parte di un'associazione/organizzazione che si occupa di tutela dell'ambiente?
    1/ Sì
    2/ No **(→ to question 47)**

46. Di quale associazione/organizzazione si tratta? …………

47. Ultimo titolo di studio ottenuto
    1/ Nessuno
    2/ Scuola elementare e scuola media
    3/ Formazione professionale di base (tirocinio), scuola specializzata
    4/ Scuole di maturità, liceo
    5/ Scuola specializzata superiore (SSS)
    6/ Scuole universitarie professionali (SUP), alte scuole pedagogiche (ASP)
    7/ Università e politecnici

48. Attuale occupazione
    1/ In formazione
    2/ Settore primario
    3/ Settore secondario
    4/ Settore terziario
    5/ Casalinga/o
    6/ Pensionata/o
    7/ Disoccupata/o

49. La sua professione è o era legata all'ambiente?
    1/ Sì
    2/ No **(→ to ending text)**

50. Di che professione si tratta? …………

La ringraziamo per il tempo messo a disposizione per completare il questionario, le sue risposte ci saranno di grande aiuto. Le ricordiamo che le informazioni saranno trattate confidenzialmente. Se lo desidera, non esiti a lasciare un commento qui di seguito.

German version

Der Zweck dieses Fragebogens ist es, die Wahrnehmung und das Wissen der Schweizer Bevölkerung über die Fortunes Hanfpalme (*Trachycarpus fortunei*), die auf dem Bild unten zu sehen ist, zu untersuchen.

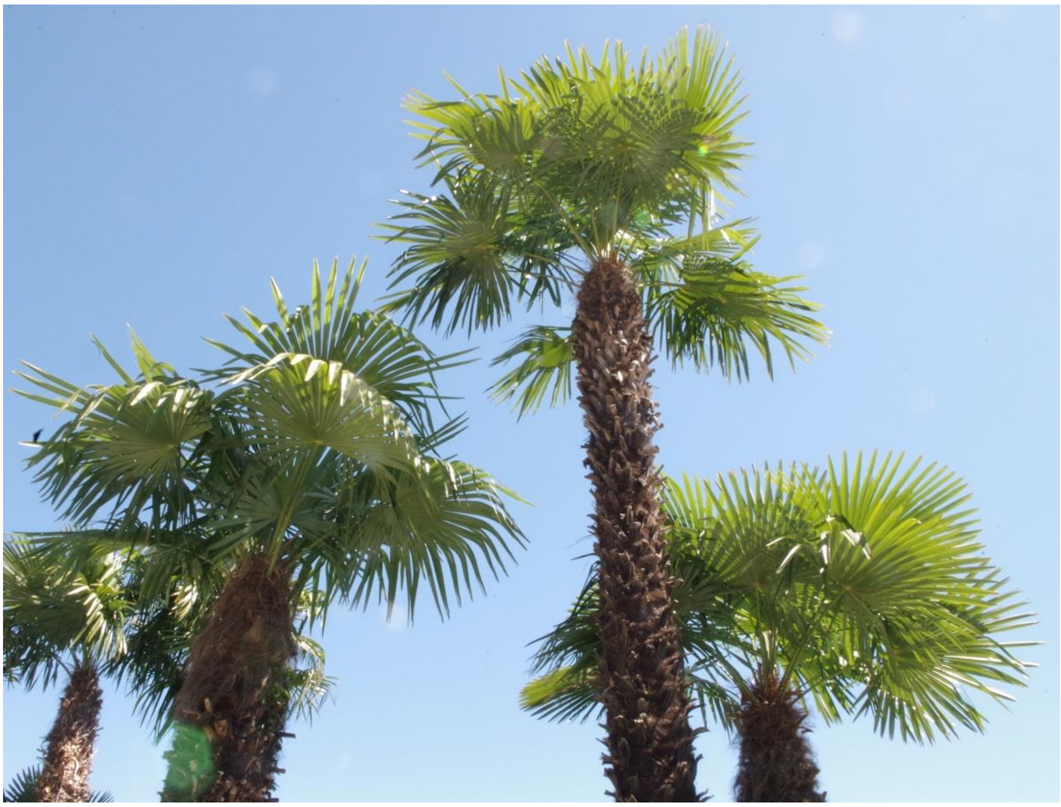

Das Ausfüllen des Fragebogens ist freiwillig und anonym, die darin enthaltenen persönlichen Daten dienen ausschließlich statistischen Zwecken. Es dauert 15 Minuten.
Die Umfrage, ist Teil einer Masterarbeit, die an der Eidgenössischen Technischen Hochschule Zürich (ETHZ) in Zusammenarbeit mit der Eidgenössischen Forschungsanstalt für Wald, Schnee und Landschaft (WSL, Standort Cadenazzo) durchgeführt wird.

WSL

ETH zürich

1. Heimatkanton
   Aargau (AG), Appenzell Innerrhoden (AI), Appenzell Ausserrhoden (AR), Bern (BE), Basel-Landschaft (BL), Basel-Stadt (BS), Freiburg (FR), Genf (GE), Glarus (GL), Graubünden (GR), Jura (JU), Luzern (LU), Neuenburg (NE), Nidwalden (NW), Obwalden (OW), St. Gallen (SG), Schaffhausen (SH), Solothurn (SO), Schwyz (SZ), Thurgau (TG), Tessin (TI), Uri (UR), Waadt (VD), Wallis (VS), Zug (ZG), Zürich (ZH)

2. Kanton mit ständigem Wohnsitz
   Aargau (AG), Appenzell Innerrhoden (AI), Appenzell Ausserrhoden (AR), Bern (BE), Basel-Landschaft (BL), Basel-Stadt (BS), Freiburg (FR), Genf (GE), Glarus (GL), Graubünden (GR), Jura (JU), Luzern (LU), Neuenburg (NE), Nidwalden (NW), Obwalden (OW), St. Gallen (SG), Schaffhausen (SH), Solothurn (SO), Schwyz (SZ), Thurgau (TG), Tessin (TI), Uri (UR), Waadt (VD), Wallis (VS), Zug (ZG), Zürich (ZH)

3. Wo wohnen Sie?
   1/ Städtischer Raum: Zentrum
   2/ Städtischer Raum: Vororte
   3/ Ländlicher Raum oder Berggebiete

4. Muttersprache
   1/ Italienisch
   2/ Französisch
   3/ Deutsch
   4/ Rätoromanisch
   5/ Andere …………

5. Geburtsjahr

2002	2001	2000	1999	1998	1997	1996	1995	1994	1993
1992	1991	1990	1989	1988	1987	1986	1985	1984	1983
1982	1981	1980	1979	1978	1977	1976	1975	1974	1973
1972	1971	1970	1969	1968	1967	1966	1965	1964	1963
1962	1961	1960	1959	1958	1957	1956	1955	1954	1953
1952	1951	1950	1949	1948	1947	1946	1945	1944	1943
1942	1941	1940	1939	1938	1937	1936	1935	1934	1933
1932	1931	1930	1929	1928	1927	1926	1925	1924	1923
1922	1921	1920							

6. Geschlecht
   1/ Weiblich
   2/ Männlich
   3/ Andere …………

7. Woher stammt Ihrer Meinung nach die Hanfpalme?
   1/ Afrika
   2/ Asien
   3/ Europa

4/ Nordamerika
5/ Ozeanien
6/ Südamerika

8. Haben Sie die Hanfpalme in der Schweiz schon gesehen?
1/ Ja
2/ Nein **(→ to question 11)**

9. In welchen Schweizer Kantonen haben Sie die Hanfpalme schon gesehen?
Aargau (AG), Appenzell Innerrhoden (AI), Appenzell Ausserrhoden (AR), Bern (BE), Basel-Landschaft (BL), Basel-Stadt (BS), Freiburg (FR), Genf (GE), Glarus (GL), Graubünden (GR), Jura (JU), Luzern (LU), Neuenburg (NE), Nidwalden (NW), Obwalden (OW), St. Gallen (SG), Schaffhausen (SH), Solothurn (SO), Schwyz (SZ), Thurgau (TG), Tessin (TI), Uri (UR), Waadt (VD), Wallis (VS), Zug (ZG), Zürich (ZH)

10. Bitte geben Sie an, in welcher Umgebung/an welchem Ort Sie die Hanfpalme bereits gesehen haben.
1/ Privater Garten
2/ Öffentlicher Garten (Park)
3/ Seeufer
4/ Flussufer
5/ Rand eines Waldes
6/ Innerhalb eines Waldes
7/ Straßenrand
8/ Laden
9/ Andere

11. Möchten Sie die Hanfpalme in der aufgeführten Umgebung/Ort sehen?

	Ja	Eher ja	Weder noch	Eher nein	Nein
	1	2	3	4	5
1/ Privater Garten					
2/ Öffentlicher Garten (Park)					
3/ Seeufer					
4/ Flussufer					
5/ Rand eines Waldes					
6/ Innerhalb eines Waldes					
7/ Straßenrand					
8/ Laden					

12. Gibt es auf den Grundstücken neben Ihrem Hauptwohnsitz eine oder mehrere Hanfpalmen?
1/ Ja
2/ Nein **(→ to question 14)**
3/ Ich weiss es nicht **(→ to question 14)**

13. Wie viele Hanfpalmen gibt es auf den Grundstücken neben Ihrem Hauptwohnsitz?

1/ Wenige (1-3)
2/ Manche (4-10)
3/ Viele (>10)

14. Falls Sie, oder das Gebäude, in dem Sie wohnen, einen eigenen Garten haben: Wer kümmert sich darum in erster Linie?
    1/ Ich habe keinen eigenen Garten / Das Gebäude, in dem ich Wohne, hat keinen eigenen Garten **(→ to question 19)**
    2/ Ich persönlich
    3/ Ein Mitglied meiner Familie
    4/ Ein nichtberuflicher Beauftragter
    5/ Ein Fachmann

15. Gibt es Hanfpalmen in Ihrem Garten oder in Ihrem Gebäude?
    1/ Ja
    2/ Nein, aber ich hätte gerne welche **(→ to question 19)**
    3/ Nein, und ich will keine **(→ to question 19)**
    4/ Ich weiss es nicht **(→ to question 19)**

16. Wie viele Hanfpalmen gibt es in Ihrem Garten oder Ihrem Gebäude?
    1/ Wenige (1-3)
    2/ Einige (4-10)
    3/ Viele (>10)

17. War die Entscheidung, diese Hanfpalmen zu pflanzen, Ihre oder von einem Mitglied Ihrer Familie?
    1/ Ja
    2/ Nein **(→ to question 19)**

18. Woher kommen diese Hanfpalmen? …………

19. Haben Sie oder Ihre Familie einen Zweitwohnsitz in der Schweiz?
    1/ Ja
    2/ Nein **(→ to question 24)**

20. In welchem Kanton?
    Aargau (AG), Appenzell Innerrhoden (AI), Appenzell Ausserrhoden (AR), Bern (BE), Basel-Landschaft (BL), Basel-Stadt (BS), Freiburg (FR), Genf (GE), Glarus (GL), Graubünden (GR), Jura (JU), Luzern (LU), Neuenburg (NE), Nidwalden (NW), Obwalden (OW), St. Gallen (SG), Schaffhausen (SH), Solothurn (SO), Schwyz (SZ), Thurgau (TG), Tessin (TI), Uri (UR), Waadt (VD), Wallis (VS), Zug (ZG), Zürich (ZH)

21. Wenn der Zweitwohnsitz einen Garten hat, wer kümmert sich um ihn in erster Linie?
    1/ Es hat keinen Garten **(→ to question 24)**
    2/ Ich persönlich
    3/ Ein Mitglied meiner Familie
    4/ Ein nichtberuflicher Beauftragter

5/ Ein Fachmann

22. Hat dieser Garten eine oder mehrere Hanfpalmen?
    1/ Ja
    2/ Nein, aber ich hätte gerne welche **(→ to question 24)**
    3/ Nein, und ich will keine **(→ to question 24)**

23. Wie viele Hanfpalmen gibt es in diesem Garten?
    1/ Wenige (1-3)
    2/ Einige (4-10)
    3/ Viele (>10)

24. Ist Ihnen bekannt, dass die Hanfpalmen nur eine Blütenart hervorbringen: entweder männlich oder weiblich und somit nicht alle Saatgut produzieren?
    1/ Ja
    2/ Nein **(→ to question 26)**

25. Können Sie die Blütenstände unterscheiden?
    1/ Ja
    2/ Nein

26. Nehmen Sie sich einen Moment Zeit, um über die Hanfpalme nachzudenken. Was ist das erste Wort, das Ihnen spontan in den Sinn kommt?
    …………

27. Mögen Sie die Hanfpalme?
    1/ Ja
    2/ Eher ja
    3/ Weder noch
    4/ Eher nein
    5/ Nein
    Wie begründen Sie Ihre Wahl? …………

28. Wünschen Sie sich, es gäbe mehr Hanfpalmen auf öffentlichen Orten?
    1/ Ja
    2/ Eher ja
    3/ Weder noch
    4/ Eher nein
    5/ Nein

29. Beeinflusst Ihrer Meinung nach die Präsenz der Hanfpalme an öffentlichen und privaten Orten in irgendeiner Weise die umgebende natürliche Umwelt?
    1/ Ja
    2/ Nein
    3/ Ich weiss es nicht

30. Im Allgemeine, wie schätzen Sie die Auswirkungen der Hanfpalme auf die natürliche Umwelt der Schweiz ein?

1/ Sehr negativ
2/ Negativ
3/ Weder negativ noch positiv
4/ Positiv
5/ Sehr positiv
6/ Dazu habe ich keine Meinung

Im Folgenden stellen wir eine Reihe von Aussagen zur Hanfpalme. Sind Sie mit diesen Aussagen einverstanden?

31. Die Hanfpalme ist ein Symbol, die die Region der Insubrischen Seen, zu denen die Seen Maggiore (Locarno), Ceresio (Lugano) und Lario (Como) gehören, charakterisiert.
    1/ Trifft zu
    2/ Trifft eher zu
    3/ Weder noch
    4/ Trifft eher nicht zu
    5/ Trifft nicht zu

32. Die Hanfpalme ist ein Symbol, die das Tessin in der Schweiz charakterisiert.
    1/ Trifft zu
    2/ Trifft eher zu
    3/ Weder noch
    4/ Trifft eher nicht zu
    5/ Trifft nicht zu

33. Im Tessin die Hanfpalme ist eine Attraktion für Touristen.
    1/ Trifft zu
    2/ Trifft eher zu
    3/ Weder noch
    4/ Trifft eher nicht zu
    5/ Trifft nicht zu

34. Bitte geben Sie für jedes der folgenden Bildpaare an, welches Sie bevorzugen.

Edge of a forest

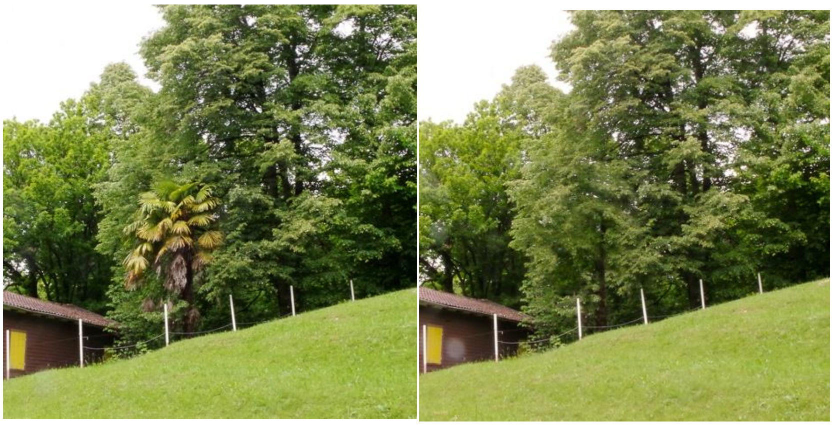

Longlake

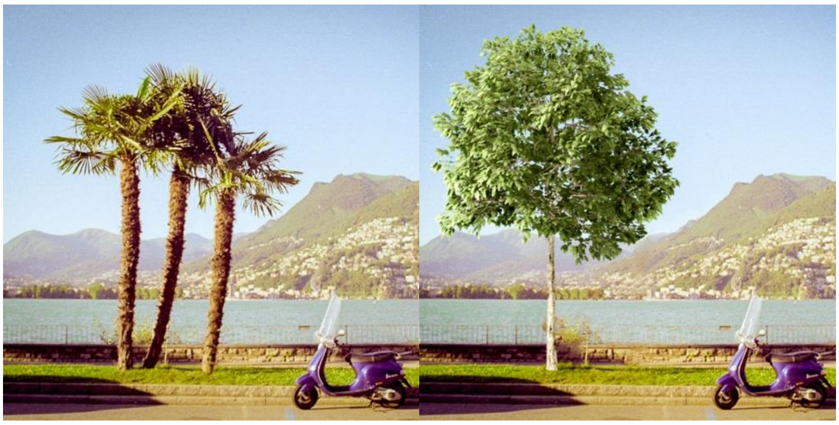

Courtyard

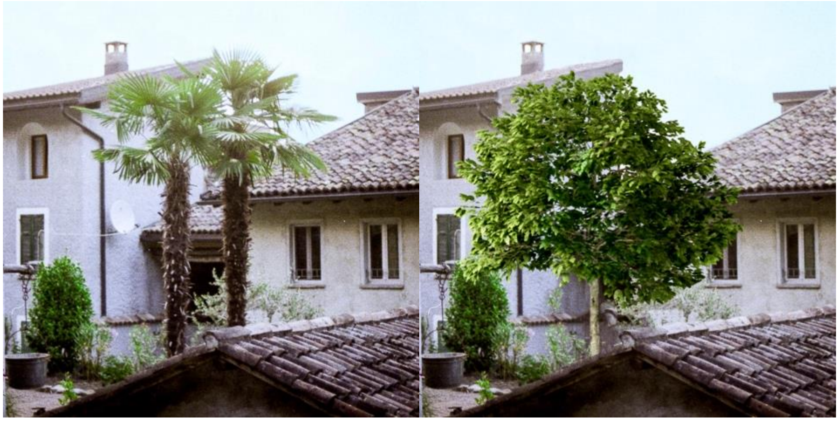

35. Haben Sie den Begriff «invasiven Neophyten» schon gehört?
    1/ Ja, und ich kenne die Bedeutung
    2/ Ja, aber ich kenne die Bedeutung nicht
    3/ Nein [→ to info paragraph]

36. In welchem Kontext haben Sie den Begriff «invasive Neophyten» gehört?
    1/ Im Fernsehen
    2/ Im Radio
    3/ In einer Zeitung
    4/ In einer Zeitschrift
    5/ Im Internet
    6/ Ein Bekannter hat mir davon erzählt
    7/ Am Arbeitsplatz / Ausbildung
    8/ Andere …………

Die Fortunes Hanfpalme ist eine Art, die in Ostasien heimisch ist und vom Menschen nach Europa nach 1500 (daher der Begriff **Neophyten**) eingeführt wurde. Sie produziert viele Früchte, die leicht keimen und leicht durch Vögel oder durch unangemessene Bewirtschaftung von Pflanzenabfällen in der Umwelt verbreitet werden. Aus diesem Grund ist die Fortunes Hanfpalme **invasiv** geworden, hat sich im Wald ausgehend von öffentlichen und privaten Gärten ausgebreitet und sich an unterschiedliche Bedingungen angepasst. Sie steht auf der **schwarzen Liste** invasiver Neophyten von Infoflora.

Gegenwärtig wird wissenschaftliche Forschung betrieben, um die **Auswirkungen** der Fortunes Hanfpalme in Waldgebieten zu quantifizieren, wo sie mit der einheimischen Vegetation konkurriert. Dadurch könnte sie die Waldverjüngung hemmen und die wichtige Funktionen der Wälder, wie der Schutz vor Naturgefahren, die Biodiversität, die Holzversorgung und die Erholungsfunktion für die Bevölkerung, langfristig behindern.

Die jährliche **Entfernung** der weiblichen Blütenstände der Fortunes Hanfpalme wäre eine wirksame Maßnahme, um die Ausbreitung der Art in natürliche Umgebungen zu verhindern.

Für Naturschutz und die Biodiversität der Wälder, gibt der Bund jährlich mehrere zehn Millionen Franken aus. Das dafür vorgesehene **Budget** hat sich in den letzten Jahren auf 77 Millionen CHF im Jahr 2020 **verdoppelt**. Die Kantone tragen mindestens gleichermaßen dazu bei.

Wir stellen Ihnen nun zwei Fragen vor, die Ihnen zuvor gestellt wurden.

37. Möchten Sie eine oder mehrere Hanfpalmen in Ihrem Garten oder im Garten Ihres Hauses haben?
    1/ Ja
    2/ Eher ja
    3/ Weder noch
    4/ Eher nein
    5/ Nein
    6/ Ich habe keinen Garten

38. Wünschen Sie sich, es gäbe mehr Hanfpalmen auf öffentlichen Orte?
    1/ Ja
    2/ Eher ja
    3/ Weder noch
    4/ Eher nein
    5/ Nein

39. Wären Sie bereit, Maßnahmen zu ergreifen, um die weiblichen Blütenstände der Hanfpalme auf Ihrem Grundstück zu entfernen?
    1/ Ja, ich würde es selbst tun
    2/ Ja, ich würde dafür jemanden einstellen
    3/ Ich würde gerne, aber ich habe nicht die Möglichkeit
    4/ Nein
    5/ Ich besitze keine Hanfpalmen

40. Hier ist wieder ein Bild der Hanfpalme.

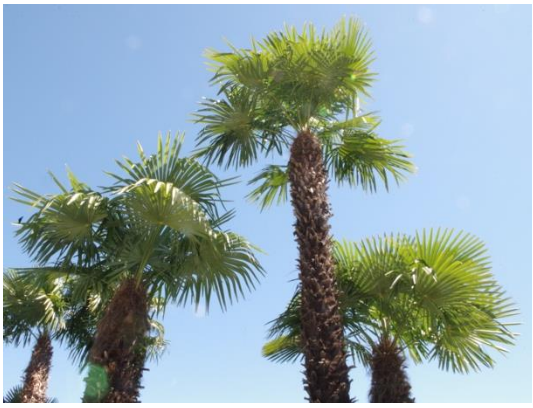

Geben Sie für jede der auf den folgenden Fotos gezeigten exotischen Arten an, ob Sie sie in öffentlichen und privaten Orten anstelle der Hanfpalme sehen möchten. Die vorgeschlagenen Arten werden in der Schweiz nicht als invasive eingestuft.

Sie müssen schnell antworten. Sie haben nur 8 Sekunden pro Bild. Um mit JA zu antworten, drücken Sie die Taste 1 (eins) auf der Tastatur. Um NEIN zu antworten, drücken Sie die Taste 0 (Null).

Um Ihre Tastatur zu testen, drücken Sie JA
Um Ihre Tastatur zu testen, drücken Sie NEIN

Bitte beantworten Sie alle Fragen, bevor Sie die Seiten wechseln, da sonst Ihre Antworten nicht aufgezeichnet werden.

Möchten Sie gerne diese Art anstelle der Hanfpalme sehen?
Hier noch einmal zur Erinnerung: 1 = JA / 0 = NEIN

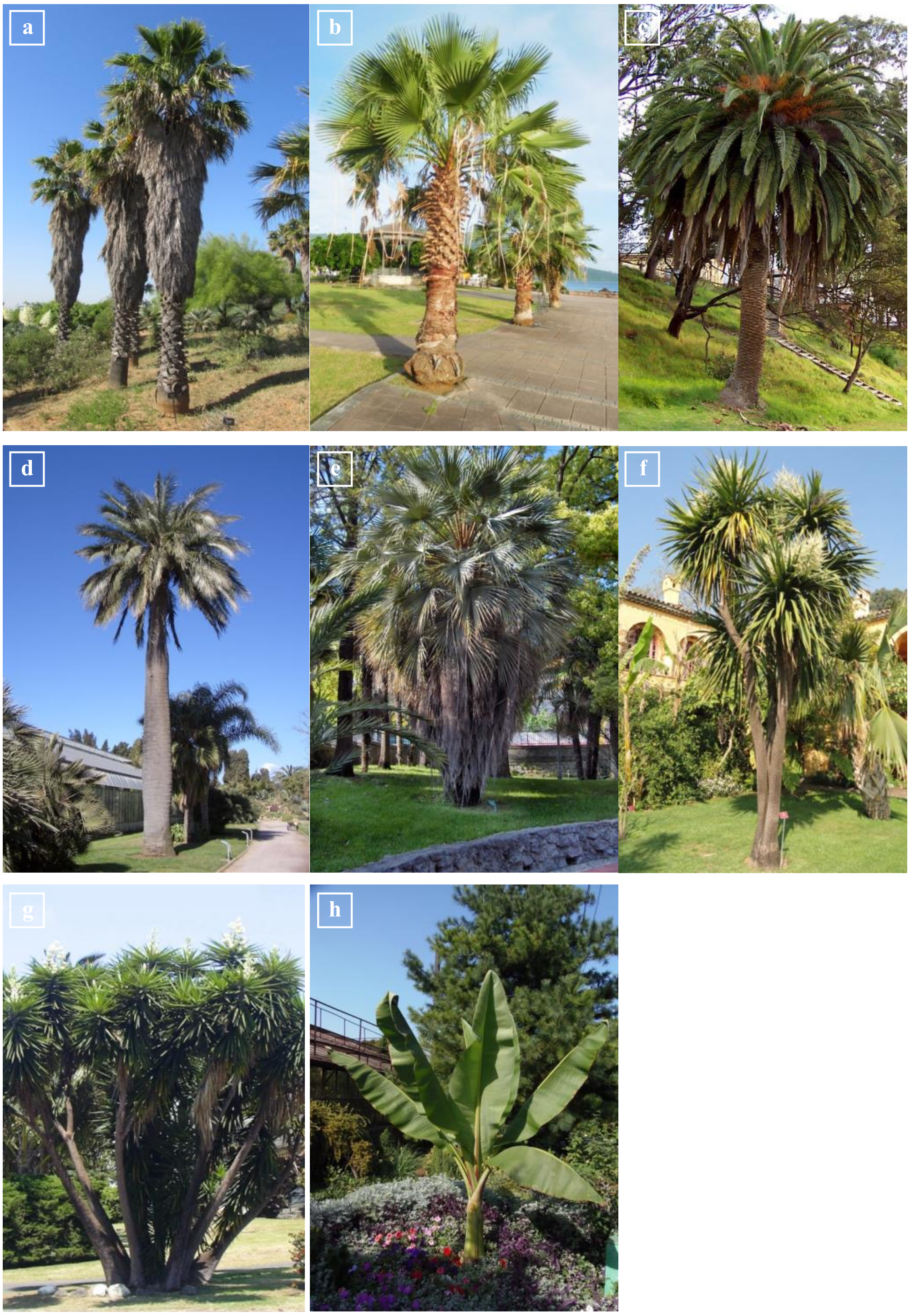
a
b
c
d
e
f
g
h

Im Folgenden wird eine Reihe von Aussagen zur Hanfpalme präsentiert. Sind Sie mit diesen Aussagen einverstanden?

41. Die Bevölkerung ist ausreichend über das Thema invasive Arten informiert.
    1/ Trifft zu
    2/ Trifft eher zu
    3/ Weder noch
    4/ Trifft eher nicht zu
    5/ Trifft nicht zu

42. Bundes- und Kantonsbehörden müssen mehr in Managementstrategien für invasive Neophyten investieren, zu denen auch die Hanfpalme gehört.
    1/ Trifft zu
    2/ Trifft eher zu
    3/ Weder noch
    4/ Trifft eher nicht zu
    5/ Trifft nicht zu

43. Es ist richtig, einzugreifen, um die Ausbreitung von die Hanfpalme außerhalb von Gärten und öffentlichen Parks zu verhindern.
    1/ Trifft zu
    2/ Trifft eher zu
    3/ Weder noch
    4/ Trifft eher nicht zu
    5/ Trifft nicht zu

44. Wir stellen nun eine Reihe von Maßnahmen vor, die die Verwaltung der Hanfpalme betreffen. Bitte geben Sie an, inwieweit Sie diese Optionen für angemessen halten.

	Unangemessen				Angemessen
	1	2	3	4	5
1/ Überwachung der Verbreitung					
2/ Sensibilisierungskampagnen für ein angemessenes Management					
3/ Tilgungmassnahmen in natürlicher Umwelt					
4/ Verbot an allen öffentlichen Orten					
5/ Verbot jeglicher Handelsart					
6/ Verbot an allen privaten Orten					

Zum Abschluss des Fragebogens bitten wir Sie um einige persönliche Angaben.

45. Gehören Sie zu einem Verband/einer Organisation, die sich mit Umweltschutz befasst?
    1/ Ja
    2/ Nein **(→ to question 47)**

46. Welcher Verband/welche Organisation ist es? …………

47. Letzter erhaltet Schulabschluss
    1/ Keine
    2/ Primar – Sekundarschule
    3/ Berufliche Grundbildung (Lehre), Fachmittelschule (FMS)
    4/ Gymnasiale Maturitätschulen, Gymnasium
    5/ Höhere Fachschulen (HF)
    6/ Fachhochschulen (FH), Pädagogische Hochschulen (PH)
    7/ Universitäre Hochschulen (UH, inkl. ETH)

48. Derzeitiger Beruf
    1/ In Ausbildung
    2/ Primärer Sektor
    3/ Sekundärer Sektor
    4/ Tertiärer Sektor
    5/ Hausfrau/-mann
    6/ Im Ruhestand
    7/ Arbeitslos

49. Ist oder war Ihr Beruf mit der Umwelt verbunden?
    1/ Ja
    2/ Nein **(→ to ending text)**

50. Welcher Beruf ist das? …………

Vielen Dank, dass Sie sich die Zeit genommen haben, den Fragebogen auszufüllen. Ihre Antworten werden uns eine grosse Hilfe sein. Wir erinnern Sie daran, dass Ihre Informationen vertraulich behandelt werden. Falls Sie es wünschen, können Sie unten einen Kommentar hinterlassen.

French version

Le but de ce questionnaire est d'investiguer la perception et les connaissances que la population suisse a au regard du palmier chanvre (*Trachycarpus fortunei*), visible dans l'image ci-dessous.

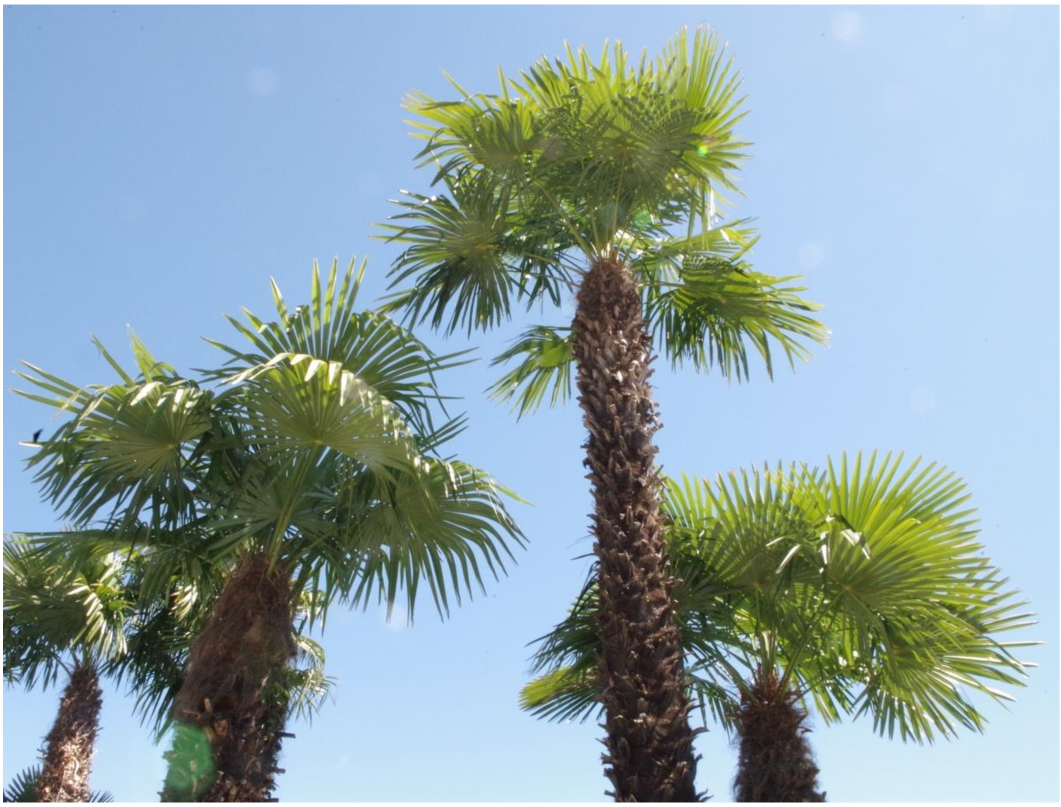

La compilation du questionnaire est volontaire et anonyme, les informations personnelles qui y sont contenues sont purement à des fins statistiques. Durée : 15 minutes.
Le questionnaire fait partie d'un Mémoire de Master à l'École Polytechnique Fédérale de Zürich (EPFZ) en collaboration avec l'Institut fédéral de recherches sur la forêt, la neige et le paysage (WSL, siège de Cadenazzo).

WSL

ETH zürich

1. Canton d'origine
   Argovie (AG), Appenzell Rhodes-Intérieures (AI), Appenzell Rhodes-Extérieures (AR), Berne (BE), Bâle-Campagne (BL), Bâle-Ville (BS), Fribourg (FR), Genève (GE), Glaris (GL), Grisons (GR), Jura (JU), Lucerne (LU), Neuchâtel (NE), Nidwald (NW), Obwald (OW), Saint-Gall (SG), Schaffhouse (SH), Soleure (SO), Schwytz (SZ), Thurgovie (TG), Tessin (TI), Uri (UR), Vaud (VD), Valais (VS), Zoug (ZG), Zurich (ZH)

2. Canton de résidence permanente
   Argovie (AG), Appenzell Rhodes-Intérieures (AI), Appenzell Rhodes-Extérieures (AR), Berne (BE), Bâle-Campagne (BL), Bâle-Ville (BS), Fribourg (FR), Genève (GE), Glaris (GL), Grisons (GR), Jura (JU), Lucerne (LU), Neuchâtel (NE), Nidwald (NW), Obwald (OW), Saint-Gall (SG), Schaffhouse (SH), Soleure (SO), Schwytz (SZ), Thurgovie (TG), Tessin (TI), Uri (UR), Vaud (VD), Valais (VS), Zoug (ZG), Zurich (ZH)

3. Où vivez vous?
   1/ Espace urbain : centre
   2/ Espace urbain : périphérie
   3/ Espace rural ou région de montagne

4. Langue maternelle
   1/ Italien
   2/ Français
   3/ Allemand
   4/ Romanche
   5/ Autre …………

5. Année de naissance …………

2002	2001	2000	1999	1998	1997	1996	1995	1994	1993
1992	1991	1990	1989	1988	1987	1986	1985	1984	1983
1982	1981	1980	1979	1978	1977	1976	1975	1974	1973
1972	1971	1970	1969	1968	1967	1966	1965	1964	1963
1962	1961	1960	1959	1958	1957	1956	1955	1954	1953
1952	1951	1950	1949	1948	1947	1946	1945	1944	1943
1942	1941	1940	1939	1938	1937	1936	1935	1934	1933
1932	1931	1930	1929	1928	1927	1926	1925	1924	1923
1922	1921	1920							

6. Genre
   1/ Femme
   2/ Homme
   3/ Autre …………

7. D'où pensez-vous que le palmier chanvre est originaire?
   1/ Afrique
   2/ Asie
   3/ Europe

4/ Amérique du Nord
5/ Océanie
6/ Amérique du Sud

8. En Suisse, avez-vous déjà observé ce palmier?
1/ Oui
2/ Non **(→ to question 11)**

9. Dans quels Cantons suisses l'avez-vous déjà observé?
Argovie (AG), Appenzell Rhodes-Intérieures (AI), Appenzell Rhodes-Extérieures (AR), Berne (BE), Bâle-Campagne (BL), Bâle-Ville (BS), Fribourg (FR), Genève (GE), Glaris (GL), Grisons (GR), Jura (JU), Lucerne (LU), Neuchâtel (NE), Nidwald (NW), Obwald (OW), Saint-Gall (SG), Schaffhouse (SH), Soleure (SO), Schwytz (SZ), Thurgovie (TG), Tessin (TI), Uri (UR), Vaud (VD), Valais (VS), Zoug (ZG), Zurich (ZH)

10. Veuillez indiquer dans quel environnement/lieu vous avez déjà vu le palmier chanvre.
1/ Jardin privé
2/ Jardin public (parc)
3/ Bord du lac
4/ Bord de rivière
5/ Marge d'une forêt
6/ À l'intérieur d'une forêt
7/ À coté d’une route
8/ Magasin
9/ Autre

11. Souhaiteriez-vous voir le palmier chanvre dans les environnements/lieux listés ?

	Oui	Plutôt oui	Ni oui ni non	Plutôt pas	Non
	1	2	3	4	5
1/ Jardin privé					
2/ Jardin public (parc)					
3/ Bord du lac					
4/ Bord de rivière					
5/ Marge d'une forêt					
6/ À l'intérieur d'une forêt					
7/ À coté d’une route					
8/ Magasin					

12. Y a-t-il un ou plusieurs palmiers chanvre sur les propriétés adjacentes à votre maison primaire?
1/ Oui
2/ Non **(→ to question 14)**
3/ Je ne sais pas **(→ to question 14)**

13. Combien de palmiers chanvre y a-t-il sur les propriétés adjacentes à votre maison primaire ?
    1/ Peu (1-3)
    2/ Quelques - uns (4-10)
    3/ Beaucoup (>10)

14. Dans le cas où vous avez un jardin privé, ou si votre immeuble en a un, qui est principalement chargé de son entretien?
    1/ Je n'ai pas de jardin privé / L'immeuble n'a pas de jardin privé **(→ to question 19)**
    2/ Moi personnellement
    3/ Un membre de ma famille
    4/ Une personne chargée à titre non-professionnel
    5/ Un professionnel

15. Y a-t-il des palmiers chanvre dans votre jardin ou dans votre immeuble?
    1/ Oui
    2/ Non, mais j'en voudrais **(→ to question 19)**
    3/ Non, et je n'en voudrais pas **(→ to question 19)**
    4/ Je ne sais pas **(→ to question 19)**

16. Combien de palmiers chanvre y a-t-il dans votre jardin ou dans votre immeuble?
    1/ Peu (1-3)
    2/ Quelques - uns (4-10)
    3/ Beaucoup (>10)

17. La décision de planter ces palmiers chanvre a-t-elle été prise par vous-même ou un autre membre de votre famille?
    1/ Oui
    2/ Non **(→ to question 19)**

18. Où les a-t-il obtenues? …………

19. Êtes-vous ou votre famille propriétaires d'une résidence secondaire en Suisse?
    1/ Oui
    2/ Non **(→ to question 24)**

20. Dans quel Canton? Argovie (AG), Appenzell Rhodes-Intérieures (AI), Appenzell Rhodes-Extérieures (AR), Berne (BE), Bâle-Campagne (BL), Bâle-Ville (BS), Fribourg (FR), Genève (GE), Glaris (GL), Grisons (GR), Jura (JU), Lucerne (LU), Neuchâtel (NE), Nidwald (NW), Obwald (OW), Saint-Gall (SG), Schaffhouse (SH), Soleure (SO), Schwytz (SZ), Thurgovie (TG), Tessin (TI), Uri (UR), Vaud (VD), Valais (VS), Zoug (ZG), Zurich (ZH)

21. Si la résidence secondaire a un jardin, qui est principalement chargé de son entretien?
    1/ Il n'y a pas de jardin **(→ to question 24)**
    2/ Moi personnellement
    3/ Un membre de ma famille
    4/ Une personne chargée à titre non-professionnel

5/ Un professionnel

22. Le jardin en question possède-t-il un ou plusieurs palmiers chanvre?
    1/ Oui
    2/ Non, mais j'en voudrais **(→ to question 24)**
    3/ Non, et je n'en voudrais pas **(→ to question 24)**

23. Combien de palmiers chanvre y a-t-il dans ce jardin?
    1/ Peu (1-3)
    2/ Quelques - uns (4-10)
    3/ Beaucoup (>10)

24. Savez-vous que les spécimens de palmiers chanvre ne produisent qu'un seul type de fleur: mâle ou femelle? Ils ne produisent donc pas tous des graines.
    1/ Oui
    2/ Non **(→ to question 26)**

25. Pouvez-vous distinguer les inflorescences?
    1/ Oui
    2/ Non

26. Prenez un moment pour penser au palmier chanvre. Quel est le premier mot qui vous vient spontanément à l'esprit?
    …………

27. Appréciez-vous le palmier chanvre?
    1/ Oui
    2/ Plutôt oui
    3/ Ni oui ni non
    4/ Plutôt pas
    5/ Non
    Justification du choix …………

28. Souhaiteriez-vous qu'il y ait davantage de palmiers chanvre dans les lieux publics?
    1/ Oui
    2/ Plutôt oui
    3/ Ni oui ni non
    4/ Plutôt pas
    5/ Non

29. A votre avis, la présence du palmier chanvre dans les lieux publics et privés influence de quelconques façons les milieux naturels environnants?
    1/ Oui
    2/ Non
    3/ Je ne sais pas

30. En général, comment évaluez-vous l'impact du palmier chanvre sur les milieux naturels suisses?

1/ Très négatif
2/ Négatif
3/ Ni négatif ni positif
4/ Positif
5/ Très positif
6/ Je n'ai pas d'opinion à ce sujet

Nous présentons ci-dessous une série de déclarations concernant le palmier chanvre. Êtes-vous d'accord avec ces déclarations?

31. Le palmier chanvre est une espèce qui représente la région des lacs insubriques, dont les lacs Maggiore (Locarno), Ceresio (Lugano) et Lario (Como) font partie.
    1/ Tout à fait d'accord
    2/ Plutôt d'accord
    3/ Ni d'accord ni en désaccord
    4/ Plutôt en désaccord
    5/ Tout à fait en désaccord

32. Le palmier chanvre est un symbole qui représente le Tessin en Suisse.
    1/ Tout à fait d'accord
    2/ Plutôt d'accord
    3/ Ni d'accord ni en désaccord
    4/ Plutôt en désaccord
    5/ Tout à fait en désaccord

33. Au Tessin, le palmier chanvre est une attraction pour les touristes.
    1/ Tout à fait d'accord
    2/ Plutôt d'accord
    3/ Ni d'accord ni en désaccord
    4/ Plutôt en désaccord
    5/ Tout à fait en désaccord

34. Pour chacune des paires de photographies suivantes, indiquez celle que vous préférez.

Edge of a forest

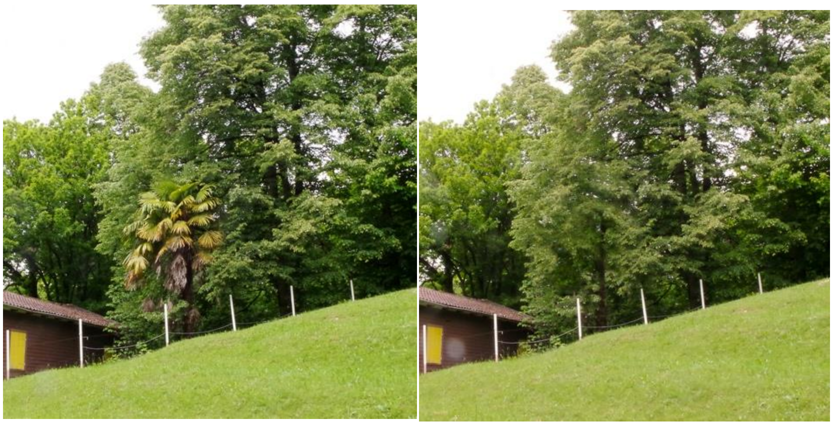

Longlake

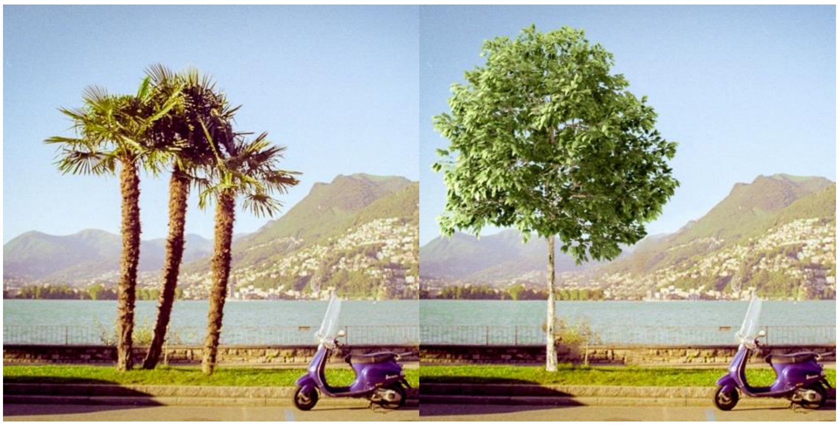

Courtyard

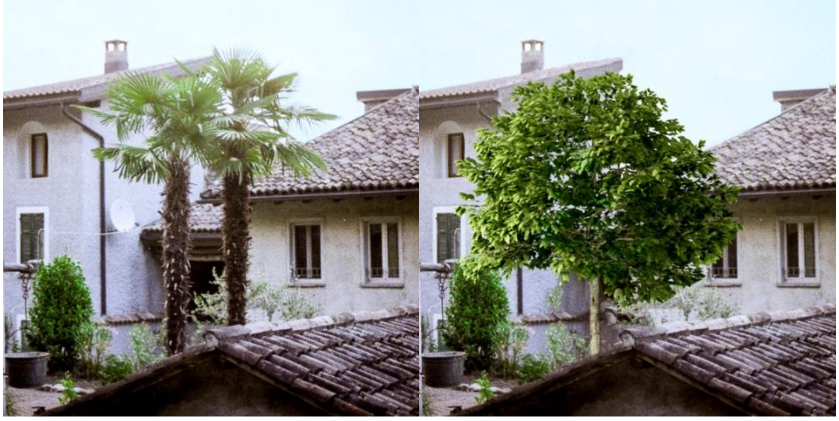

35. Avez-vous déjà entendu le terme «néophyte envahissante»?
    1/ Oui, et j'en connais le sens
    2/ Oui, mais je n'en connais pas le sens
    3/ Non [→ to info paragraph]

36. Dans quel contexte avez-vous entendu le terme «néophyte envahissante»?
    1/ À la télévision
    2/ À la radio
    3/ Dans un journal
    4/ Dans un magazine
    5/ Sur internet
    6/ Une connaissance m'en a parlé
    7/ Au travail / formation
    8/ Autre …………

Le palmier chanvre est une espèce originaire d'Asie de l'Est et introduite en Europe par l'homme après les années 1500 (d'où le terme **néophyte**). Cette espèce produit de nombreuses graines qui germent facilement et sont disséminées dans l'environnement par des oiseaux ou par une gestion inadéquate des déchets végétaux. Pour cette raison, le palmier chanvre est devenu **envahissant** et se répand dans la forêt à partir des jardins publics et privés, en s'adaptant aux différentes conditions. Il est sur la **liste noire** Infoflora des néophytes envahissantes.

Des recherches scientifiques sont actuellement en cours pour quantifier les **effets** du palmier chanvre en milieu forestier, où il entre en concurrence avec la végétation locale. En effet, il pourrait en empêcher le renouvellement et réduire à long terme les importantes fonctions des forêts, telles que la protection contre les risques naturels, la biodiversité, l'approvisionnement en bois et la fonction de récréation pour la population.

L'**élimination** annuelle des inflorescences femelles du palmier chanvre serait une mesure efficace pour empêcher la propagation de l'espèce dans les milieux naturels.

Pour la conservation de la nature et la biodiversité des forêts, la Confédération dépense chaque année plusieurs dizaines de millions de francs. Le **budget** prévu à cet effet a **doublé** ces dernières années pour atteindre 77 millions de CHF en 2020. Les cantons contribuent au moins à parts égales.

Nous allons maintenant répéter deux questions posées précédemment.

37. Souhaiteriez-vous avoir un ou plusieurs palmiers chanvre dans votre jardin ou dans le jardin de votre immeuble?
    1/ Oui
    2/ Plutôt oui
    3/ Ni oui ni non
    4/ Plutôt pas
    5/ Non
    6/ Je n'ai pas de jardin

38. Souhaiteriez-vous qu'il y ait davantage de palmiers chanvre dans les lieux publics?
    1/ Oui
    2/ Plutôt oui
    3/ Ni oui ni non
    4/ Plutôt pas
    5/ Non

39. Seriez-vous prêt à prendre des mesures pour enlever les inflorescences féminines du palmier chanvre sur votre propriété?
    1/ Oui, je le ferais moi-même
    2/ Oui, j'engagerais quelqu'un pour le faire
    3/ J'aimerais bien, mais je n'ai pas la possibilité de le faire
    4/ Non
    5/ Je ne possède pas de palmier chanvre

40. Voici à nouveau une photo du palmier chanvre.

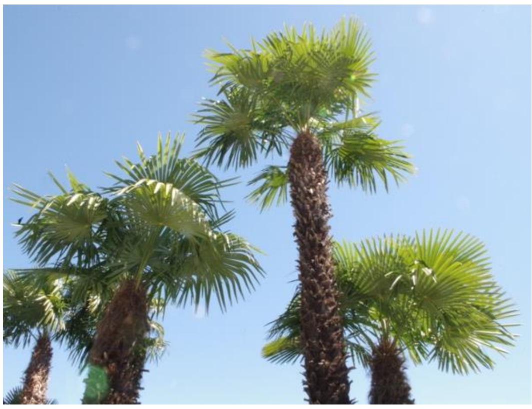

Pour chacune des espèces exotiques représentées sur les images suivantes, indiquez si vous aimeriez la voir dans des lieux publics et privés à la place du palmier chanvre. Les espèces proposées ne sont pas considérées comme envahissantes en Suisse.

Vous devez répondre rapidement. En effet, vous n'aurez que 8 secondes par image. Pour répondre OUI, appuyez sur la touche 1 (un) de votre clavier. Pour répondre NON, appuyez sur la touche 0 (zéro).

Pour tester votre clavier, appuyez maintenant sur OUI
Pour tester votre clavier, appuyez maintenant sur NON

Veuillez s'il vous plaît répondre à toutes les questions avant de changer de page, sinon vos réponses ne seront pas enregistrées.

Aimeriez-vous voir cette espèce au lieu du palmier chanvre?
Veuillez vous rappeler le choix : 1 = OUI / 0 = NON

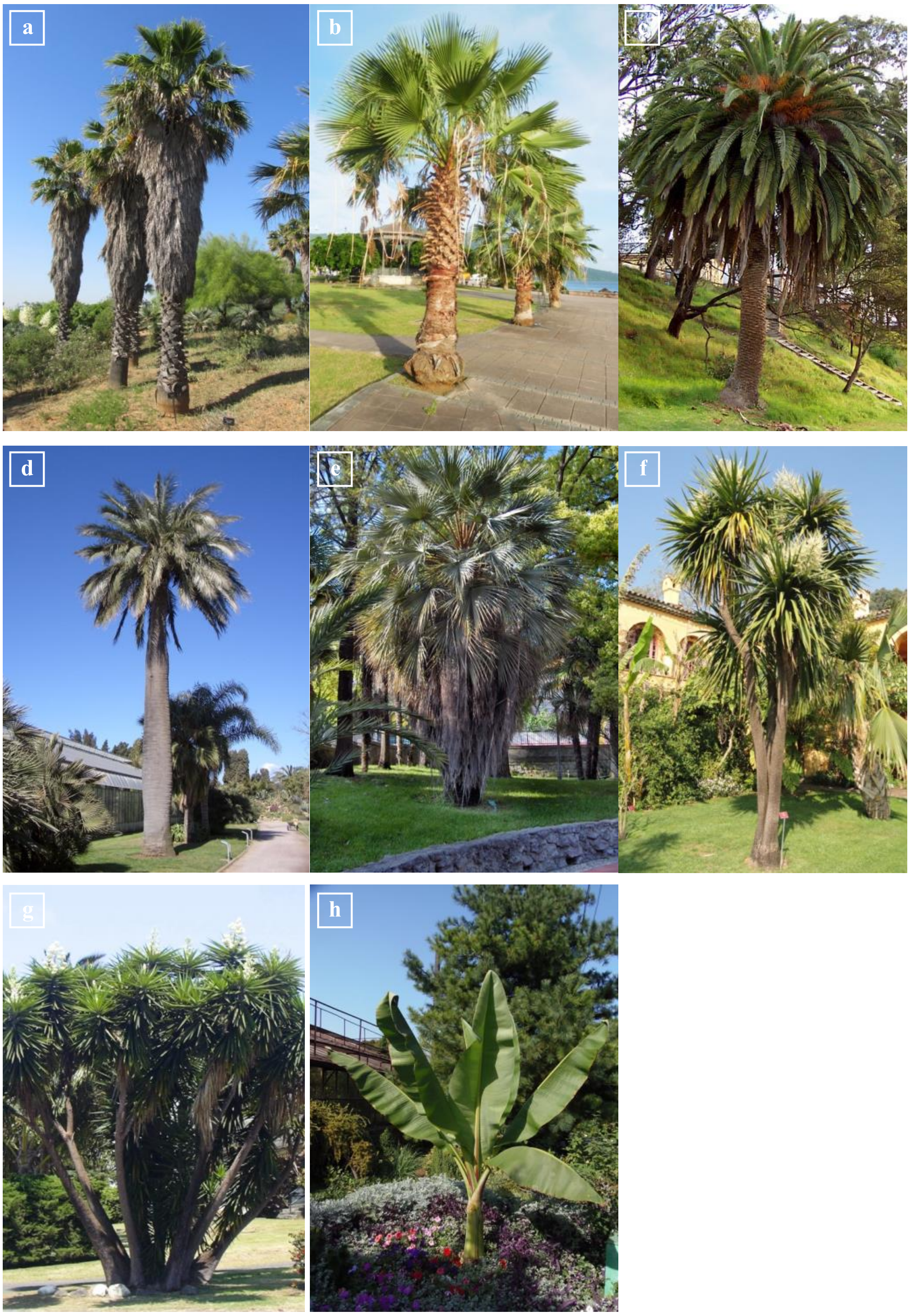
a
b
d
e
f
h

Ci-après sont présentées une série de déclarations concernant le palmier chanvre. Êtes-vous d'accord avec ces déclarations?

41. La population est suffisamment informée sur le thème des espèces envahissantes.
    1/ Tout à fait d'accord
    2/ Plutôt d'accord
    3/ Ni d'accord ni en désaccord
    4/ Plutôt en désaccord
    5/ Tout à fait en désaccord

42. Les autorités fédérales et cantonales doivent investir davantage dans les stratégies de gestion des néophytes envahissantes, dont le palmier chanvre fait partie.
    1/ Tout à fait d'accord
    2/ Plutôt d'accord
    3/ Ni d'accord ni en désaccord
    4/ Plutôt en désaccord
    5/ Tout à fait en désaccord

43. Il est juste d'intervenir pour empêcher la propagation du palmier chanvre en dehors des jardins et des parcs publics.
    1/ Tout à fait d'accord
    2/ Plutôt d'accord
    3/ Ni d'accord ni en désaccord
    4/ Plutôt en désaccord
    5/ Tout à fait en désaccord

44. Nous présentons maintenant une série de mesures concernant la gestion du palmier chanvre. Veuillez indiquer dans quelle mesure vous estimez que ces options sont appropriées.

	Pas appropriée				Appropriée
	1	2	3	4	5
1/ Surveillance de la diffusion					
2/ Campagnes de sensibilisation en faveur d'une gestion adéquate					
3/ Interventions d'éradication en milieu naturel					
4/ Interdiction dans tous les lieux publics					
5/ Interdiction de tout commerce					
6/ Interdiction dans tous les lieux privés					

Pour conclure le questionnaire on vous demande quelque information personnelle.

45. Faites-vous partie d'une association / organisation qui s’investit dans la protection de l'environnement?
    1/ Oui
    2/ Non **(→ to question 47)**

46. De quelle association/organisation s'agit-il? …………

47. Dernier diplôme obtenu
    1/ Aucun
    2/ École primaire et secondaire
    3/ Formation professionnelle initiale, écoles de culture générale
    4/ École de maturité gymnasiale
    5/ Écoles supérieures (ES)
    6/ Hautes écoles spécialisées (HES), hautes écoles pédagogiques (HEP)
    7/ Hautes écoles universitaires (HEU, inclus EPF)

48. Profession actuelle
    1/ En formation
    2/ Secteur primaire
    3/ Secteur secondaire
    4/ Secteur tertiaire
    5/ Homme / femme au foyer
    6/ Retraité
    7/ Sans emploi

49. Votre profession est-elle ou a-t-elle été liée à l'environnement?
    1/ Oui
    2/ Non **(→ to ending text)**

50. De quelle profession s'agit-il? …………

Merci beaucoup d'avoir pris le temps de remplir le questionnaire, vos réponses nous seront d'une grande utilité. Nous vous rappelons que vos informations seront traitées confidentiellement. Si vous le souhaitez, n’hésitez pas à laisser un commentaire ci-dessous.
