# Supplementary material for: Iconic but Invasive: The Public Perception of the Chinese Windmill Palm (Trachycarpus fortunei) in Switzerland
Source: Environ Manage. 2022 Apr 26;70(4):618–32. doi: 10.1007/s00267-022-01646-3 (PMC9439986; doi:10.1007/s00267-022-01646-3)
Supplement: Supplementary file 3 — Supplementary Materials S3 [file 267_2022_1646_MOESM3_ESM.pdf]

Supplementary Materials S3

Alternative non-invasive species proposed to replace *T. fortunei* in Switzerland

Taxon, family, morphological group, and amount of dead leaves of the species proposed to potentially replace the Chinese windmill palm in Switzerland. In addition, the sources of the images of the alternative species used in the questionnaire are added.

Main characteristics of the alternative species proposed to potentially replace the Chinese windmill palm.

**Taxon**	**Family**	**Morphological group**	**Amount of dead leaves of the individual in the picture**
Brahea armata	Arecaceae	Fan palms	few
Washingtonia robusta	Arecaceae	Fan palms	many
Washingtonia robusta	Arecaceae	Fan palms	no
Jubaea chilensis	Arecaceae	Feather palms	no
Phoenix canariensis	Arecaceae	Feather palms	few
Cordyline australis	Asparagaceae	Yucca-like plants	no
Yucca gigantea	Asparagaceae	Yucca-like plants	few
Ensete ventricosum	Musaceae	Banana plants	no

Sources of the images representing the alternative species (accessed 25 June 2020):

- *Washingtonia robusta* (1)
  https://commons.wikimedia.org/wiki/File:Jardi_botanic_de_barcelona_washingtonia_filifera.jpg
  Jvhertum / CC BY-SA (https://creativecommons.org/licenses/by-sa/3.0)

- *Washingtonia robusta* (2)
  https://commons.wikimedia.org/wiki/File:Washingtonia_robusta_(Arecaceae)_01.jpg
  Filo gèn' / CC BY-SA (https://creativecommons.org/licenses/by-sa/4.0)

- *Phoenix canariensis*
  https://commons.wikimedia.org/w/index.php?curid=303279
  Photo cropped from en:Image:Park.jpg by Fir0002, edit by MPF
  CC BY-SA 3.0, (https://creativecommons.org/licenses/by-sa/3.0)

- *Jubaea chilensis*
  https://commons.wikimedia.org/w/index.php?curid=954010
  Photo taken by SteffenMP (de:Benutzer:SteffenMP) - first published at the German Wikipedia project as de:Bild:Jubaea chilensis Hyères gross.jpg by de:Benutzer:SteffenMP / CC BY-SA 3.0, (https://creativecommons.org/licenses/by-sa/3.0)

- *Brahea armata*
  https://commons.wikimedia.org/wiki/File:Brahea_armata001.jpg
  Georges Jansoone (JoJan) / CC BY (https://creativecommons.org/licenses/by/3.0)

- *Cordyline australis*
  https://commons.wikimedia.org/wiki/File:Cordyline_australis_-_Val_Rahmeh_-_DSC04255.JPG
  Daderot / Public domain

- *Yucca gigantea*
  https://commons.wikimedia.org/wiki/File:Yucca_gloriosa-01.jpg
  http://www.hear.org/starr/hiplants/images/, CC BY 4.0 (https://creativecommons.org/licenses/by/4.0), via Wikimedia Commons

- *Ensete ventricosum*
  https://commons.wikimedia.org/wiki/File:Musa_basjoo_-_%D0%91%D0%B0%D0%BD%D0%B0%D0%BD_%D1%8F%D0%BF%D0%BE%D0%BD%D1%81%D1%8C%D0%BA%D0%B8%D0%B9_-_%D0%91%D0%B0%D0%BD%D0%B0%D0%BD_%D1%8F%D0%BF%D0%BE%D0%BD%D1%81%D0%BA%D0%B8%D0%B9.jpg
  Одинець Сергій / CC BY (https://creativecommons.org/licenses/by/4.0)
