# Supplementary material for: Iconic but Invasive: The Public Perception of the Chinese Windmill Palm (Trachycarpus fortunei) in Switzerland
Source: Environ Manage. 2022 Apr 26;70(4):618–32. doi: 10.1007/s00267-022-01646-3 (PMC9439986; doi:10.1007/s00267-022-01646-3)
Supplement: Supplementary file 4 — Supplementary Materials S4 [file 267_2022_1646_MOESM4_ESM.pdf]

Supplementary Materials S4

Free word association

Rank, frequency (i.e., count), and respective English category of the answers given to the free word association question according to the native language.

Participants had to answer the following question: take a moment to think about the windmill palm. What is the first word that spontaneously comes to your mind?

A total of 46 English categories have been created.

Rank	Count	Category	Italian native speakers	French native speakers	German native speakers
1	448	Drug	Droga	Drogue, fumer, fumée, chanvre, fumette, cannabis, marijuana, défonce, produit toxicologique, Marie-Jeanne, joint, medicament, jumbo	Drogenhandel, cannabis, Hanf, Droge, Medizin, Joint, Sucht, Suchtmittel, Hanfplanze, Hanfprodukte, Kiffen, Rauschmittel, Rauschgift, Hashish, rauchen, CBD, Schmerzlinderung, legal, Heilpflanze, illegal, Marijuana, Medikament, Aufputschmittel, Betäubungsmittel, Opiat, Grass, Schmerzlindernd, Missbrauchen, Arznei, Scmerztherapie, verboten, Nutzpflanzen, süchtig, Hanfkonsum, Heilmittel, Suchtgefähr, Drogenabhängig, Konsum, Heilkraut
2	196	Holidays	Rilassamento, relax, rilassante, vacanze, spiaggia	Vacances, voyage, plage, détente, relax	Ferien, Feriengefühl, Strand, Urlaub, Ferienstimmung, Ferienfeeling, Urlaubsfeeling, Entspannung, relax
3	170	Warm	Sole, estate, caldo, soleggiante, solare, estivo	Chaleur, été, soleil, chaud, chaleureux, pays chaud	Sonne, Hitze, Sommer, Wärme, Sommergefühle, schön Wetter, warmes Klima
4	151	Exotic	Esotica, alloctona, intrusa, aborigena, luogo esotico, clima extraeuropeo, specie aliena	Exotique, exotisme, pays exotique, plante exotique, plante importée	Exotisch, Fremd, Ausländische Pflanze, nicht einheimisch, fremdländisch, nicht Schweizerisch, fremdartig, zugewandert, artfremd
5	115	Beautiful	Bella, bellezza, carina, cool, spettacolare, splendida, meravigliosa	Très jolie, beau, magnifique, joli, beauté, superbe, grâce, bel arbre, sympa	Schöne, wunderschön, hübsch, sehr schön, nett, schmücke Pflanze, ansprechend, graziös, Schönheit
6	112	Others	Elastica, niente, turismo, novità, prolifica, imperialista, amaca, vento, esportazione, nonni, bosco, fuoco, omologazione, permanente, protezione, incendiabile, casa, infanzia, soldi, ventaglio	Rustique, déplacé, aucune idée, je ne sais pas, desert, popular, rien, oui, ?, coupant, évader, prix	Weiss nicht, Zeit, Bewusstseinsveränderung, Harz, nichts, keine, Hobbygärtner, weiter, nicht nötig, Witz, Betäubung, Zimmerpflanze, mehr, flash, neu, besser, rarität, keine Ahnung, neutral, Aufwand der Pflege, ?, spontan, egal, Tee, Mückenschutz, überraschend, Kopf, hertellung Produkte, nein
7	88	Sea	Mare, oceano	Bord de mer, océan, mer	Meer, Südsee
8	68	Tropics	Caraibi, tropici, tropicale	Caraïbe, tropical, tropiques	Tropisch, tropische, Tropen, sudtropische
9	58	South	Sud	Sud, méridional	Süden, südliche Pflanze, südliches Klima, Südeuropa, Südländisch, südlicher Charme, südlich
10	53	Invasive	Invasiva, infestante	Invasive, invasif, envaissant	invasive Pflanze, invasiv
11	45	Palm	Palma, Yucca, palma nana	Palmier, palmier de Chusan	Palme, Chinesische Hanfpalme, Fächerpalme, Palmenart, Palmenartiger Baum
12	36	Luck	Fortuna, benaugurante	Chance	
13	32	NULL			
14	31	Height	Altezza, alta, quota	Grand	Gross
15	29	Ticino	Ticino, palma ticinese	Tessin	Tessin, tessiner Palme
16	28	Fruit	Frutta, cocco, semi, bacche, banana, datteri	Coco, dattes	Beeren, Cocco, Früchte, Vogelfutter, Ananas
17	26	Positive	Allegria, tranquillità, meraviglia, serenità, piacevole, prosperità, benessere, vivace	Sympa, bien être, calme, calmant, chic, liberté, douceur	Ruhe, alles gut, beruhigung, beruhigend, Freiheit, nützlich, genial, Entspannung
18	25	Unknown	Sconosciuta	Inconnu, connais pas	Unnötig, kenne nicht, unbekannt
19	24	Color	Verde, giallo	Vert	Grün
20	23	Negative	Brutta, inutile, fuoriluogo, ingombrante, superflua, parassita, schifosa, nociva	Inutile, rien à faire chez nous	Kritisch, unsitt, brauch nicht, passt nicht, will nicht
21	21	Ornamental	Decorativa, giardino, ornamentale	Ornamental, décoratif	Dekorativ, Deko, Zierpflanze, Zierbaum
22	20	Fiber		Corde, cordages, fibre, sac de jutte, textile, tissu	Faser, Seil, Spinnstoff, Papierhersellung, Schnur
23	19	Leaves	Foglie, fronde	Feuilles	Palmwedel, Blätter, Palmenblatt
24	16	Environment	Natura, clima mite, clima	Nature	Klimaerwärmung, Umwelt, Klimawandel, Artenschutz, Natur, Klimaverschiebung
25	14	Luxury	Maestosa, eleganza, lussureggiante	Elégance, élégant, luxuriant, majesteux	Elegant, majestàtisch
26	12	Tree	Pianta	Arbre, plante	Baumart, Baum, Pflanze
27	11	Appearance	Aspetto, capelli, imponente, rigogliosa, particolare, peluria, punte	Puissant, robuste	
28	11	Mediterranean		Méditéranée, méditerranéen	Mittelmeer, Italien, Mediterran, Israel
29	10	Shadow	Ombra	Ombre	Schattenspender, Schatten
30	10	Island	Isola	Île	Insel
31	10	Asia	Giappone, oriente, Cina, Asia	Tibet	Asiatisch, Japan, China
32	10	Resistant	Coriacea, resistente, forza	Résistant	Braucht wenig Wasser
33	8	Oil	Olio	Huile, huile de palme	Palmöl, öl
34	8	Health			Gesund, Gesundheit
35	8	Smell			Duft, Geruch
36	7	Oasis	Oasi	Oasis	
37	7	Neophyte	Neofita		Neophyt
38	6	Africa	Africa	Afrique	Afrika
39	6	America	California, Los Angeles, Hawaii, Miami beach, Florida, America latina		
40	6	Interesting	Interessante	Intéressant	Interessant
41	5	Flowers	Fiori		Blüten
42	4	Big	Grandezza, enorme	Géant, grandeur	
43	4	Common	Onnipresente	Dominant	Gewöhnliche
44	4	Fresh	Fresca, fresco, freschezza		
45	4	Original		Original, originalité	
46	3	Lake	Lago		See
