# Supplementary material for: Iconic but Invasive: The Public Perception of the Chinese Windmill Palm (Trachycarpus fortunei) in Switzerland
Source: Environ Manage. 2022 Apr 26;70(4):618–32. doi: 10.1007/s00267-022-01646-3 (PMC9439986; doi:10.1007/s00267-022-01646-3)
Supplement: Supplementary file 5 — Supplementary Materials S5 [file 267_2022_1646_MOESM5_ESM.pdf]

Supplementary Materials S5

Statistical tests

Strength of associations among variables using the non-parametric Kruskall-Wallis test and the Wilcoxon rank sum test for the pairwise comparisons.

Statistical analysis have been carried out using RStudio (version 1.3.1093, R Core Team 2021).

Page 1	Perception questions
Page 9	Preference questions
Page 47	Knowledge (i.e., information level) questions
Page 60	Acceptance of the management options

```r
source('import.r')

# PERCEPTION vs LANGUAGE ####
# Q27
kruskal.test(Q27~Q4_recode, data = d)
```

```
##
##  Kruskal-Wallis rank sum test
##
## data:  Q27 by Q4_recode
## Kruskal-Wallis chi-squared = 57.124, df = 3, p-value = 2.418e-12
```

```r
pairwise.wilcox.test(d$Q27, d$Q4_recode, p.adjust.method = "holm")
```

```
##
##  Pairwise comparisons using Wilcoxon rank sum test with continuity correction
##
## data:  d$Q27 and d$Q4_recode
##
##   1       2       3
## 2 0.00354 -       -
## 3 3.8e-12 0.00089 -
## 4 0.73230 0.73230 0.05318
##
## P value adjustment method: holm
```

```r
# Q31
kruskal.test(Q31~Q4_recode, data = d)
```

```
##
##  Kruskal-Wallis rank sum test
##
## data:  Q31 by Q4_recode
## Kruskal-Wallis chi-squared = 42.676, df = 3, p-value = 2.883e-09
```

```r
pairwise.wilcox.test(d$Q31,d$Q4_recode, p.adjust.method = "holm")
```

```
##
##  Pairwise comparisons using Wilcoxon rank sum test with continuity correction
##
## data:  d$Q31 and d$Q4_recode
##
##   1       2    3
## 2 3.6e-09 -    -
## 3 8.4e-06 0.11 -
## 4 0.12    0.40 0.90
##
## P value adjustment method: holm
```

```r
# Q32
kruskal.test(Q32~Q4_recode, data = d)
```

```
##
##  Kruskal-Wallis rank sum test
##
## data:  Q32 by Q4_recode
## Kruskal-Wallis chi-squared = 149.67, df = 3, p-value < 2.2e-16
```

```
pairwise.wilcox.test(d$Q32,d$Q4_recode, p.adjust.method = "holm")
```

```
##
##  Pairwise comparisons using Wilcoxon rank sum test with continuity correction
##
## data:  d$Q32 and d$Q4_recode
##
##   1       2       3
## 2 < 2e-16 -       -
## 3 < 2e-16 0.97255 -
## 4 0.00025 0.97255 0.97255
##
## P value adjustment method: holm
```

```
# Q33
kruskal.test(Q33~Q4_recode, data = d)
```

```
##
##  Kruskal-Wallis rank sum test
##
## data:  Q33 by Q4_recode
## Kruskal-Wallis chi-squared = 72.492, df = 3, p-value = 1.249e-15
```

```
pairwise.wilcox.test(d$Q33,d$Q4_recode, p.adjust.method = "holm")
```

```
##
##  Pairwise comparisons using Wilcoxon rank sum test with continuity correction
##
## data:  d$Q33 and d$Q4_recode
##
##   1       2       3
## 2 3.8e-09 -       -
## 3 4.0e-14 0.96291 -
## 4 0.00044 1.00000 1.00000
##
## P value adjustment method: holm
```

```
# PERCEPTION vs AGE ####
# Q27
kruskal.test(Q27~RECODE_AGE, data = d)
```

```
##
##  Kruskal-Wallis rank sum test
##
## data:  Q27 by RECODE_AGE
## Kruskal-Wallis chi-squared = 78.27, df = 4, p-value = 4.05e-16
```

```
pairwise.wilcox.test(d$Q27, d$RECODE_AGE, p.adjust.method = "holm")
```

```
##
##  Pairwise comparisons using Wilcoxon rank sum test with continuity correction
##
## data:  d$Q27 and d$RECODE_AGE
##
##   1      2      3      4
## 2 0.3606 -      -      -
## 3 0.1464 0.5222 -      -
```

```
## 4 1.6e-05 0.0038  0.0287  -
## 5 3.3e-13 1.4e-09 6.1e-08 0.0016
##
## P value adjustment method: holm
```

```
# Q31
kruskal.test(Q31~RECODE_AGE, data = d)
```

```
##
##  Kruskal-Wallis rank sum test
##
## data:  Q31 by RECODE_AGE
## Kruskal-Wallis chi-squared = 15.209, df = 4, p-value = 0.004286
```

```
pairwise.wilcox.test(d$Q31,d$RECODE_AGE, p.adjust.method = "holm")
```

```
##
##  Pairwise comparisons using Wilcoxon rank sum test with continuity correction
##
## data:  d$Q31 and d$RECODE_AGE
##
##   1      2      3      4
## 2 1.0000 -      -      -
## 3 1.0000 0.8854 -      -
## 4 0.3546 0.1217 1.0000 -
## 5 0.0344 0.0082 0.3546 1.0000
##
## P value adjustment method: holm
```

```
# Q32
kruskal.test(Q32~RECODE_AGE, data = d)
```

```
##
##  Kruskal-Wallis rank sum test
##
## data:  Q32 by RECODE_AGE
## Kruskal-Wallis chi-squared = 63.927, df = 4, p-value = 4.329e-13
```

```
pairwise.wilcox.test(d$Q32,d$RECODE_AGE, p.adjust.method = "holm")
```

```
##
##  Pairwise comparisons using Wilcoxon rank sum test with continuity correction
##
## data:  d$Q32 and d$RECODE_AGE
##
##   1       2       3      4
## 2 0.2569  -       -      -
## 3 0.0160  0.1726  -      -
## 4 6.2e-07 8.4e-05 0.0905 -
## 5 5.5e-10 1.2e-07 0.0015 0.1921
##
## P value adjustment method: holm
```

```
# Q33
kruskal.test(Q33~RECODE_AGE, data = d)
```

```
##
##  Kruskal-Wallis rank sum test
```

```
##
## data:  Q33 by RECODE_AGE
## Kruskal-Wallis chi-squared = 43.919, df = 4, p-value = 6.67e-09
```

```
pairwise.wilcox.test(d$Q33,d$RECODE_AGE, p.adjust.method = "holm")
```

```
##
##  Pairwise comparisons using Wilcoxon rank sum test with continuity correction
##
## data:  d$Q33 and d$RECODE_AGE
##
##   1       2       3       4
## 2 0.47069 -       -       -
## 3 0.09899 0.27890 -       -
## 4 0.00055 0.00500 0.27890 -
## 5 5.3e-07 4.4e-06 0.00500 0.16883
##
## P value adjustment method: holm
```

```
# PERCEPTION vs EDUCATION ####
# Q27
kruskal.test(Q27~Q47, data = d)
```

```
##
##  Kruskal-Wallis rank sum test
##
## data:  Q27 by Q47
## Kruskal-Wallis chi-squared = 27.577, df = 6, p-value = 0.0001129
```

```
pairwise.wilcox.test(d$Q27, d$Q47, p.adjust.method = "holm")
```

```
##
##  Pairwise comparisons using Wilcoxon rank sum test with continuity correction
##
## data:  d$Q27 and d$Q47
##
##   1      2      3      4       5      6
## 2 1.0000 -      -      -       -      -
## 3 1.0000 1.0000 -      -       -      -
## 4 1.0000 1.0000 0.0036 -       -      -
## 5 1.0000 1.0000 1.0000 0.0078  -      -
## 6 1.0000 0.6595 0.4384 1.6e-05 1.0000 -
## 7 1.0000 1.0000 1.0000 0.0824  1.0000 0.1002
##
## P value adjustment method: holm
```

```
# Q31
kruskal.test(Q31~Q47, data = d) # N.S.
```

```
##
##  Kruskal-Wallis rank sum test
##
## data:  Q31 by Q47
## Kruskal-Wallis chi-squared = 5.0137, df = 6, p-value = 0.5421
```

```
# Q32
kruskal.test(Q32~Q47, data = d)
```

```
##
##  Kruskal-Wallis rank sum test
##
## data:  Q32 by Q47
## Kruskal-Wallis chi-squared = 14.931, df = 6, p-value = 0.0208
```

```
pairwise.wilcox.test(d$Q32,d$Q47, p.adjust.method = "holm")
```

```
##
##  Pairwise comparisons using Wilcoxon rank sum test with continuity correction
##
## data:  d$Q32 and d$Q47
##
##   1     2     3     4     5     6
## 2 1.000 -     -     -     -     -
## 3 1.000 1.000 -     -     -     -
## 4 1.000 1.000 0.091 -     -     -
## 5 1.000 1.000 1.000 0.137 -     -
## 6 1.000 1.000 1.000 0.194 1.000 -
## 7 1.000 1.000 0.479 1.000 0.575 0.752
##
## P value adjustment method: holm
```

```
# Q33
kruskal.test(Q33~Q47, data = d)
```

```
##
##  Kruskal-Wallis rank sum test
##
## data:  Q33 by Q47
## Kruskal-Wallis chi-squared = 16.296, df = 6, p-value = 0.01225
```

```
pairwise.wilcox.test(d$Q33,d$Q47, p.adjust.method = "holm")
```

```
##
##  Pairwise comparisons using Wilcoxon rank sum test with continuity correction
##
## data:  d$Q33 and d$Q47
##
##   1      2      3      4      5      6
## 2 1.0000 -      -      -      -      -
## 3 1.0000 1.0000 -      -      -      -
## 4 1.0000 1.0000 0.0021 -      -      -
## 5 1.0000 1.0000 1.0000 0.1728 -      -
## 6 1.0000 1.0000 1.0000 0.7462 1.0000 -
## 7 1.0000 1.0000 0.8538 0.6503 1.0000 1.0000
##
## P value adjustment method: holm
```

```
# PERCEPTION vs RESIDENCE ####
# Q27
kruskal.test(Q27~Q3, data = d)
```

```
##
##  Kruskal-Wallis rank sum test
##
## data:  Q27 by Q3
```

```
## Kruskal-Wallis chi-squared = 30.074, df = 2, p-value = 2.948e-07
```

```r
pairwise.wilcox.test(d$Q27, d$Q3, p.adjust.method = "holm")
```

```
##
##  Pairwise comparisons using Wilcoxon rank sum test with continuity correction
##
## data:  d$Q27 and d$Q3
##
##   1       2
## 2 0.9     -
## 3 8.7e-05 6.4e-07
##
## P value adjustment method: holm
```

```r
# Q31
kruskal.test(Q31~Q3, data = d) # N.S.
```

```
##
##  Kruskal-Wallis rank sum test
##
## data:  Q31 by Q3
## Kruskal-Wallis chi-squared = 3.3363, df = 2, p-value = 0.1886
```

```r
# Q32
kruskal.test(Q32~Q3, data = d)
```

```
##
##  Kruskal-Wallis rank sum test
##
## data:  Q32 by Q3
## Kruskal-Wallis chi-squared = 8.0721, df = 2, p-value = 0.01767
```

```r
pairwise.wilcox.test(d$Q32,d$Q3, p.adjust.method = "holm")
```

```
##
##  Pairwise comparisons using Wilcoxon rank sum test with continuity correction
##
## data:  d$Q32 and d$Q3
##
##   1     2
## 2 0.762 -
## 3 0.035 0.035
##
## P value adjustment method: holm
```

```r
# Q33
kruskal.test(Q33~Q3, data = d) # N.S.
```

```
##
##  Kruskal-Wallis rank sum test
##
## data:  Q33 by Q3
## Kruskal-Wallis chi-squared = 0.67459, df = 2, p-value = 0.7137
```

```r
# PERCEPTION vs OWNERS PRIMARY all answers ####
# Q27
kruskal.test(Q27~Q15, data = d)
```

```
##
##  Kruskal-Wallis rank sum test
##
## data:  Q27 by Q15
## Kruskal-Wallis chi-squared = 402.53, df = 3, p-value < 2.2e-16
```

```
pairwise.wilcox.test(d$Q27, d$Q15, p.adjust.method = "holm")
```

```
##
##  Pairwise comparisons using Wilcoxon rank sum test with continuity correction
##
## data:  d$Q27 and d$Q15
##
##   1       2       3
## 2 0.0042  -       -
## 3 < 2e-16 < 2e-16 -
## 4 2.4e-06 < 2e-16 0.0042
##
## P value adjustment method: holm
```

```
# Q31
kruskal.test(Q31~Q15, data = d)
```

```
##
##  Kruskal-Wallis rank sum test
##
## data:  Q31 by Q15
## Kruskal-Wallis chi-squared = 67.321, df = 3, p-value = 1.598e-14
```

```
pairwise.wilcox.test(d$Q31, d$Q15, p.adjust.method = "holm")
```

```
##
##  Pairwise comparisons using Wilcoxon rank sum test with continuity correction
##
## data:  d$Q31 and d$Q15
##
##   1      2       3
## 2 0.5072 -       -
## 3 0.0011 8.3e-13 -
## 4 0.0014 1.2e-07 0.5072
##
## P value adjustment method: holm
```

```
# Q32
kruskal.test(Q32~Q15, data = d)
```

```
##
##  Kruskal-Wallis rank sum test
##
## data:  Q32 by Q15
## Kruskal-Wallis chi-squared = 60.217, df = 3, p-value = 5.282e-13
```

```
pairwise.wilcox.test(d$Q32,d$Q15, p.adjust.method = "holm")
```

```
##
##  Pairwise comparisons using Wilcoxon rank sum test with continuity correction
##
## data:  d$Q32 and d$Q15
```

```
##
##   1     2       3
## 2 0.203 -       -
## 3 0.012 1.3e-12 -
## 4 0.041 3.8e-06 0.741
##
## P value adjustment method: holm
```

```r
# Q33
kruskal.test(Q33~Q15, data = d)
```

```
##
##  Kruskal-Wallis rank sum test
##
## data:  Q33 by Q15
## Kruskal-Wallis chi-squared = 43.395, df = 3, p-value = 2.029e-09
```

```r
pairwise.wilcox.test(d$Q33, d$Q15, p.adjust.method = "holm")
```

```
##
##  Pairwise comparisons using Wilcoxon rank sum test with continuity correction
##
## data:  d$Q33 and d$Q15
##
##   1      2       3
## 2 0.1092 -       -
## 3 0.1525 5.2e-10 -
## 4 0.5221 0.0017  0.7079
##
## P value adjustment method: holm
```

```r
# PERCEPTION vs OWNERS PRIMARY recode ####
# Q27
kruskal.test(Q27~Q15_recode, data = d)
```

```
##
##  Kruskal-Wallis rank sum test
##
## data:  Q27 by Q15_recode
## Kruskal-Wallis chi-squared = 28.619, df = 1, p-value = 8.81e-08
```

```r
# Q31
kruskal.test(Q31~Q15_recode, data = d)
```

```
##
##  Kruskal-Wallis rank sum test
##
## data:  Q31 by Q15_recode
## Kruskal-Wallis chi-squared = 7.6439, df = 1, p-value = 0.005697
```

```r
# Q32
kruskal.test(Q32~Q15_recode, data = d) # N.S.
```

```
##
##  Kruskal-Wallis rank sum test
##
## data:  Q32 by Q15_recode
## Kruskal-Wallis chi-squared = 3.3517, df = 1, p-value = 0.06713
```

```r
# Q33
kruskal.test(Q33~Q15_recode, data = d) # N.S.
```

```
##
##  Kruskal-Wallis rank sum test
##
## data:  Q33 by Q15_recode
## Kruskal-Wallis chi-squared = 0.65928, df = 1, p-value = 0.4168
```

```r
# PERCEPTION vs OWNERS SECONDARY recode ####
# Q27
kruskal.test(Q27~Q22_recode, data = d) # N.S.
```

```
##
##  Kruskal-Wallis rank sum test
##
## data:  Q27 by Q22_recode
## Kruskal-Wallis chi-squared = 2.1208, df = 1, p-value = 0.1453
```

```r
# Q31
kruskal.test(Q31~Q22_recode, data = d)
```

```
##
##  Kruskal-Wallis rank sum test
##
## data:  Q31 by Q22_recode
## Kruskal-Wallis chi-squared = 4.5471, df = 1, p-value = 0.03298
```

```r
# Q32
kruskal.test(Q32~Q22_recode, data = d)
```

```
##
##  Kruskal-Wallis rank sum test
##
## data:  Q32 by Q22_recode
## Kruskal-Wallis chi-squared = 5.6043, df = 1, p-value = 0.01792
```

```r
# Q33
kruskal.test(Q33~Q22_recode, data = d) # N.S.
```

```
##
##  Kruskal-Wallis rank sum test
##
## data:  Q33 by Q22_recode
## Kruskal-Wallis chi-squared = 2.3353, df = 1, p-value = 0.1265
```

```r
# PREFERENCE vs LANGUAGE ####
# Q11
kruskal.test(Q11r1~Q4_recode, data = d)
```

```
##
##  Kruskal-Wallis rank sum test
##
## data:  Q11r1 by Q4_recode
## Kruskal-Wallis chi-squared = 55.271, df = 3, p-value = 6.011e-12
```

```r
pairwise.wilcox.test(d$Q11r1, d$Q4_recode, p.adjust.method = "holm")
```

```
##
```

```
##  Pairwise comparisons using Wilcoxon rank sum test with continuity correction
##
## data:  d$Q11r1 and d$Q4_recode
##
##   1       2       3
## 2 0.00021 -       -
## 3 1.8e-12 0.05734 -
## 4 0.32996 0.59555 0.32996
##
## P value adjustment method: holm
```

```
kruskal.test(Q11r2~Q4_recode, data = d)
```

```
##
##  Kruskal-Wallis rank sum test
##
## data:  Q11r2 by Q4_recode
## Kruskal-Wallis chi-squared = 57.561, df = 3, p-value = 1.951e-12
```

```
pairwise.wilcox.test(d$Q11r2, d$Q4_recode, p.adjust.method = "holm")
```

```
##
##  Pairwise comparisons using Wilcoxon rank sum test with continuity correction
##
## data:  d$Q11r2 and d$Q4_recode
##
##   1       2     3
## 2 9.2e-05 -     -
## 3 8.1e-13 0.077 -
## 4 0.367   0.367 0.114
##
## P value adjustment method: holm
```

```
kruskal.test(Q11r3~Q4_recode, data = d)
```

```
##
##  Kruskal-Wallis rank sum test
##
## data:  Q11r3 by Q4_recode
## Kruskal-Wallis chi-squared = 45.764, df = 3, p-value = 6.366e-10
```

```
pairwise.wilcox.test(d$Q11r3, d$Q4_recode, p.adjust.method = "holm")
```

```
##
##  Pairwise comparisons using Wilcoxon rank sum test with continuity correction
##
## data:  d$Q11r3 and d$Q4_recode
##
##   1       2       3
## 2 1.000   -       -
## 3 3.5e-08 1.6e-06 -
## 4 1.000   1.000   0.058
##
## P value adjustment method: holm
```

```
kruskal.test(Q11r4~Q4_recode, data = d)
```

```
##
```

```
##  Kruskal-Wallis rank sum test
##
## data:  Q11r4 by Q4_recode
## Kruskal-Wallis chi-squared = 10.338, df = 3, p-value = 0.0159
```

```r
pairwise.wilcox.test(d$Q11r4, d$Q4_recode, p.adjust.method = "holm")
```

```
##
##  Pairwise comparisons using Wilcoxon rank sum test with continuity correction
##
## data:  d$Q11r4 and d$Q4_recode
##
##   1     2     3
## 2 0.184 -     -
## 3 0.725 0.112 -
## 4 0.184 0.021 0.184
##
## P value adjustment method: holm
```

```r
kruskal.test(Q11r5~Q4_recode, data = d)
```

```
##
##  Kruskal-Wallis rank sum test
##
## data:  Q11r5 by Q4_recode
## Kruskal-Wallis chi-squared = 8.8754, df = 3, p-value = 0.03099
```

```r
pairwise.wilcox.test(d$Q11r5, d$Q4_recode, p.adjust.method = "holm")
```

```
##
##  Pairwise comparisons using Wilcoxon rank sum test with continuity correction
##
## data:  d$Q11r5 and d$Q4_recode
##
##   1     2     3
## 2 0.165 -     -
## 3 0.493 0.493 -
## 4 0.087 0.493 0.208
##
## P value adjustment method: holm
```

```r
kruskal.test(Q11r6~Q4_recode, data = d)
```

```
##
##  Kruskal-Wallis rank sum test
##
## data:  Q11r6 by Q4_recode
## Kruskal-Wallis chi-squared = 9.33, df = 3, p-value = 0.02521
```

```r
pairwise.wilcox.test(d$Q11r6, d$Q4_recode, p.adjust.method = "holm")
```

```
##
##  Pairwise comparisons using Wilcoxon rank sum test with continuity correction
##
## data:  d$Q11r6 and d$Q4_recode
##
##   1     2     3
## 2 0.225 -     -
```

```
## 3 0.250 0.624 -
## 4 0.065 0.253 0.250
##
## P value adjustment method: holm
```

```
kruskal.test(Q11r7~Q4_recode, data = d)
```

```
##
##  Kruskal-Wallis rank sum test
##
## data:  Q11r7 by Q4_recode
## Kruskal-Wallis chi-squared = 23.982, df = 3, p-value = 2.519e-05
```

```
pairwise.wilcox.test(d$Q11r7, d$Q4_recode, p.adjust.method = "holm")
```

```
##
##  Pairwise comparisons using Wilcoxon rank sum test with continuity correction
##
## data:  d$Q11r7 and d$Q4_recode
##
##   1       2      3
## 2 0.0015  -      -
## 3 2.8e-05 1.0000 -
## 4 1.0000  1.0000 1.0000
##
## P value adjustment method: holm
```

```
kruskal.test(Q11r8~Q4_recode, data = d) # N.S.
```

```
##
##  Kruskal-Wallis rank sum test
##
## data:  Q11r8 by Q4_recode
## Kruskal-Wallis chi-squared = 3.3693, df = 3, p-value = 0.3381
```

```
# Q28
kruskal.test(Q28~Q4_recode, data = d)
```

```
##
##  Kruskal-Wallis rank sum test
##
## data:  Q28 by Q4_recode
## Kruskal-Wallis chi-squared = 36.161, df = 3, p-value = 6.922e-08
```

```
pairwise.wilcox.test(d$Q28,d$Q4_recode, p.adjust.method = "holm")
```

```
##
##  Pairwise comparisons using Wilcoxon rank sum test with continuity correction
##
## data:  d$Q28 and d$Q4_recode
##
##   1       2       3
## 2 0.82    -       -
## 3 5.8e-05 1.9e-06 -
## 4 0.82    0.82    0.02
##
## P value adjustment method: holm
```

```r
# Q34
kruskal.test(Q34r1~Q4_recode, data = d)
```

```
##
##  Kruskal-Wallis rank sum test
##
## data:  Q34r1 by Q4_recode
## Kruskal-Wallis chi-squared = 10.265, df = 3, p-value = 0.01644
```

```r
pairwise.wilcox.test(d$Q34r1,d$Q4_recode, p.adjust.method = "holm")
```

```
##
##  Pairwise comparisons using Wilcoxon rank sum test with continuity correction
##
## data:  d$Q34r1 and d$Q4_recode
##
##   1     2     3
## 2 0.042 -     -
## 3 0.094 0.880 -
## 4 0.112 0.880 0.844
##
## P value adjustment method: holm
```

```r
kruskal.test(Q34r2~Q4_recode, data = d) # N.S.
```

```
##
##  Kruskal-Wallis rank sum test
##
## data:  Q34r2 by Q4_recode
## Kruskal-Wallis chi-squared = 0.8441, df = 3, p-value = 0.8389
```

```r
kruskal.test(Q34r3~Q4_recode, data = d)
```

```
##
##  Kruskal-Wallis rank sum test
##
## data:  Q34r3 by Q4_recode
## Kruskal-Wallis chi-squared = 72.001, df = 3, p-value = 1.591e-15
```

```r
pairwise.wilcox.test(d$Q34r3,d$Q4_recode, p.adjust.method = "holm")
```

```
##
##  Pairwise comparisons using Wilcoxon rank sum test with continuity correction
##
## data:  d$Q34r3 and d$Q4_recode
##
##   1       2       3
## 2 0.00014 -       -
## 3 < 2e-16 0.01313 -
## 4 0.17006 0.67490 0.17006
##
## P value adjustment method: holm
```

```r
# Q40
kruskal.test(IT2_1~Q4_recode, data = d)
```

```
##
##  Kruskal-Wallis rank sum test
```

```
##
## data:  IT2_1 by Q4_recode
## Kruskal-Wallis chi-squared = 18.588, df = 3, p-value = 0.0003326
```

```
pairwise.wilcox.test(d$IT2_1,d$Q4_recode, p.adjust.method = "holm")
```

```
##
##  Pairwise comparisons using Wilcoxon rank sum test with continuity correction
##
## data:  d$IT2_1 and d$Q4_recode
##
##   1       2       3
## 2 0.07936 -       -
## 3 0.00014 0.71714 -
## 4 0.81245 0.81245 0.64993
##
## P value adjustment method: holm
```

```
kruskal.test(IT2_2~Q4_recode, data = d)
```

```
##
##  Kruskal-Wallis rank sum test
##
## data:  IT2_2 by Q4_recode
## Kruskal-Wallis chi-squared = 8.2256, df = 3, p-value = 0.04157
```

```
pairwise.wilcox.test(d$IT2_2,d$Q4_recode, p.adjust.method = "holm")
```

```
##
##  Pairwise comparisons using Wilcoxon rank sum test with continuity correction
##
## data:  d$IT2_2 and d$Q4_recode
##
##   1     2     3
## 2 0.332 -     -
## 3 0.077 1.000 -
## 4 0.332 1.000 1.000
##
## P value adjustment method: holm
```

```
kruskal.test(IT2_3~Q4_recode, data = d) # N.S.
```

```
##
##  Kruskal-Wallis rank sum test
##
## data:  IT2_3 by Q4_recode
## Kruskal-Wallis chi-squared = 2.0887, df = 3, p-value = 0.5542
```

```
kruskal.test(IT2_4~Q4_recode, data = d) # N.S.
```

```
##
##  Kruskal-Wallis rank sum test
##
## data:  IT2_4 by Q4_recode
## Kruskal-Wallis chi-squared = 1.644, df = 3, p-value = 0.6495
```

```
kruskal.test(IT2_5~Q4_recode, data = d)
```

```
##
##  Kruskal-Wallis rank sum test
##
## data:  IT2_5 by Q4_recode
## Kruskal-Wallis chi-squared = 21.095, df = 3, p-value = 0.0001006
```

```
pairwise.wilcox.test(d$IT2_5,d$Q4_recode, p.adjust.method = "holm")
```

```
##
##  Pairwise comparisons using Wilcoxon rank sum test with continuity correction
##
## data:  d$IT2_5 and d$Q4_recode
##
##   1       2     3
## 2 0.097   -     -
## 3 2.6e-05 0.540 -
## 4 0.550   1.000 1.000
##
## P value adjustment method: holm
```

```
kruskal.test(IT2_6~Q4_recode, data = d) # N.S.
```

```
##
##  Kruskal-Wallis rank sum test
##
## data:  IT2_6 by Q4_recode
## Kruskal-Wallis chi-squared = 3.9459, df = 3, p-value = 0.2674
```

```
kruskal.test(IT2_7~Q4_recode, data = d) # N.S.
```

```
##
##  Kruskal-Wallis rank sum test
##
## data:  IT2_7 by Q4_recode
## Kruskal-Wallis chi-squared = 1.2673, df = 3, p-value = 0.7369
```

```
kruskal.test(IT2_8~Q4_recode, data = d) # N.S.
```

```
##
##  Kruskal-Wallis rank sum test
##
## data:  IT2_8 by Q4_recode
## Kruskal-Wallis chi-squared = 2.5276, df = 3, p-value = 0.4703
```

```
# PREFERENCE vs AGE ####
# Q11
kruskal.test(Q11r1~RECODE_AGE, data = d)
```

```
##
##  Kruskal-Wallis rank sum test
##
## data:  Q11r1 by RECODE_AGE
## Kruskal-Wallis chi-squared = 76.974, df = 4, p-value = 7.618e-16
```

```
pairwise.wilcox.test(d$Q11r1, d$RECODE_AGE, p.adjust.method = "holm")
```

```
##
##  Pairwise comparisons using Wilcoxon rank sum test with continuity correction
##
```

```
## data:  d$Q11r1 and d$RECODE_AGE
##
##   1       2       3       4
## 2 1.000   -       -       -
## 3 0.838   1.000   -       -
## 4 0.029   0.081   0.428   -
## 5 2.0e-12 1.5e-11 5.6e-09 1.6e-06
##
## P value adjustment method: holm
```

```r
kruskal.test(Q11r2~RECODE_AGE, data = d)
```

```
##
##  Kruskal-Wallis rank sum test
##
## data:  Q11r2 by RECODE_AGE
## Kruskal-Wallis chi-squared = 32.21, df = 4, p-value = 1.733e-06
```

```r
pairwise.wilcox.test(d$Q11r2, d$RECODE_AGE, p.adjust.method = "holm")
```

```
##
##  Pairwise comparisons using Wilcoxon rank sum test with continuity correction
##
## data:  d$Q11r2 and d$RECODE_AGE
##
##   1       2       3       4
## 2 1.00000 -       -       -
## 3 1.00000 1.00000 -       -
## 4 0.14743 0.08365 0.14743 -
## 5 0.00018 3.3e-05 0.00024 0.10659
##
## P value adjustment method: holm
```

```r
kruskal.test(Q11r3~RECODE_AGE, data = d)
```

```
##
##  Kruskal-Wallis rank sum test
##
## data:  Q11r3 by RECODE_AGE
## Kruskal-Wallis chi-squared = 38.974, df = 4, p-value = 7.052e-08
```

```r
pairwise.wilcox.test(d$Q11r3, d$RECODE_AGE, p.adjust.method = "holm")
```

```
##
##  Pairwise comparisons using Wilcoxon rank sum test with continuity correction
##
## data:  d$Q11r3 and d$RECODE_AGE
##
##   1       2       3       4
## 2 0.30664 -       -       -
## 3 0.33980 0.88519 -       -
## 4 0.00215 0.21822 0.20331 -
## 5 2.1e-07 0.00034 0.00040 0.10377
##
## P value adjustment method: holm
```

```r
kruskal.test(Q11r4~RECODE_AGE, data = d)
```

```
##
##  Kruskal-Wallis rank sum test
##
## data:  Q11r4 by RECODE_AGE
## Kruskal-Wallis chi-squared = 25.252, df = 4, p-value = 4.477e-05
```

```r
pairwise.wilcox.test(d$Q11r4, d$RECODE_AGE, p.adjust.method = "holm")
```

```
##
##  Pairwise comparisons using Wilcoxon rank sum test with continuity correction
##
## data:  d$Q11r4 and d$RECODE_AGE
##
##   1      2       3      4
## 2 0.9066 -       -      -
## 3 0.9066 0.7213  -      -
## 4 0.1927 0.0136  0.2940 -
## 5 0.0079 8.6e-05 0.0136 0.6999
##
## P value adjustment method: holm
```

```r
kruskal.test(Q11r5~RECODE_AGE, data = d)
```

```
##
##  Kruskal-Wallis rank sum test
##
## data:  Q11r5 by RECODE_AGE
## Kruskal-Wallis chi-squared = 11.846, df = 4, p-value = 0.01853
```

```r
pairwise.wilcox.test(d$Q11r5, d$RECODE_AGE, p.adjust.method = "holm")
```

```
##
##  Pairwise comparisons using Wilcoxon rank sum test with continuity correction
##
## data:  d$Q11r5 and d$RECODE_AGE
##
##   1     2     3     4
## 2 1.000 -     -     -
## 3 1.000 1.000 -     -
## 4 1.000 0.215 0.846 -
## 5 0.507 0.019 0.162 1.000
##
## P value adjustment method: holm
```

```r
kruskal.test(Q11r6~RECODE_AGE, data = d)
```

```
##
##  Kruskal-Wallis rank sum test
##
## data:  Q11r6 by RECODE_AGE
## Kruskal-Wallis chi-squared = 19.181, df = 4, p-value = 0.000724
```

```r
pairwise.wilcox.test(d$Q11r6, d$RECODE_AGE, p.adjust.method = "holm")
```

```
##
```

```
##  Pairwise comparisons using Wilcoxon rank sum test with continuity correction
##
## data:  d$Q11r6 and d$RECODE_AGE
##
##   1       2       3       4
## 2 0.41706 -       -       -
## 3 0.88117 0.96786 -       -
## 4 0.96786 0.06299 0.37613 -
## 5 0.37613 0.00076 0.01116 0.47264
##
## P value adjustment method: holm
```

```r
kruskal.test(Q11r7~RECODE_AGE, data = d)
```

```
##
##  Kruskal-Wallis rank sum test
##
## data:  Q11r7 by RECODE_AGE
## Kruskal-Wallis chi-squared = 78.541, df = 4, p-value = 3.549e-16
```

```r
pairwise.wilcox.test(d$Q11r7, d$RECODE_AGE, p.adjust.method = "holm")
```

```
##
##  Pairwise comparisons using Wilcoxon rank sum test with continuity correction
##
## data:  d$Q11r7 and d$RECODE_AGE
##
##   1       2       3       4
## 2 0.4413  -       -       -
## 3 0.6261  0.2532  -       -
## 4 0.0051  2.2e-05 0.0141  -
## 5 2.0e-09 1.1e-13 8.8e-09 0.0040
##
## P value adjustment method: holm
```

```r
kruskal.test(Q11r8~RECODE_AGE, data = d)
```

```
##
##  Kruskal-Wallis rank sum test
##
## data:  Q11r8 by RECODE_AGE
## Kruskal-Wallis chi-squared = 28.279, df = 4, p-value = 1.095e-05
```

```r
pairwise.wilcox.test(d$Q11r8, d$RECODE_AGE, p.adjust.method = "holm")
```

```
##
##  Pairwise comparisons using Wilcoxon rank sum test with continuity correction
##
## data:  d$Q11r8 and d$RECODE_AGE
##
##   1       2       3       4
## 2 1.00000 -       -       -
## 3 1.00000 1.00000 -       -
## 4 1.00000 1.00000 1.00000 -
## 5 0.00503 0.00029 0.00526 1.9e-05
##
## P value adjustment method: holm
```

```r
# Q28
kruskal.test(Q28~RECODE_AGE, data = d)
```

```
##
##  Kruskal-Wallis rank sum test
##
## data:  Q28 by RECODE_AGE
## Kruskal-Wallis chi-squared = 63.016, df = 4, p-value = 6.735e-13
```

```r
pairwise.wilcox.test(d$Q28,d$RECODE_AGE, p.adjust.method = "holm")
```

```
##
##  Pairwise comparisons using Wilcoxon rank sum test with continuity correction
##
## data:  d$Q28 and d$RECODE_AGE
##
##   1       2       3       4
## 2 0.64766 -       -       -
## 3 0.69349 0.48132 -       -
## 4 0.02767 0.00089 0.07381 -
## 5 3.4e-08 4.5e-11 2.6e-07 0.00446
##
## P value adjustment method: holm
```

```r
# Q34
kruskal.test(Q34r1~RECODE_AGE, data = d) # N.S.
```

```
##
##  Kruskal-Wallis rank sum test
##
## data:  Q34r1 by RECODE_AGE
## Kruskal-Wallis chi-squared = 3.0587, df = 4, p-value = 0.5481
```

```r
kruskal.test(Q34r2~RECODE_AGE, data = d)
```

```
##
##  Kruskal-Wallis rank sum test
##
## data:  Q34r2 by RECODE_AGE
## Kruskal-Wallis chi-squared = 18.543, df = 4, p-value = 0.0009661
```

```r
pairwise.wilcox.test(d$Q34r2,d$RECODE_AGE, p.adjust.method = "holm")
```

```
##
##  Pairwise comparisons using Wilcoxon rank sum test with continuity correction
##
## data:  d$Q34r2 and d$RECODE_AGE
##
##   1      2      3      4
## 2 1.0000 -      -      -
## 3 1.0000 1.0000 -      -
## 4 1.0000 1.0000 1.0000 -
## 5 0.0055 0.0085 0.0125 0.0438
##
## P value adjustment method: holm
```

```r
kruskal.test(Q34r3~RECODE_AGE, data = d)
```

```
##
##  Kruskal-Wallis rank sum test
##
## data:  Q34r3 by RECODE_AGE
## Kruskal-Wallis chi-squared = 10.82, df = 4, p-value = 0.02867
```

```r
pairwise.wilcox.test(d$Q34r3,d$RECODE_AGE, p.adjust.method = "holm")
```

```
##
##  Pairwise comparisons using Wilcoxon rank sum test with continuity correction
##
## data:  d$Q34r3 and d$RECODE_AGE
##
##   1     2     3     4
## 2 0.396 -     -     -
## 3 0.046 1.000 -     -
## 4 0.028 1.000 1.000 -
## 5 0.396 1.000 1.000 1.000
##
## P value adjustment method: holm
```

```r
# Q40
kruskal.test(IT2_1~RECODE_AGE, data = d) # N.S.
```

```
##
##  Kruskal-Wallis rank sum test
##
## data:  IT2_1 by RECODE_AGE
## Kruskal-Wallis chi-squared = 5.6635, df = 4, p-value = 0.2257
```

```r
kruskal.test(IT2_2~RECODE_AGE, data = d)
```

```
##
##  Kruskal-Wallis rank sum test
##
## data:  IT2_2 by RECODE_AGE
## Kruskal-Wallis chi-squared = 16.847, df = 4, p-value = 0.00207
```

```r
pairwise.wilcox.test(d$IT2_2,d$RECODE_AGE, p.adjust.method = "holm")
```

```
##
##  Pairwise comparisons using Wilcoxon rank sum test with continuity correction
##
## data:  d$IT2_2 and d$RECODE_AGE
##
##   1      2      3      4
## 2 0.8865 -      -      -
## 3 1.0000 1.0000 -      -
## 4 0.2492 1.0000 1.0000 -
## 5 0.0014 0.1368 0.0394 0.2529
##
## P value adjustment method: holm
```

```r
kruskal.test(IT2_3~RECODE_AGE, data = d)
```

```
##
```

```
##  Kruskal-Wallis rank sum test
##
## data:  IT2_3 by RECODE_AGE
## Kruskal-Wallis chi-squared = 41.895, df = 4, p-value = 1.754e-08
```

```
pairwise.wilcox.test(d$IT2_3,d$RECODE_AGE, p.adjust.method = "holm")
```

```
##
##  Pairwise comparisons using Wilcoxon rank sum test with continuity correction
##
## data:  d$IT2_3 and d$RECODE_AGE
##
##   1       2      3      4
## 2 0.0293  -      -      -
## 3 0.0992  0.6484 -      -
## 4 6.8e-05 0.3261 0.1816 -
## 5 7.8e-09 0.0085 0.0019 0.1315
##
## P value adjustment method: holm
```

```
kruskal.test(IT2_4~RECODE_AGE, data = d)
```

```
##
##  Kruskal-Wallis rank sum test
##
## data:  IT2_4 by RECODE_AGE
## Kruskal-Wallis chi-squared = 17.841, df = 4, p-value = 0.001326
```

```
pairwise.wilcox.test(d$IT2_4,d$RECODE_AGE, p.adjust.method = "holm")
```

```
##
##  Pairwise comparisons using Wilcoxon rank sum test with continuity correction
##
## data:  d$IT2_4 and d$RECODE_AGE
##
##   1     2     3     4
## 2 1.000 -     -     -
## 3 1.000 1.000 -     -
## 4 0.113 0.113 0.152 -
## 5 0.023 0.021 0.036 1.000
##
## P value adjustment method: holm
```

```
kruskal.test(IT2_5~RECODE_AGE, data = d) # N.S.
```

```
##
##  Kruskal-Wallis rank sum test
##
## data:  IT2_5 by RECODE_AGE
## Kruskal-Wallis chi-squared = 4.6839, df = 4, p-value = 0.3213
```

```
kruskal.test(IT2_6~RECODE_AGE, data = d)
```

```
##
##  Kruskal-Wallis rank sum test
##
## data:  IT2_6 by RECODE_AGE
## Kruskal-Wallis chi-squared = 44.622, df = 4, p-value = 4.765e-09
```

```r
pairwise.wilcox.test(d$IT2_6,d$RECODE_AGE, p.adjust.method = "holm")
```

```
##
##  Pairwise comparisons using Wilcoxon rank sum test with continuity correction
##
## data:  d$IT2_6 and d$RECODE_AGE
##
##   1       2       3       4
## 2 0.75059 -       -       -
## 3 0.40324 0.75059 -       -
## 4 7.0e-05 0.00086 0.04775 -
## 5 1.4e-06 2.5e-05 0.00296 0.75059
##
## P value adjustment method: holm
```

```r
kruskal.test(IT2_7~RECODE_AGE, data = d) # N.S.
```

```
##
##  Kruskal-Wallis rank sum test
##
## data:  IT2_7 by RECODE_AGE
## Kruskal-Wallis chi-squared = 1.6856, df = 4, p-value = 0.7933
```

```r
kruskal.test(IT2_8~RECODE_AGE, data = d) # N.S.
```

```
##
##  Kruskal-Wallis rank sum test
##
## data:  IT2_8 by RECODE_AGE
## Kruskal-Wallis chi-squared = 5.1581, df = 4, p-value = 0.2715
```

```r
# PREFERENCE vs EDUCATION ####
# Q11
kruskal.test(Q11r1~Q47, data = d)
```

```
##
##  Kruskal-Wallis rank sum test
##
## data:  Q11r1 by Q47
## Kruskal-Wallis chi-squared = 17.34, df = 6, p-value = 0.008112
```

```r
pairwise.wilcox.test(d$Q11r1, d$Q47, p.adjust.method = "holm")
```

```
##
##  Pairwise comparisons using Wilcoxon rank sum test with continuity correction
##
## data:  d$Q11r1 and d$Q47
##
##   1      2      3      4      5      6
## 2 1.0000 -      -      -      -      -
## 3 1.0000 1.0000 -      -      -      -
## 4 1.0000 1.0000 0.0019 -      -      -
## 5 1.0000 1.0000 1.0000 0.0387 -      -
## 6 1.0000 1.0000 1.0000 0.0368 1.0000 -
## 7 1.0000 1.0000 1.0000 0.0363 1.0000 1.0000
##
## P value adjustment method: holm
```

```r
kruskal.test(Q11r2~Q47, data = d)
```

```
##
##  Kruskal-Wallis rank sum test
##
## data:  Q11r2 by Q47
## Kruskal-Wallis chi-squared = 14.516, df = 6, p-value = 0.02437
```

```r
pairwise.wilcox.test(d$Q11r2, d$Q47, p.adjust.method = "holm")
```

```
##
##  Pairwise comparisons using Wilcoxon rank sum test with continuity correction
##
## data:  d$Q11r2 and d$Q47
##
##   1      2      3      4      5      6
## 2 1.0000 -      -      -      -      -
## 3 1.0000 1.0000 -      -      -      -
## 4 1.0000 1.0000 1.0000 -      -      -
## 5 1.0000 1.0000 1.0000 1.0000 -      -
## 6 1.0000 1.0000 0.1301 0.0074 0.4935 -
## 7 1.0000 1.0000 1.0000 1.0000 1.0000 0.1227
##
## P value adjustment method: holm
```

```r
kruskal.test(Q11r3~Q47, data = d)
```

```
##
##  Kruskal-Wallis rank sum test
##
## data:  Q11r3 by Q47
## Kruskal-Wallis chi-squared = 18.306, df = 6, p-value = 0.005511
```

```r
pairwise.wilcox.test(d$Q11r3, d$Q47, p.adjust.method = "holm")
```

```
##
##  Pairwise comparisons using Wilcoxon rank sum test with continuity correction
##
## data:  d$Q11r3 and d$Q47
##
##   1      2      3      4      5      6
## 2 1.0000 -      -      -      -      -
## 3 1.0000 1.0000 -      -      -      -
## 4 1.0000 1.0000 1.0000 -      -      -
## 5 1.0000 1.0000 1.0000 1.0000 -      -
## 6 1.0000 0.1948 0.0068 0.0049 0.2343 -
## 7 1.0000 1.0000 1.0000 1.0000 1.0000 0.0216
##
## P value adjustment method: holm
```

```r
kruskal.test(Q11r4~Q47, data = d) # N.S.
```

```
##
##  Kruskal-Wallis rank sum test
##
## data:  Q11r4 by Q47
## Kruskal-Wallis chi-squared = 9.0511, df = 6, p-value = 0.1707
```

```
kruskal.test(Q11r5~Q47, data = d) # N.S.
```

```
##
##  Kruskal-Wallis rank sum test
##
## data:  Q11r5 by Q47
## Kruskal-Wallis chi-squared = 5.6864, df = 6, p-value = 0.4592
```

```
kruskal.test(Q11r6~Q47, data = d) # N.S.
```

```
##
##  Kruskal-Wallis rank sum test
##
## data:  Q11r6 by Q47
## Kruskal-Wallis chi-squared = 4.2597, df = 6, p-value = 0.6416
```

```
kruskal.test(Q11r7~Q47, data = d)
```

```
##
##  Kruskal-Wallis rank sum test
##
## data:  Q11r7 by Q47
## Kruskal-Wallis chi-squared = 20.415, df = 6, p-value = 0.002335
```

```
pairwise.wilcox.test(d$Q11r7, d$Q47, p.adjust.method = "holm")
```

```
##
##  Pairwise comparisons using Wilcoxon rank sum test with continuity correction
##
## data:  d$Q11r7 and d$Q47
##
##   1      2      3      4      5      6
## 2 1.0000 -      -      -      -      -
## 3 1.0000 1.0000 -      -      -      -
## 4 1.0000 0.0514 0.0027 -      -      -
## 5 1.0000 0.6776 0.6776 1.0000 -      -
## 6 1.0000 1.0000 1.0000 0.0804 1.0000 -
## 7 1.0000 0.7950 1.0000 0.6776 1.0000 1.0000
##
## P value adjustment method: holm
```

```
kruskal.test(Q11r8~Q47, data = d) # N.S.
```

```
##
##  Kruskal-Wallis rank sum test
##
## data:  Q11r8 by Q47
## Kruskal-Wallis chi-squared = 10.891, df = 6, p-value = 0.09179
```

```
# Q28
kruskal.test(Q28~Q47, data = d)
```

```
##
##  Kruskal-Wallis rank sum test
##
## data:  Q28 by Q47
## Kruskal-Wallis chi-squared = 24.509, df = 6, p-value = 0.0004209
```

```r
pairwise.wilcox.test(d$Q28,d$Q47, p.adjust.method = "holm")
```

```
##
##  Pairwise comparisons using Wilcoxon rank sum test with continuity correction
##
## data:  d$Q28 and d$Q47
##
##   1      2      3      4      5      6
## 2 1.0000 -      -      -      -      -
## 3 1.0000 0.0626 -      -      -      -
## 4 1.0000 1.0000 0.1208 -      -      -
## 5 1.0000 0.0543 1.0000 0.1202 -      -
## 6 1.0000 0.0051 0.6457 0.0040 1.0000 -
## 7 1.0000 0.2489 1.0000 0.9487 1.0000 0.2489
##
## P value adjustment method: holm
```

```r
# Q34
kruskal.test(Q34r1~Q47, data = d)
```

```
##
##  Kruskal-Wallis rank sum test
##
## data:  Q34r1 by Q47
## Kruskal-Wallis chi-squared = 15.042, df = 6, p-value = 0.01993
```

```r
pairwise.wilcox.test(d$Q34r1,d$Q47, p.adjust.method = "holm")
```

```
##
##  Pairwise comparisons using Wilcoxon rank sum test with continuity correction
##
## data:  d$Q34r1 and d$Q47
##
##   1     2     3     4     5     6
## 2 1.000 -     -     -     -     -
## 3 1.000 0.101 -     -     -     -
## 4 1.000 0.374 1.000 -     -     -
## 5 1.000 1.000 0.836 1.000 -     -
## 6 1.000 0.075 1.000 1.000 0.622 -
## 7 1.000 0.037 1.000 1.000 0.320 1.000
##
## P value adjustment method: holm
```

```r
kruskal.test(Q34r2~Q47, data = d) # N.S.
```

```
##
##  Kruskal-Wallis rank sum test
##
## data:  Q34r2 by Q47
## Kruskal-Wallis chi-squared = 12.349, df = 6, p-value = 0.05462
```

```r
kruskal.test(Q34r3~Q47, data = d) # N.S.
```

```
##
##  Kruskal-Wallis rank sum test
##
## data:  Q34r3 by Q47
```

```
## Kruskal-Wallis chi-squared = 8.6759, df = 6, p-value = 0.1926
```

```r
# Q40
kruskal.test(IT2_1~Q47, data = d) # N.S.
```

```
##
##  Kruskal-Wallis rank sum test
##
## data:  IT2_1 by Q47
## Kruskal-Wallis chi-squared = 5.9838, df = 6, p-value = 0.425
```

```r
kruskal.test(IT2_2~Q47, data = d)
```

```
##
##  Kruskal-Wallis rank sum test
##
## data:  IT2_2 by Q47
## Kruskal-Wallis chi-squared = 16.997, df = 6, p-value = 0.009292
```

```r
pairwise.wilcox.test(d$IT2_2,d$Q47, p.adjust.method = "holm")
```

```
##
##  Pairwise comparisons using Wilcoxon rank sum test with continuity correction
##
## data:  d$IT2_2 and d$Q47
##
##   1     2     3     4     5     6
## 2 1.000 -     -     -     -     -
## 3 1.000 1.000 -     -     -     -
## 4 0.646 1.000 0.055 -     -     -
## 5 1.000 1.000 1.000 0.013 -     -
## 6 1.000 1.000 1.000 0.020 1.000 -
## 7 1.000 1.000 1.000 0.079 1.000 1.000
##
## P value adjustment method: holm
```

```r
kruskal.test(IT2_3~Q47, data = d)
```

```
##
##  Kruskal-Wallis rank sum test
##
## data:  IT2_3 by Q47
## Kruskal-Wallis chi-squared = 29.118, df = 6, p-value = 5.779e-05
```

```r
pairwise.wilcox.test(d$IT2_3,d$Q47, p.adjust.method = "holm")
```

```
##
##  Pairwise comparisons using Wilcoxon rank sum test with continuity correction
##
## data:  d$IT2_3 and d$Q47
##
##   1      2      3       4      5      6
## 2 1.0000 -      -       -      -      -
## 3 1.0000 0.6211 -       -      -      -
## 4 1.0000 1.0000 4.5e-05 -      -      -
## 5 1.0000 1.0000 1.0000  0.0203 -      -
## 6 1.0000 0.8259 1.0000  0.0016 1.0000 -
## 7 1.0000 1.0000 0.1364  0.4492 1.0000 0.5635
```

```
##
## P value adjustment method: holm
```

```
kruskal.test(IT2_4~Q47, data = d)
```

```
##
##  Kruskal-Wallis rank sum test
##
## data:  IT2_4 by Q47
## Kruskal-Wallis chi-squared = 17.982, df = 6, p-value = 0.006279
```

```
pairwise.wilcox.test(d$IT2_4,d$Q47, p.adjust.method = "holm")
```

```
##
##  Pairwise comparisons using Wilcoxon rank sum test with continuity correction
##
## data:  d$IT2_4 and d$Q47
##
##   1     2     3     4     5     6
## 2 1.000 -     -     -     -     -
## 3 1.000 1.000 -     -     -     -
## 4 1.000 1.000 0.006 -     -     -
## 5 1.000 1.000 1.000 0.984 -     -
## 6 1.000 1.000 1.000 0.029 1.000 -
## 7 1.000 1.000 1.000 1.000 1.000 1.000
##
## P value adjustment method: holm
```

```
kruskal.test(IT2_5~Q47, data = d) # N.S.
```

```
##
##  Kruskal-Wallis rank sum test
##
## data:  IT2_5 by Q47
## Kruskal-Wallis chi-squared = 4.7893, df = 6, p-value = 0.5711
```

```
kruskal.test(IT2_6~Q47, data = d)
```

```
##
##  Kruskal-Wallis rank sum test
##
## data:  IT2_6 by Q47
## Kruskal-Wallis chi-squared = 22.434, df = 6, p-value = 0.00101
```

```
pairwise.wilcox.test(d$IT2_6,d$Q47, p.adjust.method = "holm")
```

```
##
##  Pairwise comparisons using Wilcoxon rank sum test with continuity correction
##
## data:  d$IT2_6 and d$Q47
##
##   1      2      3      4      5      6
## 2 1.0000 -      -      -      -      -
## 3 1.0000 1.0000 -      -      -      -
## 4 1.0000 1.0000 0.0083 -      -      -
## 5 1.0000 1.0000 1.0000 0.4586 -      -
## 6 1.0000 0.7649 1.0000 0.0042 1.0000 -
## 7 1.0000 1.0000 0.4599 1.0000 1.0000 0.1773
```

```
##
## P value adjustment method: holm
```

```r
kruskal.test(IT2_7~Q47, data = d) # N.S.
```

```
##
##  Kruskal-Wallis rank sum test
##
## data:  IT2_7 by Q47
## Kruskal-Wallis chi-squared = 12.234, df = 6, p-value = 0.05696
```

```r
kruskal.test(IT2_8~Q47, data = d)
```

```
##
##  Kruskal-Wallis rank sum test
##
## data:  IT2_8 by Q47
## Kruskal-Wallis chi-squared = 20.483, df = 6, p-value = 0.002271
```

```r
pairwise.wilcox.test(d$IT2_8,d$Q47, p.adjust.method = "holm")
```

```
##
##  Pairwise comparisons using Wilcoxon rank sum test with continuity correction
##
## data:  d$IT2_8 and d$Q47
##
##   1      2      3      4      5      6
## 2 1.0000 -      -      -      -      -
## 3 1.0000 1.0000 -      -      -      -
## 4 1.0000 1.0000 0.0160 -      -      -
## 5 1.0000 1.0000 1.0000 0.0042 -      -
## 6 1.0000 1.0000 1.0000 0.0027 1.0000 -
## 7 1.0000 1.0000 1.0000 0.0384 1.0000 1.0000
##
## P value adjustment method: holm
```

```r
# PREFERENCE vs RESIDENCE ####
# Q11
kruskal.test(Q11r1~Q3, data = d)  # N.S.
```

```
##
##  Kruskal-Wallis rank sum test
##
## data:  Q11r1 by Q3
## Kruskal-Wallis chi-squared = 5.2266, df = 2, p-value = 0.07329
```

```r
kruskal.test(Q11r2~Q3, data = d)
```

```
##
##  Kruskal-Wallis rank sum test
##
## data:  Q11r2 by Q3
## Kruskal-Wallis chi-squared = 17.491, df = 2, p-value = 0.0001592
```

```r
pairwise.wilcox.test(d$Q11r2, d$Q3, p.adjust.method = "holm")
```

```
##
##  Pairwise comparisons using Wilcoxon rank sum test with continuity correction
##
```

```
## data:  d$Q11r2 and d$Q3
##
##   1       2
## 2 0.57012 -
## 3 0.00090 0.00071
##
## P value adjustment method: holm
```

```r
kruskal.test(Q11r3~Q3, data = d)
```

```
##
##  Kruskal-Wallis rank sum test
##
## data:  Q11r3 by Q3
## Kruskal-Wallis chi-squared = 25.81, df = 2, p-value = 2.485e-06
```

```r
pairwise.wilcox.test(d$Q11r3, d$Q3, p.adjust.method = "holm")
```

```
##
##  Pairwise comparisons using Wilcoxon rank sum test with continuity correction
##
## data:  d$Q11r3 and d$Q3
##
##   1       2
## 2 0.98629 -
## 3 0.00017 7.2e-06
##
## P value adjustment method: holm
```

```r
kruskal.test(Q11r4~Q3, data = d)
```

```
##
##  Kruskal-Wallis rank sum test
##
## data:  Q11r4 by Q3
## Kruskal-Wallis chi-squared = 30.477, df = 2, p-value = 2.41e-07
```

```r
pairwise.wilcox.test(d$Q11r4, d$Q3, p.adjust.method = "holm")
```

```
##
##  Pairwise comparisons using Wilcoxon rank sum test with continuity correction
##
## data:  d$Q11r4 and d$Q3
##
##   1       2
## 2 0.34    -
## 3 4.4e-06 4.6e-06
##
## P value adjustment method: holm
```

```r
kruskal.test(Q11r5~Q3, data = d)
```

```
##
##  Kruskal-Wallis rank sum test
##
## data:  Q11r5 by Q3
## Kruskal-Wallis chi-squared = 35.046, df = 2, p-value = 2.454e-08
```

```r
pairwise.wilcox.test(d$Q11r5, d$Q3, p.adjust.method = "holm")
```

```
##
##  Pairwise comparisons using Wilcoxon rank sum test with continuity correction
##
## data:  d$Q11r5 and d$Q3
##
##   1       2
## 2 0.031   -
## 3 7.4e-08 1.8e-05
##
## P value adjustment method: holm
```

```r
kruskal.test(Q11r6~Q3, data = d)
```

```
##
##  Kruskal-Wallis rank sum test
##
## data:  Q11r6 by Q3
## Kruskal-Wallis chi-squared = 31.737, df = 2, p-value = 1.284e-07
```

```r
pairwise.wilcox.test(d$Q11r6, d$Q3, p.adjust.method = "holm")
```

```
##
##  Pairwise comparisons using Wilcoxon rank sum test with continuity correction
##
## data:  d$Q11r6 and d$Q3
##
##   1       2
## 2 0.0151  -
## 3 1.3e-07 0.0002
##
## P value adjustment method: holm
```

```r
kruskal.test(Q11r7~Q3, data = d)
```

```
##
##  Kruskal-Wallis rank sum test
##
## data:  Q11r7 by Q3
## Kruskal-Wallis chi-squared = 35.455, df = 2, p-value = 2e-08
```

```r
pairwise.wilcox.test(d$Q11r7, d$Q3, p.adjust.method = "holm")
```

```
##
##  Pairwise comparisons using Wilcoxon rank sum test with continuity correction
##
## data:  d$Q11r7 and d$Q3
##
##   1       2
## 2 0.38    -
## 3 7.3e-07 6.0e-07
##
## P value adjustment method: holm
```

```r
kruskal.test(Q11r8~Q3, data = d)  # N.S.
```

```
##
##  Kruskal-Wallis rank sum test
##
## data:  Q11r8 by Q3
## Kruskal-Wallis chi-squared = 4.6094, df = 2, p-value = 0.09979
```

```r
# Q28
kruskal.test(Q28~Q3, data = d)
```

```
##
##  Kruskal-Wallis rank sum test
##
## data:  Q28 by Q3
## Kruskal-Wallis chi-squared = 29.763, df = 2, p-value = 3.445e-07
```

```r
pairwise.wilcox.test(d$Q28,d$Q3, p.adjust.method = "holm")
```

```
##
##  Pairwise comparisons using Wilcoxon rank sum test with continuity correction
##
## data:  d$Q28 and d$Q3
##
##   1       2
## 2 0.27    -
## 3 5.9e-06 7.2e-06
##
## P value adjustment method: holm
```

```r
# Q34
kruskal.test(Q34r1~Q3, data = d)
```

```
##
##  Kruskal-Wallis rank sum test
##
## data:  Q34r1 by Q3
## Kruskal-Wallis chi-squared = 12.359, df = 2, p-value = 0.002071
```

```r
pairwise.wilcox.test(d$Q34r1,d$Q3, p.adjust.method = "holm")
```

```
##
##  Pairwise comparisons using Wilcoxon rank sum test with continuity correction
##
## data:  d$Q34r1 and d$Q3
##
##   1      2
## 2 0.1071 -
## 3 0.0014 0.0389
##
## P value adjustment method: holm
```

```r
kruskal.test(Q34r2~Q3, data = d)
```

```
##
##  Kruskal-Wallis rank sum test
##
## data:  Q34r2 by Q3
## Kruskal-Wallis chi-squared = 8.7109, df = 2, p-value = 0.01284
```

```r
pairwise.wilcox.test(d$Q34r2,d$Q3, p.adjust.method = "holm")
```

```
##
##  Pairwise comparisons using Wilcoxon rank sum test with continuity correction
##
## data:  d$Q34r2 and d$Q3
##
##   1    2
## 2 0.45 -
## 3 0.18 0.01
##
## P value adjustment method: holm
```

```r
kruskal.test(Q34r3~Q3, data = d) # N.S.
```

```
##
##  Kruskal-Wallis rank sum test
##
## data:  Q34r3 by Q3
## Kruskal-Wallis chi-squared = 2.288, df = 2, p-value = 0.3185
```

```r
# Q40
kruskal.test(IT2_1~Q3, data = d) # N.S.
```

```
##
##  Kruskal-Wallis rank sum test
##
## data:  IT2_1 by Q3
## Kruskal-Wallis chi-squared = 3.4985, df = 2, p-value = 0.1739
```

```r
kruskal.test(IT2_2~Q3, data = d) # N.S.
```

```
##
##  Kruskal-Wallis rank sum test
##
## data:  IT2_2 by Q3
## Kruskal-Wallis chi-squared = 3.258, df = 2, p-value = 0.1961
```

```r
kruskal.test(IT2_3~Q3, data = d)
```

```
##
##  Kruskal-Wallis rank sum test
##
## data:  IT2_3 by Q3
## Kruskal-Wallis chi-squared = 8.5303, df = 2, p-value = 0.01405
```

```r
pairwise.wilcox.test(d$IT2_3,d$Q3, p.adjust.method = "holm")
```

```
##
##  Pairwise comparisons using Wilcoxon rank sum test with continuity correction
##
## data:  d$IT2_3 and d$Q3
##
##   1     2
## 2 0.210 -
## 3 0.014 0.090
##
## P value adjustment method: holm
```

```r
kruskal.test(IT2_4~Q3, data = d)
```

```
##
##  Kruskal-Wallis rank sum test
##
## data:  IT2_4 by Q3
## Kruskal-Wallis chi-squared = 8.0421, df = 2, p-value = 0.01793
```

```r
pairwise.wilcox.test(d$IT2_4,d$Q3, p.adjust.method = "holm")
```

```
##
##  Pairwise comparisons using Wilcoxon rank sum test with continuity correction
##
## data:  d$IT2_4 and d$Q3
##
##   1     2
## 2 0.983 -
## 3 0.060 0.024
##
## P value adjustment method: holm
```

```r
kruskal.test(IT2_5~Q3, data = d)
```

```
##
##  Kruskal-Wallis rank sum test
##
## data:  IT2_5 by Q3
## Kruskal-Wallis chi-squared = 10.599, df = 2, p-value = 0.004995
```

```r
pairwise.wilcox.test(d$IT2_5,d$Q3, p.adjust.method = "holm")
```

```
##
##  Pairwise comparisons using Wilcoxon rank sum test with continuity correction
##
## data:  d$IT2_5 and d$Q3
##
##   1      2
## 2 0.0708 -
## 3 0.0034 0.1231
##
## P value adjustment method: holm
```

```r
kruskal.test(IT2_6~Q3, data = d)
```

```
##
##  Kruskal-Wallis rank sum test
##
## data:  IT2_6 by Q3
## Kruskal-Wallis chi-squared = 17.501, df = 2, p-value = 0.0001584
```

```r
pairwise.wilcox.test(d$IT2_6,d$Q3, p.adjust.method = "holm")
```

```
##
##  Pairwise comparisons using Wilcoxon rank sum test with continuity correction
##
## data:  d$IT2_6 and d$Q3
##
```

```
##   1       2
## 2 0.41000 -
## 3 0.01750 0.00012
##
## P value adjustment method: holm
```

```r
kruskal.test(IT2_7~Q3, data = d) # N.S.
```

```
##
##  Kruskal-Wallis rank sum test
##
## data:  IT2_7 by Q3
## Kruskal-Wallis chi-squared = 2.9289, df = 2, p-value = 0.2312
```

```r
kruskal.test(IT2_8~Q3, data = d) # N.S.
```

```
##
##  Kruskal-Wallis rank sum test
##
## data:  IT2_8 by Q3
## Kruskal-Wallis chi-squared = 4.3648, df = 2, p-value = 0.1128
```

```r
# PREFERENCE vs OWNERS PRIMARY all answers ####
# Q11
kruskal.test(Q11r1~Q15, data = d)
```

```
##
##  Kruskal-Wallis rank sum test
##
## data:  Q11r1 by Q15
## Kruskal-Wallis chi-squared = 362.16, df = 3, p-value < 2.2e-16
```

```r
pairwise.wilcox.test(d$Q11r1, d$Q15, p.adjust.method = "holm")
```

```
##
##  Pairwise comparisons using Wilcoxon rank sum test with continuity correction
##
## data:  d$Q11r1 and d$Q15
##
##   1       2       3
## 2 0.49    -       -
## 3 < 2e-16 < 2e-16 -
## 4 3.5e-10 3.6e-14 2.7e-05
##
## P value adjustment method: holm
```

```r
kruskal.test(Q11r2~Q15, data = d)
```

```
##
##  Kruskal-Wallis rank sum test
##
## data:  Q11r2 by Q15
## Kruskal-Wallis chi-squared = 268.06, df = 3, p-value < 2.2e-16
```

```r
pairwise.wilcox.test(d$Q11r2, d$Q15, p.adjust.method = "holm")
```

```
##
##  Pairwise comparisons using Wilcoxon rank sum test with continuity correction
##
```

```
## data:  d$Q11r2 and d$Q15
##
##   1       2       3
## 2 0.02484 -       -
## 3 1.2e-15 < 2e-16 -
## 4 0.00034 4.4e-12 0.00151
##
## P value adjustment method: holm
```

```
kruskal.test(Q11r3~Q15, data = d)
```

```
##
##  Kruskal-Wallis rank sum test
##
## data:  Q11r3 by Q15
## Kruskal-Wallis chi-squared = 238.66, df = 3, p-value < 2.2e-16
```

```
pairwise.wilcox.test(d$Q11r3, d$Q15, p.adjust.method = "holm")
```

```
##
##  Pairwise comparisons using Wilcoxon rank sum test with continuity correction
##
## data:  d$Q11r3 and d$Q15
##
##   1       2       3
## 2 0.06084 -       -
## 3 3.2e-13 < 2e-16 -
## 4 0.00961 1.9e-08 0.00045
##
## P value adjustment method: holm
```

```
kruskal.test(Q11r4~Q15, data = d)
```

```
##
##  Kruskal-Wallis rank sum test
##
## data:  Q11r4 by Q15
## Kruskal-Wallis chi-squared = 198.72, df = 3, p-value < 2.2e-16
```

```
pairwise.wilcox.test(d$Q11r4, d$Q15, p.adjust.method = "holm")
```

```
##
##  Pairwise comparisons using Wilcoxon rank sum test with continuity correction
##
## data:  d$Q11r4 and d$Q15
##
##   1       2       3
## 2 0.022   -       -
## 3 1.0e-08 < 2e-16 -
## 4 0.522   0.062   1.6e-11
##
## P value adjustment method: holm
```

```
kruskal.test(Q11r5~Q15, data = d)
```

```
##
##  Kruskal-Wallis rank sum test
##
```

```
## data:  Q11r5 by Q15
## Kruskal-Wallis chi-squared = 150.93, df = 3, p-value < 2.2e-16
```

```r
pairwise.wilcox.test(d$Q11r5, d$Q15, p.adjust.method = "holm")
```

```
##
##  Pairwise comparisons using Wilcoxon rank sum test with continuity correction
##
## data:  d$Q11r5 and d$Q15
##
##   1       2       3
## 2 0.205   -       -
## 3 2.5e-07 < 2e-16 -
## 4 0.042   0.205   < 2e-16
##
## P value adjustment method: holm
```

```r
kruskal.test(Q11r6~Q15, data = d)
```

```
##
##  Kruskal-Wallis rank sum test
##
## data:  Q11r6 by Q15
## Kruskal-Wallis chi-squared = 145.71, df = 3, p-value < 2.2e-16
```

```r
pairwise.wilcox.test(d$Q11r6, d$Q15, p.adjust.method = "holm")
```

```
##
##  Pairwise comparisons using Wilcoxon rank sum test with continuity correction
##
## data:  d$Q11r6 and d$Q15
##
##   1     2      3
## 2 0.137 -      -
## 3 1e-06 <2e-16 -
## 4 0.032 0.171  <2e-16
##
## P value adjustment method: holm
```

```r
kruskal.test(Q11r7~Q15, data = d)
```

```
##
##  Kruskal-Wallis rank sum test
##
## data:  Q11r7 by Q15
## Kruskal-Wallis chi-squared = 219.6, df = 3, p-value < 2.2e-16
```

```r
pairwise.wilcox.test(d$Q11r7, d$Q15, p.adjust.method = "holm")
```

```
##
##  Pairwise comparisons using Wilcoxon rank sum test with continuity correction
##
## data:  d$Q11r7 and d$Q15
##
##   1       2       3
## 2 0.18856 -       -
## 3 1.1e-13 < 2e-16 -
## 4 0.21307 0.00099 2.6e-09
```

```
##
## P value adjustment method: holm
```

```r
kruskal.test(Q11r8~Q15, data = d)
```

```
##
##  Kruskal-Wallis rank sum test
##
## data:  Q11r8 by Q15
## Kruskal-Wallis chi-squared = 108.57, df = 3, p-value < 2.2e-16
```

```r
pairwise.wilcox.test(d$Q11r8, d$Q15, p.adjust.method = "holm")
```

```
##
##  Pairwise comparisons using Wilcoxon rank sum test with continuity correction
##
## data:  d$Q11r8 and d$Q15
##
##   1       2       3
## 2 0.8006  -       -
## 3 5.3e-10 < 2e-16 -
## 4 0.0625  0.0239  0.0017
##
## P value adjustment method: holm
```

```r
# Q28
kruskal.test(Q28~Q15, data = d)
```

```
##
##  Kruskal-Wallis rank sum test
##
## data:  Q28 by Q15
## Kruskal-Wallis chi-squared = 482.9, df = 3, p-value < 2.2e-16
```

```r
pairwise.wilcox.test(d$Q28,d$Q15, p.adjust.method = "holm")
```

```
##
##  Pairwise comparisons using Wilcoxon rank sum test with continuity correction
##
## data:  d$Q28 and d$Q15
##
##   1       2       3
## 2 2.9e-14 -       -
## 3 4.4e-12 < 2e-16 -
## 4 0.74    2.8e-15 5.4e-13
##
## P value adjustment method: holm
```

```r
# Q34
kruskal.test(Q34r1~Q15, data = d)
```

```
##
##  Kruskal-Wallis rank sum test
##
## data:  Q34r1 by Q15
## Kruskal-Wallis chi-squared = 136.21, df = 3, p-value < 2.2e-16
```

```r
pairwise.wilcox.test(d$Q34r1,d$Q15, p.adjust.method = "holm")
```

```
##
##  Pairwise comparisons using Wilcoxon rank sum test with continuity correction
##
## data:  d$Q34r1 and d$Q15
##
##   1       2       3
## 2 0.0279  -       -
## 3 1.4e-09 < 2e-16 -
## 4 0.1852  0.0016  0.0016
##
## P value adjustment method: holm
```

```r
kruskal.test(Q34r2~Q15, data = d)
```

```
##
##  Kruskal-Wallis rank sum test
##
## data:  Q34r2 by Q15
## Kruskal-Wallis chi-squared = 124.99, df = 3, p-value < 2.2e-16
```

```r
pairwise.wilcox.test(d$Q34r2,d$Q15, p.adjust.method = "holm")
```

```
##
##  Pairwise comparisons using Wilcoxon rank sum test with continuity correction
##
## data:  d$Q34r2 and d$Q15
##
##   1       2       3
## 2 0.00078 -       -
## 3 3.9e-05 < 2e-16 -
## 4 0.33453 2.7e-05 0.01157
##
## P value adjustment method: holm
```

```r
kruskal.test(Q34r3~Q15, data = d)
```

```
##
##  Kruskal-Wallis rank sum test
##
## data:  Q34r3 by Q15
## Kruskal-Wallis chi-squared = 115.68, df = 3, p-value < 2.2e-16
```

```r
pairwise.wilcox.test(d$Q34r3,d$Q15, p.adjust.method = "holm")
```

```
##
##  Pairwise comparisons using Wilcoxon rank sum test with continuity correction
##
## data:  d$Q34r3 and d$Q15
##
##   1       2       3
## 2 5.2e-05 -       -
## 3 0.005   < 2e-16 -
## 4 0.540   2.3e-05 0.097
##
## P value adjustment method: holm
```

```r
# Q40
kruskal.test(IT2_1~Q15, data = d)
```

```
##
##  Kruskal-Wallis rank sum test
##
## data:  IT2_1 by Q15
## Kruskal-Wallis chi-squared = 32.461, df = 3, p-value = 4.183e-07
```

```r
pairwise.wilcox.test(d$IT2_1,d$Q15, p.adjust.method = "holm")
```

```
##
##  Pairwise comparisons using Wilcoxon rank sum test with continuity correction
##
## data:  d$IT2_1 and d$Q15
##
##   1     2       3
## 2 0.397 -       -
## 3 0.072 7.9e-08 -
## 4 0.730 0.397   0.397
##
## P value adjustment method: holm
```

```r
kruskal.test(IT2_2~Q15, data = d)
```

```
##
##  Kruskal-Wallis rank sum test
##
## data:  IT2_2 by Q15
## Kruskal-Wallis chi-squared = 93.014, df = 3, p-value < 2.2e-16
```

```r
pairwise.wilcox.test(d$IT2_2,d$Q15, p.adjust.method = "holm")
```

```
##
##  Pairwise comparisons using Wilcoxon rank sum test with continuity correction
##
## data:  d$IT2_2 and d$Q15
##
##   1       2       3
## 2 0.04078 -       -
## 3 0.00012 < 2e-16 -
## 4 0.32039 0.00332 0.04078
##
## P value adjustment method: holm
```

```r
kruskal.test(IT2_3~Q15, data = d)
```

```
##
##  Kruskal-Wallis rank sum test
##
## data:  IT2_3 by Q15
## Kruskal-Wallis chi-squared = 83.001, df = 3, p-value < 2.2e-16
```

```r
pairwise.wilcox.test(d$IT2_3,d$Q15, p.adjust.method = "holm")
```

```
##
##  Pairwise comparisons using Wilcoxon rank sum test with continuity correction
##
```

```
## data:  d$IT2_3 and d$Q15
##
##   1       2       3
## 2 0.5546  -       -
## 3 3.6e-07 < 2e-16 -
## 4 0.0832  0.0072  0.0902
##
## P value adjustment method: holm
```

```r
kruskal.test(IT2_4~Q15, data = d)
```

```
##
##  Kruskal-Wallis rank sum test
##
## data:  IT2_4 by Q15
## Kruskal-Wallis chi-squared = 59.615, df = 3, p-value = 7.104e-13
```

```r
pairwise.wilcox.test(d$IT2_4,d$Q15, p.adjust.method = "holm")
```

```
##
##  Pairwise comparisons using Wilcoxon rank sum test with continuity correction
##
## data:  d$IT2_4 and d$Q15
##
##   1      2       3
## 2 0.2099 -       -
## 3 0.0014 4.8e-13 -
## 4 0.5580 0.1053  0.0635
##
## P value adjustment method: holm
```

```r
kruskal.test(IT2_5~Q15, data = d)
```

```
##
##  Kruskal-Wallis rank sum test
##
## data:  IT2_5 by Q15
## Kruskal-Wallis chi-squared = 49.202, df = 3, p-value = 1.181e-10
```

```r
pairwise.wilcox.test(d$IT2_5,d$Q15, p.adjust.method = "holm")
```

```
##
##  Pairwise comparisons using Wilcoxon rank sum test with continuity correction
##
## data:  d$IT2_5 and d$Q15
##
##   1     2       3
## 2 0.334 -       -
## 3 0.003 4.2e-11 -
## 4 0.714 0.315   0.051
##
## P value adjustment method: holm
```

```r
kruskal.test(IT2_6~Q15, data = d)
```

```
##
##  Kruskal-Wallis rank sum test
##
```

```
## data:  IT2_6 by Q15
## Kruskal-Wallis chi-squared = 64.709, df = 3, p-value = 5.789e-14
```

```
pairwise.wilcox.test(d$IT2_6,d$Q15, p.adjust.method = "holm")
```

```
##
##  Pairwise comparisons using Wilcoxon rank sum test with continuity correction
##
## data:  d$IT2_6 and d$Q15
##
##   1      2       3
## 2 0.0927 -       -
## 3 0.0021 2.8e-14 -
## 4 0.6006 0.0683  0.0683
##
## P value adjustment method: holm
```

```
kruskal.test(IT2_7~Q15, data = d)
```

```
##
##  Kruskal-Wallis rank sum test
##
## data:  IT2_7 by Q15
## Kruskal-Wallis chi-squared = 45.835, df = 3, p-value = 6.148e-10
```

```
pairwise.wilcox.test(d$IT2_7,d$Q15, p.adjust.method = "holm")
```

```
##
##  Pairwise comparisons using Wilcoxon rank sum test with continuity correction
##
## data:  d$IT2_7 and d$Q15
##
##   1     2       3
## 2 0.220 -       -
## 3 0.013 1.9e-10 -
## 4 0.625 0.188   0.188
##
## P value adjustment method: holm
```

```
kruskal.test(IT2_8~Q15, data = d)
```

```
##
##  Kruskal-Wallis rank sum test
##
## data:  IT2_8 by Q15
## Kruskal-Wallis chi-squared = 23.509, df = 3, p-value = 3.163e-05
```

```
pairwise.wilcox.test(d$IT2_8,d$Q15, p.adjust.method = "holm")
```

```
##
##  Pairwise comparisons using Wilcoxon rank sum test with continuity correction
##
## data:  d$IT2_8 and d$Q15
##
##   1      2      3
## 2 1.0000 -      -
## 3 0.0088 0.0001 -
## 4 1.0000 1.0000 0.3256
```

```
##
## P value adjustment method: holm
```

```r
# PREFERENCE vs OWNERS PRIMARY recode ####
# Q11
kruskal.test(Q11r1~Q15_recode, data = d)
```

```
##
##  Kruskal-Wallis rank sum test
##
## data:  Q11r1 by Q15_recode
## Kruskal-Wallis chi-squared = 70.254, df = 1, p-value < 2.2e-16
```

```r
kruskal.test(Q11r2~Q15_recode, data = d)
```

```
##
##  Kruskal-Wallis rank sum test
##
## data:  Q11r2 by Q15_recode
## Kruskal-Wallis chi-squared = 23.931, df = 1, p-value = 9.983e-07
```

```r
kruskal.test(Q11r3~Q15_recode, data = d)
```

```
##
##  Kruskal-Wallis rank sum test
##
## data:  Q11r3 by Q15_recode
## Kruskal-Wallis chi-squared = 18.257, df = 1, p-value = 1.93e-05
```

```r
kruskal.test(Q11r4~Q15_recode, data = d) # N.S.
```

```
##
##  Kruskal-Wallis rank sum test
##
## data:  Q11r4 by Q15_recode
## Kruskal-Wallis chi-squared = 3.3337, df = 1, p-value = 0.06787
```

```r
kruskal.test(Q11r5~Q15_recode, data = d) # N.S.
```

```
##
##  Kruskal-Wallis rank sum test
##
## data:  Q11r5 by Q15_recode
## Kruskal-Wallis chi-squared = 2.2217, df = 1, p-value = 0.1361
```

```r
kruskal.test(Q11r6~Q15_recode, data = d) # N.S.
```

```
##
##  Kruskal-Wallis rank sum test
##
## data:  Q11r6 by Q15_recode
## Kruskal-Wallis chi-squared = 1.3124, df = 1, p-value = 0.252
```

```r
kruskal.test(Q11r7~Q15_recode, data = d)
```

```
##
##  Kruskal-Wallis rank sum test
##
## data:  Q11r7 by Q15_recode
```

```
## Kruskal-Wallis chi-squared = 15.592, df = 1, p-value = 7.86e-05
```

```r
kruskal.test(Q11r8~Q15_recode, data = d)
```

```
##
##  Kruskal-Wallis rank sum test
##
## data:  Q11r8 by Q15_recode
## Kruskal-Wallis chi-squared = 19.023, df = 1, p-value = 1.291e-05
```

```r
# Q28
kruskal.test(Q28~Q15_recode, data = d) # N.S
```

```
##
##  Kruskal-Wallis rank sum test
##
## data:  Q28 by Q15_recode
## Kruskal-Wallis chi-squared = 1.632, df = 1, p-value = 0.2014
```

```r
# Q34
kruskal.test(Q34r1~Q15_recode, data = d) # N.S.
```

```
##
##  Kruskal-Wallis rank sum test
##
## data:  Q34r1 by Q15_recode
## Kruskal-Wallis chi-squared = 1.9226, df = 1, p-value = 0.1656
```

```r
kruskal.test(Q34r2~Q15_recode, data = d)
```

```
##
##  Kruskal-Wallis rank sum test
##
## data:  Q34r2 by Q15_recode
## Kruskal-Wallis chi-squared = 4.33, df = 1, p-value = 0.03745
```

```r
kruskal.test(Q34r3~Q15_recode, data = d) # N.S.
```

```
##
##  Kruskal-Wallis rank sum test
##
## data:  Q34r3 by Q15_recode
## Kruskal-Wallis chi-squared = 0.02489, df = 1, p-value = 0.8746
```

```r
# Q40
kruskal.test(IT2_1~Q15_recode, data = d) # N.S.
```

```
##
##  Kruskal-Wallis rank sum test
##
## data:  IT2_1 by Q15_recode
## Kruskal-Wallis chi-squared = 0.2702, df = 1, p-value = 0.6032
```

```r
kruskal.test(IT2_2~Q15_recode, data = d) # N.S.
```

```
##
##  Kruskal-Wallis rank sum test
##
## data:  IT2_2 by Q15_recode
## Kruskal-Wallis chi-squared = 3.5068, df = 1, p-value = 0.06112
```

```
kruskal.test(IT2_3~Q15_recode, data = d)

##
##  Kruskal-Wallis rank sum test
##
## data:  IT2_3 by Q15_recode
## Kruskal-Wallis chi-squared = 8.4568, df = 1, p-value = 0.003637

kruskal.test(IT2_4~Q15_recode, data = d) # N.S.

##
##  Kruskal-Wallis rank sum test
##
## data:  IT2_4 by Q15_recode
## Kruskal-Wallis chi-squared = 2.973, df = 1, p-value = 0.08467

kruskal.test(IT2_5~Q15_recode, data = d) # N.S.

##
##  Kruskal-Wallis rank sum test
##
## data:  IT2_5 by Q15_recode
## Kruskal-Wallis chi-squared = 0.97042, df = 1, p-value = 0.3246

kruskal.test(IT2_6~Q15_recode, data = d) # N.S.

##
##  Kruskal-Wallis rank sum test
##
## data:  IT2_6 by Q15_recode
## Kruskal-Wallis chi-squared = 1.4423, df = 1, p-value = 0.2298

kruskal.test(IT2_7~Q15_recode, data = d) # N.S.

##
##  Kruskal-Wallis rank sum test
##
## data:  IT2_7 by Q15_recode
## Kruskal-Wallis chi-squared = 1.7028, df = 1, p-value = 0.1919

kruskal.test(IT2_8~Q15_recode, data = d)

##
##  Kruskal-Wallis rank sum test
##
## data:  IT2_8 by Q15_recode
## Kruskal-Wallis chi-squared = 3.925, df = 1, p-value = 0.04757

# PREFERENCE vs OWNERS SECONDARY recode ####
# Q11
kruskal.test(Q11r1~Q22_recode, data = d)

##
##  Kruskal-Wallis rank sum test
##
## data:  Q11r1 by Q22_recode
## Kruskal-Wallis chi-squared = 4.5671, df = 1, p-value = 0.03259
```

```r
kruskal.test(Q11r2~Q22_recode, data = d) # N.S.
```

```
##
##  Kruskal-Wallis rank sum test
##
## data:  Q11r2 by Q22_recode
## Kruskal-Wallis chi-squared = 0.39646, df = 1, p-value = 0.5289
```

```r
kruskal.test(Q11r3~Q22_recode, data = d) # N.S.
```

```
##
##  Kruskal-Wallis rank sum test
##
## data:  Q11r3 by Q22_recode
## Kruskal-Wallis chi-squared = 1.0163, df = 1, p-value = 0.3134
```

```r
kruskal.test(Q11r4~Q22_recode, data = d)
```

```
##
##  Kruskal-Wallis rank sum test
##
## data:  Q11r4 by Q22_recode
## Kruskal-Wallis chi-squared = 8.3246, df = 1, p-value = 0.003911
```

```r
kruskal.test(Q11r5~Q22_recode, data = d)
```

```
##
##  Kruskal-Wallis rank sum test
##
## data:  Q11r5 by Q22_recode
## Kruskal-Wallis chi-squared = 5.8528, df = 1, p-value = 0.01555
```

```r
kruskal.test(Q11r6~Q22_recode, data = d)
```

```
##
##  Kruskal-Wallis rank sum test
##
## data:  Q11r6 by Q22_recode
## Kruskal-Wallis chi-squared = 7.5048, df = 1, p-value = 0.006153
```

```r
kruskal.test(Q11r7~Q22_recode, data = d)
```

```
##
##  Kruskal-Wallis rank sum test
##
## data:  Q11r7 by Q22_recode
## Kruskal-Wallis chi-squared = 5.1186, df = 1, p-value = 0.02367
```

```r
kruskal.test(Q11r8~Q22_recode, data = d)
```

```
##
##  Kruskal-Wallis rank sum test
##
## data:  Q11r8 by Q22_recode
## Kruskal-Wallis chi-squared = 4.3888, df = 1, p-value = 0.03618
```

```r
# Q28
kruskal.test(Q28~Q22_recode, data = d)
```

```
##
##  Kruskal-Wallis rank sum test
##
## data:  Q28 by Q22_recode
## Kruskal-Wallis chi-squared = 4.9426, df = 1, p-value = 0.0262
```

```r
# Q34
kruskal.test(Q34r1~Q22_recode, data = d)
```

```
##
##  Kruskal-Wallis rank sum test
##
## data:  Q34r1 by Q22_recode
## Kruskal-Wallis chi-squared = 4.391, df = 1, p-value = 0.03613
```

```r
kruskal.test(Q34r2~Q22_recode, data = d) # N.S.
```

```
##
##  Kruskal-Wallis rank sum test
##
## data:  Q34r2 by Q22_recode
## Kruskal-Wallis chi-squared = 1.2281, df = 1, p-value = 0.2678
```

```r
kruskal.test(Q34r3~Q22_recode, data = d) # N.S.
```

```
##
##  Kruskal-Wallis rank sum test
##
## data:  Q34r3 by Q22_recode
## Kruskal-Wallis chi-squared = 2.7323, df = 1, p-value = 0.09833
```

```r
# Q40
kruskal.test(IT2_1~Q22_recode, data = d)
```

```
##
##  Kruskal-Wallis rank sum test
##
## data:  IT2_1 by Q22_recode
## Kruskal-Wallis chi-squared = 7.4568, df = 1, p-value = 0.00632
```

```r
kruskal.test(IT2_2~Q22_recode, data = d) # N.S.
```

```
##
##  Kruskal-Wallis rank sum test
##
## data:  IT2_2 by Q22_recode
## Kruskal-Wallis chi-squared = 0.81827, df = 1, p-value = 0.3657
```

```r
kruskal.test(IT2_3~Q22_recode, data = d)
```

```
##
##  Kruskal-Wallis rank sum test
##
## data:  IT2_3 by Q22_recode
## Kruskal-Wallis chi-squared = 5.1352, df = 1, p-value = 0.02345
```

```r
kruskal.test(IT2_4~Q22_recode, data = d) # N.S.
```

```
##
##  Kruskal-Wallis rank sum test
```

```
##
## data:  IT2_4 by Q22_recode
## Kruskal-Wallis chi-squared = 2.0015, df = 1, p-value = 0.1571
```

```r
kruskal.test(IT2_5~Q22_recode, data = d) # N.S.
```

```
##
##  Kruskal-Wallis rank sum test
##
## data:  IT2_5 by Q22_recode
## Kruskal-Wallis chi-squared = 1.3136, df = 1, p-value = 0.2517
```

```r
kruskal.test(IT2_6~Q22_recode, data = d) # N.S.
```

```
##
##  Kruskal-Wallis rank sum test
##
## data:  IT2_6 by Q22_recode
## Kruskal-Wallis chi-squared = 3.7663, df = 1, p-value = 0.0523
```

```r
kruskal.test(IT2_7~Q22_recode, data = d) # N.S.
```

```
##
##  Kruskal-Wallis rank sum test
##
## data:  IT2_7 by Q22_recode
## Kruskal-Wallis chi-squared = 3.7585, df = 1, p-value = 0.05254
```

```r
kruskal.test(IT2_8~Q22_recode, data = d) # N.S.
```

```
##
##  Kruskal-Wallis rank sum test
##
## data:  IT2_8 by Q22_recode
## Kruskal-Wallis chi-squared = 0.36522, df = 1, p-value = 0.5456
```

```r
# KNOWLEDGE vs LANGUAGE ####
# Q7
kruskal.test(Q7_recode~Q4_recode, data = d) # N.S.
```

```
##
##  Kruskal-Wallis rank sum test
##
## data:  Q7_recode by Q4_recode
## Kruskal-Wallis chi-squared = 3.5276, df = 3, p-value = 0.3172
```

```r
kruskal.test(Q7~Q4_recode, data = d)
```

```
##
##  Kruskal-Wallis rank sum test
##
## data:  Q7 by Q4_recode
## Kruskal-Wallis chi-squared = 21.76, df = 3, p-value = 7.317e-05
```

```r
pairwise.wilcox.test(d$Q7,d$Q4_recode, p.adjust.method = "holm")
```

```
##
##  Pairwise comparisons using Wilcoxon rank sum test with continuity correction
##
```

```
## data:  d$Q7 and d$Q4_recode
##
##   1       2       3
## 2 0.68244 -       -
## 3 0.00425 0.00012 -
## 4 0.78578 0.58609 0.78578
##
## P value adjustment method: holm
```

```
# Q24
kruskal.test(Q24~Q4_recode, data = d)
```

```
##
##  Kruskal-Wallis rank sum test
##
## data:  Q24 by Q4_recode
## Kruskal-Wallis chi-squared = 32.651, df = 3, p-value = 3.816e-07
```

```
pairwise.wilcox.test(d$Q24,d$Q4_recode, p.adjust.method = "holm")
```

```
##
##  Pairwise comparisons using Wilcoxon rank sum test with continuity correction
##
## data:  d$Q24 and d$Q4_recode
##
##   1       2       3
## 2 1.0     -       -
## 3 6.1e-05 4.3e-05 -
## 4 1.0     1.0     0.1
##
## P value adjustment method: holm
```

```
# Q25
kruskal.test(Q25~Q4_recode, data = d) # N.S.
```

```
##
##  Kruskal-Wallis rank sum test
##
## data:  Q25 by Q4_recode
## Kruskal-Wallis chi-squared = 5.9772, df = 3, p-value = 0.1127
```

```
# Q29
kruskal.test(Q29~Q4_recode, data = d)
```

```
##
##  Kruskal-Wallis rank sum test
##
## data:  Q29 by Q4_recode
## Kruskal-Wallis chi-squared = 37.357, df = 3, p-value = 3.868e-08
```

```
pairwise.wilcox.test(d$Q29,d$Q4_recode, p.adjust.method = "holm")
```

```
##
##  Pairwise comparisons using Wilcoxon rank sum test with continuity correction
##
## data:  d$Q29 and d$Q4_recode
##
##   1       2       3
```

```
## 2 0.00016 -       -
## 3 1.5e-08 1.00000 -
## 4 0.23322 1.00000 1.00000
##
## P value adjustment method: holm
```

```r
# Q30
kruskal.test(Q30~Q4_recode, data = d) # N.S.
```

```
##
##  Kruskal-Wallis rank sum test
##
## data:  Q30 by Q4_recode
## Kruskal-Wallis chi-squared = 5.8448, df = 3, p-value = 0.1194
```

```r
# Q35
kruskal.test(Q35~Q4_recode, data = d)
```

```
##
##  Kruskal-Wallis rank sum test
##
## data:  Q35 by Q4_recode
## Kruskal-Wallis chi-squared = 52.038, df = 3, p-value = 2.94e-11
```

```r
pairwise.wilcox.test(d$Q35,d$Q4_recode, p.adjust.method = "holm")
```

```
##
##  Pairwise comparisons using Wilcoxon rank sum test with continuity correction
##
## data:  d$Q35 and d$Q4_recode
##
##   1       2       3
## 2 2.5e-08 -       -
## 3 0.71853 7.7e-08 -
## 4 0.00017 0.51860 0.00034
##
## P value adjustment method: holm
```

```r
# KNOWLEDGE vs AGE ####
# Q7
kruskal.test(Q7_recode~RECODE_AGE, data = d)
```

```
##
##  Kruskal-Wallis rank sum test
##
## data:  Q7_recode by RECODE_AGE
## Kruskal-Wallis chi-squared = 15.128, df = 4, p-value = 0.004443
```

```r
pairwise.wilcox.test(d$Q7,d$RECODE_AGE, p.adjust.method = "holm")
```

```
##
##  Pairwise comparisons using Wilcoxon rank sum test with continuity correction
##
## data:  d$Q7 and d$RECODE_AGE
##
##   1 2 3 4
## 2 1 - - -
## 3 1 1 - -
```

```
## 4 1 1 1 -
## 5 1 1 1 1
##
## P value adjustment method: holm
```

```r
kruskal.test(Q7~RECODE_AGE, data = d) # N.S.
```

```
##
##  Kruskal-Wallis rank sum test
##
## data:  Q7 by RECODE_AGE
## Kruskal-Wallis chi-squared = 3.0713, df = 4, p-value = 0.546
```

```r
# Q24
kruskal.test(Q24~RECODE_AGE, data = d) # N.S.
```

```
##
##  Kruskal-Wallis rank sum test
##
## data:  Q24 by RECODE_AGE
## Kruskal-Wallis chi-squared = 3.7574, df = 4, p-value = 0.4398
```

```r
# Q25
kruskal.test(Q25~RECODE_AGE, data = d)
```

```
##
##  Kruskal-Wallis rank sum test
##
## data:  Q25 by RECODE_AGE
## Kruskal-Wallis chi-squared = 12.491, df = 4, p-value = 0.01405
```

```r
pairwise.wilcox.test(d$Q25,d$RECODE_AGE, p.adjust.method = "holm")
```

```
##
##  Pairwise comparisons using Wilcoxon rank sum test with continuity correction
##
## data:  d$Q25 and d$RECODE_AGE
##
##   1     2     3     4
## 2 1.000 -     -     -
## 3 1.000 1.000 -     -
## 4 1.000 1.000 0.493 -
## 5 0.095 0.069 0.012 0.493
##
## P value adjustment method: holm
```

```r
# Q29
kruskal.test(Q29~RECODE_AGE, data = d)
```

```
##
##  Kruskal-Wallis rank sum test
##
## data:  Q29 by RECODE_AGE
## Kruskal-Wallis chi-squared = 30.939, df = 4, p-value = 3.151e-06
```

```r
pairwise.wilcox.test(d$Q29,d$RECODE_AGE, p.adjust.method = "holm")
```

```
##
##  Pairwise comparisons using Wilcoxon rank sum test with continuity correction
```

```
##
## data:  d$Q29 and d$RECODE_AGE
##
##   1       2       3       4
## 2 1.00000 -       -       -
## 3 0.00280 0.00664 -       -
## 4 0.00766 0.02000 1.00000 -
## 5 0.00022 0.00061 1.00000 0.57609
##
## P value adjustment method: holm
```

```r
# Q30
kruskal.test(Q30~RECODE_AGE, data = d)
```

```
##
##  Kruskal-Wallis rank sum test
##
## data:  Q30 by RECODE_AGE
## Kruskal-Wallis chi-squared = 22.211, df = 4, p-value = 0.0001819
```

```r
pairwise.wilcox.test(d$Q30,d$RECODE_AGE, p.adjust.method = "holm")
```

```
##
##  Pairwise comparisons using Wilcoxon rank sum test with continuity correction
##
## data:  d$Q30 and d$RECODE_AGE
##
##   1       2       3       4
## 2 0.19078 -       -       -
## 3 0.22314 1.00000 -       -
## 4 1.00000 0.12033 0.19078 -
## 5 0.30907 0.00058 0.00173 0.22314
##
## P value adjustment method: holm
```

```r
# Q35
kruskal.test(Q35~RECODE_AGE, data = d)
```

```
##
##  Kruskal-Wallis rank sum test
##
## data:  Q35 by RECODE_AGE
## Kruskal-Wallis chi-squared = 17.132, df = 4, p-value = 0.001822
```

```r
pairwise.wilcox.test(d$Q35,d$RECODE_AGE, p.adjust.method = "holm")
```

```
##
##  Pairwise comparisons using Wilcoxon rank sum test with continuity correction
##
## data:  d$Q35 and d$RECODE_AGE
##
##   1       2       3       4
## 2 0.66068 -       -       -
## 3 0.88870 1.00000 -       -
## 4 0.27428 1.00000 1.00000 -
## 5 0.00056 0.14892 0.06428 0.17249
##
```

```
## P value adjustment method: holm
```

```r
# KNOWLEDGE vs EDUCATION ####
# Q7
kruskal.test(Q7_recode~Q47, data = d) # N.S.
```

```
##
##  Kruskal-Wallis rank sum test
##
## data:  Q7_recode by Q47
## Kruskal-Wallis chi-squared = 5.1052, df = 6, p-value = 0.5304
```

```r
kruskal.test(Q7~Q47, data = d)
```

```
##
##  Kruskal-Wallis rank sum test
##
## data:  Q7 by Q47
## Kruskal-Wallis chi-squared = 16.447, df = 6, p-value = 0.01154
```

```r
pairwise.wilcox.test(d$Q7,d$Q47, p.adjust.method = "holm")
```

```
##
##  Pairwise comparisons using Wilcoxon rank sum test with continuity correction
##
## data:  d$Q7 and d$Q47
##
##   1     2     3     4     5     6
## 2 1.000 -     -     -     -     -
## 3 1.000 1.000 -     -     -     -
## 4 1.000 1.000 1.000 -     -     -
## 5 1.000 1.000 1.000 1.000 -     -
## 6 1.000 1.000 0.646 0.956 0.555 -
## 7 1.000 1.000 0.074 0.262 0.087 1.000
##
## P value adjustment method: holm
```

```r
# Q24
kruskal.test(Q24~Q47, data = d) # N.S.
```

```
##
##  Kruskal-Wallis rank sum test
##
## data:  Q24 by Q47
## Kruskal-Wallis chi-squared = 8.3469, df = 6, p-value = 0.2138
```

```r
# Q25
kruskal.test(Q25~Q47, data = d) # N.S.
```

```
##
##  Kruskal-Wallis rank sum test
##
## data:  Q25 by Q47
## Kruskal-Wallis chi-squared = 5.5206, df = 6, p-value = 0.479
```

```r
# Q29
kruskal.test(Q29~Q47, data = d)
```

```
##
```

```
##  Kruskal-Wallis rank sum test
##
## data:  Q29 by Q47
## Kruskal-Wallis chi-squared = 23.593, df = 6, p-value = 0.0006204
```

```r
pairwise.wilcox.test(d$Q29,d$Q47, p.adjust.method = "holm")
```

```
##
##  Pairwise comparisons using Wilcoxon rank sum test with continuity correction
##
## data:  d$Q29 and d$Q47
##
##   1     2     3     4     5     6
## 2 0.482 -     -     -     -     -
## 3 0.421 1.000 -     -     -     -
## 4 0.182 1.000 0.182 -     -     -
## 5 0.327 1.000 1.000 1.000 -     -
## 6 0.181 0.819 0.056 1.000 1.000 -
## 7 0.208 0.819 0.023 1.000 1.000 1.000
##
## P value adjustment method: holm
```

```r
# Q30
kruskal.test(Q30~Q47, data = d)
```

```
##
##  Kruskal-Wallis rank sum test
##
## data:  Q30 by Q47
## Kruskal-Wallis chi-squared = 18.763, df = 6, p-value = 0.004583
```

```r
pairwise.wilcox.test(d$Q30,d$Q47, p.adjust.method = "holm")
```

```
##
##  Pairwise comparisons using Wilcoxon rank sum test with continuity correction
##
## data:  d$Q30 and d$Q47
##
##   1      2      3      4      5      6
## 2 1.0000 -      -      -      -      -
## 3 1.0000 0.0236 -      -      -      -
## 4 1.0000 0.1690 1.0000 -      -      -
## 5 1.0000 0.0326 1.0000 1.0000 -      -
## 6 1.0000 0.0048 1.0000 0.6531 1.0000 -
## 7 1.0000 0.0100 1.0000 1.0000 1.0000 1.0000
##
## P value adjustment method: holm
```

```r
# Q35
kruskal.test(Q35~Q47, data = d)
```

```
##
##  Kruskal-Wallis rank sum test
##
## data:  Q35 by Q47
## Kruskal-Wallis chi-squared = 67.45, df = 6, p-value = 1.361e-12
```

```r
pairwise.wilcox.test(d$Q35,d$Q47, p.adjust.method = "holm")
```

```
##
##  Pairwise comparisons using Wilcoxon rank sum test with continuity correction
##
## data:  d$Q35 and d$Q47
##
##   1       2       3       4       5       6
## 2 1.00000 -       -       -       -       -
## 3 0.81196 9.2e-05 -       -       -       -
## 4 0.81196 0.00035 1.00000 -       -       -
## 5 0.39208 5.9e-08 0.08433 0.20741 -       -
## 6 0.40614 3.5e-07 0.13512 0.23139 1.00000 -
## 7 0.21188 7.2e-11 1.9e-05 0.00071 0.51956 0.81196
##
## P value adjustment method: holm
```

```r
# KNOWLEDGE vs RESIDENCE ####
# Q7
kruskal.test(Q7_recode~Q3, data = d) # N.S.
```

```
##
##  Kruskal-Wallis rank sum test
##
## data:  Q7_recode by Q3
## Kruskal-Wallis chi-squared = 0.71761, df = 2, p-value = 0.6985
```

```r
kruskal.test(Q7~Q3, data = d) # N.S.
```

```
##
##  Kruskal-Wallis rank sum test
##
## data:  Q7 by Q3
## Kruskal-Wallis chi-squared = 4.1608, df = 2, p-value = 0.1249
```

```r
# Q24
kruskal.test(Q24~Q3, data = d) # N.S.
```

```
##
##  Kruskal-Wallis rank sum test
##
## data:  Q24 by Q3
## Kruskal-Wallis chi-squared = 3.7296, df = 2, p-value = 0.1549
```

```r
# Q25
kruskal.test(Q25~Q3, data = d) # N.S.
```

```
##
##  Kruskal-Wallis rank sum test
##
## data:  Q25 by Q3
## Kruskal-Wallis chi-squared = 2.9756, df = 2, p-value = 0.2259
```

```r
# Q29
kruskal.test(Q29~Q3, data = d) # N.S.
```

```
##
##  Kruskal-Wallis rank sum test
```

```
##
## data:  Q29 by Q3
## Kruskal-Wallis chi-squared = 5.1378, df = 2, p-value = 0.07662
```

```r
# Q30
kruskal.test(Q30~Q3, data = d)
```

```
##
##  Kruskal-Wallis rank sum test
##
## data:  Q30 by Q3
## Kruskal-Wallis chi-squared = 10.818, df = 2, p-value = 0.004475
```

```r
pairwise.wilcox.test(d$Q30,d$Q3, p.adjust.method = "holm")
```

```
##
##  Pairwise comparisons using Wilcoxon rank sum test with continuity correction
##
## data:  d$Q30 and d$Q3
##
##   1      2
## 2 0.3447 -
## 3 0.0079 0.0179
##
## P value adjustment method: holm
```

```r
# Q35
kruskal.test(Q35~Q3, data = d)
```

```
##
##  Kruskal-Wallis rank sum test
##
## data:  Q35 by Q3
## Kruskal-Wallis chi-squared = 6.3835, df = 2, p-value = 0.0411
```

```r
pairwise.wilcox.test(d$Q35,d$Q3, p.adjust.method = "holm")
```

```
##
##  Pairwise comparisons using Wilcoxon rank sum test with continuity correction
##
## data:  d$Q35 and d$Q3
##
##   1     2
## 2 0.989 -
## 3 0.099 0.056
##
## P value adjustment method: holm
```

```r
# KNOWLEDGE vs OWNERS PRIMARY all answers ####
# Q7
kruskal.test(Q7_recode~Q15, data = d) # N.S.
```

```
##
##  Kruskal-Wallis rank sum test
##
## data:  Q7_recode by Q15
## Kruskal-Wallis chi-squared = 1.2928, df = 3, p-value = 0.7308
```

```r
kruskal.test(Q7~Q15, data = d) # N.S.
```

```
##
##  Kruskal-Wallis rank sum test
##
## data:  Q7 by Q15
## Kruskal-Wallis chi-squared = 4.7919, df = 3, p-value = 0.1877
```

```r
# Q24
kruskal.test(Q24~Q15, data = d)
```

```
##
##  Kruskal-Wallis rank sum test
##
## data:  Q24 by Q15
## Kruskal-Wallis chi-squared = 22.815, df = 3, p-value = 4.413e-05
```

```r
pairwise.wilcox.test(d$Q24,d$Q15, p.adjust.method = "holm")
```

```
##
##  Pairwise comparisons using Wilcoxon rank sum test with continuity correction
##
## data:  d$Q24 and d$Q15
##
##   1     2     3
## 2 0.011 -     -
## 3 1e-05 0.197 -
## 4 0.074 0.866 0.679
##
## P value adjustment method: holm
```

```r
# Q25
kruskal.test(Q25~Q15, data = d)
```

```
##
##  Kruskal-Wallis rank sum test
##
## data:  Q25 by Q15
## Kruskal-Wallis chi-squared = 12.873, df = 3, p-value = 0.004921
```

```r
pairwise.wilcox.test(d$Q25,d$Q15, p.adjust.method = "holm")
```

```
##
##  Pairwise comparisons using Wilcoxon rank sum test with continuity correction
##
## data:  d$Q25 and d$Q15
##
##   1     2     3
## 2 0.968 -     -
## 3 0.034 0.051 -
## 4 0.087 0.113 0.968
##
## P value adjustment method: holm
```

```r
Q29
```

```
## Error in eval(expr, envir, enclos): object 'Q29' not found
```

```r
kruskal.test(Q29~Q15, data = d)
```

```
##
##  Kruskal-Wallis rank sum test
##
## data:  Q29 by Q15
## Kruskal-Wallis chi-squared = 21.933, df = 3, p-value = 6.736e-05
```

```r
pairwise.wilcox.test(d$Q29,d$Q15, p.adjust.method = "holm")
```

```
##
##  Pairwise comparisons using Wilcoxon rank sum test with continuity correction
##
## data:  d$Q29 and d$Q15
##
##   1       2      3
## 2 0.0112  -      -
## 3 0.0110  0.6345 -
## 4 3.2e-05 0.0046 0.0110
##
## P value adjustment method: holm
```

```r
# Q30
kruskal.test(Q30~Q15, data = d)
```

```
##
##  Kruskal-Wallis rank sum test
##
## data:  Q30 by Q15
## Kruskal-Wallis chi-squared = 279.71, df = 3, p-value < 2.2e-16
```

```r
pairwise.wilcox.test(d$Q30,d$Q15, p.adjust.method = "holm")
```

```
##
##  Pairwise comparisons using Wilcoxon rank sum test with continuity correction
##
## data:  d$Q30 and d$Q15
##
##   1       2       3
## 2 5.1e-10 -       -
## 3 6.2e-05 < 2e-16 -
## 4 0.11    3.3e-05 2.5e-08
##
## P value adjustment method: holm
```

```r
# Q35
kruskal.test(Q35~Q15, data = d)
```

```
##
##  Kruskal-Wallis rank sum test
##
## data:  Q35 by Q15
## Kruskal-Wallis chi-squared = 68.417, df = 3, p-value = 9.316e-15
```

```r
pairwise.wilcox.test(d$Q35,d$Q15, p.adjust.method = "holm")
```

```
##
##  Pairwise comparisons using Wilcoxon rank sum test with continuity correction
```

```
##
## data:  d$Q35 and d$Q15
##
##   1       2       3
## 2 4.5e-06 -       -
## 3 0.13854 4.1e-06 -
## 4 2.0e-10 0.00049 3.8e-10
##
## P value adjustment method: holm
```

```r
# KNOWLEDGE vs OWNERS PRIMARY recode ####
# Q7
kruskal.test(Q7_recode~Q15_recode, data = d) # N.S.
```

```
##
##  Kruskal-Wallis rank sum test
##
## data:  Q7_recode by Q15_recode
## Kruskal-Wallis chi-squared = 0.11588, df = 1, p-value = 0.7335
```

```r
kruskal.test(Q7~Q15_recode, data = d) # N.S.
```

```
##
##  Kruskal-Wallis rank sum test
##
## data:  Q7 by Q15_recode
## Kruskal-Wallis chi-squared = 2.6507, df = 1, p-value = 0.1035
```

```r
# Q24
kruskal.test(Q24~Q15_recode, data = d)
```

```
##
##  Kruskal-Wallis rank sum test
##
## data:  Q24 by Q15_recode
## Kruskal-Wallis chi-squared = 19.542, df = 1, p-value = 9.84e-06
```

```r
# Q25
kruskal.test(Q25~Q15_recode, data = d)
```

```
##
##  Kruskal-Wallis rank sum test
##
## data:  Q25 by Q15_recode
## Kruskal-Wallis chi-squared = 5.5381, df = 1, p-value = 0.01861
```

```r
# Q29
kruskal.test(Q29~Q15_recode, data = d)
```

```
##
##  Kruskal-Wallis rank sum test
##
## data:  Q29 by Q15_recode
## Kruskal-Wallis chi-squared = 14.041, df = 1, p-value = 0.0001789
```

```r
# Q30
kruskal.test(Q30~Q15_recode, data = d) # N.S.
```

```
##
```

```
##  Kruskal-Wallis rank sum test
##
## data:  Q30 by Q15_recode
## Kruskal-Wallis chi-squared = 0.40292, df = 1, p-value = 0.5256
```

```
# Q35
kruskal.test(Q35~Q15_recode, data = d)
```

```
##
##  Kruskal-Wallis rank sum test
##
## data:  Q35 by Q15_recode
## Kruskal-Wallis chi-squared = 20.487, df = 1, p-value = 6.003e-06
```

```
# KNOWLEDGE vs OWNERS SECONDARY recode ####
# Q7
kruskal.test(Q7_recode~Q22_recode, data = d) # N.S.
```

```
##
##  Kruskal-Wallis rank sum test
##
## data:  Q7_recode by Q22_recode
## Kruskal-Wallis chi-squared = 1.8203, df = 1, p-value = 0.1773
```

```
kruskal.test(Q7~Q22_recode, data = d) # N.S.
```

```
##
##  Kruskal-Wallis rank sum test
##
## data:  Q7 by Q22_recode
## Kruskal-Wallis chi-squared = 0.12805, df = 1, p-value = 0.7205
```

```
# Q24
kruskal.test(Q24~Q22_recode, data = d)
```

```
##
##  Kruskal-Wallis rank sum test
##
## data:  Q24 by Q22_recode
## Kruskal-Wallis chi-squared = 20.444, df = 1, p-value = 6.14e-06
```

```
# Q25
kruskal.test(Q25~Q22_recode, data = d)
```

```
##
##  Kruskal-Wallis rank sum test
##
## data:  Q25 by Q22_recode
## Kruskal-Wallis chi-squared = 27.812, df = 1, p-value = 1.337e-07
```

```
# Q29
kruskal.test(Q29~Q22_recode, data = d)
```

```
##
##  Kruskal-Wallis rank sum test
##
## data:  Q29 by Q22_recode
## Kruskal-Wallis chi-squared = 11.495, df = 1, p-value = 0.0006978
```

```r
# Q30
kruskal.test(Q30~Q22_recode, data = d) # N.S.
```

```
##
##  Kruskal-Wallis rank sum test
##
## data:  Q30 by Q22_recode
## Kruskal-Wallis chi-squared = 2.3328, df = 1, p-value = 0.1267
```

```r
# Q35
kruskal.test(Q35~Q22_recode, data = d)
```

```
##
##  Kruskal-Wallis rank sum test
##
## data:  Q35 by Q22_recode
## Kruskal-Wallis chi-squared = 6.2103, df = 1, p-value = 0.0127
```

```r
# MANAGEMENT vs LANGUAGE ####
# Q39
kruskal.test(Q39~Q4_recode, data = d)
```

```
##
##  Kruskal-Wallis rank sum test
##
## data:  Q39 by Q4_recode
## Kruskal-Wallis chi-squared = 10.934, df = 3, p-value = 0.01209
```

```r
pairwise.wilcox.test(d$Q39, d$Q4_recode, p.adjust.method = "holm")
```

```
##
##  Pairwise comparisons using Wilcoxon rank sum test with continuity correction
##
## data:  d$Q39 and d$Q4_recode
##
##   1     2     3
## 2 0.079 -     -
## 3 0.011 1.000 -
## 4 1.000 1.000 1.000
##
## P value adjustment method: holm
```

```r
# Q41
kruskal.test(Q41~Q4_recode, data = d)
```

```
##
##  Kruskal-Wallis rank sum test
##
## data:  Q41 by Q4_recode
## Kruskal-Wallis chi-squared = 12.423, df = 3, p-value = 0.006066
```

```r
pairwise.wilcox.test(d$Q41, d$Q4_recode, p.adjust.method = "holm")
```

```
##
##  Pairwise comparisons using Wilcoxon rank sum test with continuity correction
##
## data:  d$Q41 and d$Q4_recode
##
```

```
##   1     2     3    
## 2 0.752 -     -    
## 3 0.003 0.752 -    
## 4 0.752 1.000 1.000
## 
## P value adjustment method: holm
```

```r
# Q42
kruskal.test(Q42~Q4_recode, data = d)
```

```
## 
##  Kruskal-Wallis rank sum test
## 
## data:  Q42 by Q4_recode
## Kruskal-Wallis chi-squared = 9.7026, df = 3, p-value = 0.02127
```

```r
pairwise.wilcox.test(d$Q42,d$Q4_recode, p.adjust.method = "holm")
```

```
## 
##  Pairwise comparisons using Wilcoxon rank sum test with continuity correction 
## 
## data:  d$Q42 and d$Q4_recode 
## 
##   1     2     3    
## 2 0.012 -     -    
## 3 0.701 0.188 -    
## 4 1.000 1.000 1.000
## 
## P value adjustment method: holm
```

```r
# Q43
kruskal.test(Q43~Q4_recode, data = d) # N.S.
```

```
## 
##  Kruskal-Wallis rank sum test
## 
## data:  Q43 by Q4_recode
## Kruskal-Wallis chi-squared = 5.5253, df = 3, p-value = 0.1371
```

```r
# Q44r1
kruskal.test(Q44r1~Q4_recode, data = d)
```

```
## 
##  Kruskal-Wallis rank sum test
## 
## data:  Q44r1 by Q4_recode
## Kruskal-Wallis chi-squared = 21.513, df = 3, p-value = 8.236e-05
```

```r
pairwise.wilcox.test(d$Q44r1,d$Q4_recode, p.adjust.method = "holm")
```

```
## 
##  Pairwise comparisons using Wilcoxon rank sum test with continuity correction 
## 
## data:  d$Q44r1 and d$Q4_recode 
## 
##   1       2     3    
## 2 0.031   -     -    
## 3 2.8e-05 0.795 -
```

```
## 4 0.441   1.000 1.000
##
## P value adjustment method: holm
```

```r
# Q44r2
kruskal.test(Q44r2~Q4_recode, data = d) # N.S.
```

```
##
##  Kruskal-Wallis rank sum test
##
## data:  Q44r2 by Q4_recode
## Kruskal-Wallis chi-squared = 6.541, df = 3, p-value = 0.08806
```

```r
# Q44r3
kruskal.test(Q44r3~Q4_recode, data = d) # N.S.
```

```
##
##  Kruskal-Wallis rank sum test
##
## data:  Q44r3 by Q4_recode
## Kruskal-Wallis chi-squared = 2.4128, df = 3, p-value = 0.4912
```

```r
# Q44r4
kruskal.test(Q44r4~Q4_recode, data = d)
```

```
##
##  Kruskal-Wallis rank sum test
##
## data:  Q44r4 by Q4_recode
## Kruskal-Wallis chi-squared = 12.872, df = 3, p-value = 0.004922
```

```r
pairwise.wilcox.test(d$Q44r4,d$Q4_recode, p.adjust.method = "holm")
```

```
##
##  Pairwise comparisons using Wilcoxon rank sum test with continuity correction
##
## data:  d$Q44r4 and d$Q4_recode
##
##   1     2     3
## 2 0.016 -     -
## 3 0.016 1.000 -
## 4 1.000 0.533 0.576
##
## P value adjustment method: holm
```

```r
# Q44r5
kruskal.test(Q44r5~Q4_recode, data = d)
```

```
##
##  Kruskal-Wallis rank sum test
##
## data:  Q44r5 by Q4_recode
## Kruskal-Wallis chi-squared = 29.325, df = 3, p-value = 1.913e-06
```

```r
pairwise.wilcox.test(d$Q44r5,d$Q4_recode, p.adjust.method = "holm")
```

```
##
##  Pairwise comparisons using Wilcoxon rank sum test with continuity correction
##
```

```
## data:  d$Q44r5 and d$Q4_recode
##
##   1      2      3
## 2 6e-07  -      -
## 3 0.0475 0.0017 -
## 4 1.0000 0.0685 1.0000
##
## P value adjustment method: holm
```

```r
# Q44r6
kruskal.test(Q44r6~Q4_recode, data = d)
```

```
##
##  Kruskal-Wallis rank sum test
##
## data:  Q44r6 by Q4_recode
## Kruskal-Wallis chi-squared = 30.252, df = 3, p-value = 1.222e-06
```

```r
pairwise.wilcox.test(d$Q44r6,d$Q4_recode, p.adjust.method = "holm")
```

```
##
##  Pairwise comparisons using Wilcoxon rank sum test with continuity correction
##
## data:  d$Q44r6 and d$Q4_recode
##
##   1       2      3
## 2 3.8e-07 -      -
## 3 0.0036  0.0203 -
## 4 0.4038  0.3895 0.8566
##
## P value adjustment method: holm
```

```r
# MANAGEMENT vs AGE ####
# Q39
kruskal.test(Q39~RECODE_AGE, data = d)
```

```
##
##  Kruskal-Wallis rank sum test
##
## data:  Q39 by RECODE_AGE
## Kruskal-Wallis chi-squared = 18.984, df = 4, p-value = 0.0007915
```

```r
pairwise.wilcox.test(d$Q39, d$RECODE_AGE, p.adjust.method = "holm")
```

```
##
##  Pairwise comparisons using Wilcoxon rank sum test with continuity correction
##
## data:  d$Q39 and d$RECODE_AGE
##
##   1       2       3       4
## 2 1.00000 -       -       -
## 3 1.00000 1.00000 -       -
## 4 1.00000 1.00000 1.00000 -
## 5 0.00062 0.00667 0.05786 0.02296
##
## P value adjustment method: holm
```

```r
# Q41
kruskal.test(Q41~RECODE_AGE, data = d)
```

```
##
##  Kruskal-Wallis rank sum test
##
## data:  Q41 by RECODE_AGE
## Kruskal-Wallis chi-squared = 32.34, df = 4, p-value = 1.63e-06
```

```r
pairwise.wilcox.test(d$Q41, d$RECODE_AGE, p.adjust.method = "holm")
```

```
##
##  Pairwise comparisons using Wilcoxon rank sum test with continuity correction
##
## data:  d$Q41 and d$RECODE_AGE
##
##   1       2       3       4
## 2 0.50157 -       -       -
## 3 0.00032 0.07946 -       -
## 4 0.00410 0.37435 0.50157 -
## 5 2.3e-06 0.00699 0.58543 0.21366
##
## P value adjustment method: holm
```

```r
# Q42
kruskal.test(Q42~RECODE_AGE, data = d)
```

```
##
##  Kruskal-Wallis rank sum test
##
## data:  Q42 by RECODE_AGE
## Kruskal-Wallis chi-squared = 53.731, df = 4, p-value = 5.992e-11
```

```r
pairwise.wilcox.test(d$Q42,d$RECODE_AGE, p.adjust.method = "holm")
```

```
##
##  Pairwise comparisons using Wilcoxon rank sum test with continuity correction
##
## data:  d$Q42 and d$RECODE_AGE
##
##   1       2       3       4
## 2 0.3332  -       -       -
## 3 0.8414  0.3332  -       -
## 4 0.0994  0.0018  0.1647  -
## 5 6.0e-07 7.2e-10 4.9e-06 0.0100
##
## P value adjustment method: holm
```

```r
# Q43
kruskal.test(Q43~RECODE_AGE, data = d)
```

```
##
##  Kruskal-Wallis rank sum test
##
## data:  Q43 by RECODE_AGE
## Kruskal-Wallis chi-squared = 48.895, df = 4, p-value = 6.142e-10
```

```r
pairwise.wilcox.test(d$Q43,d$RECODE_AGE, p.adjust.method = "holm")
```

```
##
##  Pairwise comparisons using Wilcoxon rank sum test with continuity correction
##
## data:  d$Q43 and d$RECODE_AGE
##
##   1       2       3       4
## 2 0.5693  -       -       -
## 3 0.8684  0.8684  -       -
## 4 0.5693  0.0250  0.2915  -
## 5 4.1e-06 3.9e-09 1.9e-06 0.0011
##
## P value adjustment method: holm
```

```r
# Q44r1
kruskal.test(Q44r1~RECODE_AGE, data = d)
```

```
##
##  Kruskal-Wallis rank sum test
##
## data:  Q44r1 by RECODE_AGE
## Kruskal-Wallis chi-squared = 16.121, df = 4, p-value = 0.002861
```

```r
pairwise.wilcox.test(d$Q44r1,d$RECODE_AGE, p.adjust.method = "holm")
```

```
##
##  Pairwise comparisons using Wilcoxon rank sum test with continuity correction
##
## data:  d$Q44r1 and d$RECODE_AGE
##
##   1      2      3      4
## 2 0.3512 -      -      -
## 3 0.1104 0.9419 -      -
## 4 0.9419 0.9419 0.3373 -
## 5 0.9419 0.0386 0.0043 0.3314
##
## P value adjustment method: holm
```

```r
# Q44r2
kruskal.test(Q44r2~RECODE_AGE, data = d)
```

```
##
##  Kruskal-Wallis rank sum test
##
## data:  Q44r2 by RECODE_AGE
## Kruskal-Wallis chi-squared = 23.359, df = 4, p-value = 0.0001073
```

```r
pairwise.wilcox.test(d$Q44r2, d$RECODE_AGE, p.adjust.method = "holm")
```

```
##
##  Pairwise comparisons using Wilcoxon rank sum test with continuity correction
##
## data:  d$Q44r2 and d$RECODE_AGE
##
##   1      2      3      4
## 2 1.0000 -      -      -
```

```
## 3 1.0000 1.0000 -      -
## 4 0.0453 0.0031 0.0453 -
## 5 0.0438 0.0031 0.0453 1.0000
##
## P value adjustment method: holm
```

```r
# Q44r3
kruskal.test(Q44r3~RECODE_AGE, data = d)
```

```
##
##  Kruskal-Wallis rank sum test
##
## data:  Q44r3 by RECODE_AGE
## Kruskal-Wallis chi-squared = 19.659, df = 4, p-value = 0.0005831
```

```r
pairwise.wilcox.test(d$Q44r3,d$RECODE_AGE, p.adjust.method = "holm")
```

```
##
##  Pairwise comparisons using Wilcoxon rank sum test with continuity correction
##
## data:  d$Q44r3 and d$RECODE_AGE
##
##   1      2      3      4
## 2 1.0000 -      -      -
## 3 1.0000 1.0000 -      -
## 4 0.2287 0.5761 0.3109 -
## 5 0.0020 0.0212 0.0053 0.4375
##
## P value adjustment method: holm
```

```r
# Q44r4
kruskal.test(Q44r4~RECODE_AGE, data = d)
```

```
##
##  Kruskal-Wallis rank sum test
##
## data:  Q44r4 by RECODE_AGE
## Kruskal-Wallis chi-squared = 47.612, df = 4, p-value = 1.137e-09
```

```r
pairwise.wilcox.test(d$Q44r4,d$RECODE_AGE, p.adjust.method = "holm")
```

```
##
##  Pairwise comparisons using Wilcoxon rank sum test with continuity correction
##
## data:  d$Q44r4 and d$RECODE_AGE
##
##   1       2       3       4
## 2 0.16485 -       -       -
## 3 0.08254 0.55330 -       -
## 4 3.1e-05 0.04516 0.15498 -
## 5 3.2e-09 0.00012 0.00119 0.16485
##
## P value adjustment method: holm
```

```r
# Q44r5
kruskal.test(Q44r5~RECODE_AGE, data = d)
```

```
##
```

```
##  Kruskal-Wallis rank sum test
##
## data:  Q44r5 by RECODE_AGE
## Kruskal-Wallis chi-squared = 66.217, df = 4, p-value = 1.426e-13
```

```r
pairwise.wilcox.test(d$Q44r5,d$RECODE_AGE, p.adjust.method = "holm")
```

```
##
##  Pairwise comparisons using Wilcoxon rank sum test with continuity correction
##
## data:  d$Q44r5 and d$RECODE_AGE
##
##   1       2       3       4
## 2 0.1517  -       -       -
## 3 0.0845  0.5149  -       -
## 4 3.1e-05 0.0210  0.0937  -
## 5 3.1e-12 1.0e-07 4.2e-06 0.0052
##
## P value adjustment method: holm
```

```r
# Q44r6
kruskal.test(Q44r6~RECODE_AGE, data = d)
```

```
##
##  Kruskal-Wallis rank sum test
##
## data:  Q44r6 by RECODE_AGE
## Kruskal-Wallis chi-squared = 67.09, df = 4, p-value = 9.333e-14
```

```r
pairwise.wilcox.test(d$Q44r6,d$RECODE_AGE, p.adjust.method = "holm")
```

```
##
##  Pairwise comparisons using Wilcoxon rank sum test with continuity correction
##
## data:  d$Q44r6 and d$RECODE_AGE
##
##   1       2       3       4
## 2 0.2764  -       -       -
## 3 0.0240  0.2764  -       -
## 4 5.6e-05 0.0067  0.2764  -
## 5 3.9e-12 8.5e-09 2.7e-05 0.0067
##
## P value adjustment method: holm
```

```r
# MANAGEMENT vs EDUCATION ####
# Q39
kruskal.test(Q39~Q47, data = d)
```

```
##
##  Kruskal-Wallis rank sum test
##
## data:  Q39 by Q47
## Kruskal-Wallis chi-squared = 19.205, df = 6, p-value = 0.003831
```

```r
pairwise.wilcox.test(d$Q39, d$Q47, p.adjust.method = "holm")
```

```
##
##  Pairwise comparisons using Wilcoxon rank sum test with continuity correction
```

```
##
## data:  d$Q39 and d$Q47
##
##   1     2     3     4     5     6
## 2 1.000 -     -     -     -     -
## 3 1.000 1.000 -     -     -     -
## 4 1.000 1.000 1.000 -     -     -
## 5 1.000 0.878 0.066 1.000 -     -
## 6 1.000 1.000 0.233 1.000 1.000 -
## 7 1.000 0.648 0.017 1.000 1.000 1.000
##
## P value adjustment method: holm
```

```
# Q41
kruskal.test(Q41~Q47, data = d)
```

```
##
##  Kruskal-Wallis rank sum test
##
## data:  Q41 by Q47
## Kruskal-Wallis chi-squared = 32.063, df = 6, p-value = 1.587e-05
```

```
pairwise.wilcox.test(d$Q41, d$Q47, p.adjust.method = "holm")
```

```
##
##  Pairwise comparisons using Wilcoxon rank sum test with continuity correction
##
## data:  d$Q41 and d$Q47
##
##   1      2      3      4      5      6
## 2 1.0000 -      -      -      -      -
## 3 1.0000 0.5175 -      -      -      -
## 4 1.0000 0.0022 0.0022 -      -      -
## 5 1.0000 0.2467 1.0000 0.1500 -      -
## 6 1.0000 0.0386 0.2558 1.0000 1.0000 -
## 7 1.0000 0.0081 0.0195 1.0000 0.7265 1.0000
##
## P value adjustment method: holm
```

```
# Q42
kruskal.test(Q42~Q47, data = d) # N.S.
```

```
##
##  Kruskal-Wallis rank sum test
##
## data:  Q42 by Q47
## Kruskal-Wallis chi-squared = 4.932, df = 6, p-value = 0.5526
```

```
# Q43
kruskal.test(Q43~Q47, data = d)
```

```
##
##  Kruskal-Wallis rank sum test
##
## data:  Q43 by Q47
## Kruskal-Wallis chi-squared = 20.564, df = 6, p-value = 0.002196
```

```r
pairwise.wilcox.test(d$Q43,d$Q47, p.adjust.method = "holm")
```

```
##
##  Pairwise comparisons using Wilcoxon rank sum test with continuity correction
##
## data:  d$Q43 and d$Q47
##
##   1     2     3     4     5     6
## 2 1.000 -     -     -     -     -
## 3 1.000 0.585 -     -     -     -
## 4 1.000 0.835 1.000 -     -     -
## 5 1.000 0.835 1.000 1.000 -     -
## 6 0.954 0.026 0.302 0.327 0.342 -
## 7 0.954 0.030 0.405 0.439 0.459 1.000
##
## P value adjustment method: holm
```

```r
# Q44r1
kruskal.test(Q44r1~Q47, data = d)
```

```
##
##  Kruskal-Wallis rank sum test
##
## data:  Q44r1 by Q47
## Kruskal-Wallis chi-squared = 14.635, df = 6, p-value = 0.0233
```

```r
pairwise.wilcox.test(d$Q44r1,d$Q47, p.adjust.method = "holm")
```

```
##
##  Pairwise comparisons using Wilcoxon rank sum test with continuity correction
##
## data:  d$Q44r1 and d$Q47
##
##   1     2     3     4     5     6
## 2 1.000 -     -     -     -     -
## 3 1.000 1.000 -     -     -     -
## 4 1.000 1.000 1.000 -     -     -
## 5 1.000 1.000 1.000 1.000 -     -
## 6 1.000 1.000 1.000 1.000 1.000 -
## 7 1.000 0.735 0.072 1.000 0.772 1.000
##
## P value adjustment method: holm
```

```r
# Q44r2
kruskal.test(Q44r2~Q47, data = d) # N.S.
```

```
##
##  Kruskal-Wallis rank sum test
##
## data:  Q44r2 by Q47
## Kruskal-Wallis chi-squared = 11.39, df = 6, p-value = 0.07705
```

```r
# Q44r3
kruskal.test(Q44r3~Q47, data = d)
```

```
##
##  Kruskal-Wallis rank sum test
```

```
##
## data:  Q44r3 by Q47
## Kruskal-Wallis chi-squared = 17.64, df = 6, p-value = 0.007198
```

```
pairwise.wilcox.test(d$Q44r3,d$Q47, p.adjust.method = "holm")
```

```
##
##  Pairwise comparisons using Wilcoxon rank sum test with continuity correction
##
## data:  d$Q44r3 and d$Q47
##
##   1    2    3    4    5    6
## 2 1.00 -    -    -    -    -
## 3 1.00 1.00 -    -    -    -
## 4 1.00 1.00 1.00 -    -    -
## 5 0.84 0.21 0.27 1.00 -    -
## 6 0.84 0.27 0.39 1.00 1.00 -
## 7 0.84 0.39 0.84 1.00 1.00 1.00
##
## P value adjustment method: holm
```

```
# Q44r4
kruskal.test(Q44r4~Q47, data = d) # N.S.
```

```
##
##  Kruskal-Wallis rank sum test
##
## data:  Q44r4 by Q47
## Kruskal-Wallis chi-squared = 6.9643, df = 6, p-value = 0.3242
```

```
# Q44r5
kruskal.test(Q44r5~Q47, data = d)
```

```
##
##  Kruskal-Wallis rank sum test
##
## data:  Q44r5 by Q47
## Kruskal-Wallis chi-squared = 17.682, df = 6, p-value = 0.007078
```

```
pairwise.wilcox.test(d$Q44r5,d$Q47, p.adjust.method = "holm")
```

```
##
##  Pairwise comparisons using Wilcoxon rank sum test with continuity correction
##
## data:  d$Q44r5 and d$Q47
##
##   1      2      3      4      5      6
## 2 1.0000 -      -      -      -      -
## 3 1.0000 1.0000 -      -      -      -
## 4 1.0000 1.0000 0.0276 -      -      -
## 5 1.0000 1.0000 1.0000 0.1242 -      -
## 6 1.0000 1.0000 1.0000 0.0019 1.0000 -
## 7 1.0000 1.0000 1.0000 0.7910 1.0000 0.4690
##
## P value adjustment method: holm
```

```r
# Q44r6
kruskal.test(Q44r6~Q47, data = d)
```

```
##
##  Kruskal-Wallis rank sum test
##
## data:  Q44r6 by Q47
## Kruskal-Wallis chi-squared = 16.067, df = 6, p-value = 0.0134
```

```r
pairwise.wilcox.test(d$Q44r6,d$Q47, p.adjust.method = "holm")
```

```
##
##  Pairwise comparisons using Wilcoxon rank sum test with continuity correction
##
## data:  d$Q44r6 and d$Q47
##
##   1     2     3     4     5     6
## 2 1.000 -     -     -     -     -
## 3 1.000 1.000 -     -     -     -
## 4 1.000 1.000 0.014 -     -     -
## 5 1.000 1.000 1.000 0.128 -     -
## 6 1.000 1.000 1.000 0.281 1.000 -
## 7 1.000 1.000 0.231 1.000 0.984 1.000
##
## P value adjustment method: holm
```

```r
# MANAGEMENT vs RESIDENCE ####
# Q39
kruskal.test(Q39~Q3, data = d) # N.S.
```

```
##
##  Kruskal-Wallis rank sum test
##
## data:  Q39 by Q3
## Kruskal-Wallis chi-squared = 0.022915, df = 2, p-value = 0.9886
```

```r
# Q41
kruskal.test(Q41~Q3, data = d) # N.S.
```

```
##
##  Kruskal-Wallis rank sum test
##
## data:  Q41 by Q3
## Kruskal-Wallis chi-squared = 2.0157, df = 2, p-value = 0.365
```

```r
# Q42
kruskal.test(Q42~Q3, data = d) # N.S.
```

```
##
##  Kruskal-Wallis rank sum test
##
## data:  Q42 by Q3
## Kruskal-Wallis chi-squared = 2.1471, df = 2, p-value = 0.3418
```

```r
# Q43
kruskal.test(Q43~Q3, data = d)
```

```
##
```

```
##  Kruskal-Wallis rank sum test
##
## data:  Q43 by Q3
## Kruskal-Wallis chi-squared = 6.2243, df = 2, p-value = 0.04451
```

```r
pairwise.wilcox.test(d$Q43,d$Q3, p.adjust.method = "holm")
```

```
##
##  Pairwise comparisons using Wilcoxon rank sum test with continuity correction
##
## data:  d$Q43 and d$Q3
##
##   1     2
## 2 0.606 -
## 3 0.229 0.042
##
## P value adjustment method: holm
```

```r
# Q44r1
kruskal.test(Q44r1~Q3, data = d) # N.S.
```

```
##
##  Kruskal-Wallis rank sum test
##
## data:  Q44r1 by Q3
## Kruskal-Wallis chi-squared = 0.34493, df = 2, p-value = 0.8416
```

```r
# Q44r2
kruskal.test(Q44r2~Q3, data = d)
```

```
##
##  Kruskal-Wallis rank sum test
##
## data:  Q44r2 by Q3
## Kruskal-Wallis chi-squared = 8.693, df = 2, p-value = 0.01295
```

```r
pairwise.wilcox.test(d$Q44r2, d$Q3, p.adjust.method = "holm")
```

```
##
##  Pairwise comparisons using Wilcoxon rank sum test with continuity correction
##
## data:  d$Q44r2 and d$Q3
##
##   1     2
## 2 0.049 -
## 3 0.012 0.322
##
## P value adjustment method: holm
```

```r
# Q44r3
kruskal.test(Q44r3~Q3, data = d) # N.S.
```

```
##
##  Kruskal-Wallis rank sum test
##
## data:  Q44r3 by Q3
## Kruskal-Wallis chi-squared = 0.87663, df = 2, p-value = 0.6451
```

```r
# Q44r4
kruskal.test(Q44r4~Q3, data = d) # N.S.
```

```
##
##  Kruskal-Wallis rank sum test
##
## data:  Q44r4 by Q3
## Kruskal-Wallis chi-squared = 4.5823, df = 2, p-value = 0.1011
```

```r
# Q44r5
kruskal.test(Q44r5~Q3, data = d) # N.S.
```

```
##
##  Kruskal-Wallis rank sum test
##
## data:  Q44r5 by Q3
## Kruskal-Wallis chi-squared = 3.3982, df = 2, p-value = 0.1828
```

```r
# Q44r6
kruskal.test(Q44r6~Q3, data = d) # N.S.
```

```
##
##  Kruskal-Wallis rank sum test
##
## data:  Q44r6 by Q3
## Kruskal-Wallis chi-squared = 3.5073, df = 2, p-value = 0.1731
```

```r
# MANAGEMENT vs OWNERS PRIMARY all answers ####
# Q41
kruskal.test(Q41~Q15, data = d) # N.S.
```

```
##
##  Kruskal-Wallis rank sum test
##
## data:  Q41 by Q15
## Kruskal-Wallis chi-squared = 7.0421, df = 3, p-value = 0.07057
```

```r
# Q42
kruskal.test(Q42~Q15, data = d)
```

```
##
##  Kruskal-Wallis rank sum test
##
## data:  Q42 by Q15
## Kruskal-Wallis chi-squared = 12.123, df = 3, p-value = 0.006975
```

```r
pairwise.wilcox.test(d$Q42,d$Q15, p.adjust.method = "holm")
```

```
##
##  Pairwise comparisons using Wilcoxon rank sum test with continuity correction
##
## data:  d$Q42 and d$Q15
##
##   1      2      3
## 2 0.4227 -      -
## 3 1.0000 0.0099 -
## 4 0.5283 1.0000 0.1725
##
```

```
## P value adjustment method: holm
```

```r
# Q43
kruskal.test(Q43~Q15, data = d)
```

```
##
##  Kruskal-Wallis rank sum test
##
## data:  Q43 by Q15
## Kruskal-Wallis chi-squared = 81.96, df = 3, p-value < 2.2e-16
```

```r
pairwise.wilcox.test(d$Q43,d$Q15, p.adjust.method = "holm")
```

```
##
##  Pairwise comparisons using Wilcoxon rank sum test with continuity correction
##
## data:  d$Q43 and d$Q15
##
##   1       2       3
## 2 0.24726 -       -
## 3 0.00043 1.9e-15 -
## 4 0.24726 0.64361 9.4e-08
##
## P value adjustment method: holm
```

```r
# Q44r1
kruskal.test(Q44r1~Q15, data = d)
```

```
##
##  Kruskal-Wallis rank sum test
##
## data:  Q44r1 by Q15
## Kruskal-Wallis chi-squared = 22.594, df = 3, p-value = 4.908e-05
```

```r
pairwise.wilcox.test(d$Q44r1,d$Q15, p.adjust.method = "holm")
```

```
##
##  Pairwise comparisons using Wilcoxon rank sum test with continuity correction
##
## data:  d$Q44r1 and d$Q15
##
##   1       2       3
## 2 1.00000 -       -
## 3 0.10087 0.00015 -
## 4 1.00000 1.00000 0.01297
##
## P value adjustment method: holm
```

```r
# Q44r2
kruskal.test(Q44r2~Q15, data = d)
```

```
##
##  Kruskal-Wallis rank sum test
##
## data:  Q44r2 by Q15
## Kruskal-Wallis chi-squared = 28.818, df = 3, p-value = 2.445e-06
```

```r
pairwise.wilcox.test(d$Q44r2, d$Q15, p.adjust.method = "holm")
```

```
##
##  Pairwise comparisons using Wilcoxon rank sum test with continuity correction
##
## data:  d$Q44r2 and d$Q15
##
##   1     2       3
## 2 0.041 -       -
## 3 0.975 1.7e-06 -
## 4 0.410 0.975   0.041
##
## P value adjustment method: holm
```

```r
# Q44r3
kruskal.test(Q44r3~Q15, data = d)
```

```
##
##  Kruskal-Wallis rank sum test
##
## data:  Q44r3 by Q15
## Kruskal-Wallis chi-squared = 59.423, df = 3, p-value = 7.808e-13
```

```r
pairwise.wilcox.test(d$Q44r3, d$Q15, p.adjust.method = "holm")
```

```
##
##  Pairwise comparisons using Wilcoxon rank sum test with continuity correction
##
## data:  d$Q44r3 and d$Q15
##
##   1       2       3
## 2 0.05248 -       -
## 3 0.04367 1.1e-12 -
## 4 0.39132 0.51575 0.00034
##
## P value adjustment method: holm
```

```r
# Q44r4
kruskal.test(Q44r4~Q15, data = d)
```

```
##
##  Kruskal-Wallis rank sum test
##
## data:  Q44r4 by Q15
## Kruskal-Wallis chi-squared = 108.25, df = 3, p-value < 2.2e-16
```

```r
pairwise.wilcox.test(d$Q44r4, d$Q15, p.adjust.method = "holm")
```

```
##
##  Pairwise comparisons using Wilcoxon rank sum test with continuity correction
##
## data:  d$Q44r4 and d$Q15
##
##   1       2       3
## 2 0.32089 -       -
## 3 2e-07   < 2e-16 -
## 4 0.32089 0.02844 0.00028
```

```
##
## P value adjustment method: holm
```

```r
# Q44r5
kruskal.test(Q44r5~Q15, data = d)
```

```
##
##  Kruskal-Wallis rank sum test
##
## data:  Q44r5 by Q15
## Kruskal-Wallis chi-squared = 103.62, df = 3, p-value < 2.2e-16
```

```r
pairwise.wilcox.test(d$Q44r5, d$Q15, p.adjust.method = "holm")
```

```
##
##  Pairwise comparisons using Wilcoxon rank sum test with continuity correction
##
## data:  d$Q44r5 and d$Q15
##
##   1       2       3
## 2 0.7378  -       -
## 3 2.3e-09 < 2e-16 -
## 4 0.0319  0.0074  0.0074
##
## P value adjustment method: holm
```

```r
# Q44r6
kruskal.test(Q44r6~Q15, data = d)
```

```
##
##  Kruskal-Wallis rank sum test
##
## data:  Q44r6 by Q15
## Kruskal-Wallis chi-squared = 106.49, df = 3, p-value < 2.2e-16
```

```r
pairwise.wilcox.test(d$Q44r6, d$Q15, p.adjust.method = "holm")
```

```
##
##  Pairwise comparisons using Wilcoxon rank sum test with continuity correction
##
## data:  d$Q44r6 and d$Q15
##
##   1       2       3
## 2 0.9242  -       -
## 3 4.1e-10 < 2e-16 -
## 4 0.0378  0.0166  0.0036
##
## P value adjustment method: holm
```

```r
# MANAGEMENT vs OWNERS PRIMARY recode ####
# Q39
kruskal.test(Q39~Q15_recode, data = d)
```

```
##
##  Kruskal-Wallis rank sum test
##
## data:  Q39 by Q15_recode
## Kruskal-Wallis chi-squared = 88.722, df = 1, p-value < 2.2e-16
```

```
# Q41
kruskal.test(Q41~Q15_recode, data = d) # N.S.
```

```
##
##  Kruskal-Wallis rank sum test
##
## data:  Q41 by Q15_recode
## Kruskal-Wallis chi-squared = 2.732, df = 1, p-value = 0.09836
```

```
# Q42
kruskal.test(Q42~Q15_recode, data = d) # N.S.
```

```
##
##  Kruskal-Wallis rank sum test
##
## data:  Q42 by Q15_recode
## Kruskal-Wallis chi-squared = 0.40732, df = 1, p-value = 0.5233
```

```
# Q43
kruskal.test(Q43~Q15_recode, data = d) # N.S.
```

```
##
##  Kruskal-Wallis rank sum test
##
## data:  Q43 by Q15_recode
## Kruskal-Wallis chi-squared = 0.6939, df = 1, p-value = 0.4048
```

```
# Q44r1
kruskal.test(Q44r1~Q15_recode, data = d) # N.S.
```

```
##
##  Kruskal-Wallis rank sum test
##
## data:  Q44r1 by Q15_recode
## Kruskal-Wallis chi-squared = 0.45277, df = 1, p-value = 0.501
```

```
# Q44r2
kruskal.test(Q44r2~Q15_recode, data = d) # N.S.
```

```
##
##  Kruskal-Wallis rank sum test
##
## data:  Q44r2 by Q15_recode
## Kruskal-Wallis chi-squared = 1.9781, df = 1, p-value = 0.1596
```

```
# Q44r3
kruskal.test(Q44r3~Q15_recode, data = d) # N.S.
```

```
##
##  Kruskal-Wallis rank sum test
##
## data:  Q44r3 by Q15_recode
## Kruskal-Wallis chi-squared = 0.0094649, df = 1, p-value = 0.9225
```

```
# Q44r4
kruskal.test(Q44r4~Q15_recode, data = d)
```

```
##
##  Kruskal-Wallis rank sum test
```

```
##
## data:  Q44r4 by Q15_recode
## Kruskal-Wallis chi-squared = 7.5964, df = 1, p-value = 0.005848
```

```r
# Q44r5
kruskal.test(Q44r5~Q15_recode, data = d)
```

```
##
##  Kruskal-Wallis rank sum test
##
## data:  Q44r5 by Q15_recode
## Kruskal-Wallis chi-squared = 16.19, df = 1, p-value = 5.73e-05
```

```r
# Q44r6
kruskal.test(Q44r6~Q15_recode, data = d)
```

```
##
##  Kruskal-Wallis rank sum test
##
## data:  Q44r6 by Q15_recode
## Kruskal-Wallis chi-squared = 18.651, df = 1, p-value = 1.569e-05
```

```r
# MANAGEMENT vs OWNERS SECONDARY recode ####
# Q39
kruskal.test(Q39~Q22_recode, data = d)
```

```
##
##  Kruskal-Wallis rank sum test
##
## data:  Q39 by Q22_recode
## Kruskal-Wallis chi-squared = 25.857, df = 1, p-value = 3.676e-07
```

```r
# Q41
kruskal.test(Q41~Q22_recode, data = d)
```

```
##
##  Kruskal-Wallis rank sum test
##
## data:  Q41 by Q22_recode
## Kruskal-Wallis chi-squared = 15.766, df = 1, p-value = 7.169e-05
```

```r
# Q42
kruskal.test(Q42~Q22_recode, data = d) # N.S.
```

```
##
##  Kruskal-Wallis rank sum test
##
## data:  Q42 by Q22_recode
## Kruskal-Wallis chi-squared = 0.21914, df = 1, p-value = 0.6397
```

```r
# Q43
kruskal.test(Q43~Q22_recode, data = d) # N.S.
```

```
##
##  Kruskal-Wallis rank sum test
##
## data:  Q43 by Q22_recode
## Kruskal-Wallis chi-squared = 0.011395, df = 1, p-value = 0.915
```

```r
# Q44r1
kruskal.test(Q44r1~Q22_recode, data = d) # N.S.
```

```
##
##  Kruskal-Wallis rank sum test
##
## data:  Q44r1 by Q22_recode
## Kruskal-Wallis chi-squared = 3.7893, df = 1, p-value = 0.05158
```

```r
# Q44r2
kruskal.test(Q44r2~Q22_recode, data = d)
```

```
##
##  Kruskal-Wallis rank sum test
##
## data:  Q44r2 by Q22_recode
## Kruskal-Wallis chi-squared = 5.0563, df = 1, p-value = 0.02454
```

```r
# Q44r3
kruskal.test(Q44r3~Q22_recode, data = d) # N.S.
```

```
##
##  Kruskal-Wallis rank sum test
##
## data:  Q44r3 by Q22_recode
## Kruskal-Wallis chi-squared = 1.9905, df = 1, p-value = 0.1583
```

```r
# Q44r4
kruskal.test(Q44r4~Q22_recode, data = d) # N.S.
```

```
##
##  Kruskal-Wallis rank sum test
##
## data:  Q44r4 by Q22_recode
## Kruskal-Wallis chi-squared = 0.18484, df = 1, p-value = 0.6672
```

```r
# Q44r5
kruskal.test(Q44r5~Q22_recode, data = d) # N.S.
```

```
##
##  Kruskal-Wallis rank sum test
##
## data:  Q44r5 by Q22_recode
## Kruskal-Wallis chi-squared = 0.10064, df = 1, p-value = 0.7511
```

```r
# Q44r6
kruskal.test(Q44r6~Q22_recode, data = d) # N.S.
```

```
##
##  Kruskal-Wallis rank sum test
##
## data:  Q44r6 by Q22_recode
## Kruskal-Wallis chi-squared = 0.36227, df = 1, p-value = 0.5472
```
