# Supplementary material for: Iconic but Invasive: The Public Perception of the Chinese Windmill Palm (Trachycarpus fortunei) in Switzerland
Source: Environ Manage. 2022 Apr 26;70(4):618–32. doi: 10.1007/s00267-022-01646-3 (PMC9439986; doi:10.1007/s00267-022-01646-3)

Supplementary Materials S6

Additional plots

Additional plots representing differences among the social groups based on their information level, perception, preference and management acceptance. In addition, plots representing the importance of the information box are added.

p. 2 Information level

p. 19 Perception questions

p. 24 Importance of the information box

p. 28 Preference questions

p. 43 Acceptance of the management options

Information level

p. 3 Age class

p. 5 Level of education

p. 9 Native language

p. 11 Current occupation

p. 15 Owners and non-owners of *T. fortunei*

p. 17 Zone of residence

Information level according to the age class (1/2)

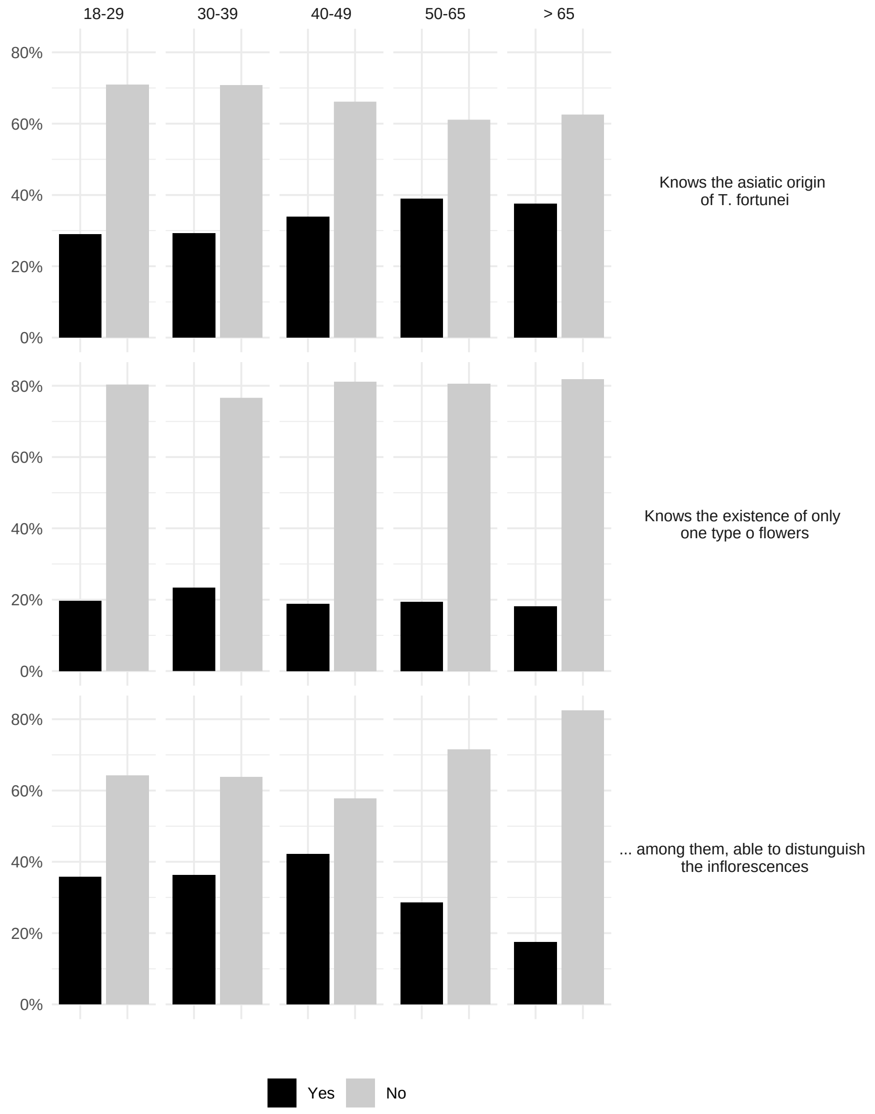

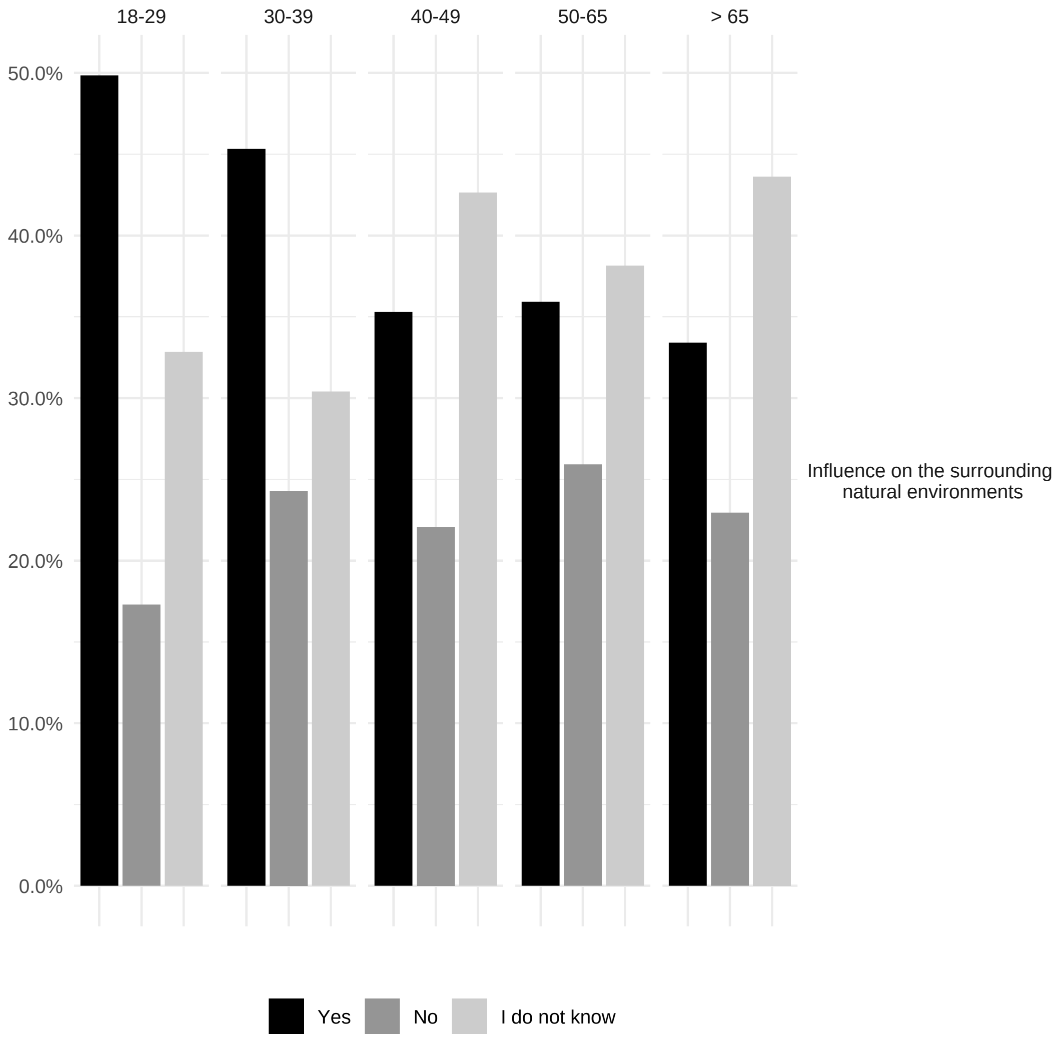

Information level according to the age class (2/2)

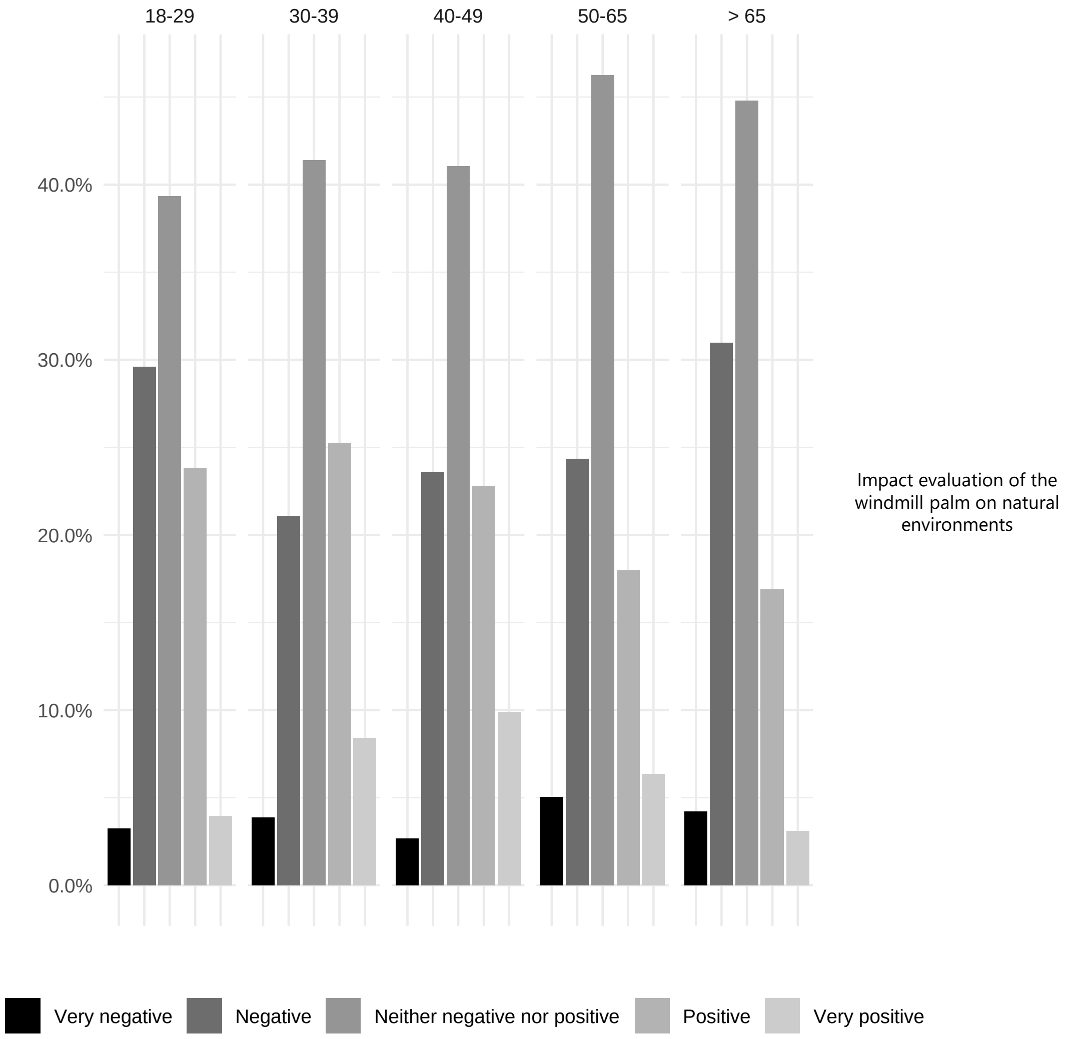

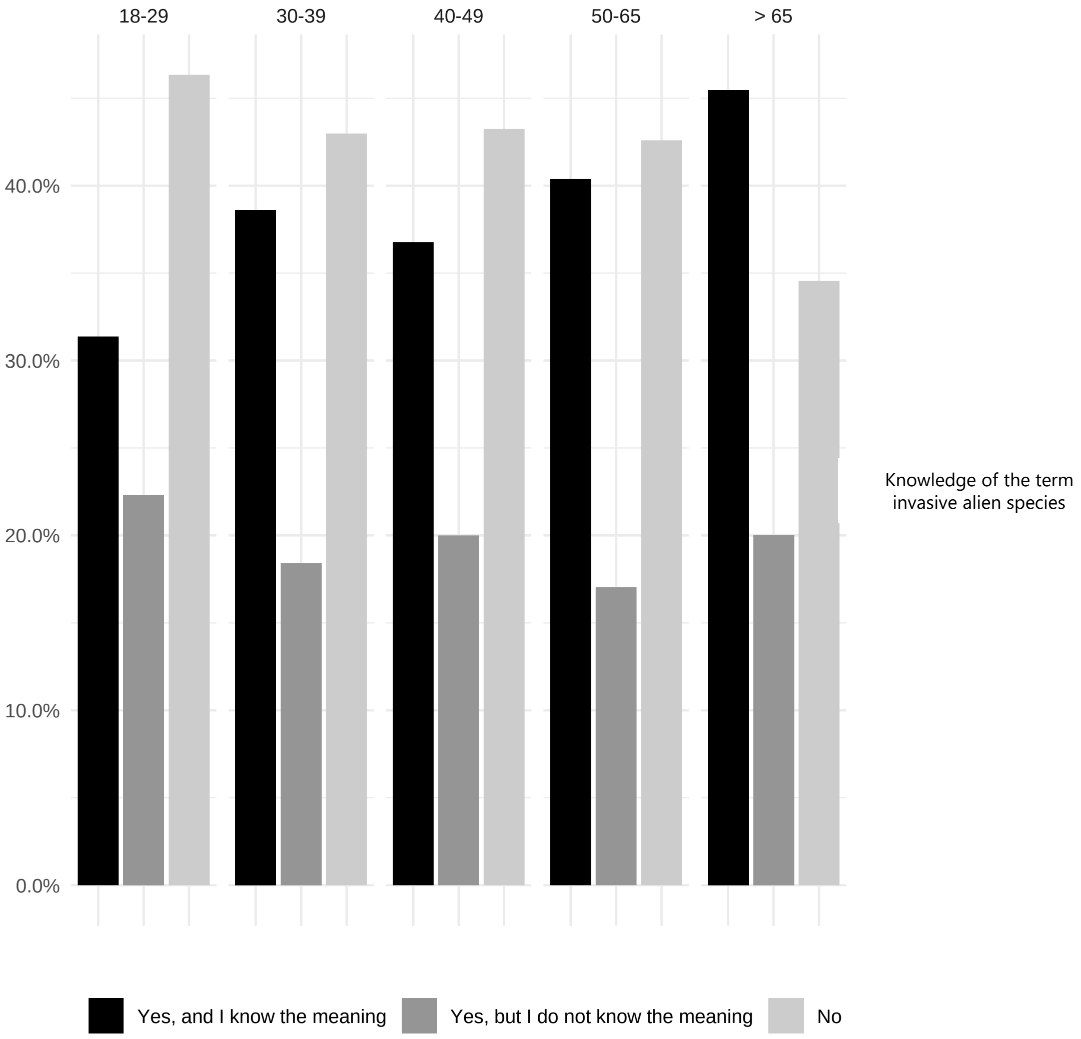

Information level according to the level of education (1/4)

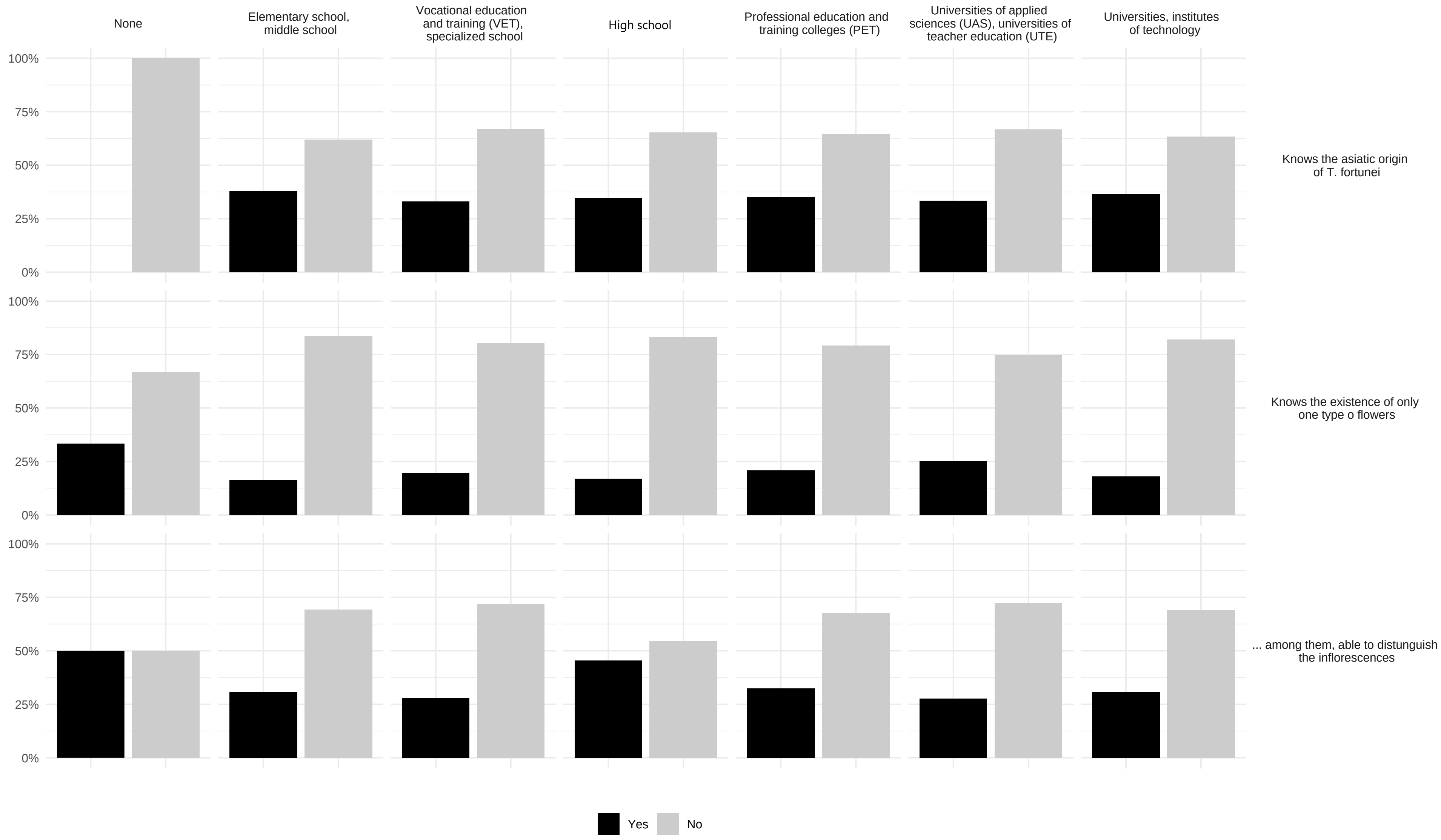

Information level according to the level of education (2/4)

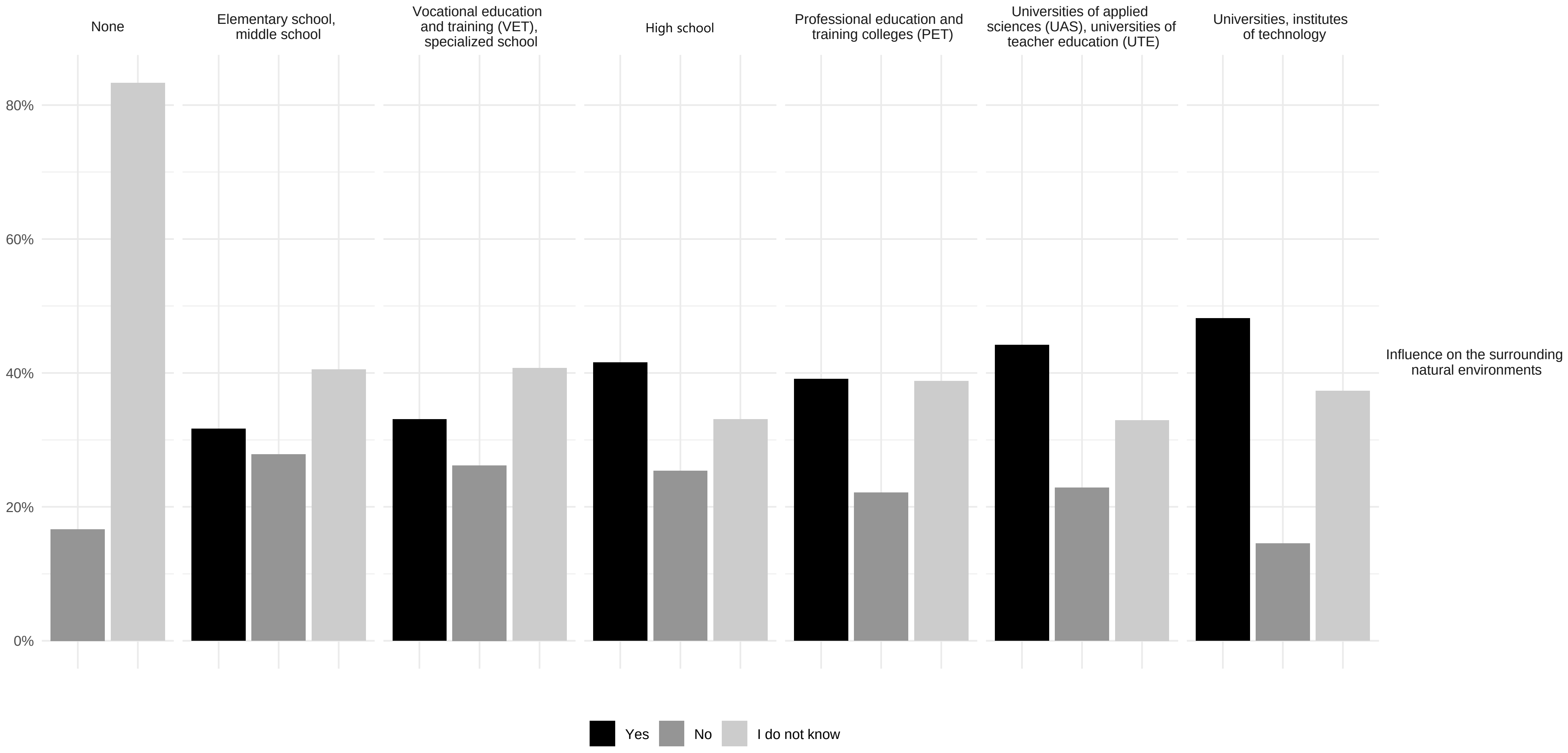

Information level according to the level of education (3/4)

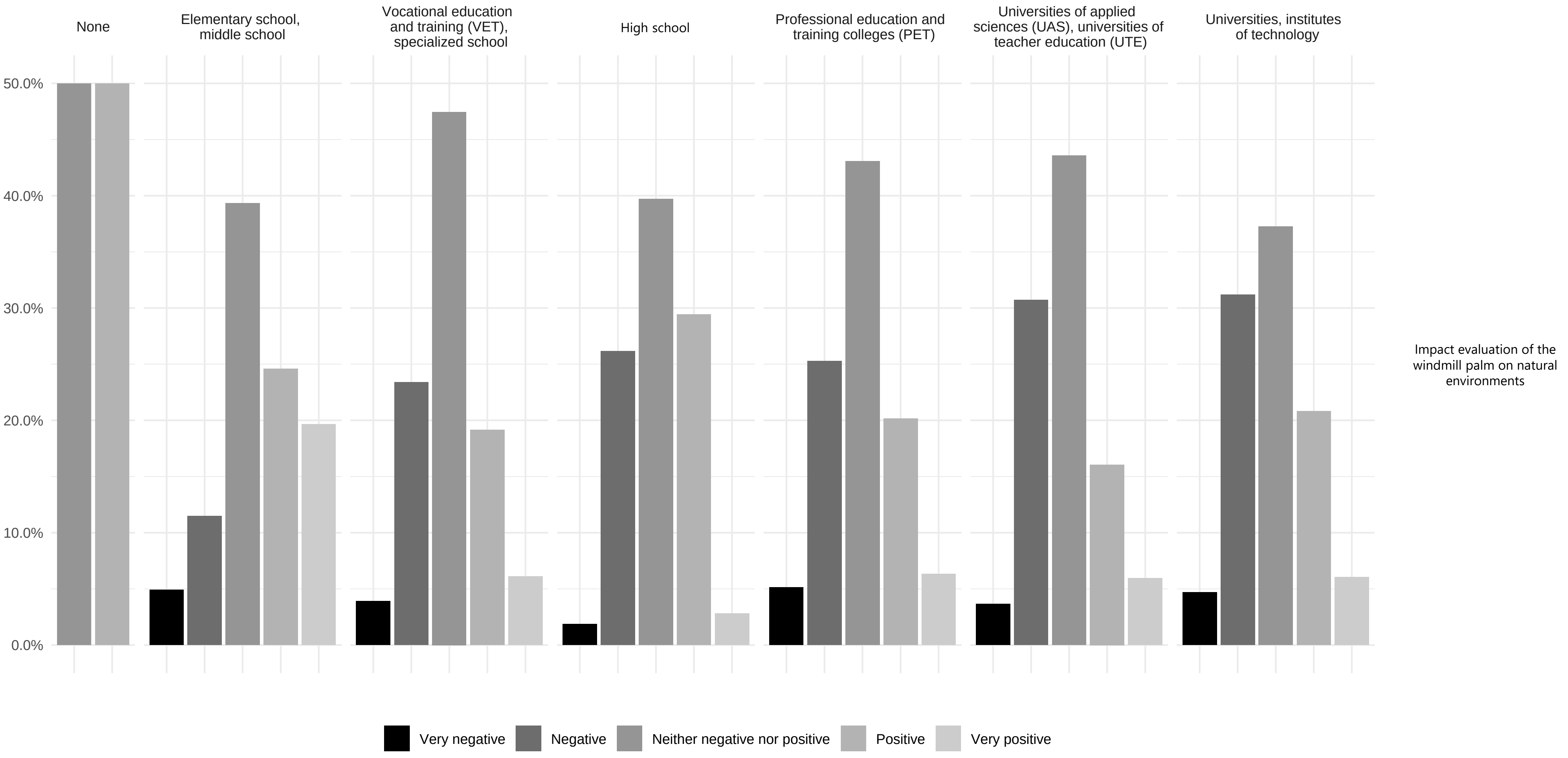

Information level according to the level of education (4/4)

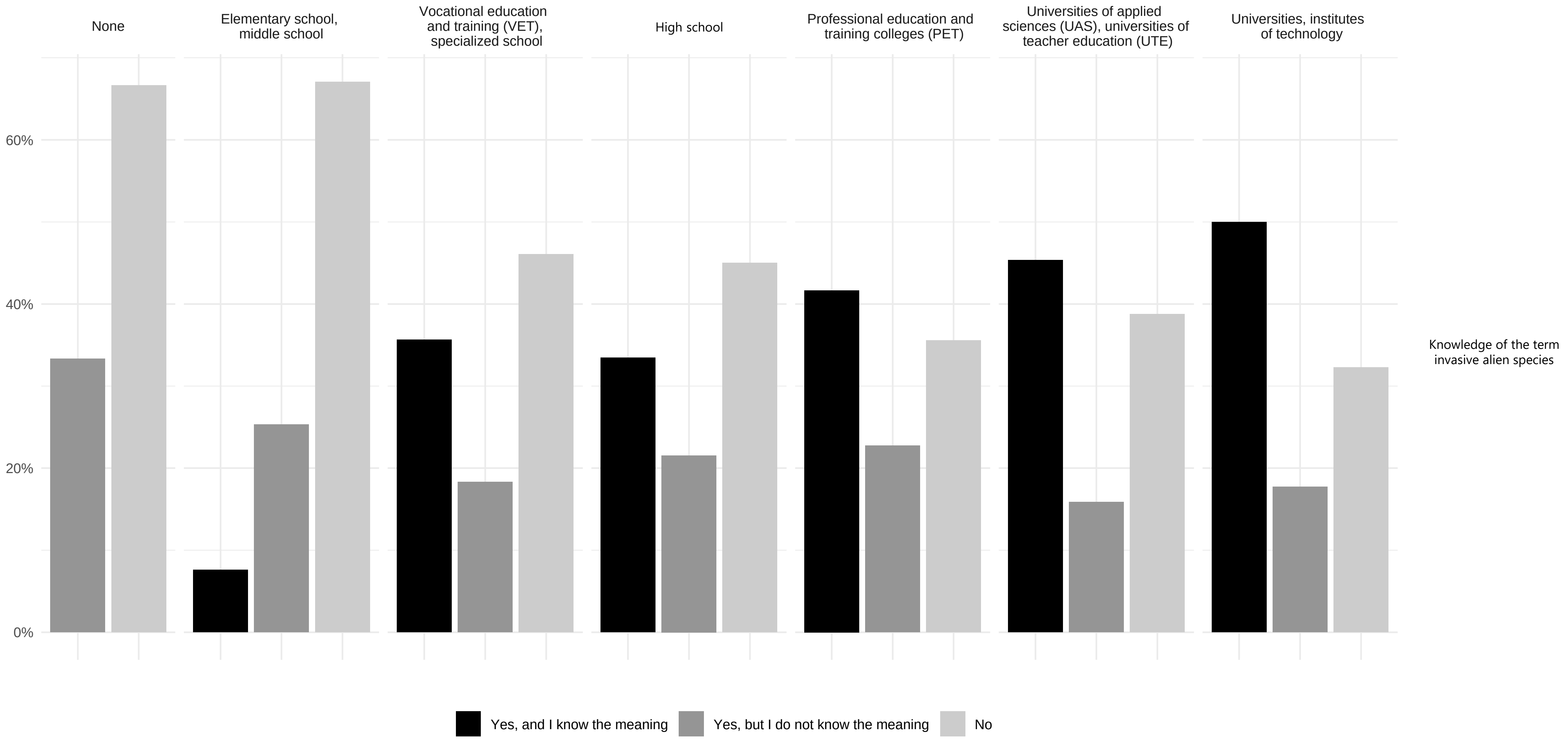

Information level according to the native language (1/2)

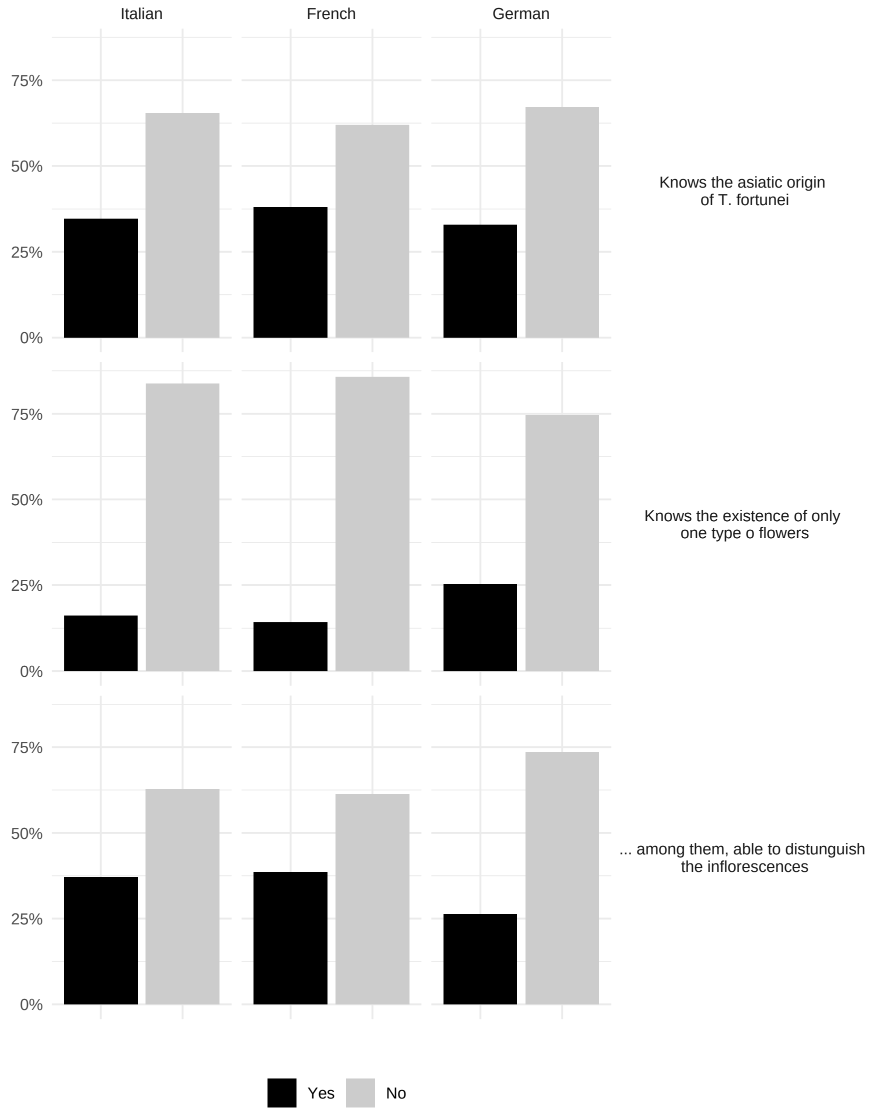

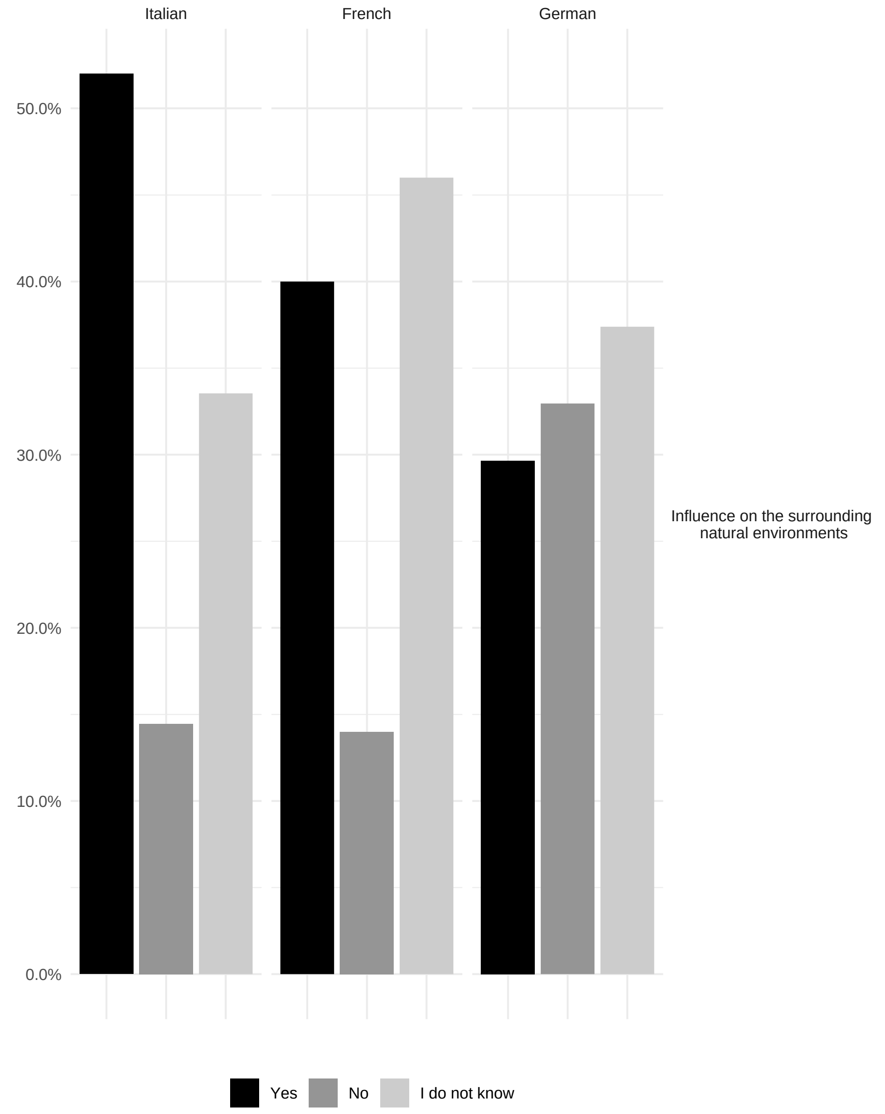

Information level according to the native language (2/2)

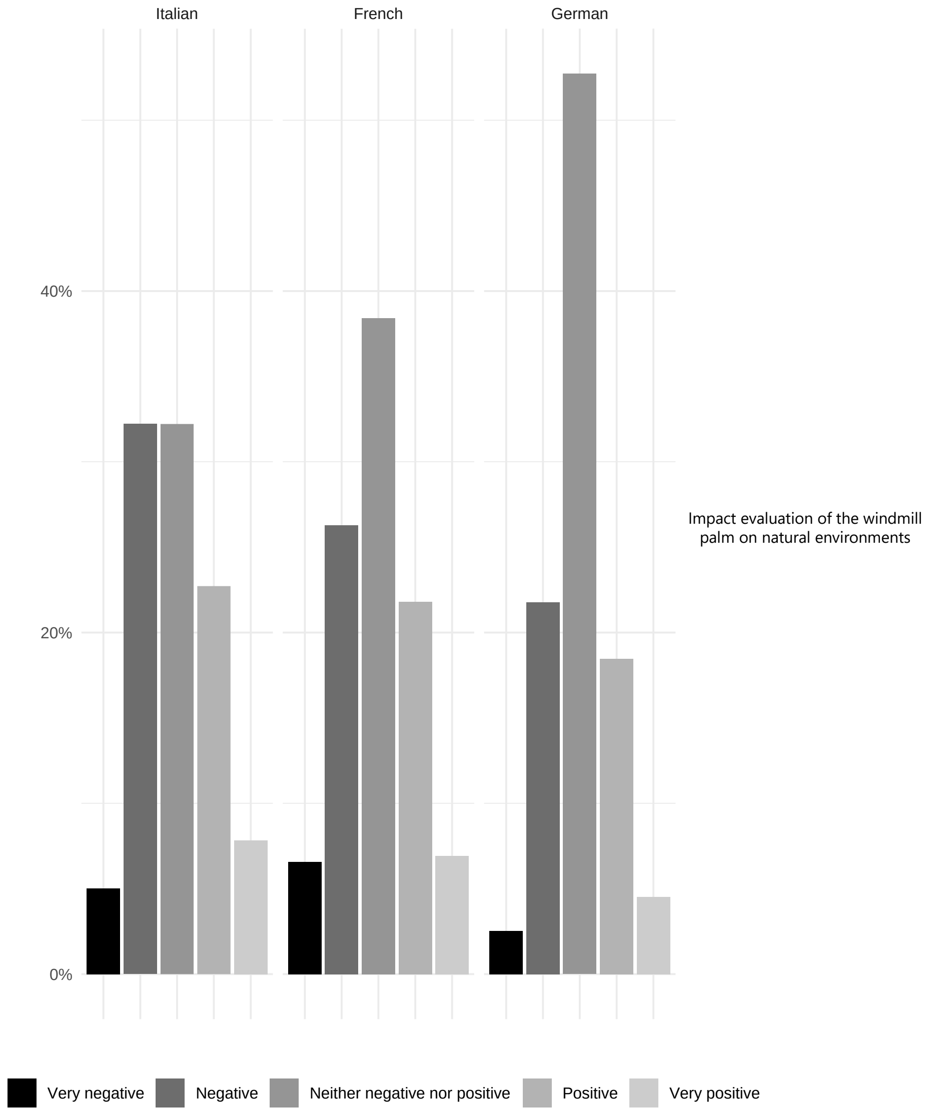

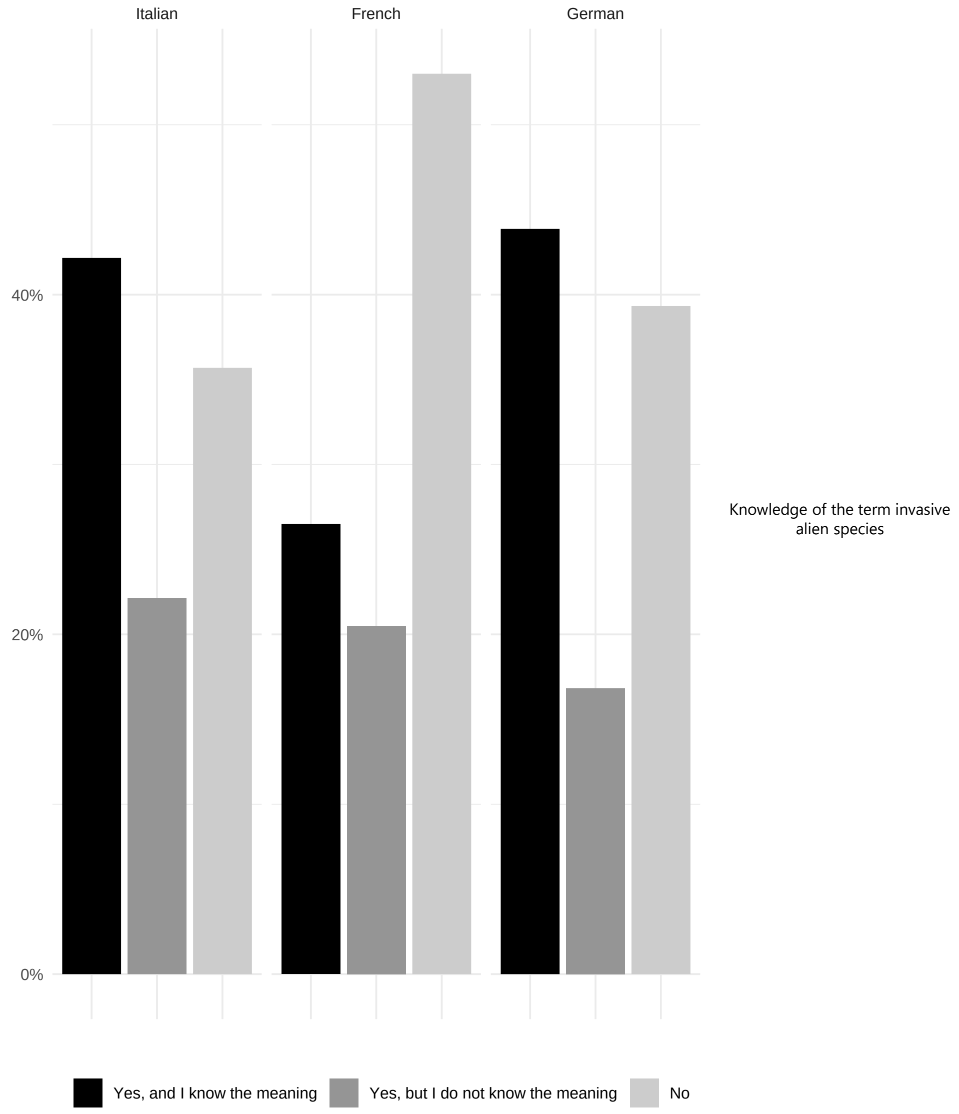

Information level according to the current occupation (1/4)

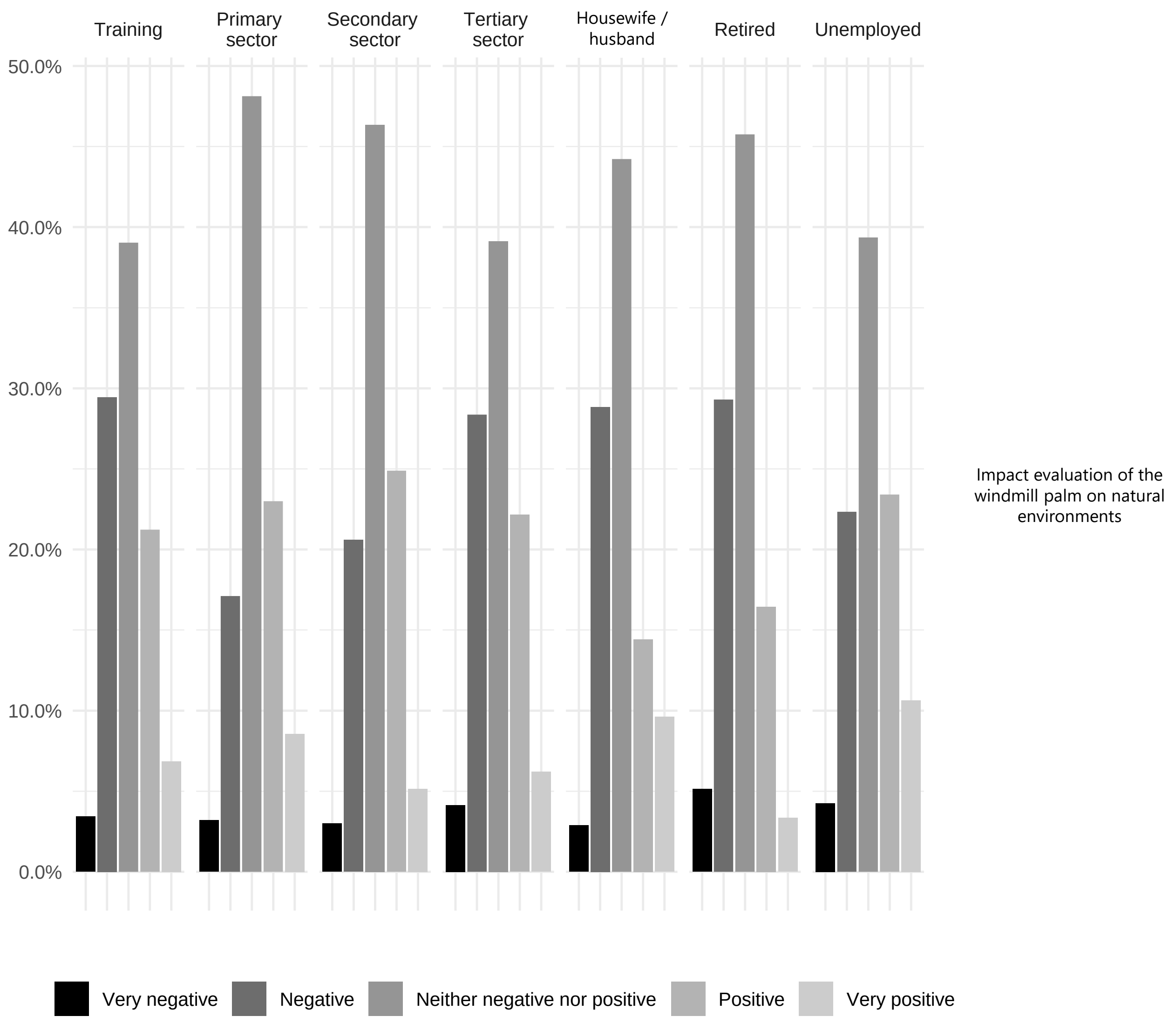

Information level according to the current occupation (2/4)

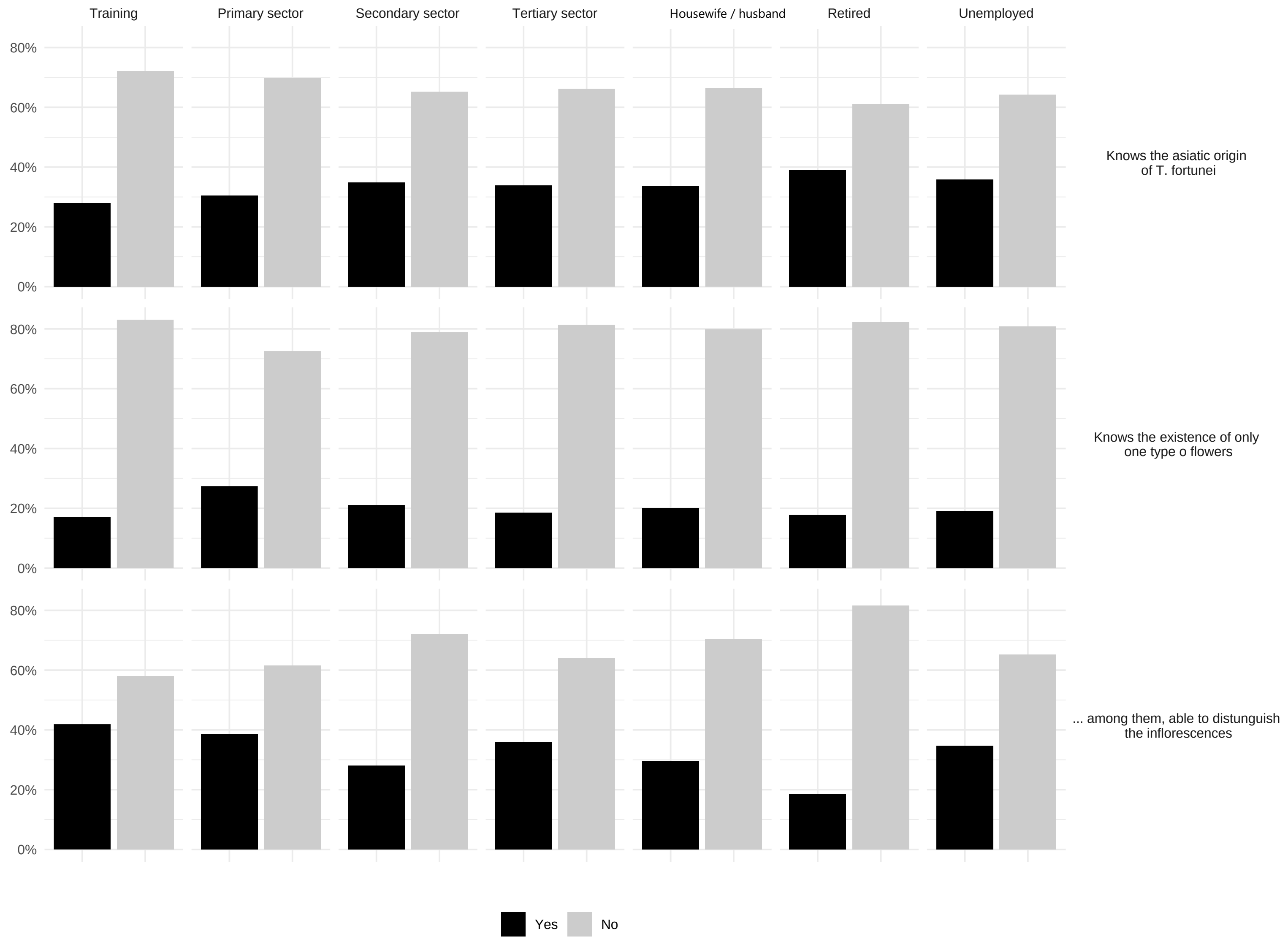

Information level according to the current occupation (3/4)

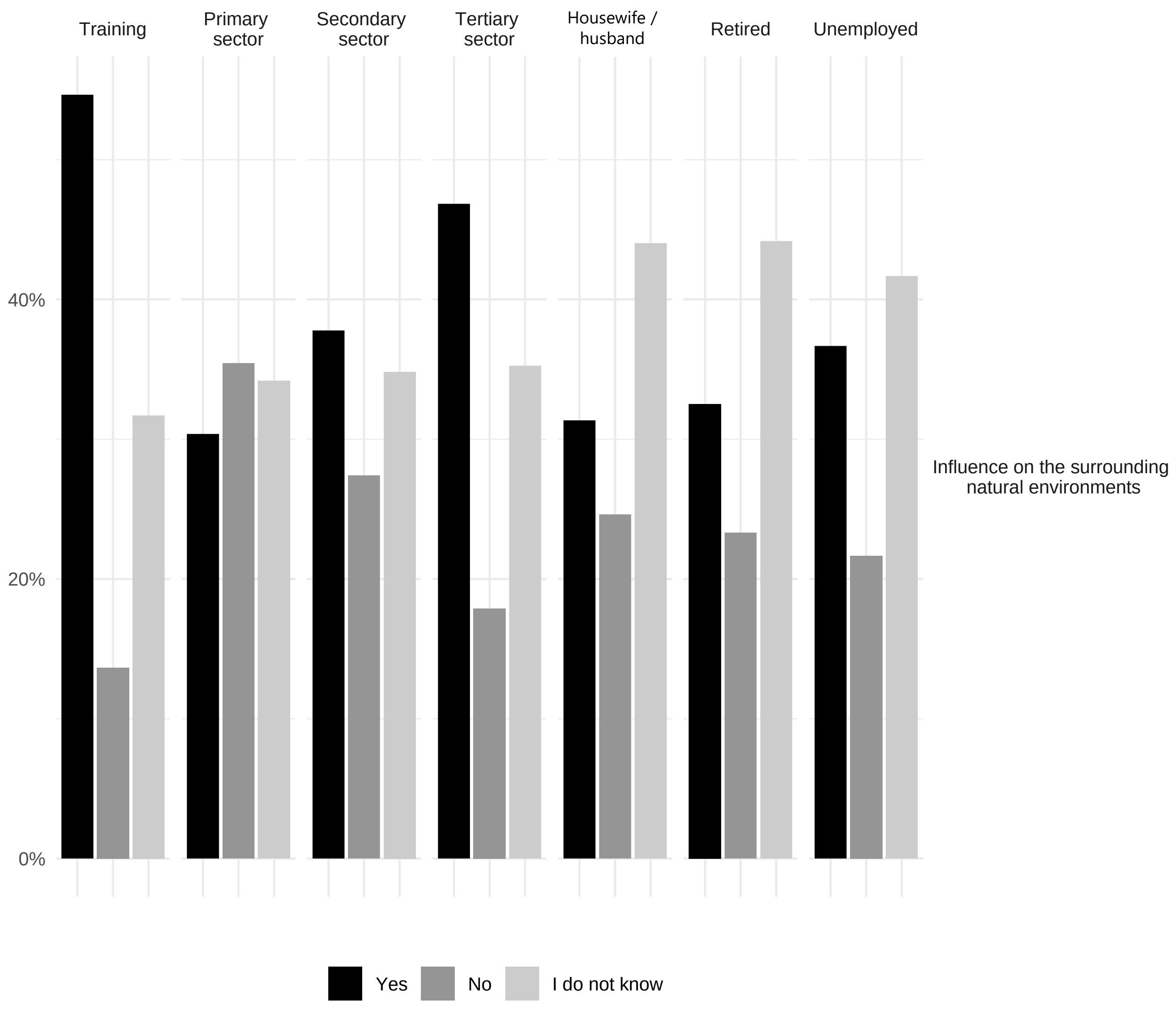

Information level according to the current occupation (4/4)

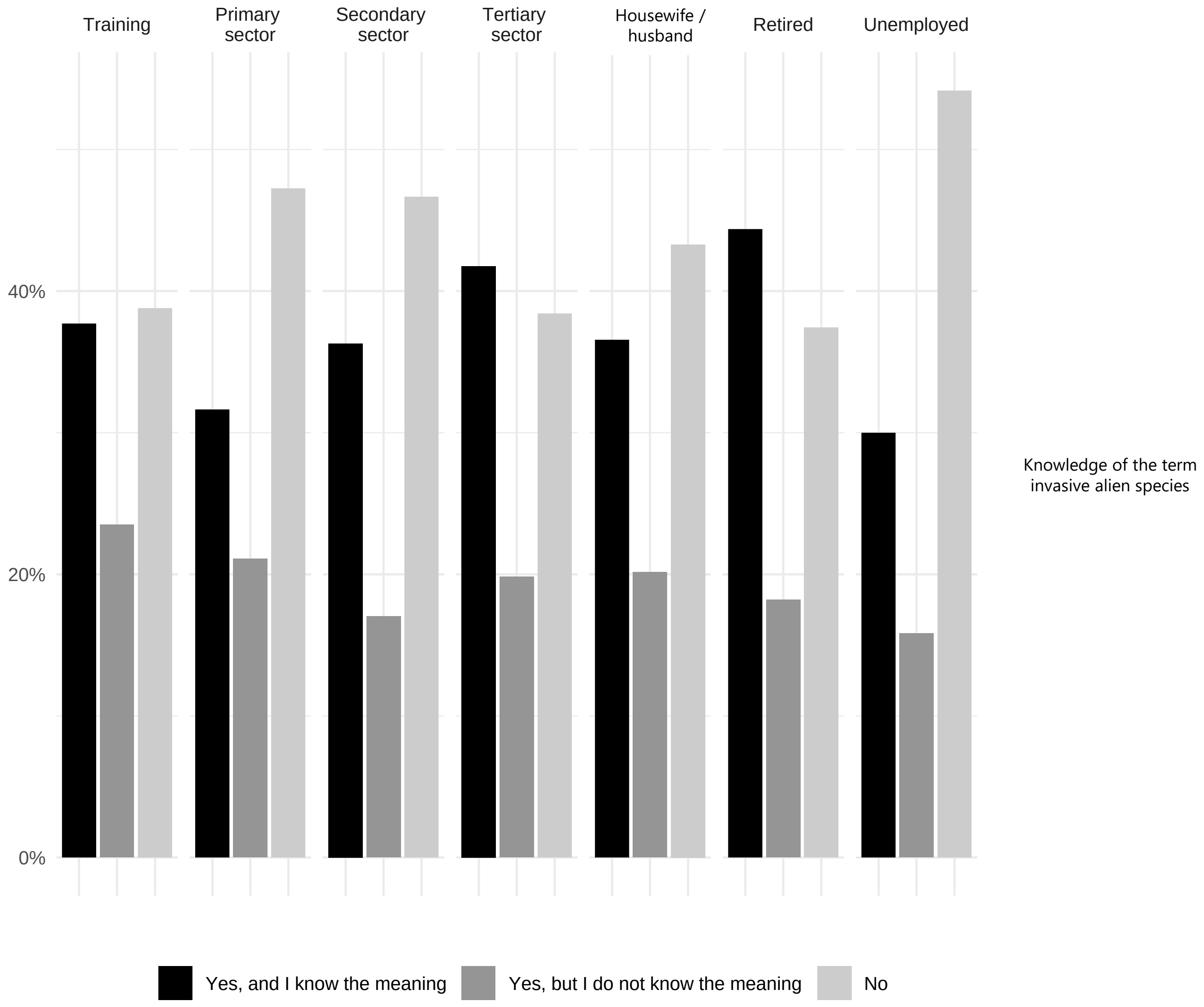

Information level among owners and non-owners of *T. fortunei* (1/2)

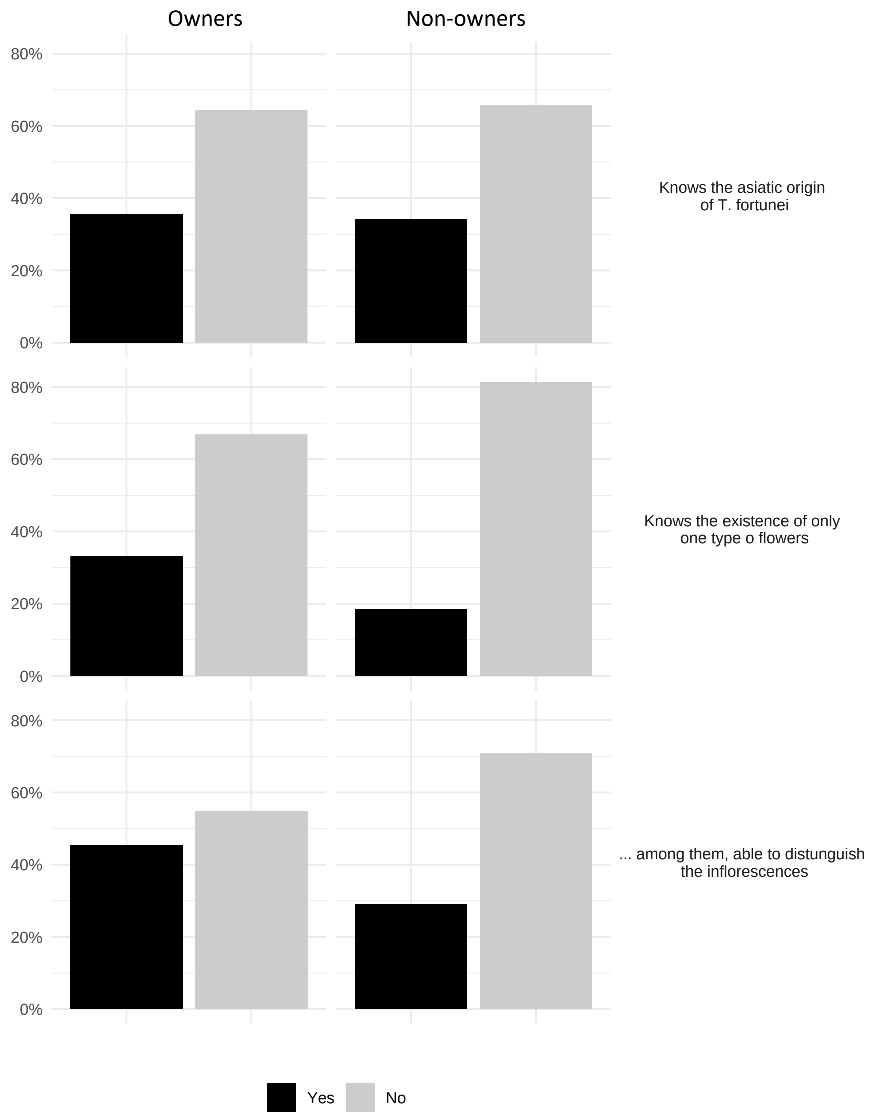

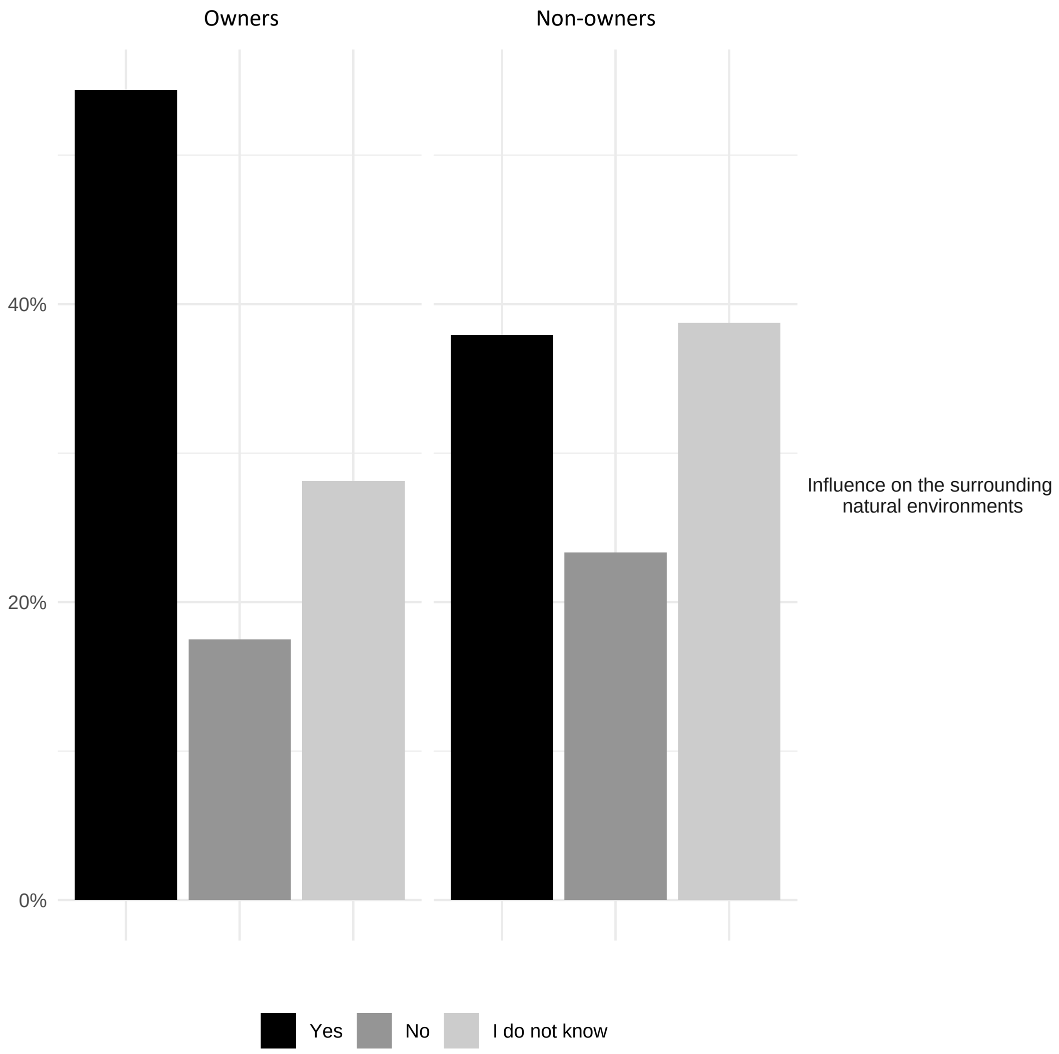

Information level among owners and non-owners of *T. fortunei* (2/2)

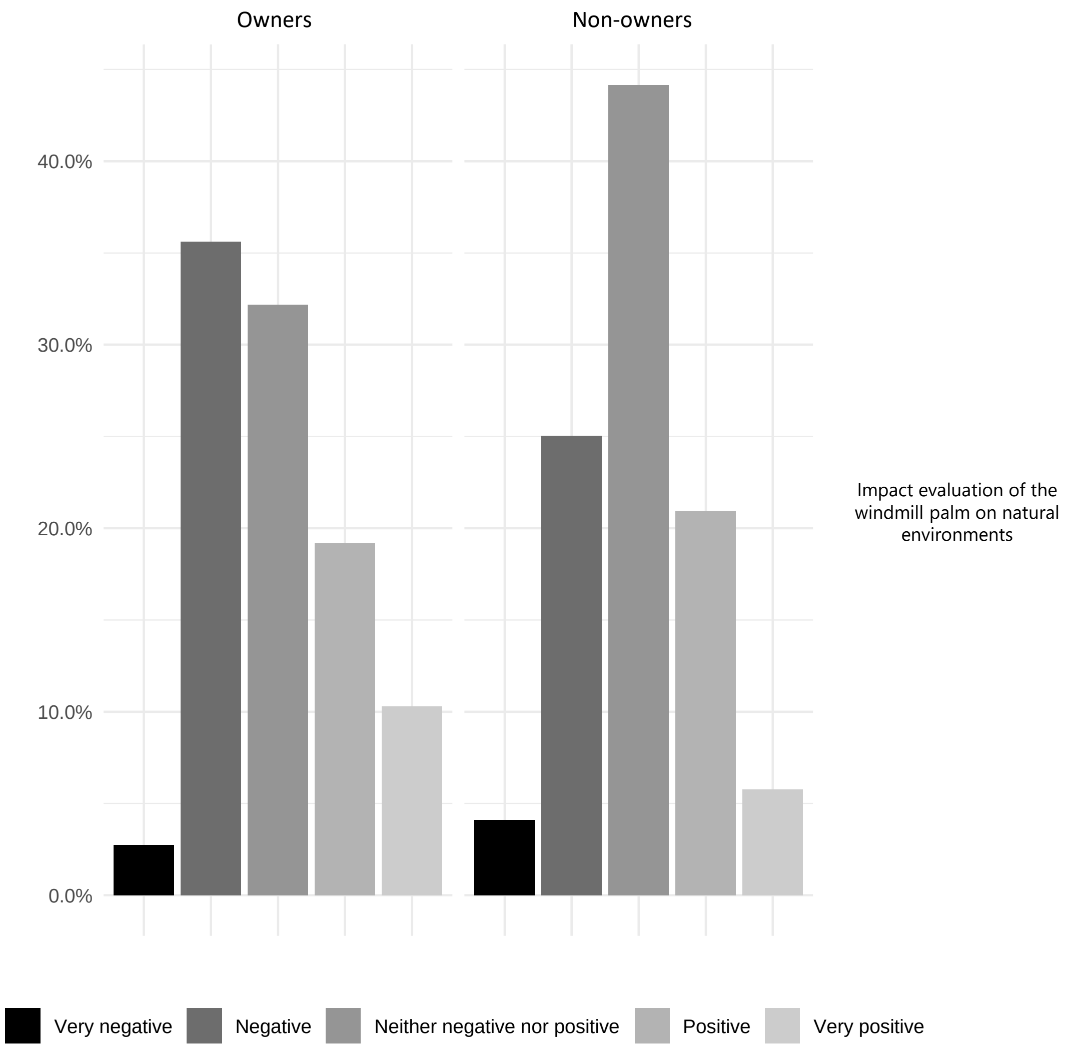

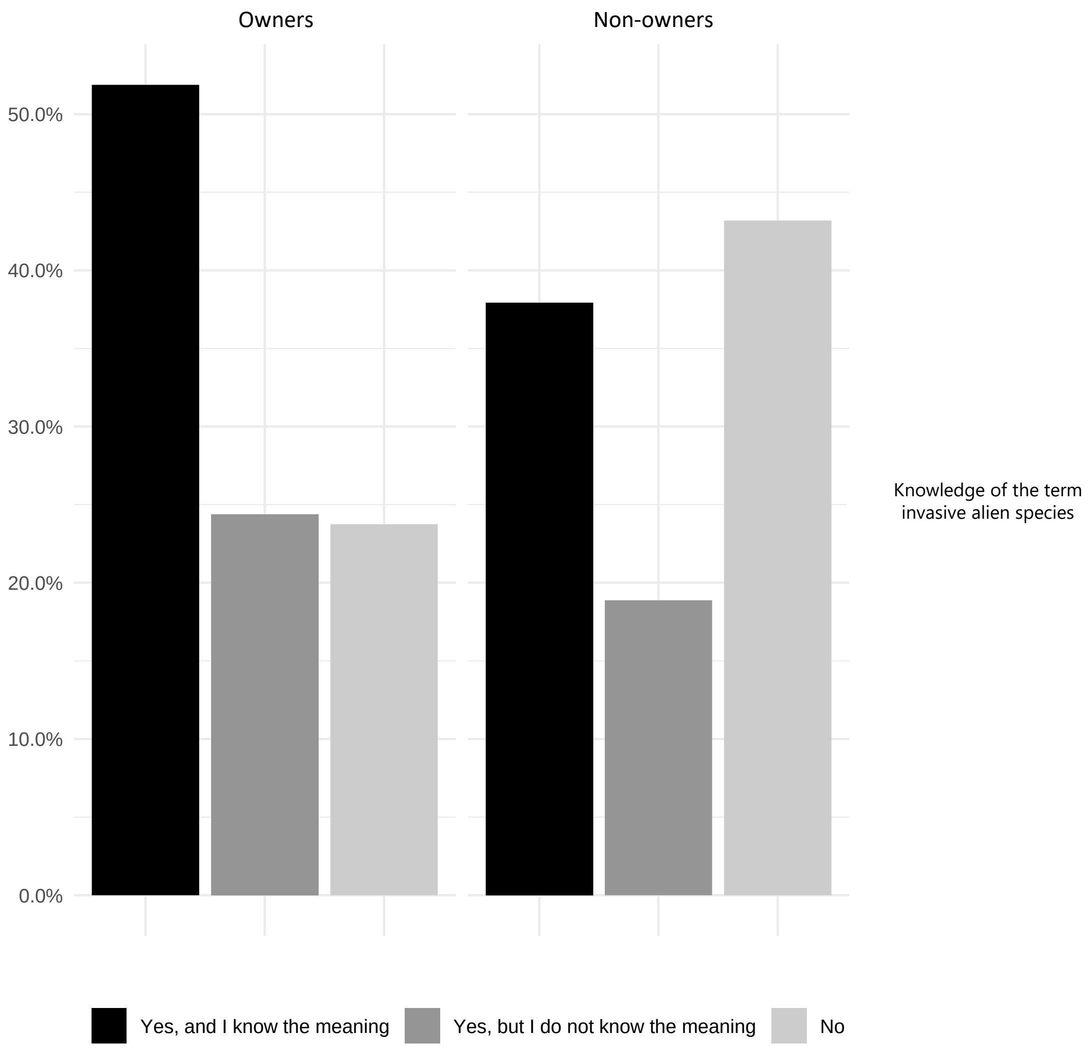

Information level according to the zone of residence (1/2)

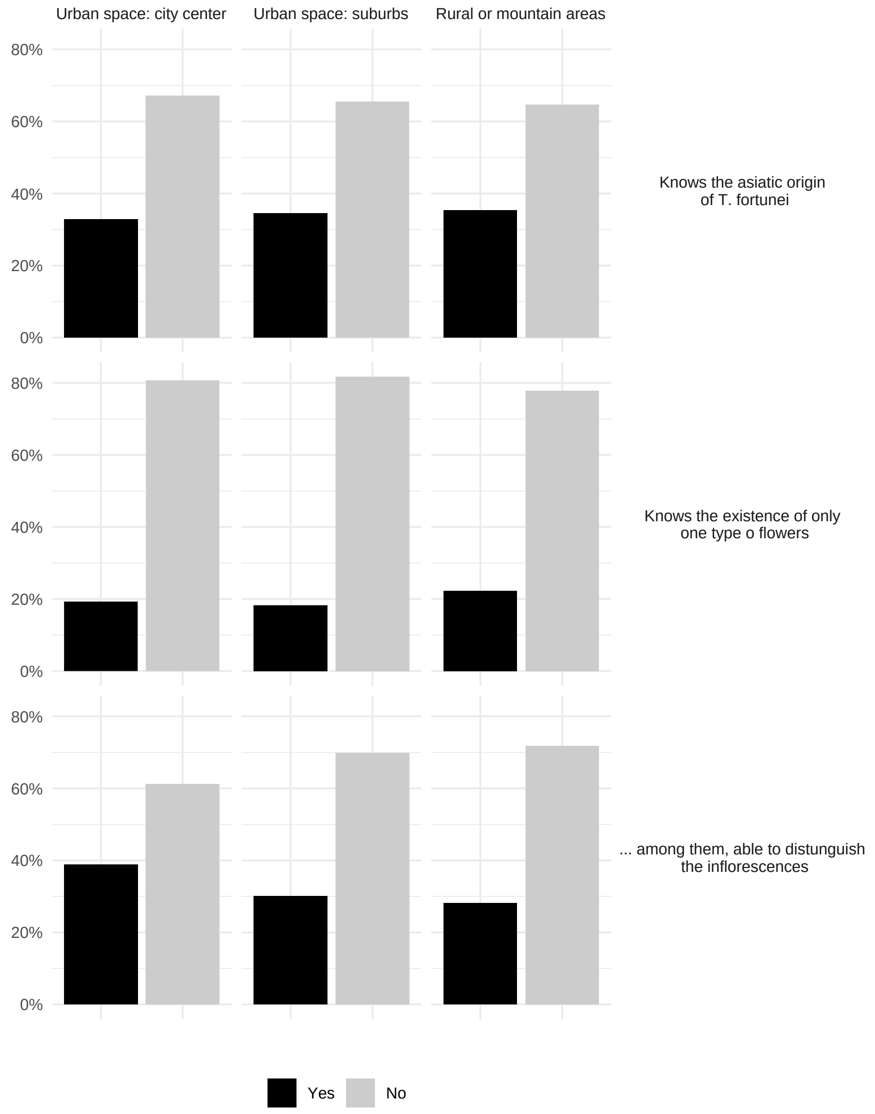

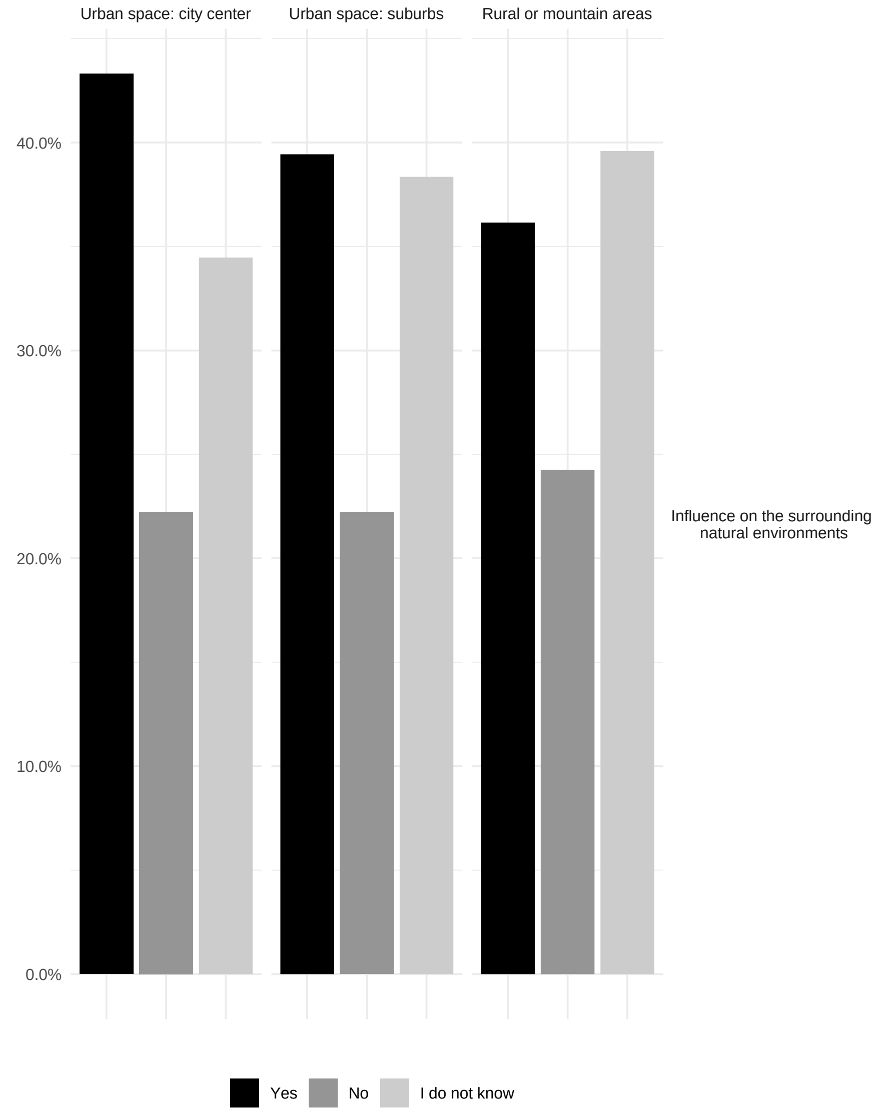

Information level according to the zone of residence (2/2)

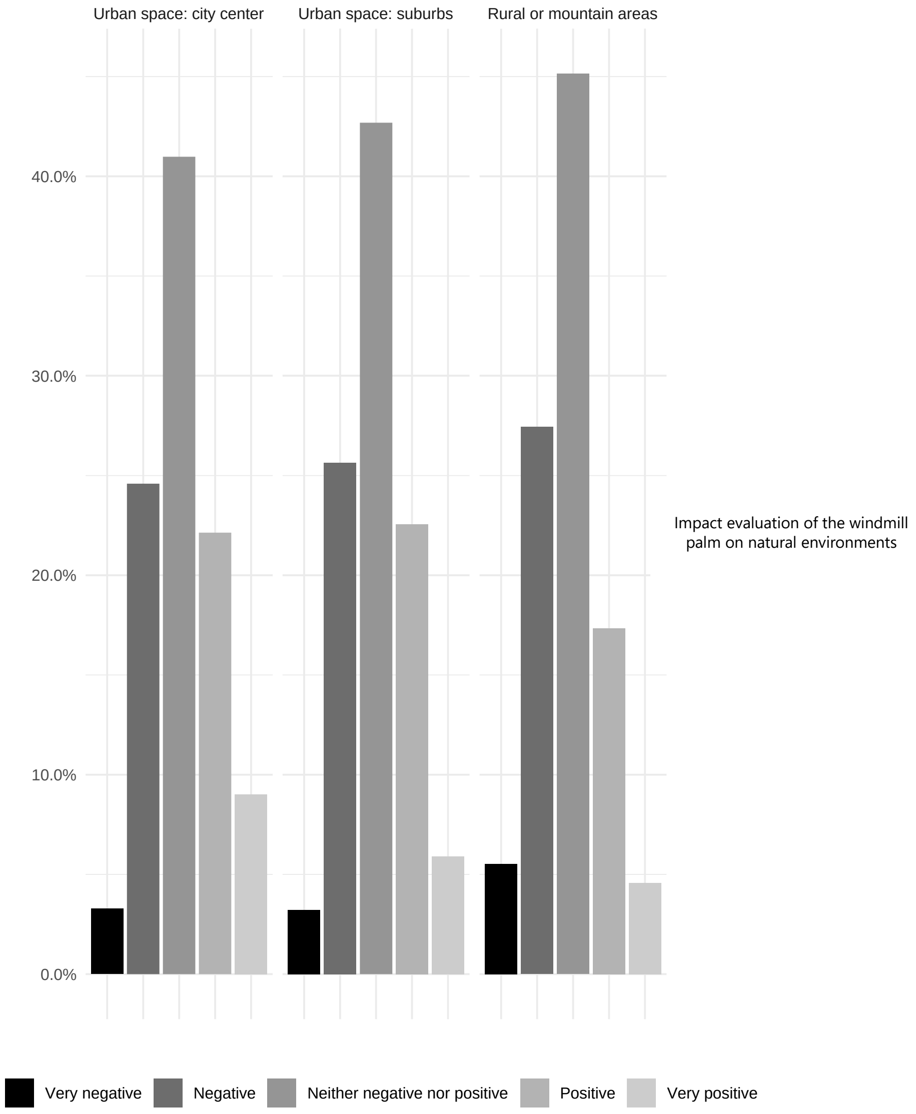

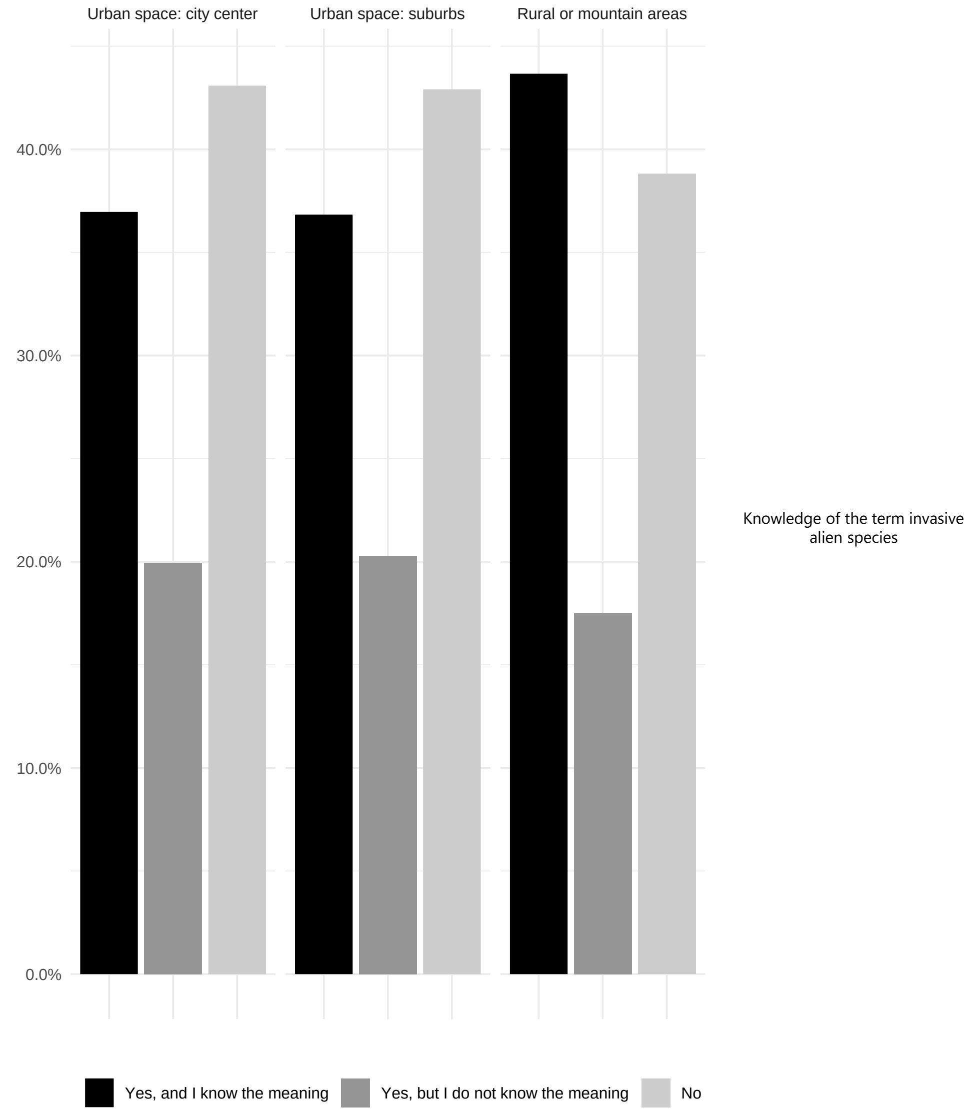

Perception questions

p. 20 Age class

p. 21 Level of education

p. 22 Current occupation

p. 23 Zone of residence and Owners and non-owners of *T. fortunei*

Perception according to the age class

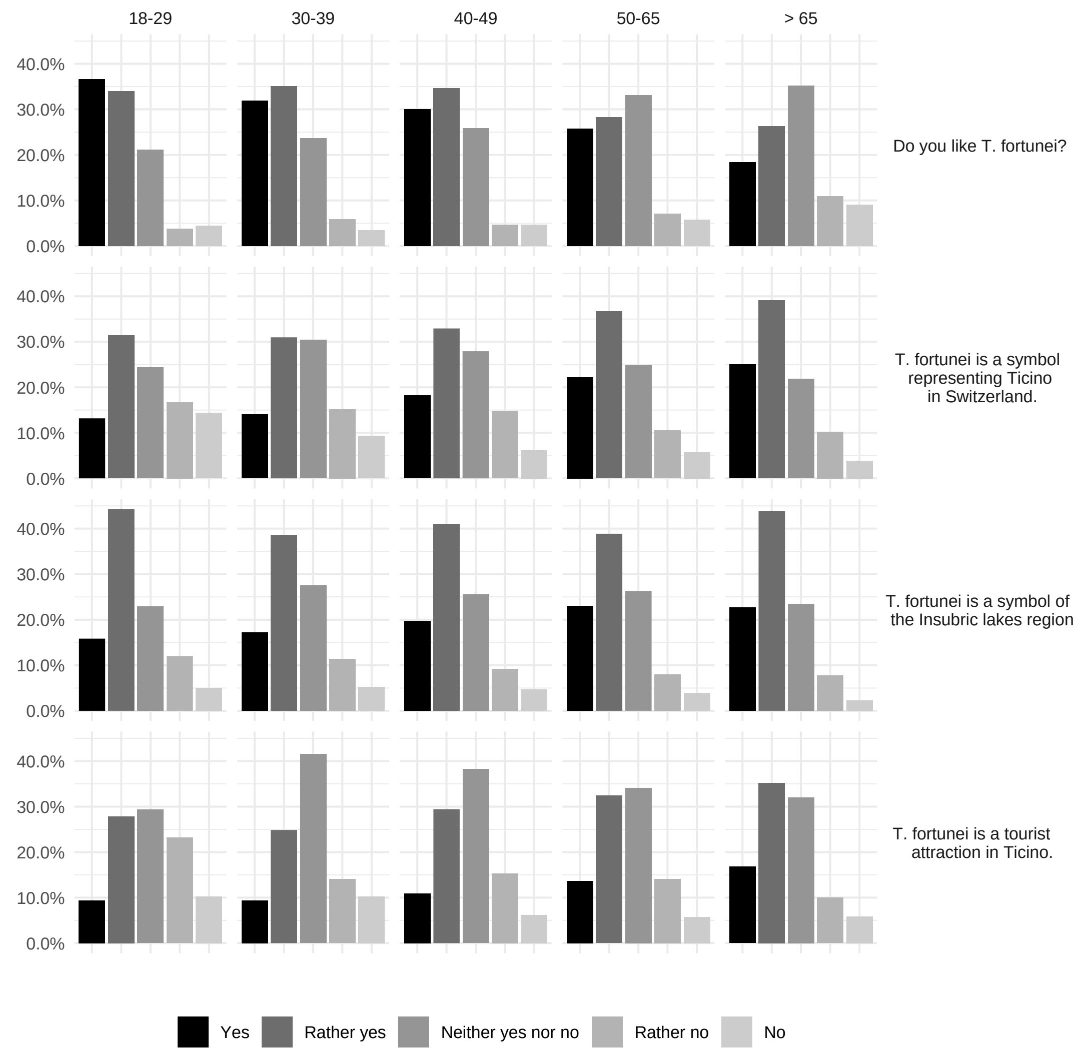

Perception according to the level of education

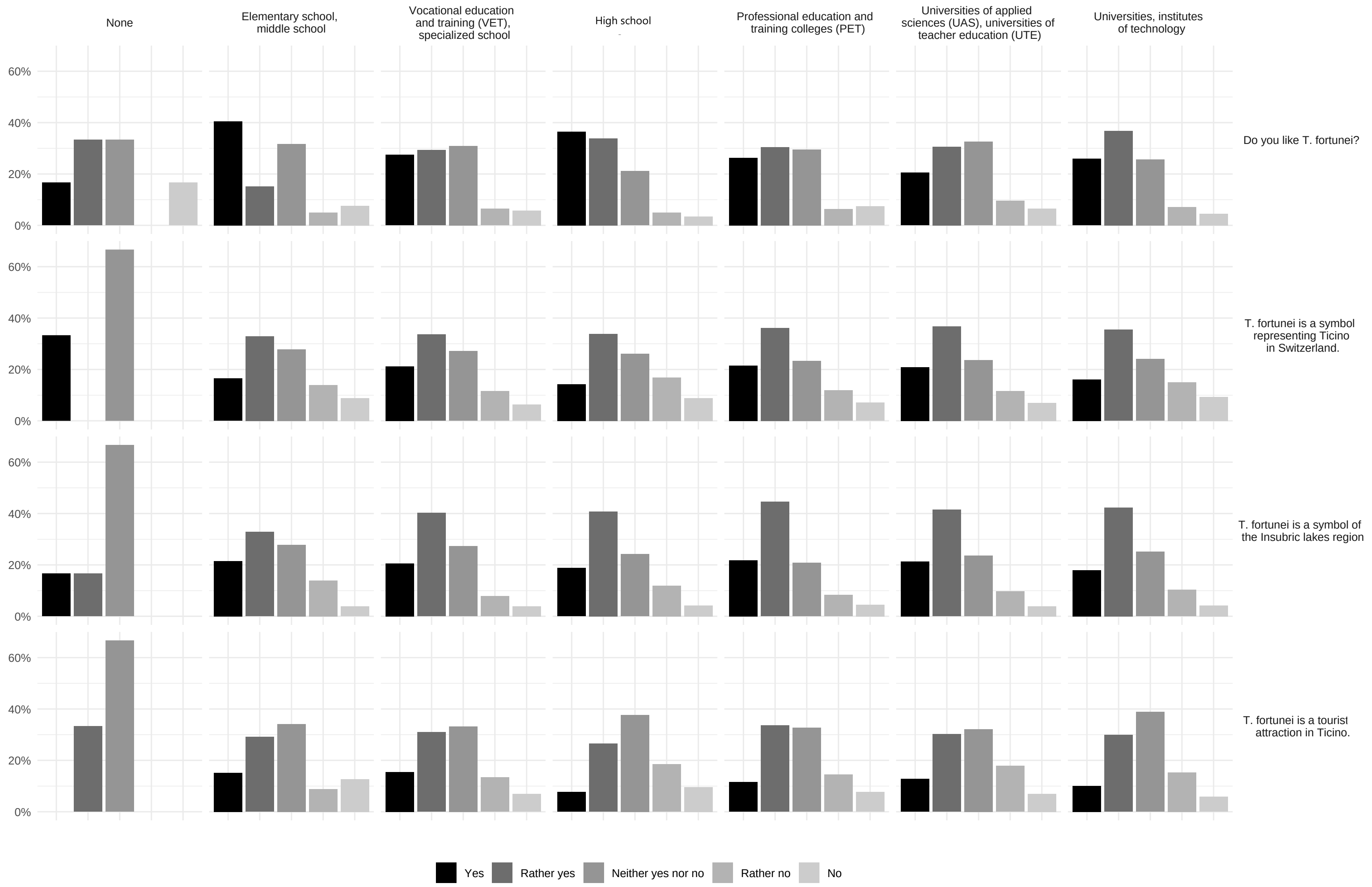

Perception according to the current occupation

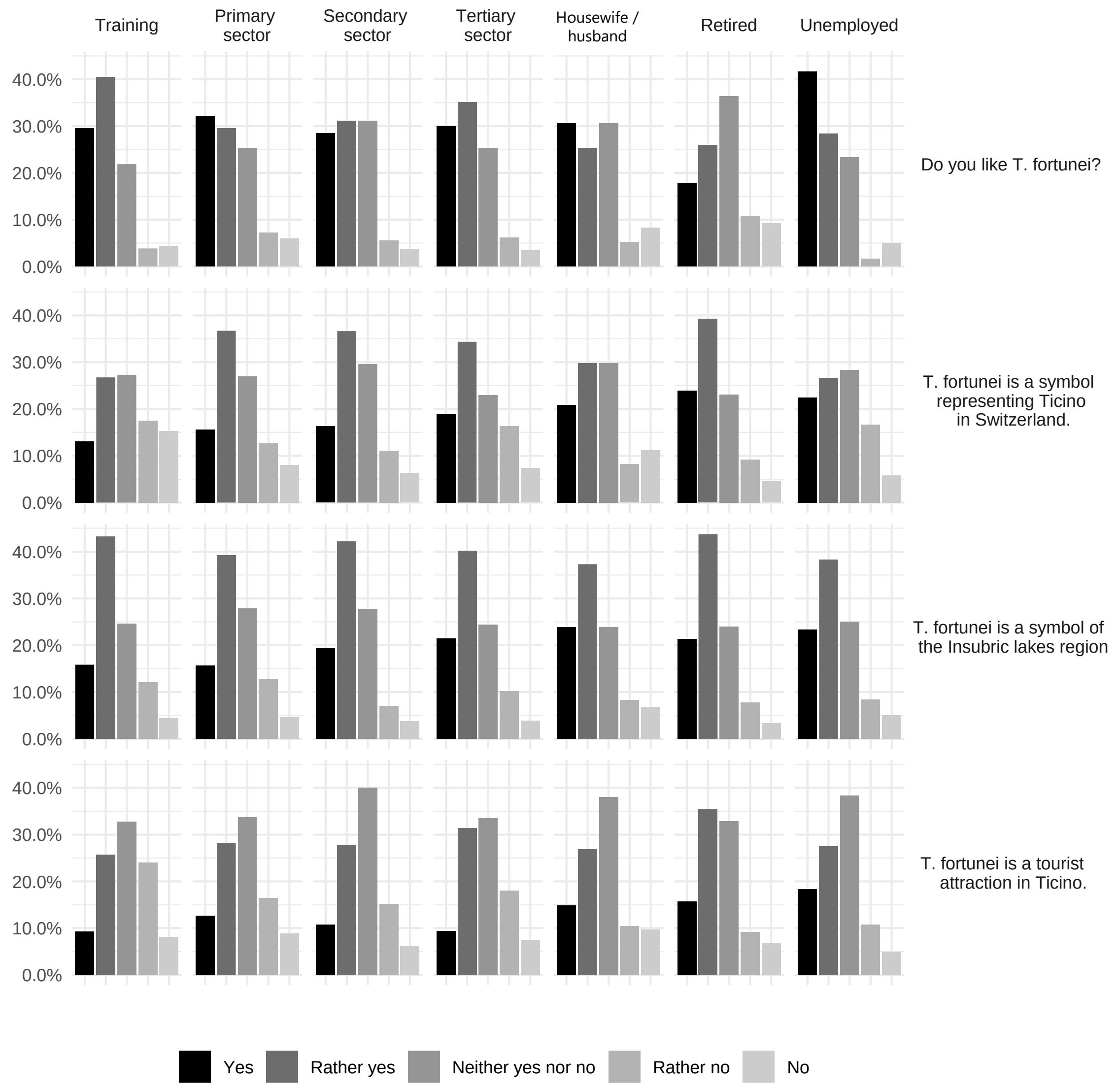

Perception according to the zone of residence (left) and among owners and non-owners of *T. fortunei* (right)

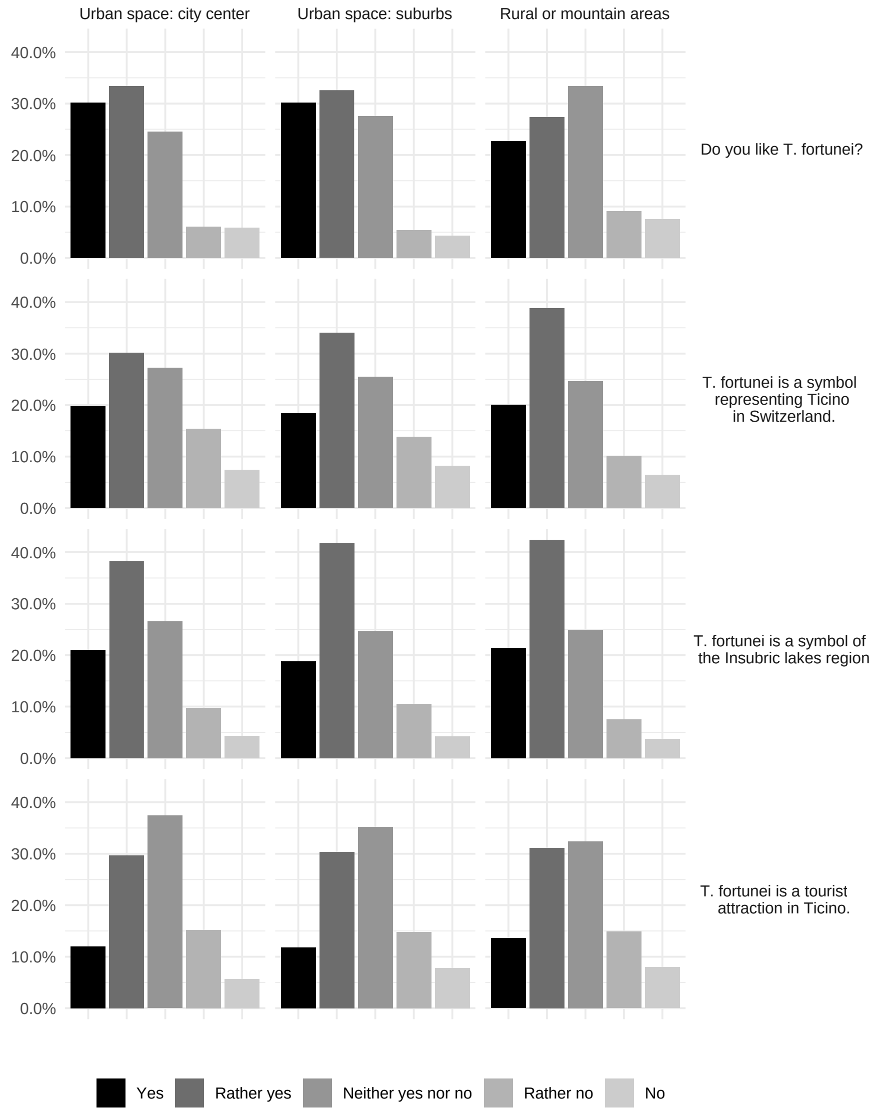

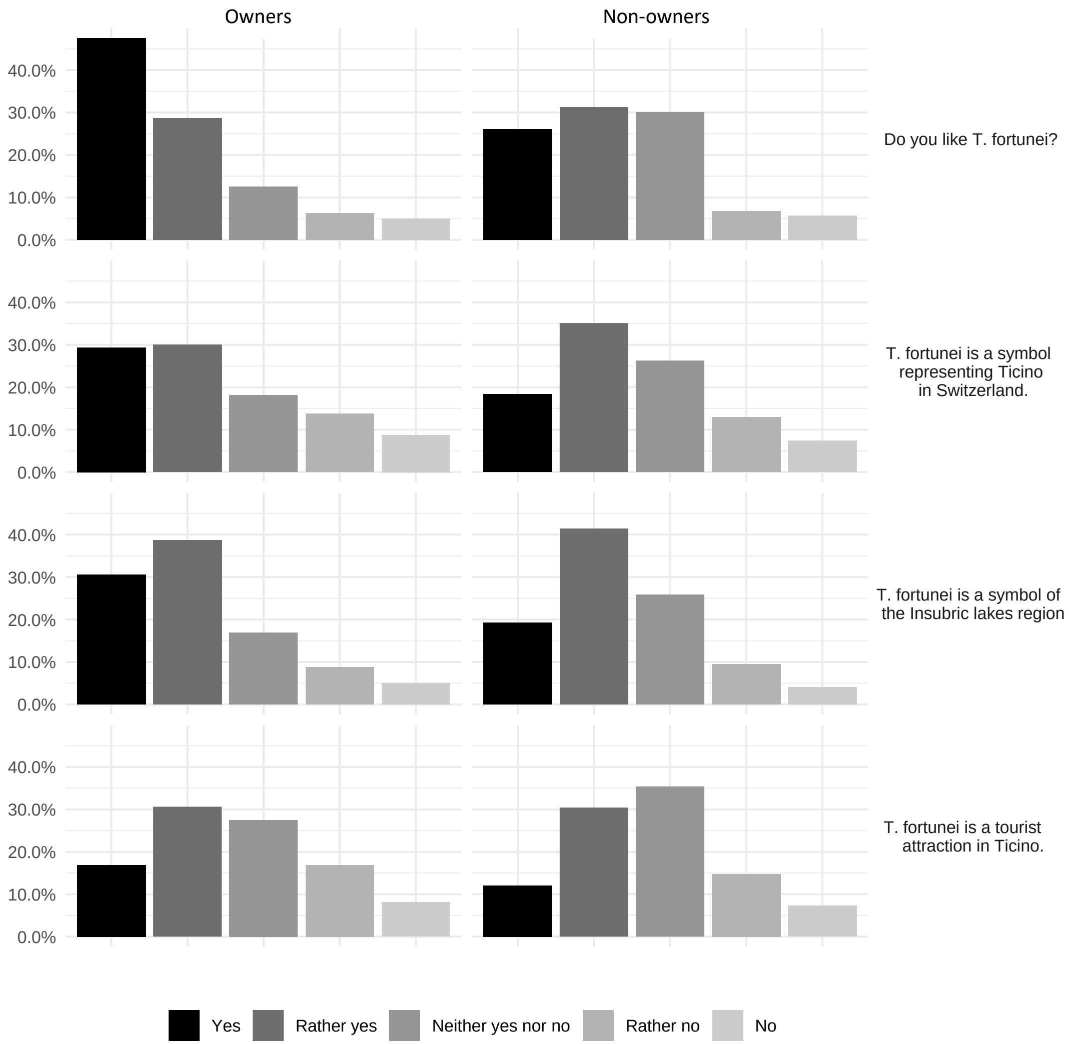

Importance of the information box

p. 25 Age class and Level of education

p. 26 Native language and Zone of residence

p. 27 Current occupation and Owners and non-owners of *T. fortunei*

Importance of the information box according to the age class (left) and the level of education (right)

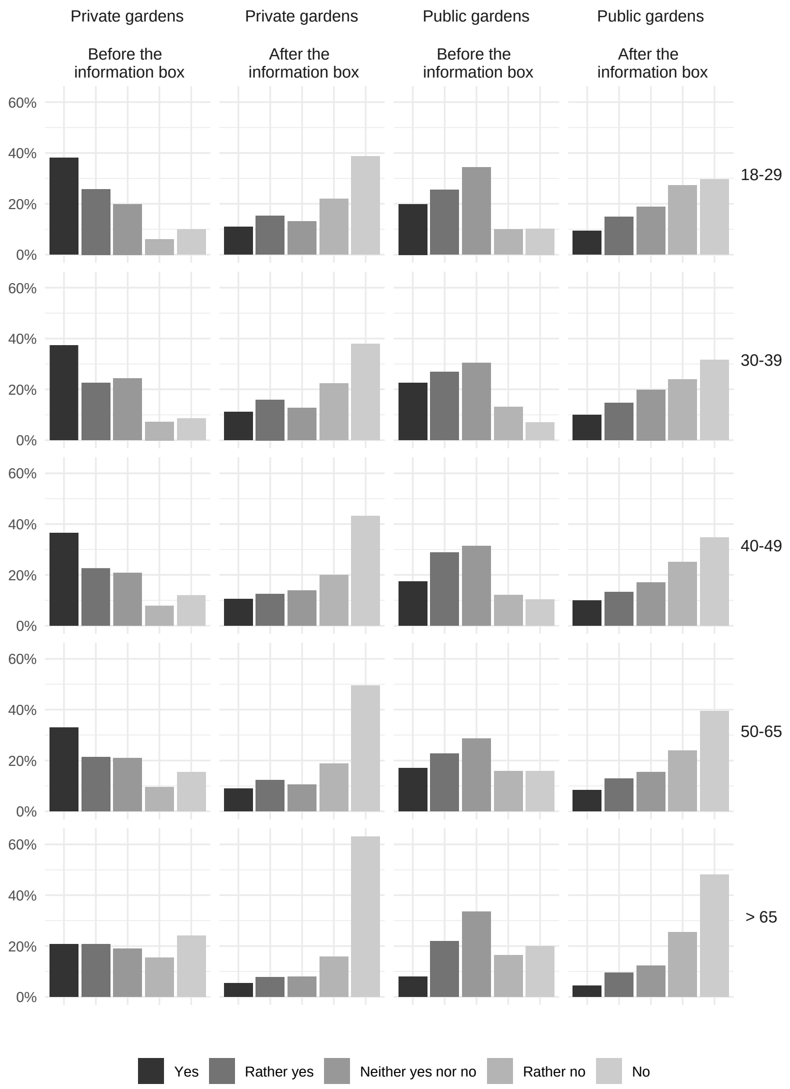

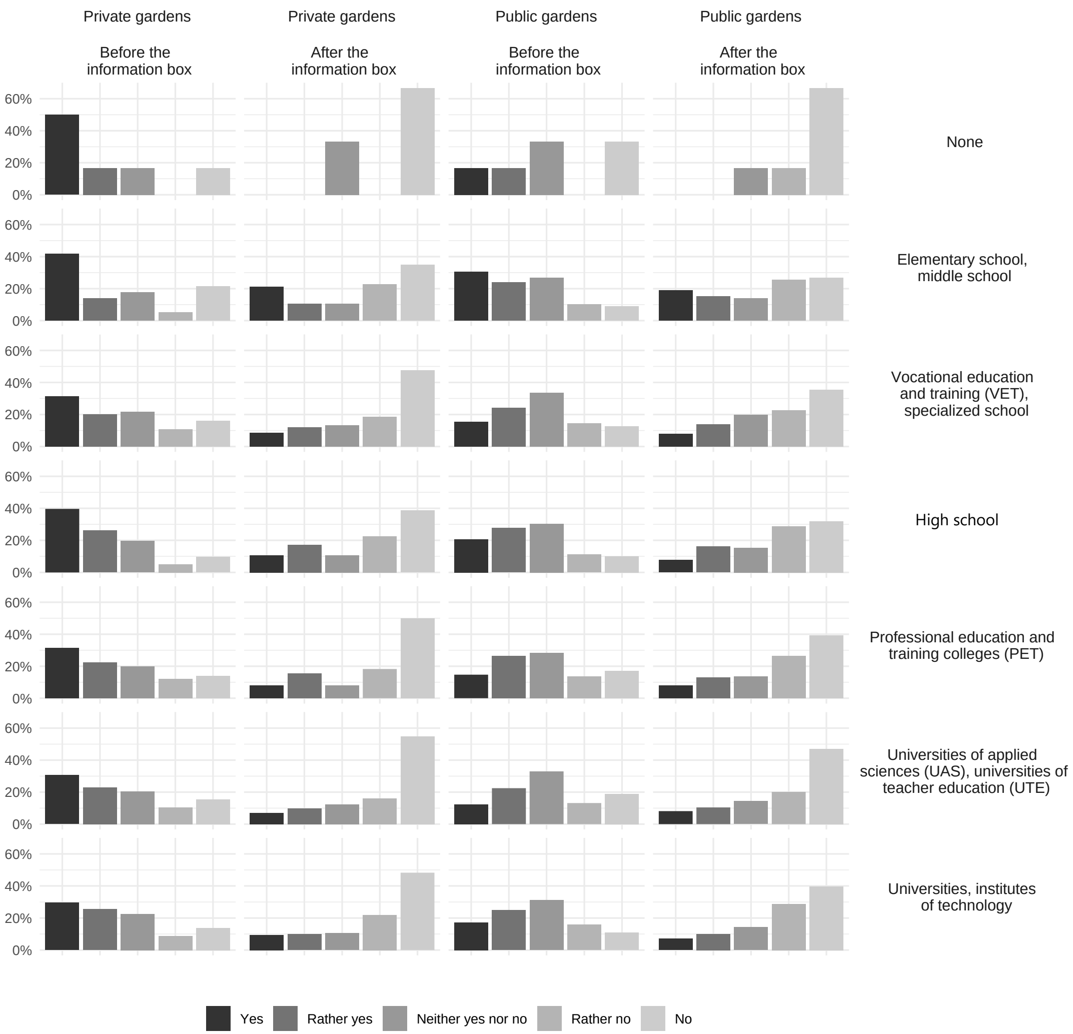

Importance of the information box according to the native language (left) and the zone of residence (right)

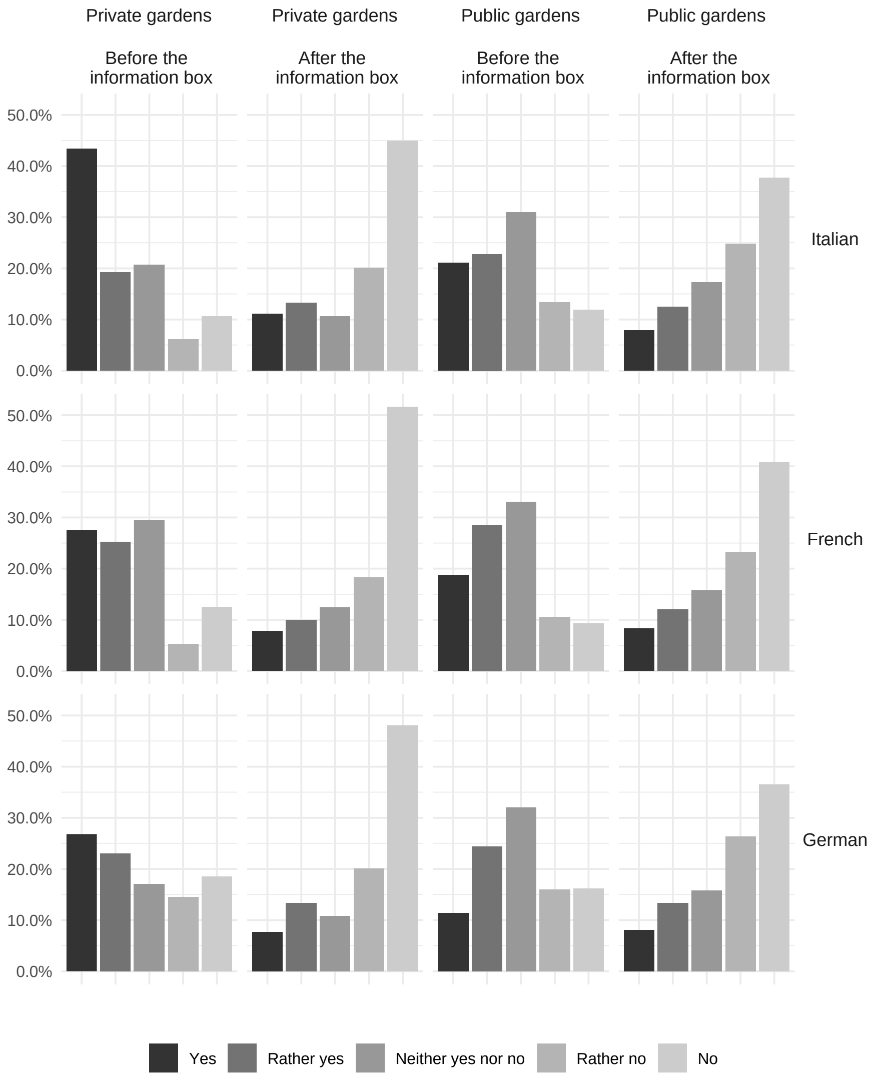

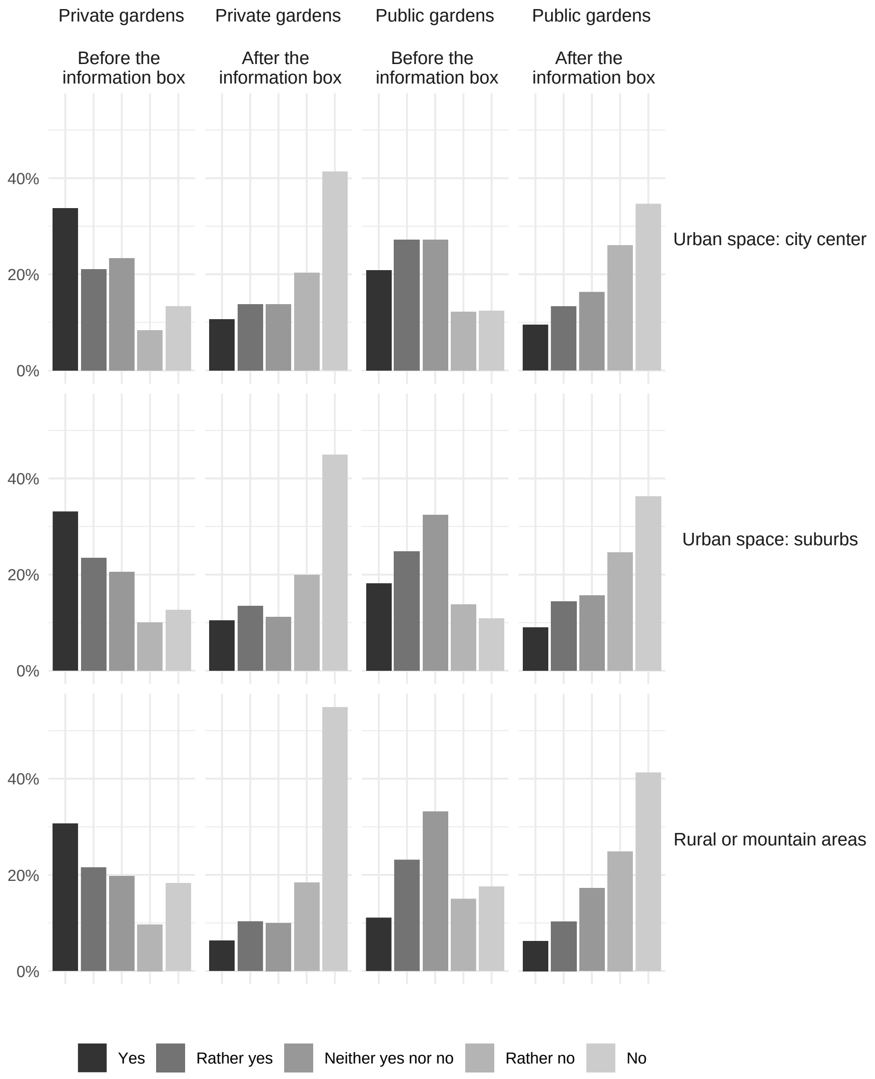

Importance of the information box according to the current occupation (left) and among owners and non-owners of *T. fortunei* (right)

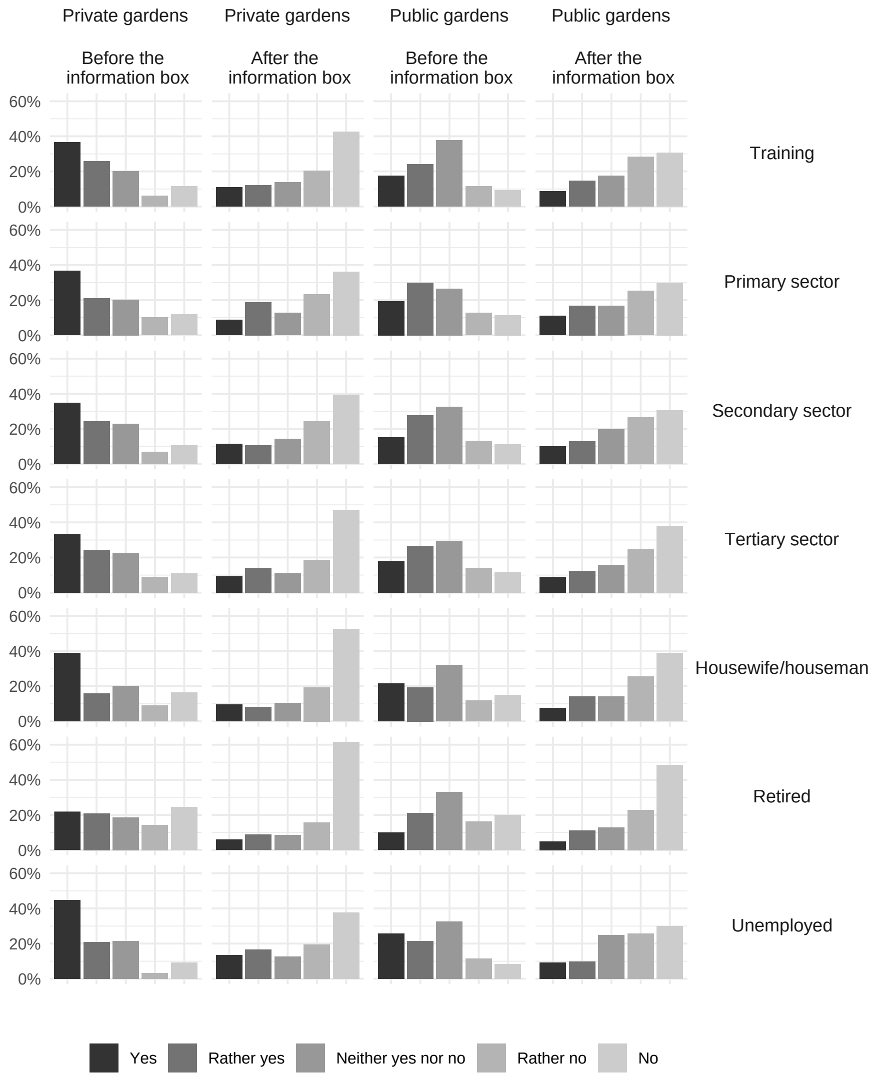

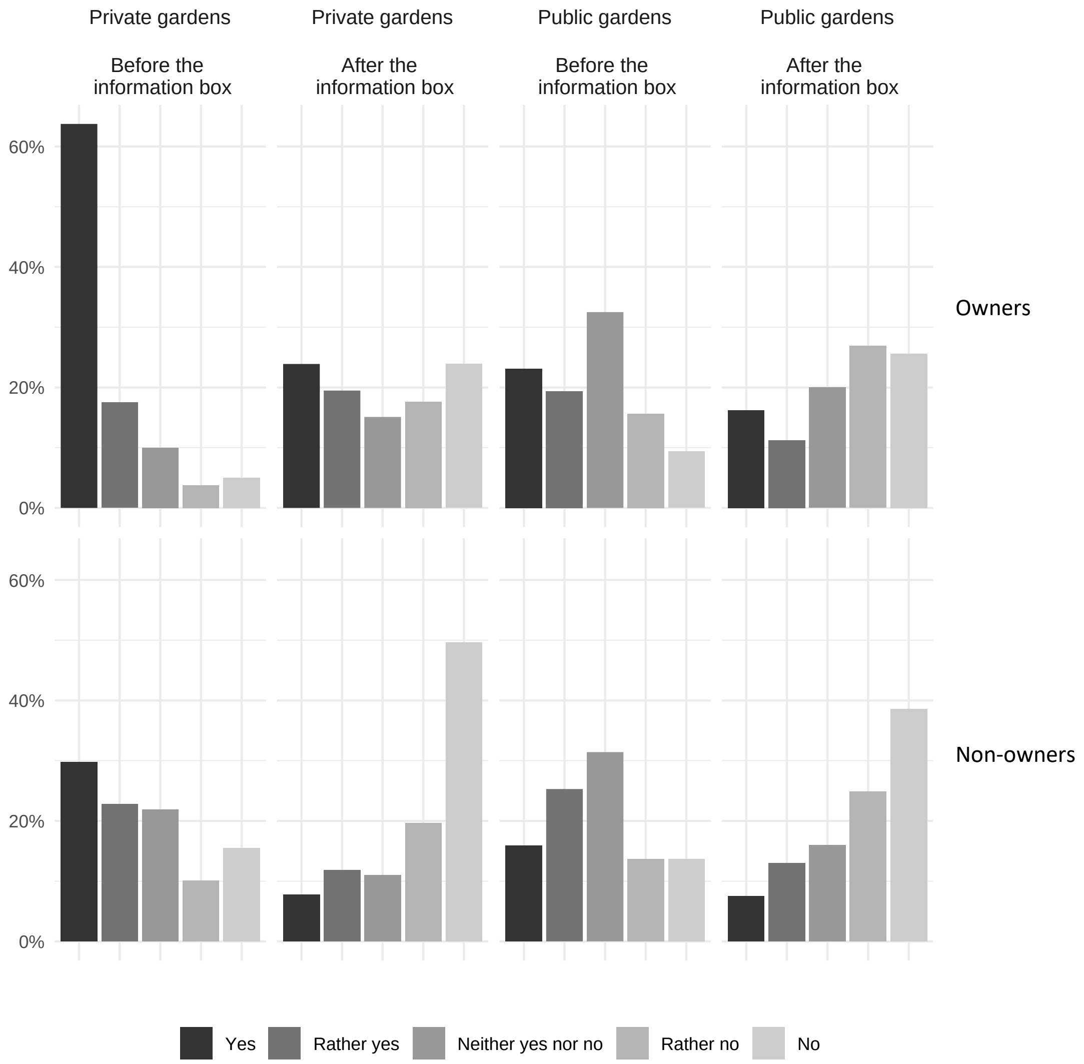

Preference questions

p. 29 Age class

p. 31 Level of education

p. 34 Native language

p. 36 Current occupation

p. 39 Owners and non-owners of *T. fortunei*

p. 41 Zone of residence

Preference according to the age class (1/2)

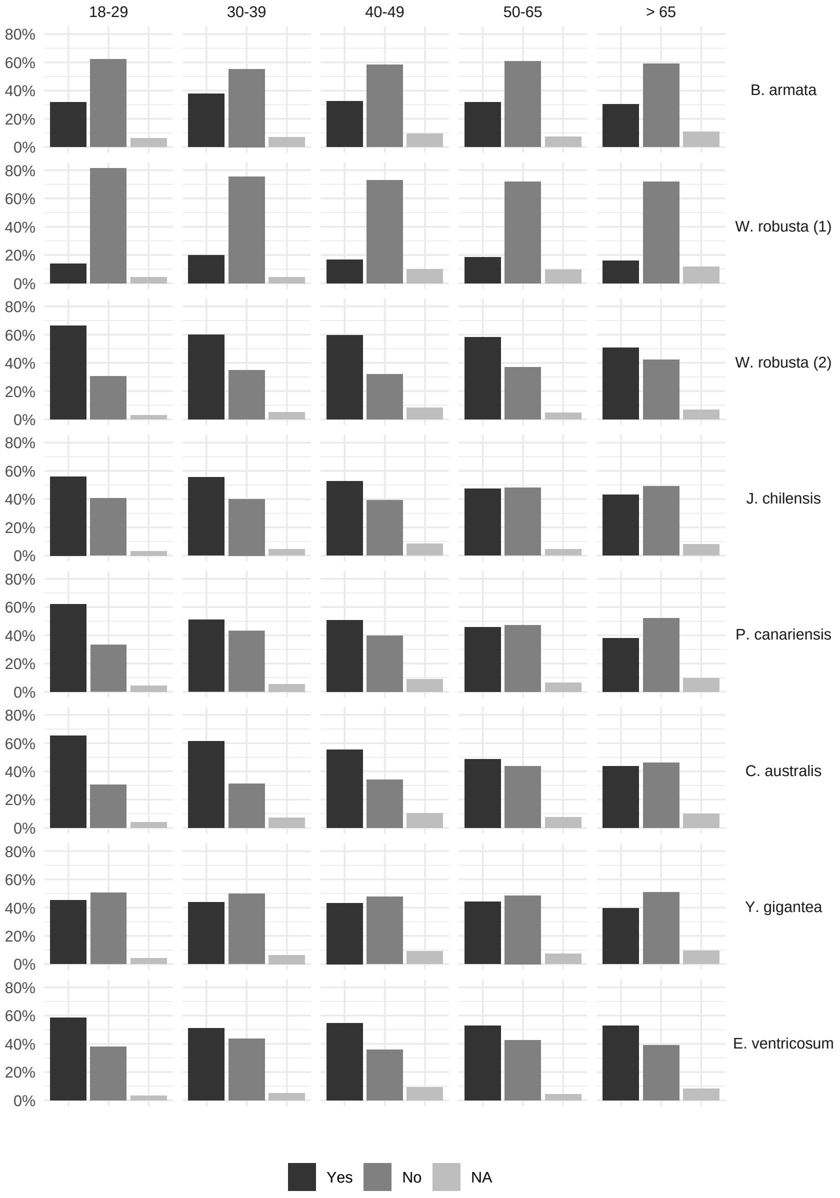

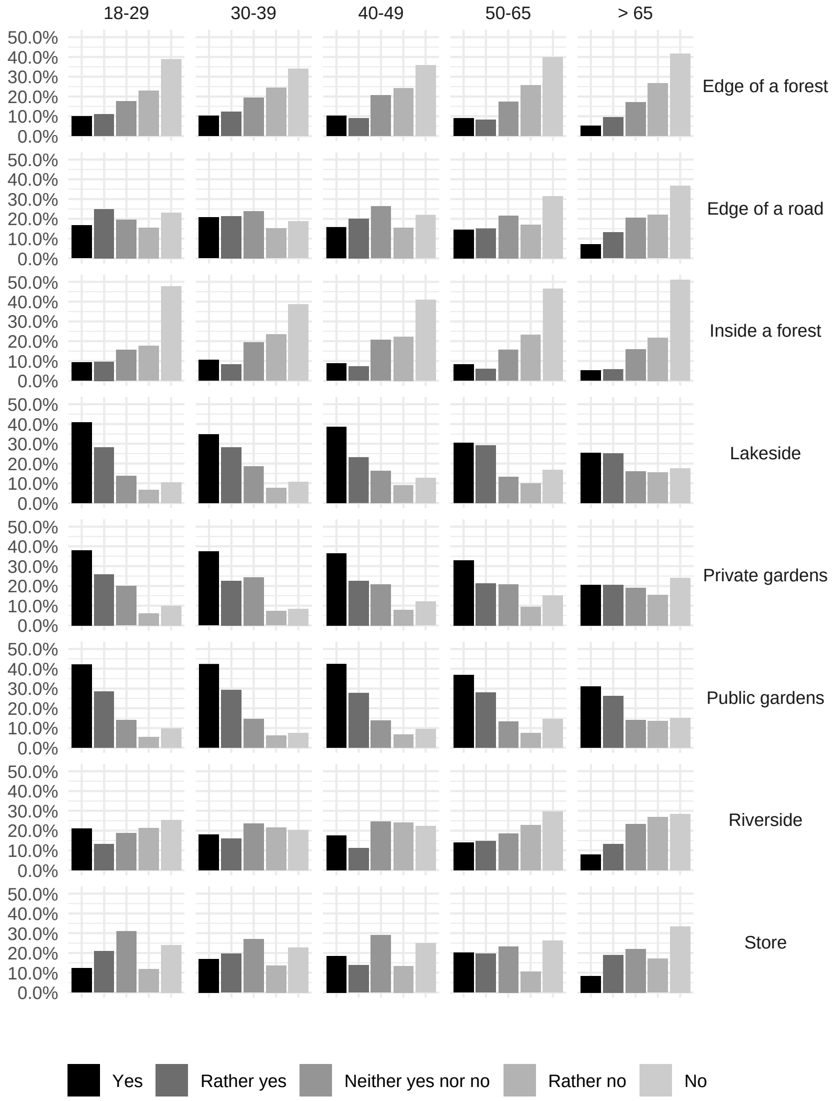

Preference according to the age class (2/2)

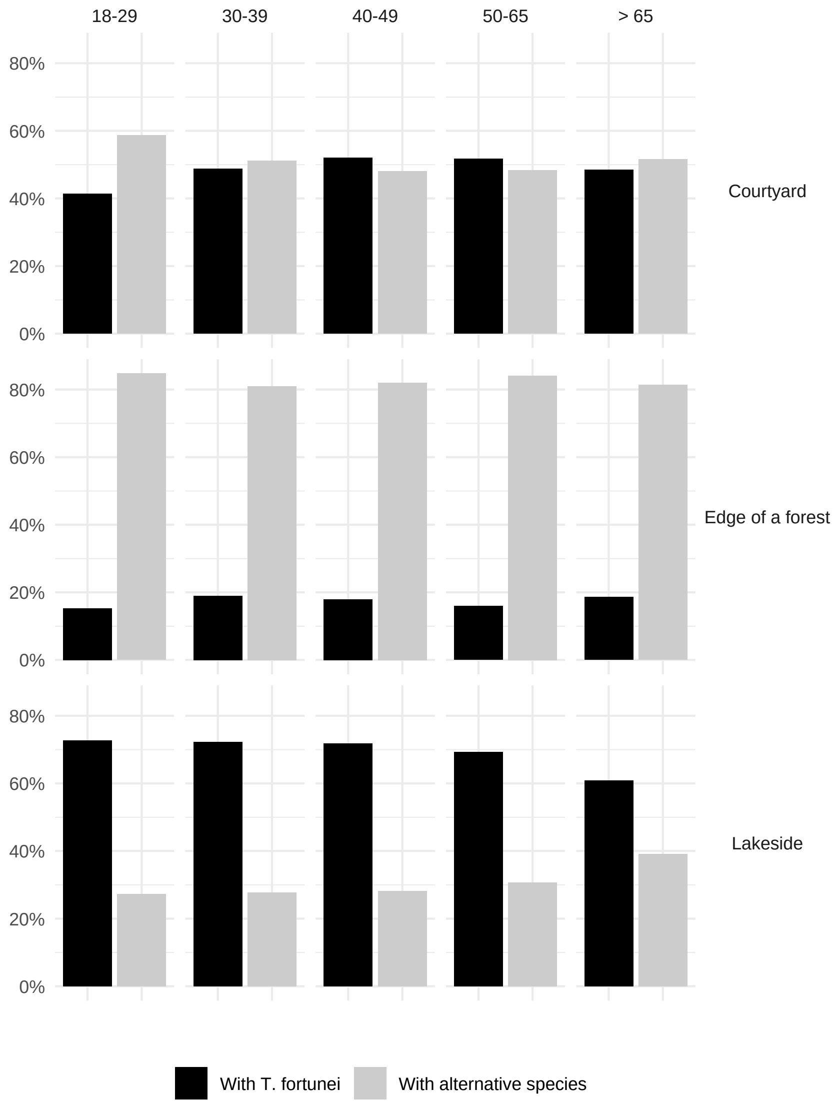

Preference according to the level of education (1/3)

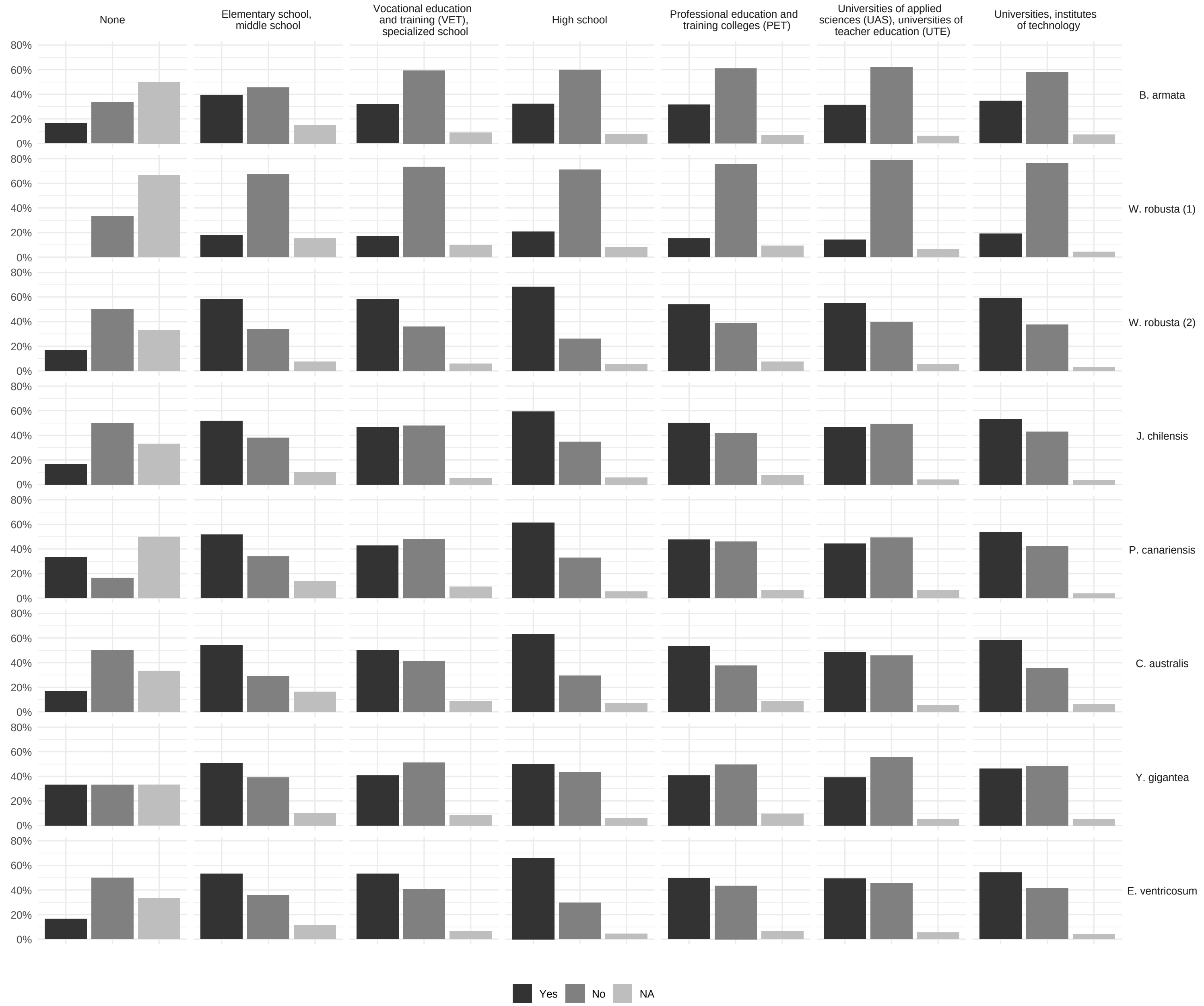

Preference according to the level of education (2/3)

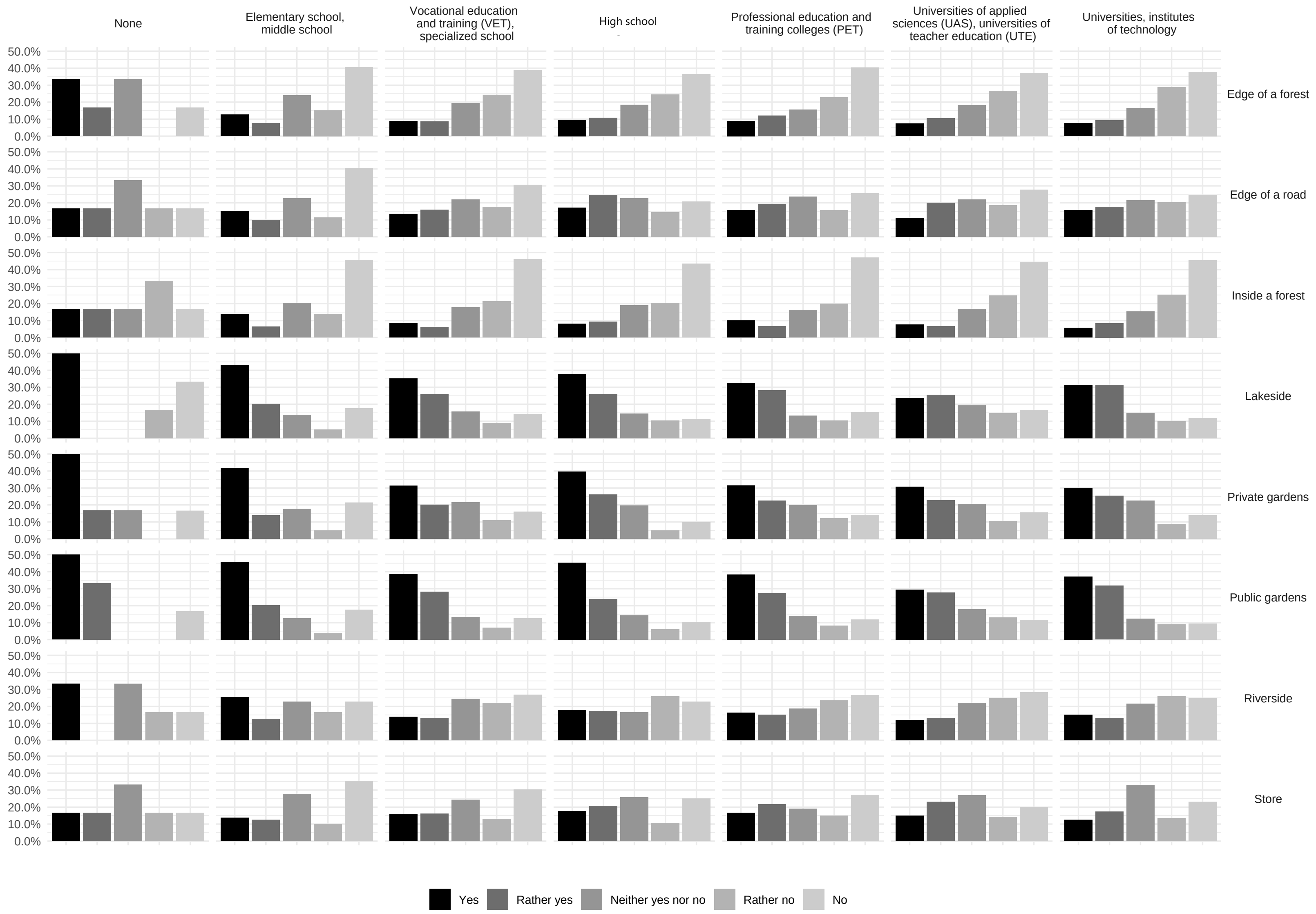

Preference according to the level of education (3/3)

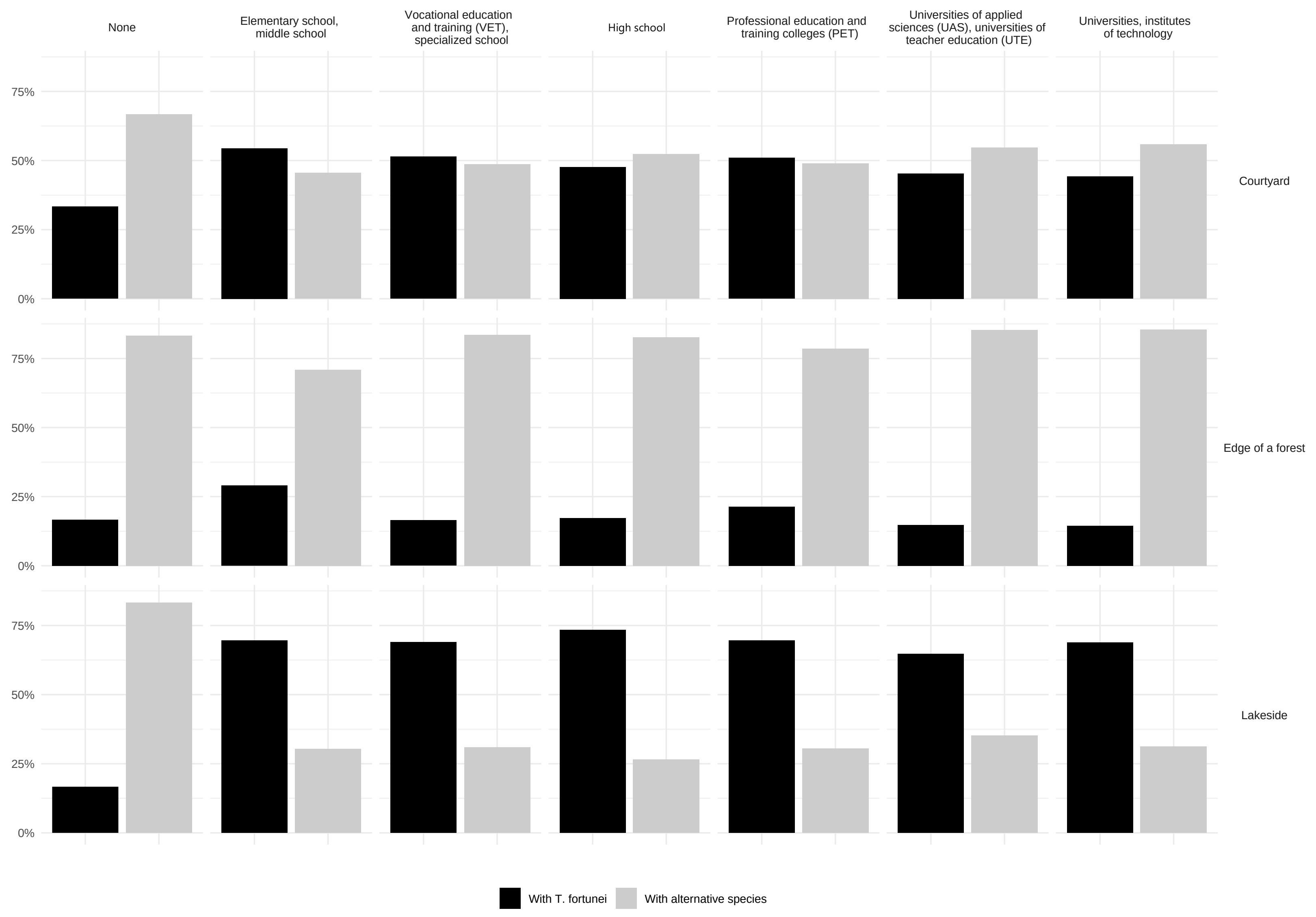

Preference according to the native language (1/2)

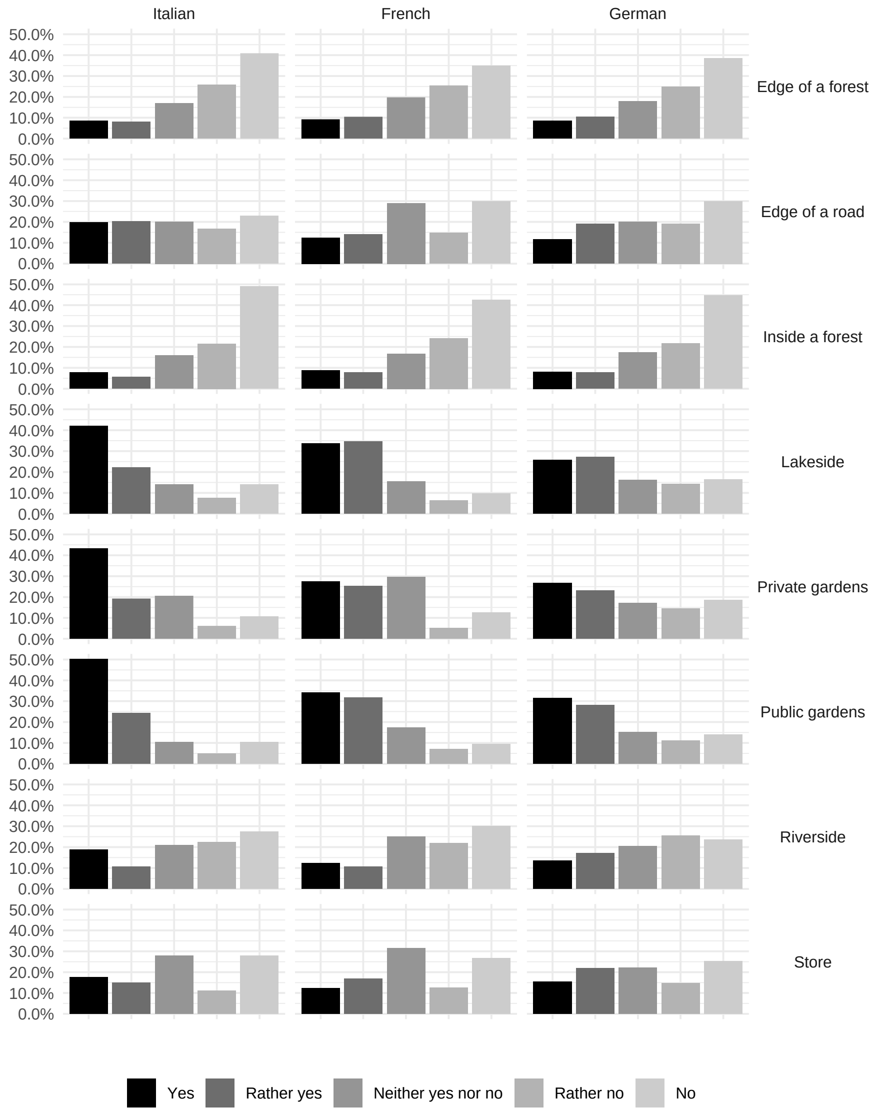

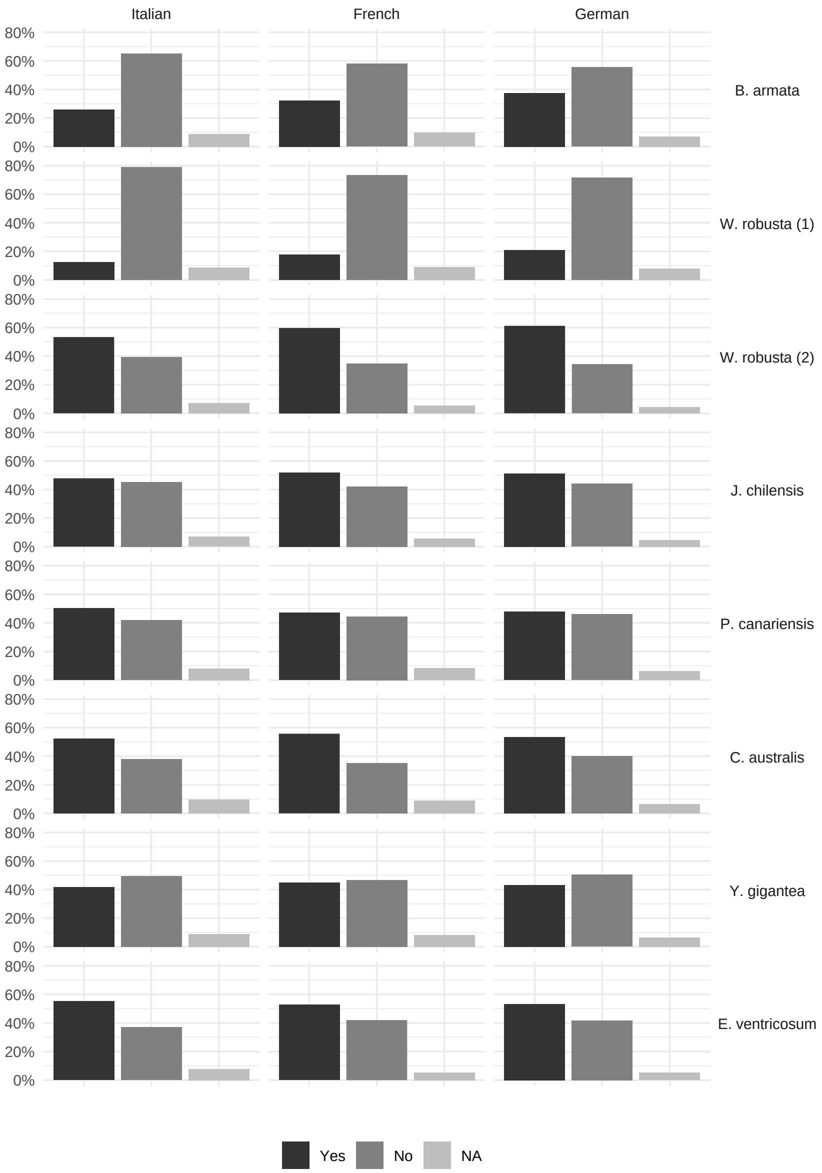

Preference according to the native language (2/2)

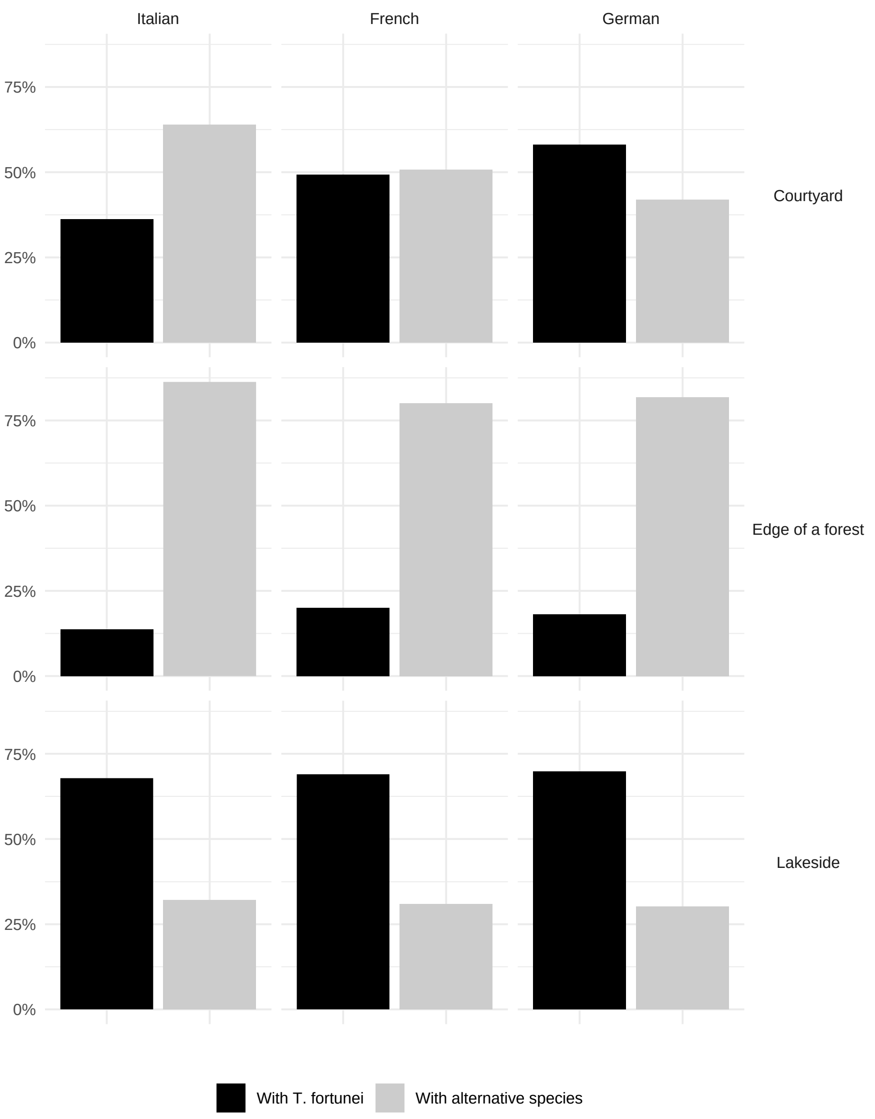

Preference according to the current occupation (1/3)

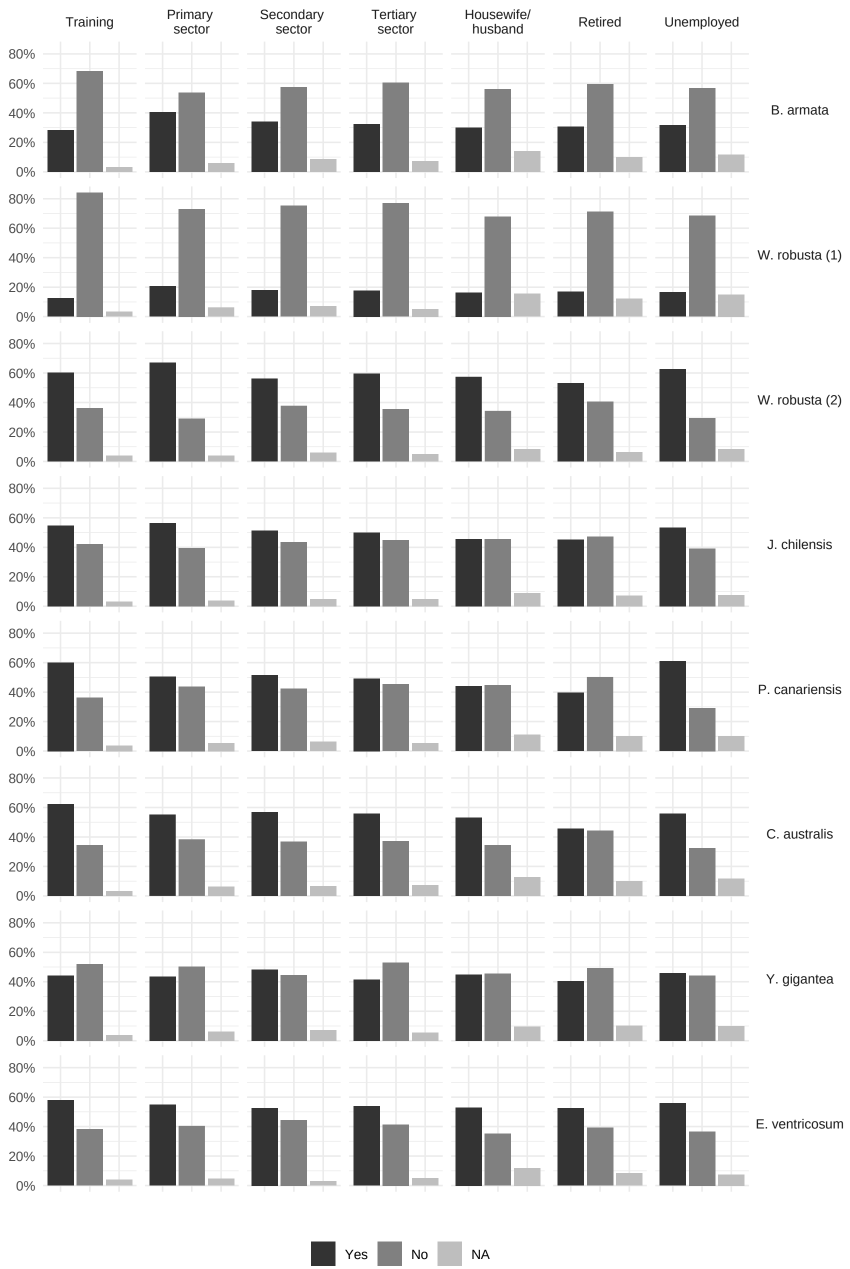

Preference according to the current occupation (2/3)

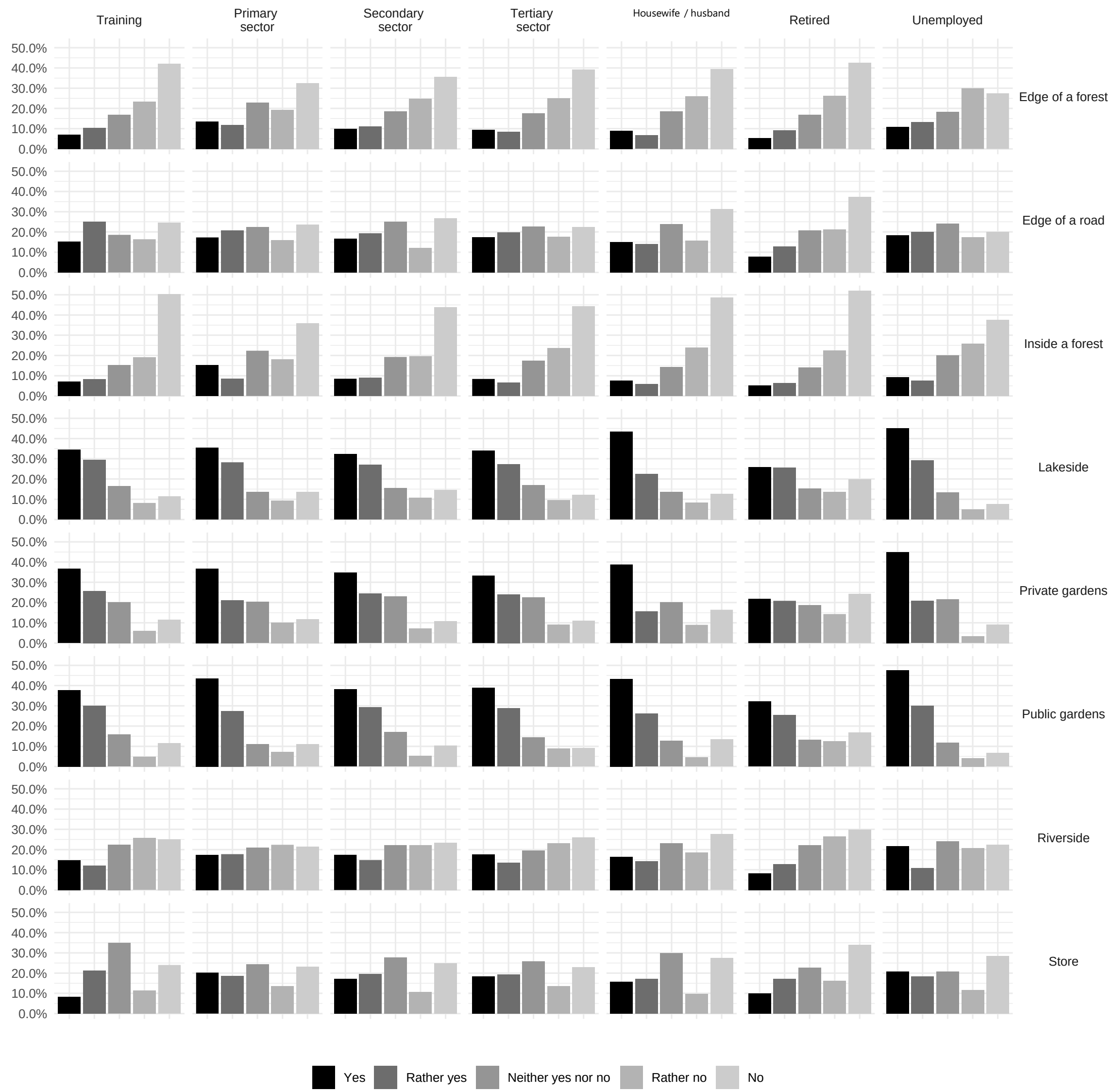

Preference according to the current occupation (3/3)

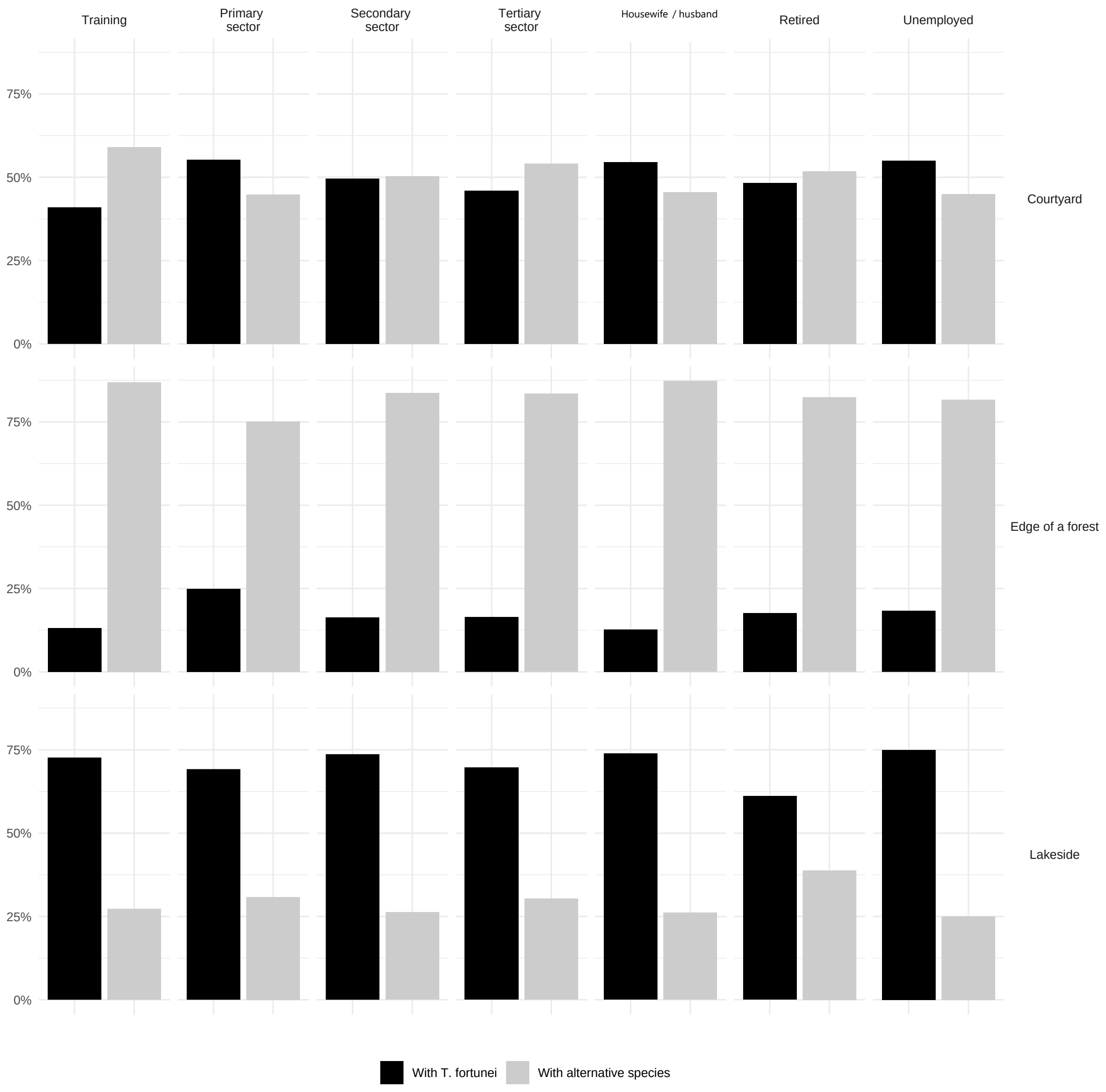

Preference among owners and non-owners of *T. fortunei* (1/2)

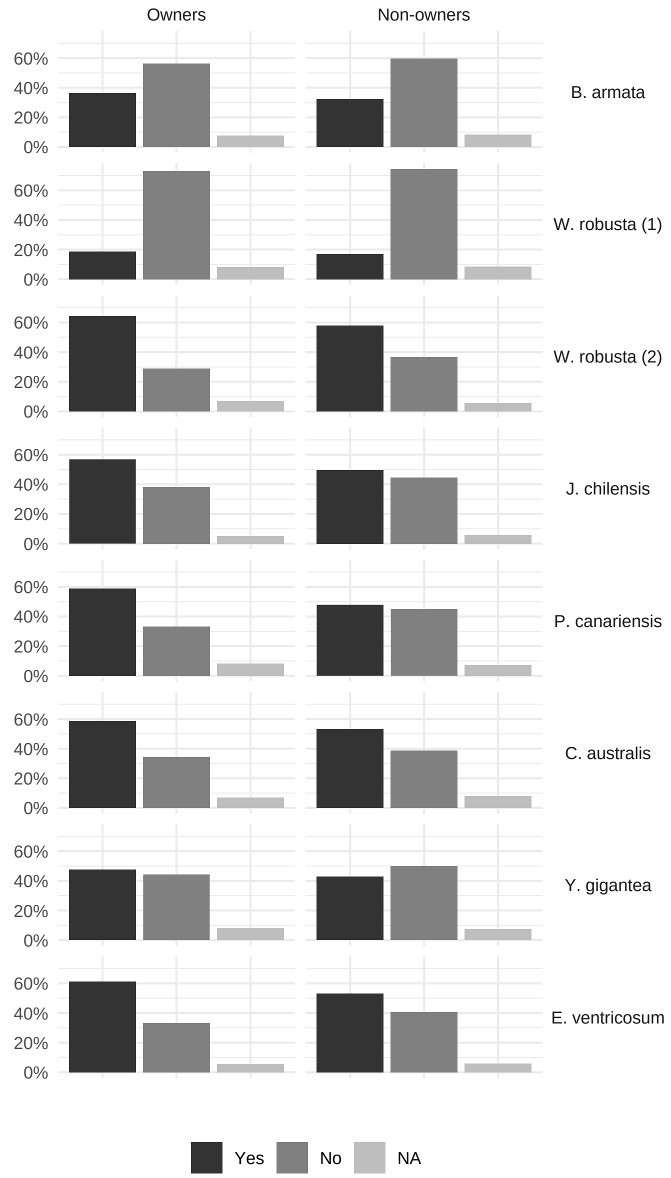

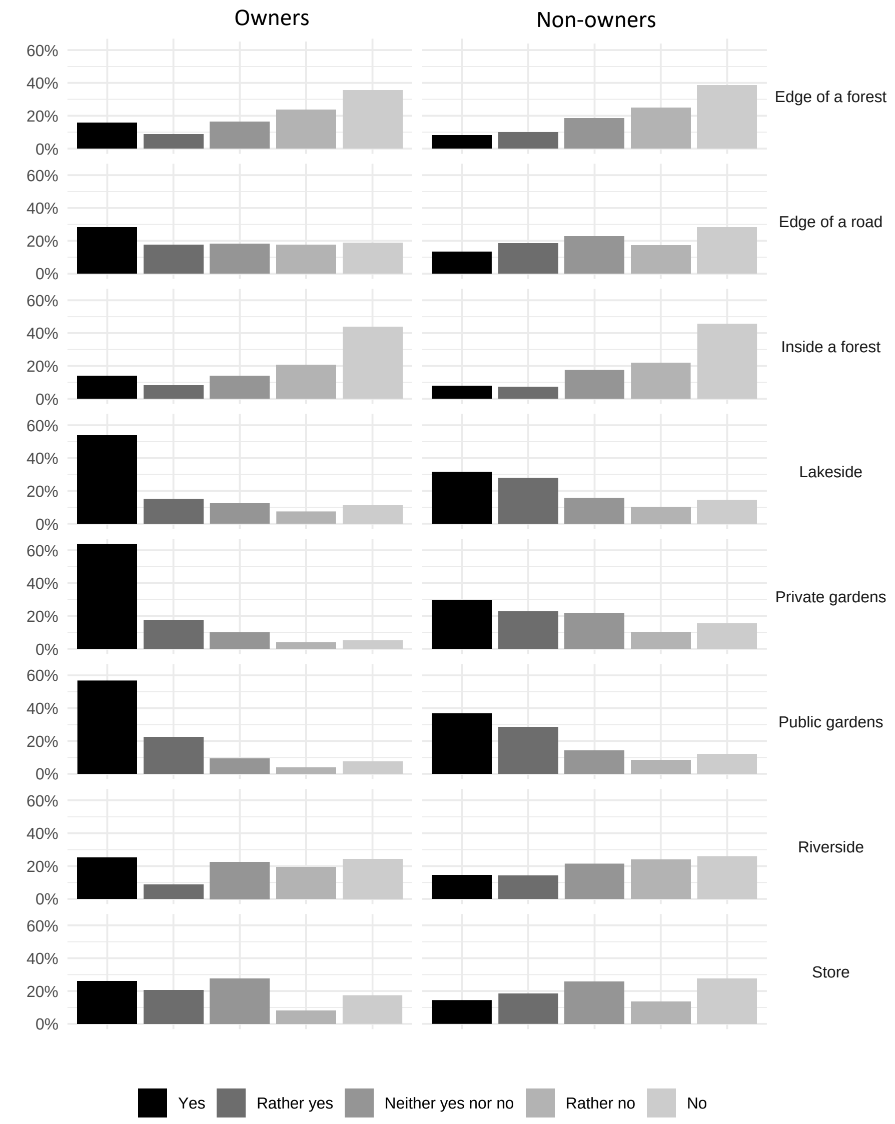

Preference among owners and non-owners of *T. fortunei* (2/2)

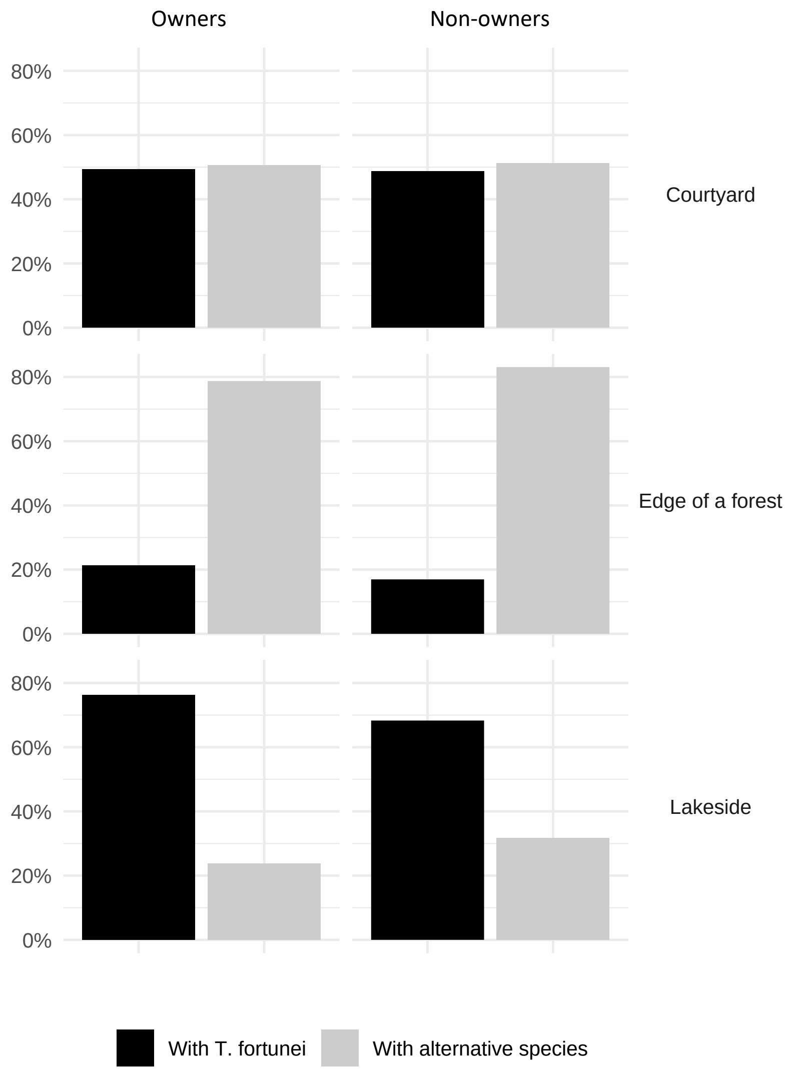

Preference according to the zone of residence (1/2)

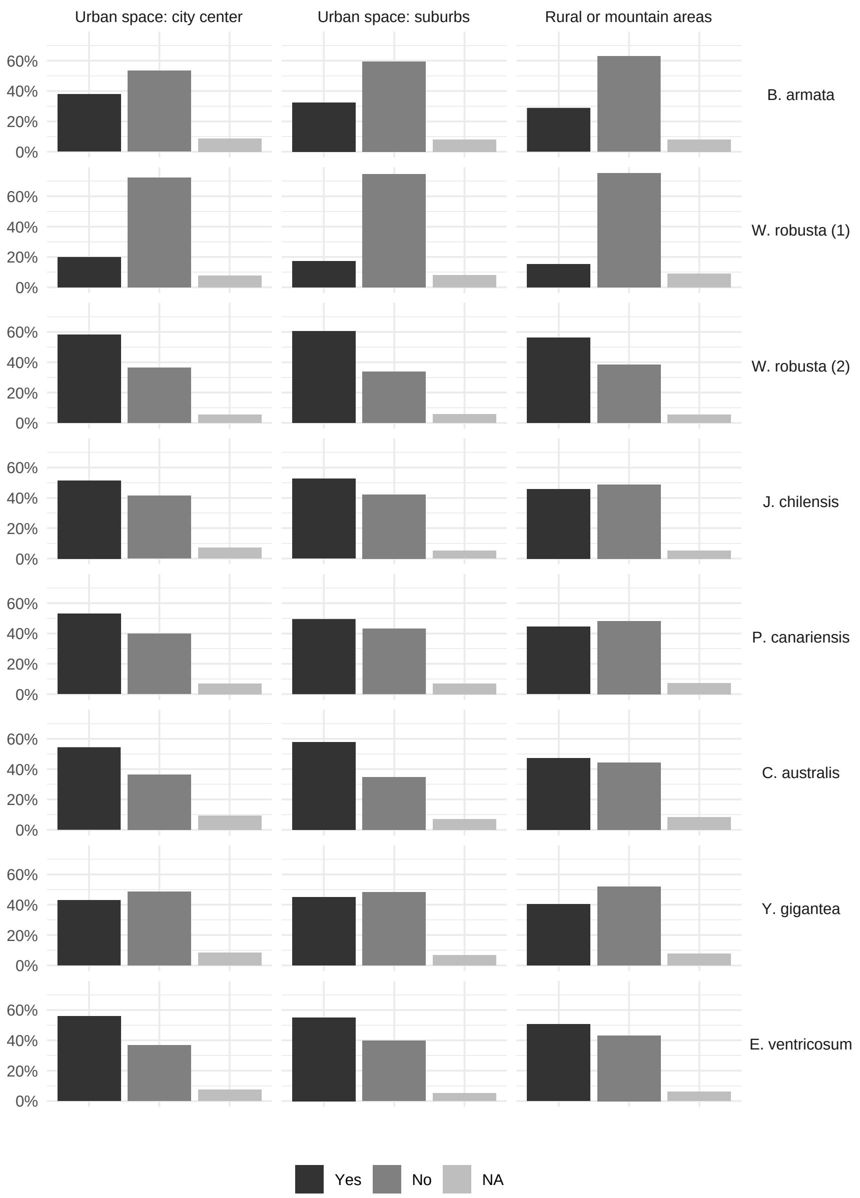

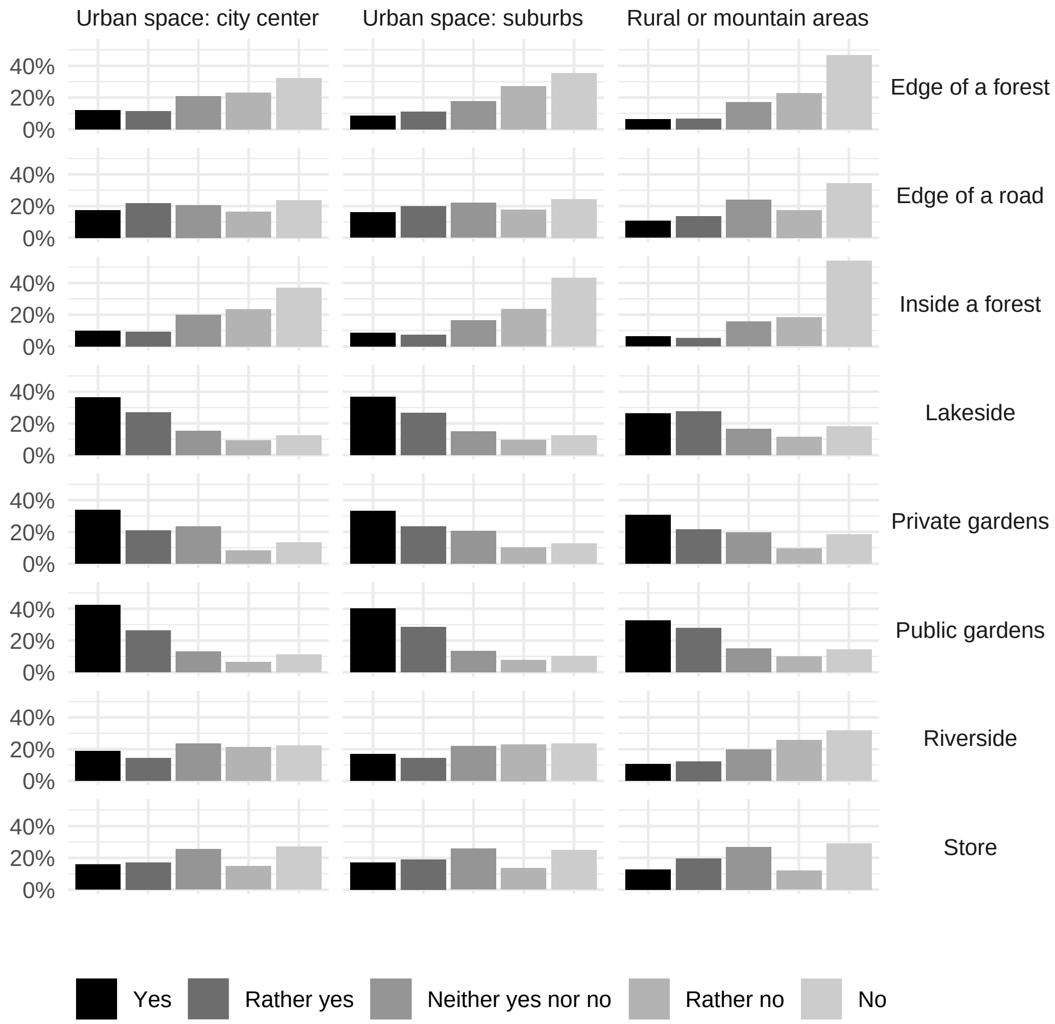

Preference according to the zone of residence (2/2)

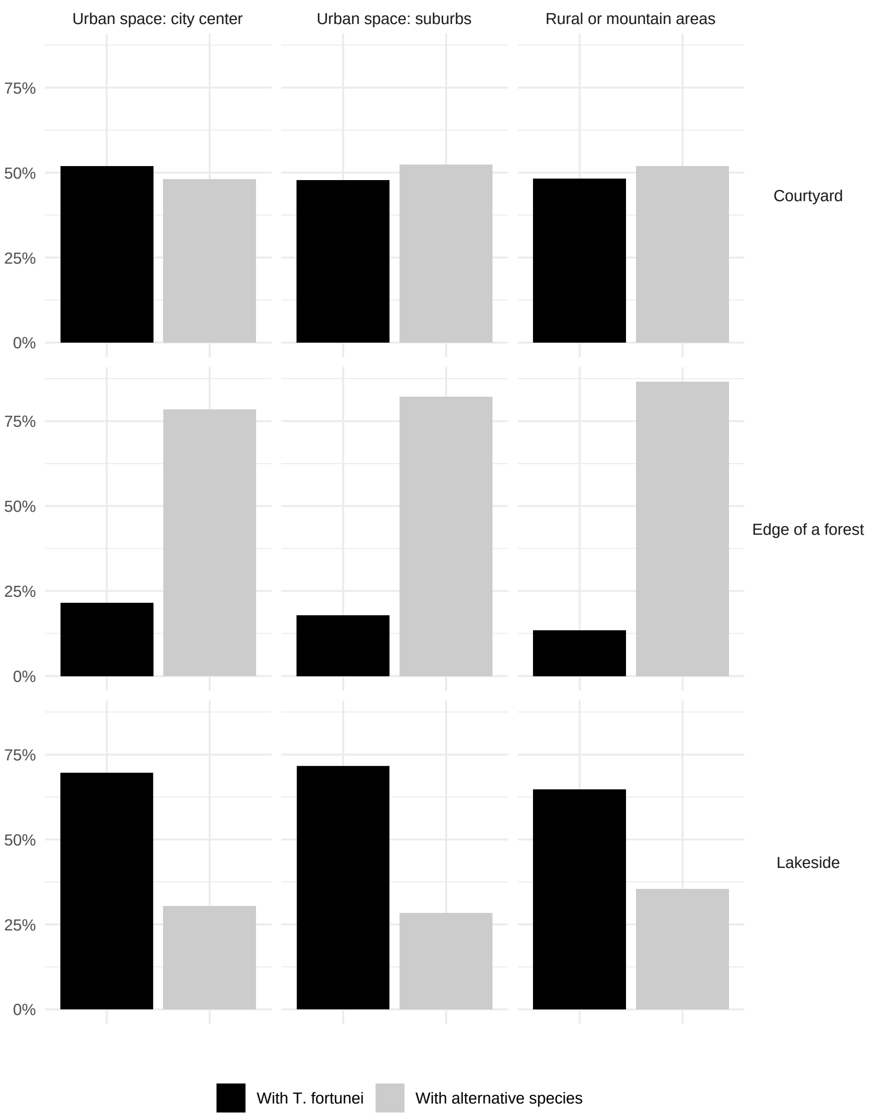

Acceptance of the management options

p. 44 Age class

p. 45 Level of education

p. 46 Current occupation

p. 47 Owners and non-owners of *T. fortunei*

p. 48 Zone of residence

Acceptance of the management options according to the age class

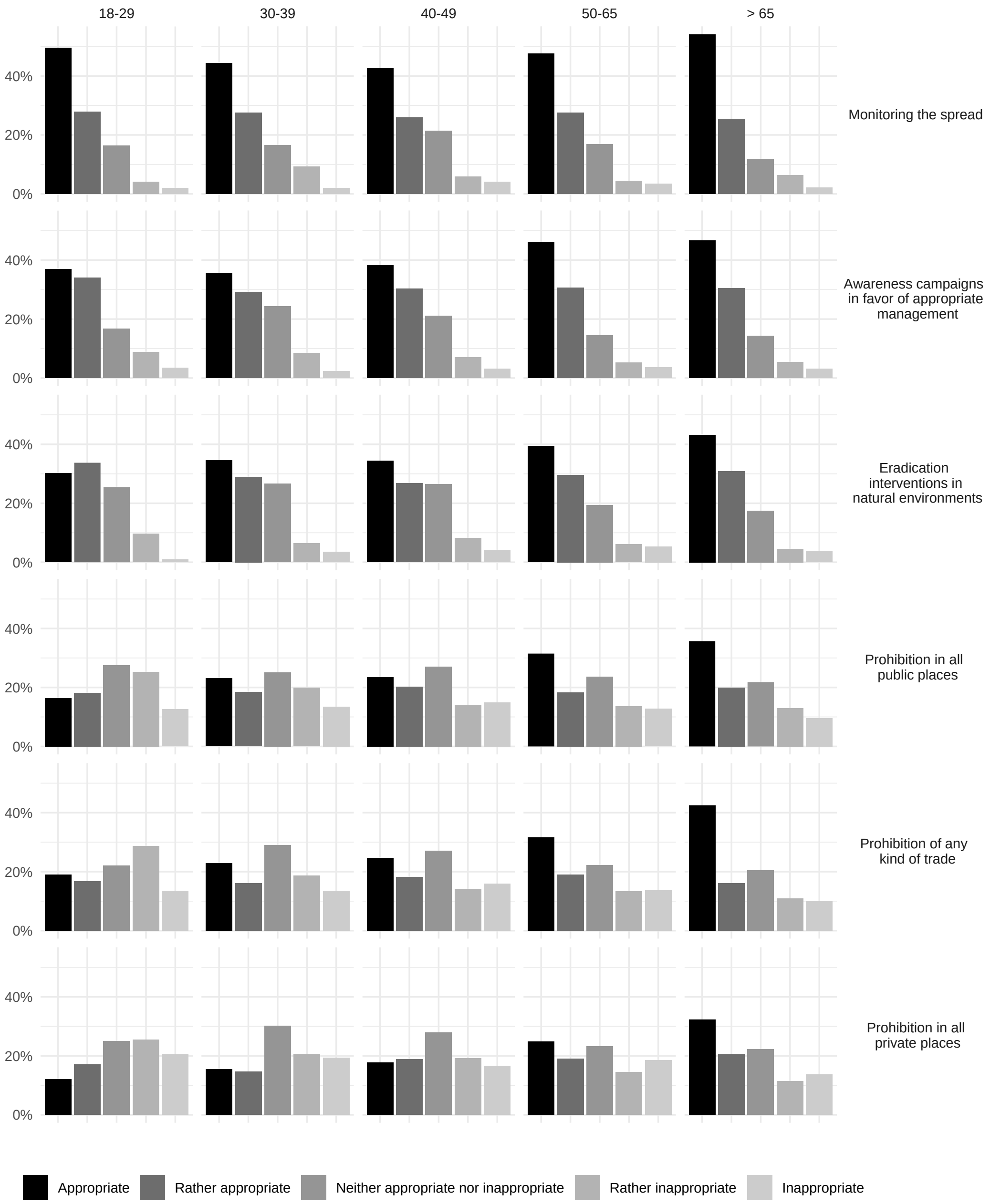

Acceptance of the management options according to the level of education

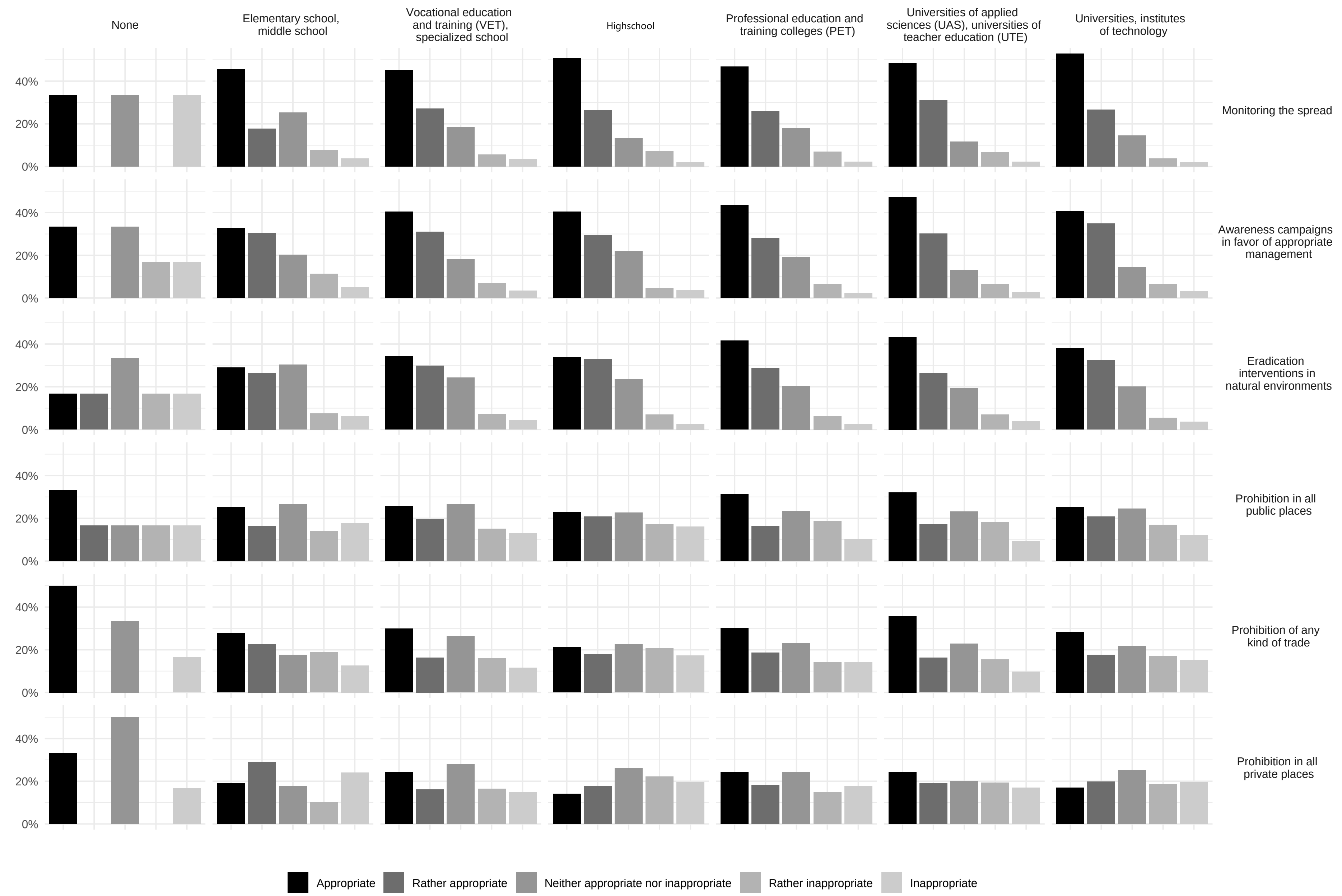

Acceptance of the management options according to the current occupation

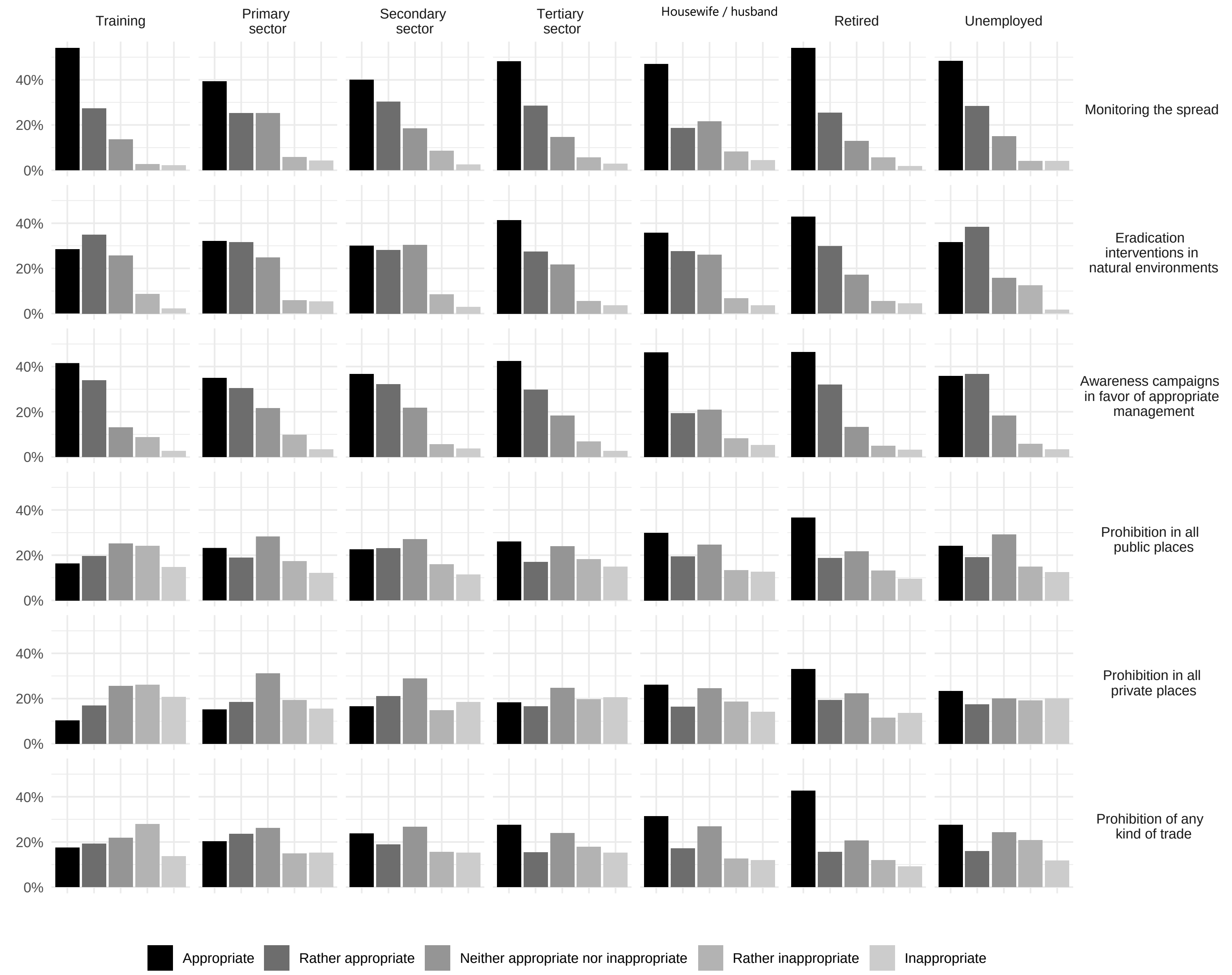

Acceptance of the management options among owners and non-owners of *T. fortunei*

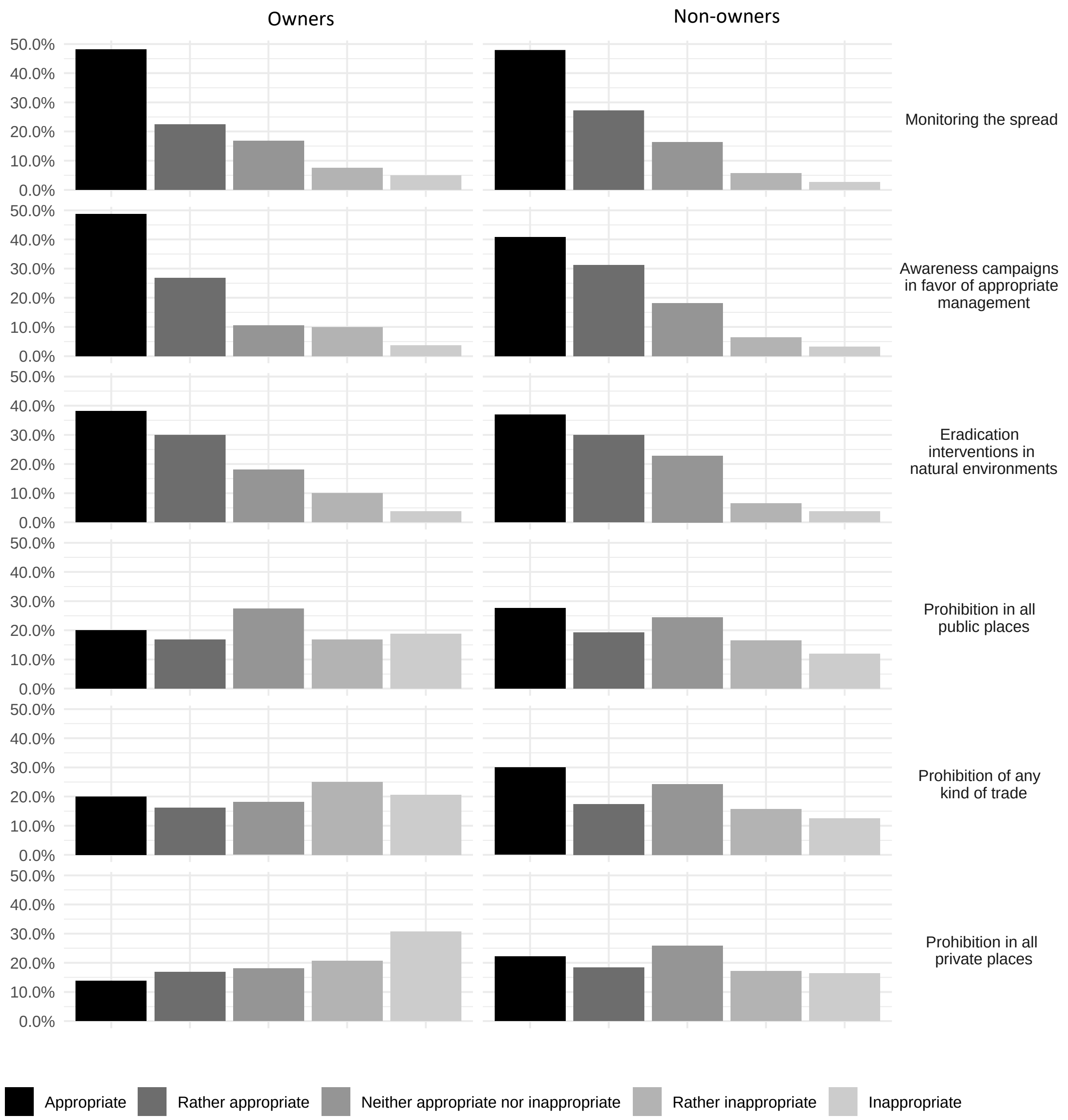

Acceptance of the management options according to the zone of residence

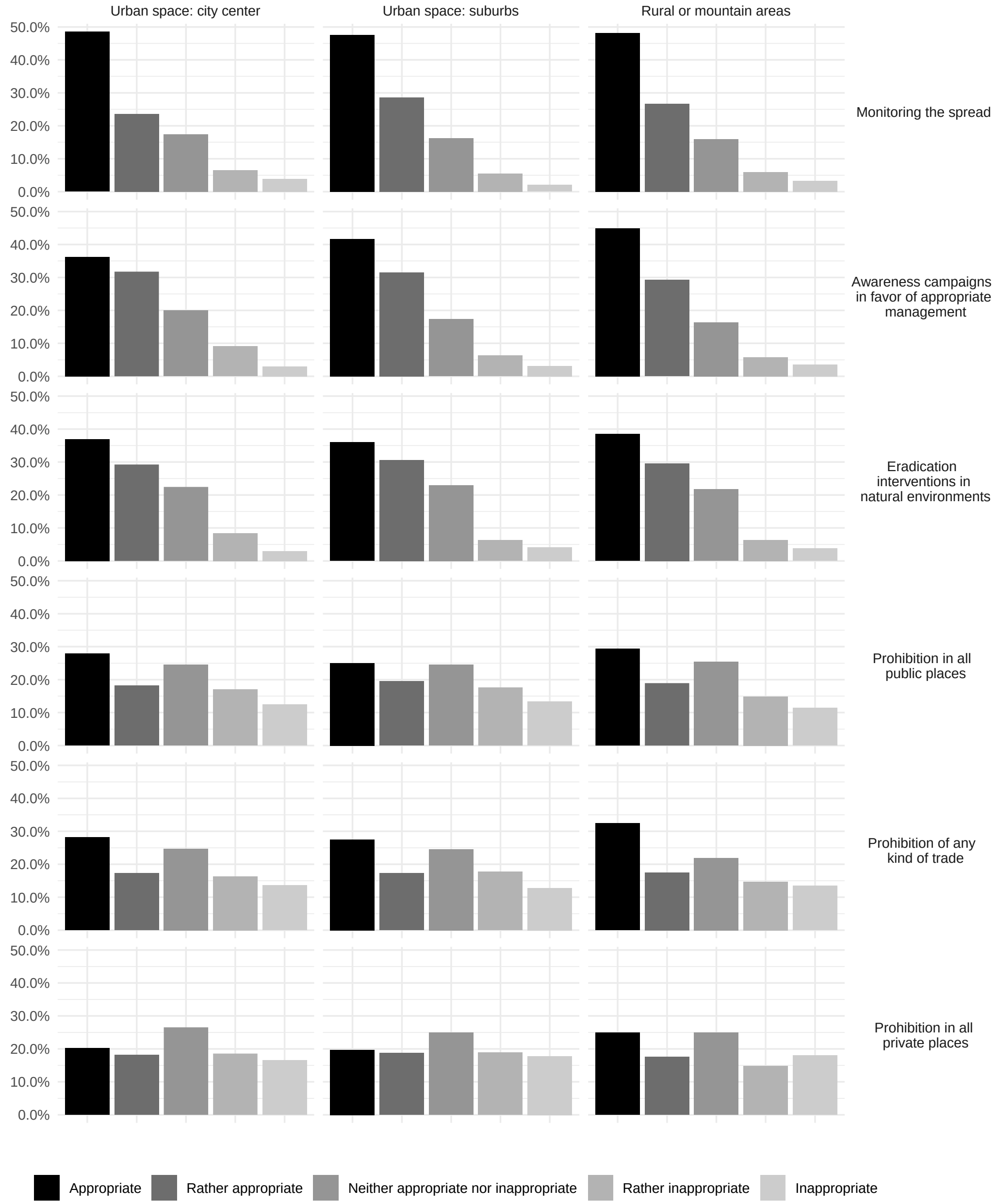

Supplement: Supplementary file 6 — Supplementary Materials S6 [file 267_2022_1646_MOESM6_ESM.pdf]
